# High-resolution analysis of condition-specific regulatory modules in *Saccharomyces cerevisiae*

**DOI:** 10.1186/gb-2008-9-1-r2

**Published:** 2008-01-03

**Authors:** Hun-Goo Lee, Hyo-Soo Lee, Sang-Hoon Jeon, Tae-Hoon Chung, Young-Sung Lim, Won-Ki Huh

**Affiliations:** 1 Department of Bioinformatics, Dong-a Seetech Research Institute, Seoul 135-010, Republic of Korea; 2 School of Biological Sciences and Research Center for Functional Cellulomics, Institute of Microbiology, Seoul National University, Seoul 151-747, Republic of Korea; 3 Computational Biology Division, TGEN, N 5th St, Phoenix, Arizona 85004, USA

## Abstract

We present an approach for identifying condition-specific regulatory modules by using separate units of gene expression profiles along with ChIP-chip and motif data from *Saccharomyces cerevisiae*. By investigating the unique and common features of the obtained condition-specific modules, we detected several important properties of transcriptional network reorganization. Our approach reveals the functionally distinct coregulated submodules embedded in a coexpressed gene module and provides an effective method for identifying various condition-specific regulatory events at high resolution.

## Abstract 2

A novel approach for identifying condition-specific regulatory modules in yeast reveals functionally distinct coregulated submodules.

## Background

Transcription regulation is a starting point for controlling a variety of biological processes, such as cell cycle progression and adaptive responses to environmental stimuli. Moreover, the regulation is realized by intricate regulatory gene networks that are mainly controlled by transcription factors. In order to appropriately process and respond to environmental changes, cells are likely to use distinct transcriptional regulatory networks by detecting specific features of complex environmental stimuli. Through altering the activities and targets of transcription factors depending on the cellular conditions, rewiring of transcriptional regulatory network occurs to adapt to various stimuli or initiate cellular programs [1]. Therefore, identifying the sophisticated architecture of transcriptional regulatory networks and further deciphering the mechanisms of transcriptional rewiring in response to various conditions would reveal the fundamental aspects of the mechanisms involved in the maintenance of life and adaptation to new environments.

Recently, many studies attempted to address these challenges by examining the transcriptional regulatory networks of *Saccharomyces cerevisiae *from various complementary perspectives. Luscombe *et al*. [2] analyzed the dynamics of transcriptional networks by using known transcriptional regulatory information and gene expression profiles of five specific environmental and developmental conditions. They reported that a majority of regulatory interactions among transcription factors and genes are highly condition specific, based on the observation that many of the transcription factors that regulated a large number of target genes in a certain condition did not maintain their regulation in other conditions. They also suggested that the topological properties of the networks differ considerably depending on the types of the conditions, classified as exogenous (for example, environmental stress) and endogenous (for example, cell cycle and sporulation). Harbison *et al*. [3] attempted to identify the dynamic nature of the transcriptional regulatory networks by conducting genome-wide binding assays for 203 transcription factors under various conditions. They found that, for most of the examined transcription factors, transcription factor binding to a regulatory sequence is highly dependent on the environmental condition of the cells. From these results, it is evident that dynamic alterations in the transcriptional network occur in response to changes in cellular conditions, although the actual mechanisms of rewiring and the detailed descriptions of the condition-specific regulatory networks remain to be explored.

To study all these aspects, we need to identify reliable condition-specific transcriptional regulatory modules. Identification of transcriptional regulatory modules, that is, gene groups sharing common regulatory mechanisms, is a major step toward deciphering the dynamic cellular regulation system more concretely. Many previous studies strived to identify the transcriptional regulatory modules and contributed to the detection of the links between gene expression and gene regulation by suggesting coexpressed gene modules controlled by their own regulators in various manners [4-6]. However, most studies assumed that a transcriptional regulatory network is static and usually defined coexpressed gene groups as the genes displaying similar expression profiles across multiple conditions; this viewpoint prevented the detection of the distinct features of condition-specific regulation. Although other studies employed condition-specific approaches [7-11], they did not clearly show the actual rewiring mechanisms of the condition-specific regulatory networks in response to external or internal signals. Moreover, most of them also presumed that the similarity in expression profiles among several genes implies their coregulation. In fact, stratification based on expression similarity obscures the transcriptional regulation program in many cases because an environmental or biological condition can activate multiple processes in parallel, and similar expression patterns can be elicited under multiple alternative regulatory mechanisms [12].

Here, we present an approach for identifying condition-specific regulatory modules in high resolution by integrating ChIP-chip, mRNA expression and known transcription factor binding motif data. By investigating diverse aspects of the identified modules and their regulators, we tried to dissect the dynamic properties of the condition-dependent regulatory networks and their rewiring mechanism. In this study, we adopted two distinctive strategies to reveal the dynamic transcriptional regulatory modules in detail. First, we identified the modules from each of the selected cellular conditions independently and then compared them in order to reveal the detailed and distinct features of the reorganized transcriptional regulatory network specified in each condition. Our results included various examples of regulatory events occurring in specific conditions that describe the reorganization of the transcriptional regulatory program depending on the change in stimuli conditions. Second, we identified multiple coregulated submodules from each of the coexpressed gene modules in high resolution. In order to obtain coregulated gene groups, we identified small coexpressed gene groups - initial module candidates (IMCs) - that comprised genes sharing common transcription factor binding evidence and employed them to identify the transcriptional regulatory modules. By considering the notion that the same expression can be activated through many independent transcriptional regulatory programs [12], this bottom-up approach allowed the detection of the local regulatory mechanisms that affect only a part of the entire coexpressed genes.

Through specialized strategies, we identified various condition-specific regulatory modules and their designated transcription factors in high resolution by using gene expression data obtained under different experimental conditions: heat shock, nitrogen depletion and mitotic cell cycle [13,14]. Excluding the treatment for cell cycle synchronization, the cell cycle condition can be regarded as a normal condition (YPD medium) with no limitation in cell growth and proliferation. The two stress conditions - heat shock and nitrogen depletion - were selected in order to investigate the distinct effects of environmental stress; the former elicits rapid and massive alterations in gene expression, while the latter is a prolonged nutrient-limiting condition. Although the regulatory modules from the three conditions shared some functional modules, most of them displayed unique functional properties specific to each condition due to the rewiring of the transcriptional regulatory network. In addition, many of the functional gene groups that exhibited distinct expression profiles in other conditions were coexpressed in a certain condition. We also investigated the distinguished condition-specific regulatory roles of the transcription factors by classifying them based on the degree and the manner in which they switch their target genes. Among the results obtained, many clear cases indicated that target switching by a transcription factor depending on the change in conditions entailed alteration of transcription factor combination and nucleosome occupancy on the promoters of the condition-specific target genes; these provided clues to the condition-specific rewiring mechanisms of the dynamic transcriptional regulation programs. We further examined the condition-specific features of the specialized regulatory networks by investigating the structure of the networks among the transcription factors and identifying the feed-forward loops (FFLs). We found that, compared to the cell cycle condition, the stress conditions required a wider propagation of regulatory signals and a substantially larger number of FFLs. Finally, through a case study on an expression pattern module (EPM), we determined a novel regulatory mechanism that can explain how several different transcription factors can induce similar expression profiles of their target genes by suggesting a regulatory hierarchy among the transcription factors.

## Results

### Identification of regulatory modules

**Figure 1:**
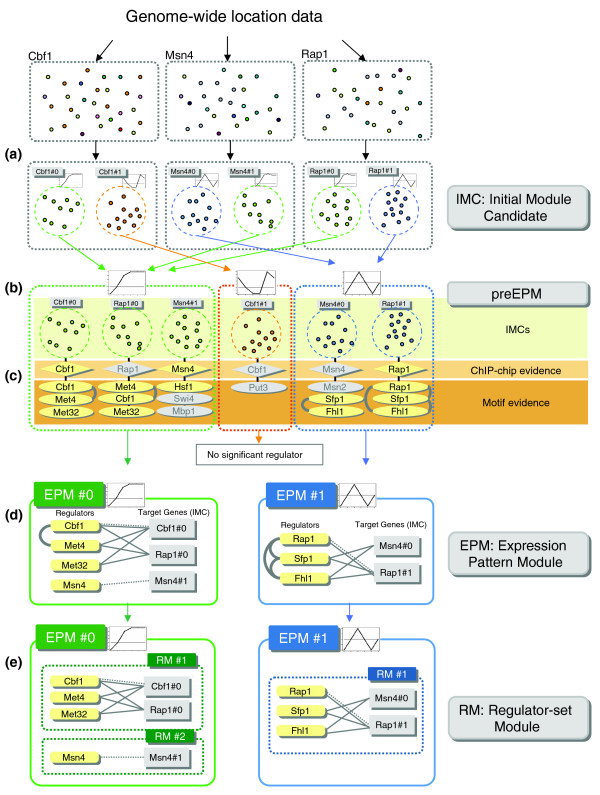
Overview of the method. **(a) **Splitting the genome-wide location (ChIP-chip) data into several coexpressed gene sets. Each of the derived target gene sets was called an IMC. Each IMC was named after the transcription factor of the ChIP-chip data followed by a serial number. Gray rectangles indicate the IMCs. Small dots indicate the genes bound to the transcription factor. **(b) **Generation of preEPMs. The IMCs with similar mean expression patterns were grouped for further analysis. **(c) **Detecting the regulators in each IMC. Initially, the over-represented motifs in each IMC were detected by the *t*-test. Next, biologically significant motif evidence and ChIP-chip evidence were selected using a test based on the hypergeometric distribution. Subsequently, in the case of motif evidence, recurrently confirmed motifs in each preEPM were selected. Yellow diamonds and ellipses indicate biologically significant regulators. Gray diamonds and ellipses represent the regulators that were not qualified by the test. Gray curved lines between the regulators indicate synergistic pairs. **(d) **Identification of an EPM. For each preEPM, the IMCs without a confirmed regulator were eliminated, and the retained IMCs and their corresponding regulators were arranged. Solid lines indicate motif evidence, and dotted lines indicate ChIP-chip evidence. **(e) **Identification of an RM. Regulators with highly overlapped target genes were united to identify an RM.

For the condition-specific analysis, we used three different gene expression data sets obtained from experiments performed under the heat shock, nitrogen depletion and cell cycle conditions [13,14]. For each condition, we identified small regulatory units (IMCs) by using the gene expression data and ChIP-chip data [3]. Each IMC comprised genes that are coexpressed under a specific experimental condition and share the same transcription factor binding evidence, as determined by ChIP-chip data (Figure 1a). Since the experimental conditions available in ChIP-chip data are not consistent with those in gene expression data, transcription factor binding evidence in any ChIP-chip data was respected at this step. Due to the augmented evidence by ChIP-chip data, IMCs were more informative than simple gene sets that are grouped by expression similarity alone. Supporting this notion, it has been reported that splitting the coexpressed genes into smaller subsets based on prior knowledge can enhance the identification of new regulatory elements [6]. The similarly expressed IMCs were grouped together and used as the precursors of the expression pattern modules (preEPMs; Figure 1b).

In order to detect the plausible regulators of each preEPM, transcription factor binding information from ChIP-chip data [3], known motif data from SCPD [15], TRANSFAC [16] and putative motifs from Harbison *et al*. [3] were exploited to detect the regulators of each IMC (Figure 1c). First, we examined whether the shared transcription factor of an IMC is a reliable regulator for the IMC. Just the fact that the transcription factor was bound to the genes might not necessarily imply regulation because the gene regulation activity of the transcription factor depends on the condition or cofactors [17,18]. Hence, we performed a hypergeometric test to investigate whether the binding of a transcription factor is associated with gene expression. The hypergeometric test assessed the enrichment of the transcription factor-bound genes among the genes showing expression profiles similar to the mean expression pattern of the IMC in all yeast genes. Throughout the test, we filtered out the transcription factors that were not associated with gene expression. In addition, we employed the transcription factor binding motif data to identify additional regulatory elements. For each IMC, we examined whether a motif was over-represented in the IMC by using the *t*-test (see Materials and methods). Similar to the relationship between transcription factor binding and gene expression, the presence of a binding site does not guarantee recruitment of transcription factor nor gene regulation. Therefore, we filtered out the motifs that were not significantly associated with expression pattern in the same manner described above. To remove false positives, a motif was considered as the reliable evidence of transcription factor regulation only when it was qualified by the tests for at least two IMCs in a preEPM. As a result, more than half of the initial candidate regulatory evidence was filtered out (Additional data file 1).

Finally, after discarding the IMCs that did not involve any confirmed regulators, EPMs were identified by gathering the retained IMCs in preEPMs. An EPM is defined as a group of genes that share similar expression profiles under a specific condition and their regulators that were confirmed by the statistical examination of the association with the common expression pattern of the EPM. To each regulator identified from the IMCs in the EPM, we allocated the target genes by gathering the genes of the IMCs that had provided confirmatory evidence of the transcription factor (Figure 1d). To further characterize the distinct coregulated gene subgroups in an EPM, we analyzed the combination of regulators in the EPM by examining the overlap level (*OL*) of their target genes and subsequently defined the regulator-set modules (RMs). A regulator set is a set of transcription factors that share many target genes in an EPM, and the union of their target genes is considered as the member genes of an RM (Figure 1e).

In order to characterize the genes in the EPMs/RMs and the target genes of transcription factors, we conducted a functional category enrichment analysis. Briefly, each gene set was verified for significant enrichment in any of the Gene Ontology (GO) categories [19] (shown in Additional data files 2 and 11). Interestingly, most of our regulatory modules (EPMs and RMs) and the target genes of the transcription factors appeared to have condition-specific functional roles. Moreover, each RM or a combination of multiple RMs appeared to represent a functional part of an EPM. We will discuss the functional enrichment of RMs in detail later in the paper.

### Overall results of module analysis

The module analysis described above revealed that several EPMs and RMs differed in the average module size (number of member genes) or in the average number of identified transcription factors depending on the conditions (Table 1). The average number of member genes per EPM was greater in stress conditions, namely, heat shock and nitrogen depletion, whereas that in the cell cycle condition was relatively small. This indicates that a large number of genes are coexpressed in response to stress stimuli, whereas a relatively small number of genes are similarly expressed in response to intrinsic signals for cell cycle progression. A similar tendency was also observed with regard to the number of target genes per transcription factor; on average, 97 genes in the heat shock condition, 78 genes in the nitrogen depletion condition, and 32 genes in the cell cycle condition were found to be regulated by a transcription factor. This tendency is in agreement with the result of a previous report on the properties of condition-specific transcriptional regulatory networks [2], which suggested that a relatively smaller number of target genes are linked to a transcription factor in the cell cycle condition than to regulatory networks in stress conditions.

**Table 1:** Number of IMCs, EPMs, RMs and their average number of member genes and regulators

				Average no. of genes/transcription factors			
					
Condition	No. of survived IMCs	No. of EPMs	No. of RMs (average number of RMs per EPM)	IMC	EPM	RM	No. of confirmed transcription factors (average number of targets per transcription factor)
Heat shock	249	14	88 (6.3)	9.8/3.2	102.7/11.6	58.9/3.0	67 (96.6)
Nitrogen depletion	340	24	166 (6.9)	9.1/3.3	77.5/13.1	40.3/3.1	96 (78.0)
Cell cycle	77	9	35 (3.9)	7.5/2.9	36.3/7.3	26.6/3.0	43 (31.5)

For each condition, we calculated the number of retained IMCs that have at least one confirmed transcription factor. And then, total numbers of EPMs and RMs were counted. We also calculated the average number of genes and transcription factors per IMC, EPM and RM.

Interestingly, the average number of transcription factors per RM was quite similar across all the three conditions. We have previously noted that an RM is a coregulated functional unit for the coexpressed genes. The number of regulators in each functional unit was approximately three in all the conditions, implying that, on average, three transcription factors participate in the gene regulation of a specific functional unit, regardless of the condition. However, the average number of RMs per EPM displayed a clear difference; the EPMs in the stress conditions tended to have more RMs than those in the cell cycle condition. On average, seven RMs in the nitrogen depletion condition, six RMs in the heat shock condition, and four RMs in the cell cycle condition were included in an EPM. This implies that EPMs in the stress conditions include more diverse functional units than those in the cell cycle condition. Accordingly, the average number of transcription factors per EPM in the two stress conditions was significantly larger than that in the cell cycle condition. This might be the result of a more intensive need for cooperation among various functional gene groups in order to respond to stress stimuli. We will describe the detailed examples of this cooperation later in the paper.

### Condition-specific organization of regulatory modules

Our results showed that the transcriptional regulatory modules were largely reorganized depending on the cellular conditions. As expected, the difference between the normal condition (for example, cell cycle) and the environmental stress conditions (for example, heat shock and nitrogen depletion) was conspicuous. In the cell cycle condition, periodic changes in the gene expression levels along cell cycle progression were reflected in the organization of relatively small EPMs. On the other hand, in the environmental stress conditions, an evident symmetry of expression profiles appeared between stress-induced EPMs and stress-repressed EPMs. Moreover, clear differences in the reorganizing patterns between the EPMs under the heat shock condition and those under the nitrogen depletion condition were observed, although they shared some common features of general response to stress. Regarding the average expression profiles of the EPMs, the heat stress-induced or the heat stress-repressed EPMs displayed transient but significant changes in their transcription levels, whereas the genes in the nitrogen depletion-induced EPMs showed induction or repression over an extended period. Besides, there were many unique features of the organized condition-specific modules depending on the type of the stimulus.

**Figure 2:**
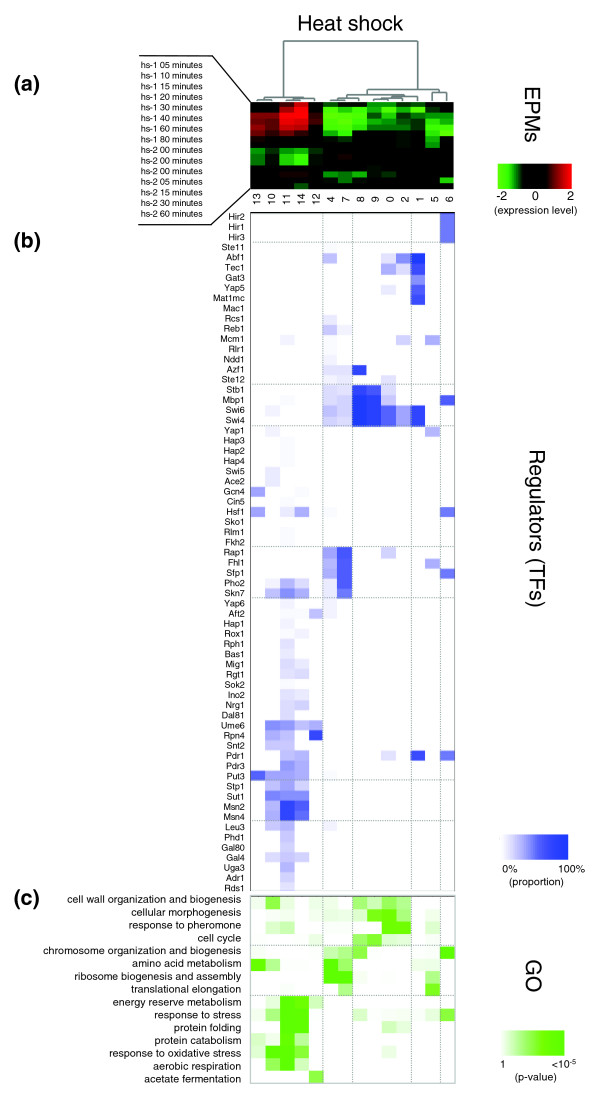
EPMs identified in the heat shock condition. **(a) **The result by hierarchical clustering of the average expression patterns of EPMs in the heat shock condition. The numbers indicate the EPM indices. **(b) **Regulator matrix whose entries represent the percentage of genes controlled by each transcription factor in the EPM. The names of transcription factors are shown on the left side. **(c) **Gene annotation enrichment matrix whose entries represent the enrichment levels of each EPM in the GO 'biological process' categories shown on the left side. For efficient explanation and visualization, only selected GO categories are shown. EPMs identified in the nitrogen depletion and the cell cycle conditions are shown in Additional data file 2.

In the heat shock condition, two large clusters of EPMs exhibited reciprocal expression profiles: one comprised upregulated EPMs and the other comprised downregulated EPMs. Further, the EPMs in each of the clusters could be distinguished based on their distinct peak points (Figure 2a and Additional data file 3). In the upregulated EPMs (heat shock EPMs 10-14), various stress-response genes (for example, protein folding and degradation, oxidative stress response, and energy reserve metabolism-related genes) were included together with the genes for energy derivation (for example, aerobic respiration and fermentation genes) (Figure 2c). These results are consistent with several known facts: first, the concurrent induction of protein folding/degradation genes and aerobic respiration genes supports the notion that chaperones and proteolytic proteins require large amounts of ATP [20] that can be supplied by aerobic respiration and fermentation; second, it has also been reported that the levels of major energy reserves (for example, glycogen and trehalose) increase in response to the heat shock condition [21]; and third, heat stress produces oxidative stress that involves mitochondrial respiratory electron carriers [22]. The downregulated EPMs were largely organized into two groups: one comprised the genes related to cell cycle, mating and cell wall (heat shock EPMs 0, 2, 8 and 9), and the other comprised the genes involved in ribosome biogenesis and protein biosynthesis (heat shock EPMs 4 and 7). Their expression profiles exhibited the process of adaptation to the heat shock condition, that is, initially they are highly repressed, but after significant time has elapsed, their expression levels start increasing [23] (more detailed descriptions are provided in Additional data file 3).

In the nitrogen depletion condition, a wide range of functional gene groups displayed various expression profiles, and a number of EPMs were organized; these demonstrated interesting condition-specific features. There were four EPMs related to amino acid metabolism, and they could be divided into two groups - amino acid biosynthetic EPMs (nitrogen depletion EPMs 0, 1 and 2) and amino acid catabolic EPMs (nitrogen depletion EPM 25) (see Additional data file 2). In the microarray experiments for nitrogen depletion, a medium containing a small amount of a nitrogen source but neither amino acids nor nucleotides was used [14]. Until the depletion of the nitrogen source, the cells behaved as if they were under amino acid starvation. Genes in the amino acid biosynthetic EPMs (EPM 0, 1 and 2) were induced as long as the nitrogen source was available but displayed an abrupt decline after the depletion of the nitrogen source. On the other hand, EPM 25, which included amino acid catabolic genes and the genes responsible for the nitrogen starvation response, displayed a reverse pattern; they were quiescent while the nitrogen source was available but started to be induced after the depletion of the nitrogen source. It appears that amino acid catabolic EPMs contribute to increasing the turnover rate of amino acids in response to nitrogen starvation. Moreover, the expression profiles of ribosome biogenesis EPMs (nitrogen depletion EPMs 11, 12 and 19) fluctuated depending on the availability of amino acids; their expression levels were upregulated when amino acids were available (Additional data file 3).

In the cell cycle condition, several phase-specific cell cycle EPMs (cell cycle EPMs 1, 5 and 6) were identified, and their regulators were largely in agreement with those mentioned in the previous reports (Additional data file 4). In addition, we detected ribosome biogenesis EPMs (cell cycle EPMs 0 and 4), an energy generation-related EPM (cell cycle EPM 7) and an amino acid metabolism-related EPM (cell cycle EPM 8) (Additional data file 2). The expression levels of all these EPMs commonly peaked at the G1 phase and the G2/M transition, although their overall expression profiles were distinguishable (Additional data file 3). This result indicates that the roles of these EPMs are particularly important during the G1 phase and the G2/M transition; this finding is supported by the previous studies wherein genes controlling ribosome biogenesis and protein translation have been identified as the critical regulators of cell growth and cell cycle in yeast [24-26] and by the studies demonstrating that the critical cell size requirement is fulfilled in the G1/S and G2/M transitions [27,28]. Unexpectedly, a stress response-related EPM was also detected (cell cycle EPM 3). The presence of this EPM appears to reflect the experimental condition adopted by Cho *et al*. [13]; they employed the heat shock treatment for cell cycle synchronization before their measurements. The average expression of this EPM displayed a peak at the beginning of the experiments but abruptly decreased later, implying that the influence of the heat shock treatment vanishes with time. The phase-specific cell cycle EPMs are discussed in more detail in Additional data file 4.

### Comparison of modules across conditions

**Figure 3:**
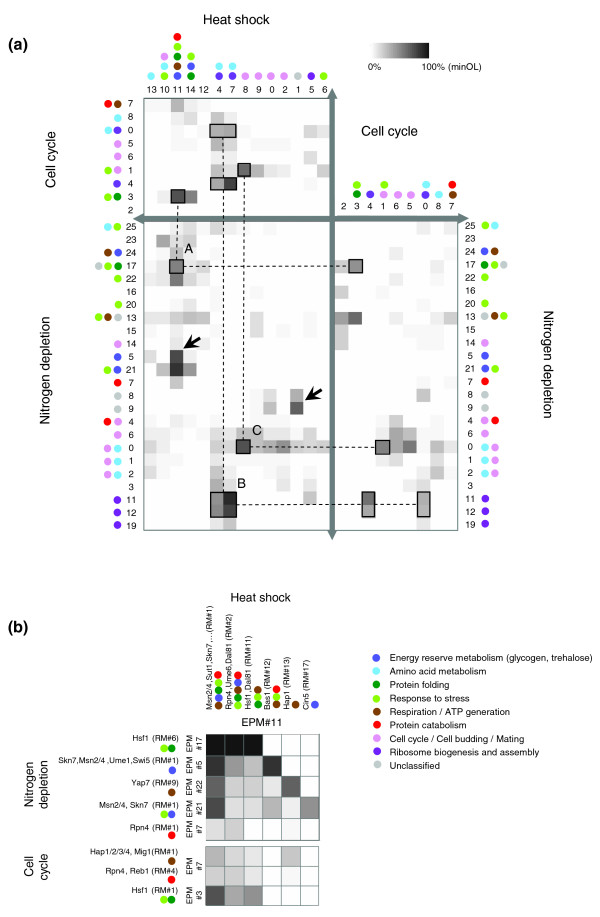
Overlap matrices of regulatory modules. **(a) **Overlap matrices between EPMs in all the three conditions. The *OL*s were calculated as the proportion of the intersection genes in the smaller EPM (*minOL*). The enriched GO categories of each EPM are also shown as several colored dots. Black-lined boxes represent the EPMs that are significantly overlapped across all the three conditions. 'A' indicates the overlapped stress-related EPMs represented by the three boxes linked by dashed lines. They have the common regulators Msn2/4 and Hsf1. Identically, the EPMs indicated as 'B' have the common regulators Rap1, Sfp1 and Fhl1. The EPMs indicated as 'C' have Mbp1, Swi4, Swi6 and Stb1 as their common regulators. Black arrows indicate EPMs that are highly overlapped between the heat shock and nitrogen depletion conditions. **(b) **Overlap matrices between RMs (*minOL*). Several RMs, which were included in the distinct EPMs in the nitrogen depletion and cell cycle conditions, are significantly overlapped with the RMs in heat shock EPM 11.

To further investigate the differences and similarities among EPMs from the three tested conditions, the member genes in the EPMs were compared across conditions. Although the shapes of the reorganized EPMs differed among the three conditions, the following three highly overlapped EPM clusters were detected in all the conditions (Figure 3a): EPMs of stress response (heat shock EPM 11, nitrogen depletion EPM 17 and cell cycle EPM 3), EPMs of ribosome biogenesis (heat shock EPMs 4 and 7, nitrogen depletion EPMs 11 and 12 and cell cycle EPMs 0 and 4) and EPMs of the cell cycle (heat shock EPM 8, nitrogen depletion EPM 0 and cell cycle EPM 1). These modules shared some common transcription factors, and we conjecture that the regulation of these modules would be conserved in various physiological conditions.

Some functional EPMs were detected in only the two environmental stress conditions. For instance, genes for energy reservation (for example, generating glycogen and trehalose) were included only in the EPMs in the heat shock (EPMs 11 and 14) and nitrogen depletion (EPMs 5 and 21) conditions. All these EPMs were commonly regulated by Msn2/4 and Skn7 (Figure 2b), which are well-known stress-response regulators [29-31]. Furthermore, both heat shock EPM 1 and nitrogen depletion EPM 9 were enriched with 'biological process unknown' genes and contained several common regulators (Yap5, Gat3, Swi4/6, Tec1, Mat1-Mc and Abf1) and were found to overlap significantly; however, these EPMs did not overlap with any cell cycle EPMs. These EPMs may be related to some unknown functions that are commonly involved in heat shock and nitrogen depletion response.

By analyzing the overlap of several RMs, we found that various gene groups involved in several distinct EPMs in other conditions converged to form a single EPM in a specific condition. For example, several stress-response gene groups and energy generation-related gene groups, which showed diverse expression patterns and were organized into several independent EPMs in the nitrogen depletion or cell cycle condition, were coexpressed under the heat shock condition and formed an integrated EPM (Figure 3b). Among the nitrogen depletion EPMs, the crucial parts of the EPMs for energy reserve metabolism (nitrogen depletion EPMs 5 and 21), protein folding and degradation (nitrogen depletion EPMs 17 and 7, respectively) and respiration (nitrogen depletion EPM 22) converged into a single heat-shock EPM (heat shock EPM 11). Similarly, many genes for protein folding, protein degradation and respiration in the EPMs in the cell cycle condition (cell cycle EPMs 3 and 7) were found to be included together in the heat shock EPM 11. Nitrogen depletion EPM 0 also exhibited coexpression of multiple functional gene groups that were included in several different EPMs in other conditions (Additional data file 5).

It is also notable that the list of target genes of Rpn4, a transcription factor for heat shock EPM 11 and known as a transcriptional activator of genes encoding proteasomal subunits [32], was expanded to include the protein folding-related genes, while Rpn4 retained its regulatory role on the genes related to protein degradation in the heat shock condition. Similarly, in addition to the previously characterized stress response-related target genes, energy generation-related genes were included in the target genes of Msn2/4 and Skn7, which are the major regulators of heat shock EPM 11. From these examples, we conjecture that some coordinated regulation might operate for a more efficient response to the heat stress. In the heat shock condition, protein folding and protein degradation might be coherently regulated because the failure of the protein folding process often entails degradation of the misfolded proteins. In addition, the coupling of energy generation and protein folding (and degradation) would enhance the response to heat stress because the latter process requires considerable energy, as mentioned before. Several previous studies support our inferences. It has been reported that molecular chaperones assist in not only protein refolding but also protein degradation by interacting with protein degradation systems; when chaperones fail in their functions of protein folding, assembly or translocation, they facilitate degradation of the mishandled proteins [33,34]. Our results and experimental evidence suggest that cells can respond to a stimulus more rapidly and efficiently by co-inducing the energy-consuming stress response genes and the energy-providing genes.

### Specified regulatory roles of transcription factors depending on conditions

A total of 109 transcription factors were confirmed as regulators of all the EPMs and RMs identified from the three conditions; 67, 96 and 43 transcription factors were confirmed in the identified modules from the heat shock, the nitrogen depletion and the cell cycle conditions, respectively. There were 33 transcription factors common in all the three conditions (Additional data file 6). In order to investigate the overall regulatory roles of the transcription factors in each condition, we identified all the target genes of each transcription factor and analyzed their enriched functional GO categories (Additional data file 7). Of the 33 common transcription factors, 20 appeared to retain at least one of their regulatory roles in all the conditions. Among the 109 total transcription factors, 69 exhibited their known regulatory roles in at least one condition. Considering that we conducted the analysis for only three conditions and that many transcription factors exhibit their roles only under specific conditions, we believe that the number of transcription factors that agree with their experimentally proven roles would increase if more diverse conditions were analyzed.

**Figure 4:**
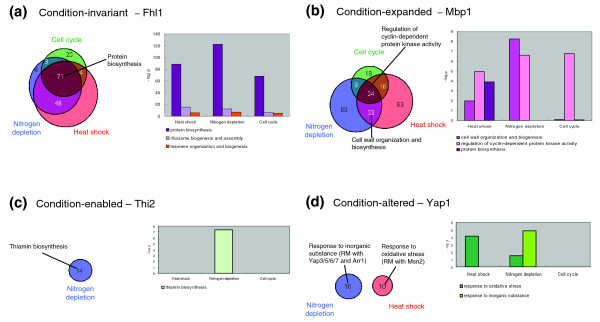
Condition-specific types of transcription factor. The transcription factors were classified into four types based on the alteration in the target genes: **(a) **condition-invariant, **(b) **condition-expanded, **(c) **condition-enabled and **(d) **condition-altered. The venn diagrams show the overlapped target genes of the representative transcription factors among the three conditions. In the bar graph, the y-axis represents the significance of the *p *value for the enriched functional categories of the target genes in each condition.

Similar to the classification of the transcription factor binding patterns into four types based on the change in conditions by Harbison *et al*. [3], we attempted to classify the transcription factors based on the alterations in target genes as follows: 'condition-invariant', in which the transcription factor target genes are highly conserved across the conditions; 'condition-expanded', in which the list of target genes in one condition is further expanded to include more target genes in other condition; 'condition-enabled', in which the transcription factor regulates some target genes in one specific condition but not in other; and 'condition-altered', in which different sets of target genes are regulated by the same transcription factor in different conditions. We found that most transcription factors could be classified into one or more of these groups, and the overall *OL *between the target genes of transcription factors in different conditions indirectly reflected their types (Figure 4 and Additional data file 7).

The transcription factors Rap1, Fhl1, and Sfp1, which are the well-established ribosome biogenesis-related regulators [35,36], were classified into the 'condition-invariant' group; they retained most of their regulatory roles (protein biosynthesis, ribosome biogenesis and assembly, and telomere maintenance) in all the three conditions (Figure 4a). Mbp1, a renowned cell cycle regulator, could be categorized as a 'condition-expanded' transcription factor; it expanded its targets to include the cell wall biosynthesis-related genes under the two environmental stress conditions (Figure 4b). Many other cell cycle-related transcription factors, including Swi4/6 and Stb1, showed a similar expansion of targets to regulate the cell wall biosynthesis-related genes under the two stress conditions. Rpn4 was another good example of 'condition-expanded' transcription factors. As mentioned earlier, the target gene list of Rpn4 was expanded to include the protein folding-related genes in response to heat shock, while Rpn4 retained its own regulatory role of protein degradation. Many transcription factors could be categorized as 'condition-enabled' transcription factors; Thi2, a transcriptional activator of thiamin biosynthetic genes [37], appeared to exhibit its known role only under the nitrogen depletion condition (Figure 4c). Zap1, a zinc-responsive transcription factor that activates the zinc transporter genes [38], was confirmed as a regulator of zinc transportation-related genes only under the cell cycle condition. Snt2, a previously uncharacterized DNA-binding protein, was predicted to control the genes related to ATP synthesis and energy reserve metabolism only under the heat shock condition. There were some 'condition-altered' transcription factors whose regulatory roles changed completely depending on the condition. Interestingly, the change in regulatory roles often necessitated the alteration of partner transcription factors. For example, Yap1, a regulator of genes related to the response to oxidative stress and inorganic substances [39,40], was predicted to regulate the genes related to oxidative stress response along with Msn2 under the heat shock condition (RM 9 of heat shock EPM 10), whereas it appeared to control the genes involved in response to inorganic substances along with Yap3/5/6/7 and Arr1 (Yap8) under the nitrogen depletion condition (RM 18 of nitrogen depletion EPM 13); all these roles detected in this study were largely in agreement with their known functions (Figure 4d) [41,42]. Similarly, Uga3 appeared to regulate the genes related to protein biosynthesis and ribosome biogenesis along with Sfp1, Fhl1, Skn7 and Sut1 under the cell cycle condition (RM 3 of cell cycle EPM 0), while it was predicted to regulate the branched chain amino acid biosynthetic genes along with Leu3 under the nitrogen depletion condition (RM 3 of nitrogen depletion EPM 2). Moreover, Uga3 along with Msn2/4 and many other transcription factors exerted its regulatory role on the genes related to ATP generation and energy reserve metabolism under the heat shock condition (RMs 2 and 3 of heat shock EPM 11).

In order to validate the prediction of 'condition-altered' regulators, we examined whether the accessibility of the predicted target promoters undergoes changes depending on the conditions by using an independent data set that represents the genome-wide occupancy of nucleosomes and transcriptional machinery under the heat shock condition [18]. Compared to the normal condition, the promoter regions of Uga3's and Yap1's target genes predicted by our analysis of heat shock response exhibited lower occupancy of histones and higher occupancy of most transcriptional machinery components (for example, RNA polymerase II) under the heat shock condition; on the contrary, the occupancy on the promoters of Uga3's target genes predicted by our analysis of cell cycle condition exhibited the opposite pattern (Additional data file 8). This result indicates that the predicted target genes of Uga3 and Yap1 become accessible to the transcriptional machinery to different extents depending on the conditions, supporting the predictions by our analysis.

### Condition-specific regulatory networks among transcription factors

**Figure 5:**
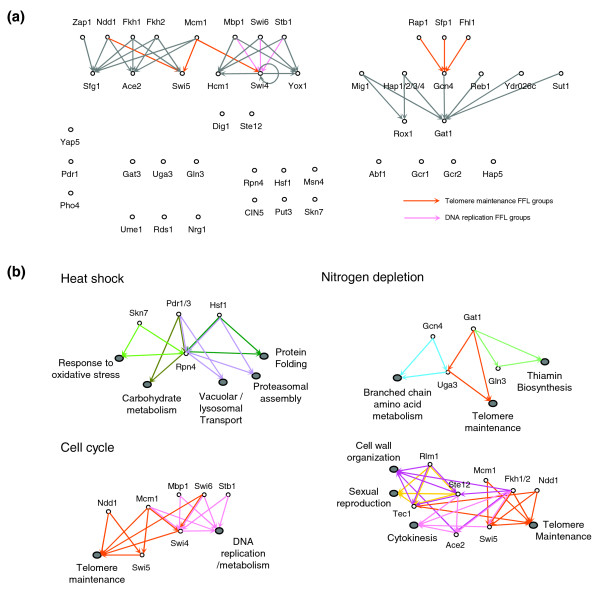
Transcriptional regulation among transcription factors. **(a) **Regulatory network among the transcription factors in the cell cycle condition. Each arrow represents transcriptional regulation. Two transcription factors linked by each colored arrow form an FFL group with the genes that are enriched in some specific functional category. The colors of the arrows imply the enriched functional categories. The networks in the two stress conditions are shown in Additional data file 9. **(b) **Condition-specific FFL groups. Each gray ellipse represents the overlapped target genes of the two transcription factors in an FFL group. Only selected FFL groups are shown (see Additional data file 10 for the complete list of the enriched functional categories of the FFL groups).

To further investigate the specific properties of the transcriptional regulatory networks in each condition, we analyzed the relationship among the transcription factors by identifying the transcription factors that regulate the expression levels of other transcription factors. We could detect some condition-dependent topological and functional features in the networks. The complexity of the regulatory networks among the transcription factors (for example, the number of target transcription factors of a transcription factor) in the stress conditions was higher than that in the cell cycle condition; on average, 4.5, 3.7 and 1 transcription factor-encoding genes were found to be regulated by a transcription factor under the heat shock, the nitrogen depletion and the cell cycle conditions, respectively (see Figure 5a for the cell cycle condition and Additional data file 9 for the two stress conditions). It appears that the number of target transcription factors and the overall topology of networks are related to the different responses to different types of stimuli. Under the heat shock condition, fast and global signal propagation for adapting to the new environment would be required; therefore, a highly dispersed transcription factor hierarchy would be efficient for such a response. On the other hand, such widespread regulatory signal propagation would not be necessary under the cell cycle condition because cell cycle progression requires only periodic control of some specific functional genes.

We found many FFLs in the condition-specific transcriptional regulatory networks. An FFL is composed of two input transcription factors, one of which regulates the other and both jointly regulate a specific target gene. It responds only to a persistent stimulus and is often detected under the conditions wherein an external signal causes many systems to respond rapidly [43]. Moreover, the combination of two input transcription factors may contribute to the gain of specificity for certain functional target genes. We defined an 'FFL group' when two transcription factors shared some functionally coherent target genes enriched in at least one of the GO categories. The numbers of the identified FFL groups were consistent with the properties of the FFLs, and considerably more FFL groups were identified in the two stress conditions than the cell cycle condition. In total, we identified 91 FFL groups in the heat shock condition, 132 in the nitrogen depletion condition and 9 in the cell cycle condition, reflecting the characteristics of the cellular conditions; nitrogen depletion generates prolonged nitrogen starvation signals, while the signals in cell cycle progression are relatively transient. Moreover, the gene expression data of the heat shock condition indicated that a number of genes undergo an abrupt change in expression level, implying that extremely rapid and widespread regulatory signal propagation occurs during this condition.

The target genes of the FFL groups reflected specifically activated (or repressed) functions that were related to the conditions in which the FFL groups were identified. Recently, it has been reported that Hsf1 and Pdr1/3 participate in the regulation of Rpn4 that in turn activates the expression of proteasomal genes, particularly under the heat shock condition [44]. Our results from the heat shock condition were in good agreement with this experimental evidence; Hsf1 and Rpn4 were predicted to constitute an FFL group whose target genes are involved in proteosomal assembly and protein folding, and Pdr1 and Pdr3 appeared to form FFL groups along with Rpn4 to regulate the genes related to vacuolar and lysosomal transport, respectively (Figure 5b, heat shock). In the nitrogen depletion condition, we observed many FFL groups that specified their targets by altering the partner transcription factors; Gcn4 and Uga3 were predicted to form an FFL group that regulates the branched chain amino acid metabolic genes, while Gat1 and Uga3 constituted another FFL group controlling the genes involved in telomere maintenance. Gat1 also appeared to participate in an FFL group along with Gln3 to regulate the thiamin biosynthetic genes (Figure 5b, nitrogen depletion). Besides, we detected many cases in which the FFLs reflected the known roles of the participating transcription factors. For example, Ste12, Tec1 and Rlm1, all of which are reported to participate in the cell wall integrity signaling pathway [45-47], appeared to constitute the FFL groups regulating the cell wall biosynthetic genes. In the cell cycle condition, all the identified FFL groups were involved in cell cycle control or regulation of telomere maintenance; Mbp1, Swi6, Stb1 and Mcm1 appeared to regulate the genes involved in the cell cycle along with Swi4 as a second transcription factor, while Ndd1 and Mcm1 were found to regulate the expression of Swi5, leading to a coordinated regulation of the genes related to telomere maintenance (Figure 5b, cell cycle).

### High-resolution regulatory modules - a case study with nitrogen depletion EPM 2

**Figure 6:**
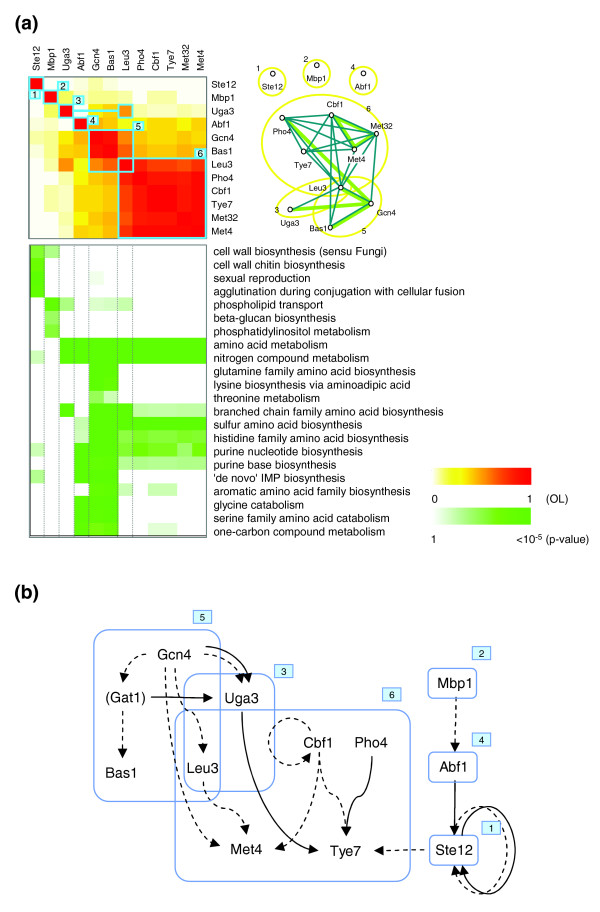
Case study: RMs in nitrogen depletion EPM 2. **(a) **EPM 2 of the nitrogen depletion condition is represented by two matrices. The upper matrix represents the overlap between the target genes of the regulators in the EPM, and blue boxes represent the RMs. The lower matrix represents the enrichment of the target genes per regulator in the GO 'biological process' categories. For simplicity, only selected categories are shown. A more detailed explanation is presented in Additional data file 4, and the complete matrices with all significantly enriched categories (*p *value < 0.01) are presented in Additional data file 11. In the graph, yellow ellipses indicate the RMs. Blue lines represent a significant overlap (*OL *≥ 0.5) between the target genes of the two transcription factors at nodes, and green lines represent synergistic links identified by the hypergeometric test for transcription factor pairs (see Materials and methods). The detailed information about other EPMs and RMs is available in Additional data file 11. **(b) **Potential regulatory scheme among the regulators in the nitrogen depletion EPM 2. Solid arrows indicate the regulator-target gene relationship from the prediction under the nitrogen depletion condition, and dotted arrows indicate the transcription factor binding information from ChIP-chip data. Gat1 is included for completion of the hierarchy, although it is not among the predicted regulators of the nitrogen depletion EPM 2.

One of our interesting results is that each coexpressed gene module (EPM) appears to include several separate submodules (RMs) that are regulated by different sets of transcription factors but yet share the same expression pattern. Figure 6a shows how well RMs are defined as functional parts in nitrogen depletion EPM 2. In this EPM, we detected six RMs that were largely involved in cell wall biosynthesis (RMs 1 and 2) and amino acid metabolism (RMs 3-6). Each of the RMs further included distinguishable detailed functional parts. Notably, each or various combinations of the RMs 3-6 were specifically enriched with the functional categories related to amino acid metabolism (for example, branched chain family amino acid biosynthesis, sulfur amino acid biosynthesis). In addition, most of the predicted regulators of the RMs in this EPM have relevant experimental evidence (more detailed explanations and literature evidence are described in Additional data file 4).

An interesting issue raised by our results is how several different transcription factors in an EPM can drive a similar expression pattern of their target genes. It is presumable that there are 'master regulators' that, by hierarchically controlling distinct sets of transcription factors, allow coordinated transcriptional responses of a large set of genes that are not directly coregulated by the same transcription factors. By combining our transcription factor-target gene prediction and ChIP-chip data [3], we could detect a potential hierarchical structure of the regulators within nitrogen depletion EPM 2 (Figure 6b). Except Met32, 11 out of the 12 transcription factors participated in this potential regulatory scheme, and Gcn4, Cbf1, Pho4 and Mbp1 were located at the top of the hierarchy, suggesting that these four transcription factors are the putative 'master regulators' for this EPM. Across all the EPMs, on average 56% of the regulators in an EPM (60%, 59% and 41% under the heat shock, the nitrogen depletion and the cell cycle conditions, respectively) appeared to participate in the potential regulatory network among the regulators in the EPM (Additional data file 12).

### Comparison with other methods

To evaluate our method, we compared our modules with several other predicted gene regulatory modules, including GRAM [5] and COGRIM [48], which were predicted using similar types of data but different approaches. The GRAM algorithm identified 106 modules by combining the ChIP-chip and gene expression data, and COGRIM predicted 2,298 gene-transcription factor interactions by integrating ChIP-chip, transcription factor binding motif and gene expression data. First, for comparison of the coverage of the results from different approaches, we compared the numbersof distinct genes andtranscription factors that are included in the predicted modules. As shown in Table 2, the EPMs and RMs included much more genes (2,099) and transcription factors (109) than GRAM and COGRIM, indicating that our method provides more information about transcriptional regulatory events than other methods, despite that we used gene expression data from only 42 experiments while other methods employed approximately 500 expression experiments.

**Table 2:** Overall comparison with other methods

				GO functional enrichment level			
				
				Overall modules			
					
Method	No. of modules	No. of distinct genes	No. of distinct TFs	BP	MF	CC	Target genes of common 38 TFs
EPM	47	2,099	109	14.36 (8.63)	12.94 (8.53)	15.32 (7.80)	17.86 (21.49)
RM	289	2,099	109	7.56 (3.43)	6.51 (3.24)	7.23 (4.95)	
GRAM	106	655	69	8.54 (1.05)	7.77 (4.64)	8.73 (2.18)	12.28 (19.47)
COGRIM (B-/C+)	39	841	39	8.67 (2.97)	6.81 (4.24)	7.43 (2.72)	8.81 (13.43)
COGRIM (B+/C+)	39	936	39	5.3 (0.68)	4.55 (0.18)	4.86 (0.68)	5.19 (4.81)

The number of modules obtained from each method and the number of distinct genes and transcription factors (TFs) that constitute modules identified by each method are shown. In addition, GO functional enrichment levels are shown. The GO (BP, biological process; MF, molecular function; CC, cellular process) enrichment *p *values were transformed to negative log values and averaged over all modules. We also performed a functional enrichment test for the target genes of 38 transcriptional factors that were commonly predicted in all the three methods. The standard deviations are shown in parentheses.

We then compared the average GO enrichment level of the predicted regulatory modules by calculating a negative-log transformed *p *value. As shown in Table 2, the average enrichment levels of EPMs were higher than those of the other predicted modules. This implies that EPMs comprise more functionally coherent genes according to the GO annotation. For a more objective comparison, we also analyzed the enrichment levels of the target genes of 38 transcription factors that are commonly included in all the predictions. The result showed that our method has the highest enrichment level (Table 2).

In order to compare how well the modules can explain the detailed parts of a function, we further analyzed the enrichment levels of the modules related to ribosome biogenesis and assembly. We found that our EPMs and RMs showed higher enrichment levels than other methods in 11 out of 14 GO categories related to the function (Additional data file 13). Furthermore, we compared nitrogen depletion EPM 12 with several modules from GRAM, all of which are significantly related to ribosome biogenesis. In GRAM, 17 transcription factors were linked to 21 ribosome biogenesis-related gene modules. However, many of their modules were found to highly overlap with each other; therefore, they could not be distinguished in terms of function. In contrast, nitrogen depletion EPM 12 contained ten transcription factors (eight transcription factors overlapped with GRAM) and was divided into five RMs that clearly described the detailed cellular process of ribosomal biogenesis, from rRNA synthesis to ribosome assembly (Additional data file 13).

In general, a high coverage can be obtained by simply adopting lenient criteria for identifying modules. However, the more lenient criteria are applied, the more false positives are incorporated in the analysis result, leading to lower specificity that would yield poor functional enrichment. Remarkably, comparison with other methods suggests that our results are not only functionally more relevant but also cover a wider range of the whole yeast genome. We believe that higher enrichment level and coverage of our results could be accomplished by the bottom-up approach that initially identified small coexpressed and coregulated gene groups by efficiently combining the ChIP-chip and expression data. In contrast to other methods that used combined expression data over a number of experimental conditions, we used separate experimental units of expression data for identifying condition-specific regulatory modules; this enabled us to obtain the target genes of transcription factors that are specific to each condition and functionally more coherent. Through this bottom-up approach, it was possible to identify many distinct submodules included in a coexpressed gene group and describe the detailed multiple alternative regulatory mechanisms involved in the coexpression.

## Discussion

In this study, we elucidated the coexpressed gene modules (EPMs) and their intrinsic functional submodules (RMs) along with their regulators through a sensitive and robust method using ChIP-chip, known motif and microarray data. We presented several condition-specific EPMs and described the differences between the EPMs reorganized depending on the change in conditions. Substantial parts of the results are consistent with the previously reported condition-specific regulatory events and well-characterized regulatory mechanisms, suggesting that our strategy and results are reliable.

Our results revealed several important features about the dynamic nature of the condition-specific reorganization of the transcriptional regulatory network. Certain functions appeared predominantly in the organized gene regulatory modules in each condition. For example, various stress response- and energy-related genes constituted a large proportion of the modules detected in the heat shock condition, while genes for amino acid and nucleotide metabolism were organized into many modules in the nitrogen depletion condition. In the cell cycle condition, many gene regulatory modules responsible for the internal periodic phase-specific cell cycle signals were identified. Besides, in the two stress conditions, the change in condition triggered the coexpression of many distinct functional groups related to the specific stimuli, and this coexpression might contribute to the efficient response to the stress. Our results also suggest that the roles of transcription factors can be altered depending on the condition. Most transcription factors expanded or altered their targets in order to regulate the genes that are required for the specific condition, and this alteration of targets often entailed the switching of partner transcription factors and a change in nucleosome occupancy, which provide clues for understanding the rewiring mechanism of transcriptional network. Several possible factors might contribute to the condition-specific transcriptional regulation of diverse genes. For regulation of a specific group of genes, the promoters of the genes should become accessible and transcriptional machinery should be recruited to them. As we demonstrated, condition-specific binding of transcription factors is related to the change in nucleosome occupancy, which can be either a cause or a result of changing promoter specificity of the transcription factors. The change in nucleosome occupancy induced by chromatin remodeling in response to the specific cellular condition might cause the condition-dependent transcription factor binding [49]. On the other hand, the target specificity might be obtained by variable association with other transcription factors that can affect the nucleosome occupancy. Supporting this notion, some transcription factors, such as Rap1 and Msn2, are known to have a role in influencing the accessibility of promoters [50,51].

Interestingly, our results indicated distinct and interesting hypotheses regarding the condition-specific regulatory mechanisms, which are somewhat different from those of the previous studies. As mentioned before, Luscombe *et al*. [2] employed an invaluable approach for revealing the dynamic properties of transcriptional regulatory networks. Their findings are partially consistent with ours; enormous alteration of the targets of transcription factors occurred depending on the conditions, and greater numbers of target genes per transcription factor were observed in exogenous conditions (for example, stress response condition) than in endogenous conditions (for example, cell cycle condition). However, there was some discrepancy between our results and those of Luscombe *et al*. [2]. In our results, constancy in the number of transcription factors for regulating a certain functional gene group across all conditions was observed. In contrast, Luscombe *et al*. [2] concluded that endogenous conditions require more complex transcription factor combinations than exogenous conditions based on the greater 'in-degree' of endogenous conditions, implying that a larger number of transcription factors regulate a target gene. They also found significantly more FFLs in the endogenous transcriptional regulatory networks, while we found a much larger number of functional FFL groups in the exogenous conditions. This discrepancy might be due to the different definition of the 'stress response condition'. While we separately used only the small subsets of the microarray experiments (heat shock and nitrogen depletion) for analyzing the detailed features of the selected stress conditions, Luscombe *et al*. [2] considered all the various stress conditions included in the experiments of Gasch *et al*. [14] as the stress response condition; therefore, their results might reflect the regulatory events corresponding to 'general stress responses', which occur commonly in all stress conditions [14].

Although our approach deciphered the condition-specific regulatory mechanisms efficiently, it has some limitations. Our methodology depends largely on the quality and abundance of the known transcription factor binding motif information and ChIP-chip data; hence, the results would reflect only a part of the events actually happening in a cell. Moreover, the number of our tested conditions is too small to depict a wide variety of alteration in the regulatory mechanisms corresponding to various conditions. In the future, we would like to apply this method to other important conditions and perform putative motif analysis to identify additional novel regulatory factors. The increasing knowledge of regulatory mechanisms in various conditions together with in-depth studies focused on the comparison of the 'condition-specific' transcriptional modules revealed by more diverse condition-specific results will shed light on the highly sophisticated and elaborate regulatory mechanisms of transcription in yeast cells. Furthermore, with increasing ChIP-chip, mRNA expression data and reliable genome information about higher eukaryotes, the approach used in this work can be readily extended to study condition-, tissue- and developmental stage-specific transcriptional regulatory networks in diverse organisms.

## Conclusion

In this study, we aimed at deciphering the transcriptional regulatory mechanisms in yeast with two major perspectives; we focused on unveiling the dynamic nature of transcriptional rewiring entailed by the change in conditions and investigating multiple distinct coregulated functional parts existing in a coexpressed gene group. Consequently, we could detect several important features of the condition-specific transcriptional regulatory networks. First, the organization of the coexpressed gene modules is altered depending on the cellular conditions; many functional modules unique to each condition were identified, although some modules related to fundamental cellular processes were sustained over multiple conditions. Second, there are some specific functions emphasized in each condition, and the genes related to these functions tend to be coexpressed and, therefore, constitute relatively larger EPMs. Third, the reorganization of the transcriptional regulatory modules entail the alteration of targets and partner regulators of transcription factors, suggesting that rewiring of the transcriptional regulatory networks occurs due to the dynamic properties of the transcription factors. In fact, many FFL groups, which include a common transcription factor, exhibited distinct functional roles depending on various secondary transcription factors, leading to a conclusion that alteration of partner transcription factors can determine target specificity. Furthermore, our results indicate that several different transcription factors in an EPM can drive a similar expression pattern of their target genes, most probably by the involvement of 'master regulators' that hierarchically control distinct sets of transcription factors. Besides, many EPMs and RMs suggest novel regulatory mechanisms of various transcription factors, including their partnerships and target genes, in a condition-specific manner. These results provide reliable hypotheses for unveiling the condition-specific transcriptional regulatory networks and for studying the regulation of biological processes induced under specific conditions.

## Materials and methods

### Identification of initial module candidates

The first step in our method was integration of the genome-wide location data (ChIP-chip) and the gene expression data to identify IMCs. In this study, we used the ChIP-chip data [3] that contain the binding information of 204 transcription factors (although Harbison *et al*. [3] describe only 203 transcription factors), and the gene expression data under three experimental conditions - mitotic cell cycle (17 experiments) from Cho *et al*. [13] and heat shock (15 experiments) and nitrogen depletion (10 experiments) conditions from Gasch *et al*. [14]. Each of the identified IMCs satisfied three requirements: first, all genes should have common transcription factor binding evidence with a *p *value < 10^-3^; second, the pair-wise Pearson correlation coefficient (PCC) between two expression profiles should be >0.7; and third, there should be at least five element genes (Figure 1a). Eventually, the IMCs with similar mean expression profiles (PCC > 0.7) were grouped together and called preEPMs (Figure 1b). Determination of the appropriate cut-off values are described in Additional data file 13.

### Detecting over-represented motifs

To identify the over-represented motifs in each IMC, we used 152 known or putative motifs (corresponding to 110 transcription factors) obtained from SCPD (23 matrices) [15], TRANSFAC (27 matrices) [16] and Harbison *et al*. (102 matrices) [3]. A motif was considered over-represented in an IMC if the *p *value was found to be <0.01 in the *t*-test examining the difference between the distribution of maximum log-odds scores calculated for the promoters of the genes in the IMC and those calculated for the promoters of the genes that did not have sufficient binding evidence regarding the transcription factor of the IMC (*p *value > 0.95). In this study, the promoter region was defined as the 750 bp upstream sequences from the transcription start site. As usual, the log-odds score of a motif for a promoter sequence was calculated using the position-weight matrix constructed for each motif.

### Confirming motif evidence and ChIP-chip evidence

From the over-represented motifs, we further identified the motifs associated with the expression coherence in each IMC (Figure 1c). Specifically, we performed the hypergeometric test with the number of genes in the yeast genome (*G*), the number of yeast genes that contained a motif in their promoters (*B*), the number of yeast genes whose expression profiles were similar to that of the IMC (*g*), and the number of yeast genes whose expression profiles were similar to that of the IMC and contained the motif in their promoters simultaneously (*b*) using the equation:

$$p=\sum_{i=b}^{\min(B,g)}\frac{\left(\begin{array}{c} B\\ i \end{array}\right)\left(\begin{array}{c} G-B\\ g-i \end{array}\right)}{\left(\begin{array}{c} G\\ g \end{array}\right)}$$

This hypergeometric test measured the level of enrichment in a gene group whose expression profiles were similar to that of an IMC by genes that contained the motif in their promoters against the average number of genes that contained the motif in their promoters for a randomly selected gene group. Motifs with a *p *value < 0.01 were considered as biologically significant in this study.

For the ChIP-chip transcription factor that was used for identifying the IMC, we also examined the association between transcription factor binding and expression coherence using the hypergeometric test. In this case, *B *and *b *were calculated for the transcription factor binding target genes with a *p *value < 0.001 in the ChIP-chip data instead of the genes containing the motifs (Figure 1c).

### Identification of EPMs and RMs

All the IMCs without biologically significant regulators were eliminated, and the selected genes of a preEPM were combined to form an EPM (Figure 1d). The RMs were identified by grouping the regulators in each EPM according to the degree of overlap between their target genes (Figure 1e). We listed the non-redundant regulators in the IMCs and calculated the *OL *between the target gene sets for all the possible pairs of regulators in each EPM using the equation:

$$OL_{ij}=\left|S_{i}\cap S_{j}\right|/\sqrt{\left|S_{i}\right|•\left|S_{j}\right|}:$$

where *S*_*i *_and *S*_*j *_denote the target gene sets of regulators *i *and *j*, respectively.

An RM was identified by gathering the regulators and their target genes in an EPM, where all the *OL*s between any pair of target gene sets were >0.5. The cut-off value (*OL *of 0.5) guaranteed a sufficient diversity in functional enrichment for explaining the functional parts in detail and the low redundancy level of the RMs (Additional data file 14).

### Analysis of synergistic pairs

We defined a combination of transcription factors as a synergistic pair when the genes having evidence of both transcription factors showed a significantly more coherent gene expression pattern than the genes with evidence for only one transcription factor. There is a clear difference between sharing target genes (RMs) and synergism of a transcription factor pair; synergism might involve physical or genetic interaction between the transcription factors ('AND' logic), whereas overlap of target genes implies that the transcription factors might have similar regulatory roles regardless of significant dependency between them ('OR' logic). To identify synergistic pairs, all the pairs of over-represented motifs in each IMC were examined using the hypergeometric test. For a motif pair 'a and b', P(b|a, X) was considered the *p *value in the hypergeometric test examining the enrichment of genes with motif 'b' in the group of genes that have motif 'a' and show a similar expression pattern with an IMC X against the genes that have motif 'a' alone. We defined the motif pair 'a and b' as a synergistic pair when the value of P(b|a, X) or P(a|b, X) was <0.01. For each of the two motifs, we used the mean maximal log-odds score of the IMC X as the threshold score for deciding whether the motif exists in the promoter of a gene. The motif synergistic pairs detected in more than one IMC of each preEPM were considered reliable in this study. We also searched for transcription factor synergistic pairs by applying the same method to a pool of IMCs with a similar expression pattern and their related transcription factors.

### Functional enrichment analysis

We examined whether the genes of each EPM and the assigned target genes of each regulator in the EPM were significantly enriched by specific functional categories using the GO categories. Categories with a *p *value of <0.01, as revealed by the hypergeometric test, were considered statistically significant in this study, and the redundant categories were trimmed (for example, when 'response to pheromone', 'conjugation with cellular fusion' and 'response to pheromone during conjugation with cellular fusion' were simultaneously enriched, we adopted only one proper category). For Figure 2, we used only the GO 'biological process' categories, and a subset of the attributes was selected for their diversity.

## Abbreviations

ChIP-chip, chromatin immunoprecipitation microarray; EPM, expression pattern module; FFL, feed-forward loop; GO, Gene Ontology; IMC, initial module candidate; *OL*, overlap level; PCC, Pearson correlation coefficient; preEPM, precursor of expression pattern module; RM, regulator-set module.

## Authors' contributions

HGL and HSL conceived the study and developed the algorithm. WKH, SHJ and YSL managed and coordinated the project. WKH and THC contributed to drafting the manuscript by ongoing discussions. HGL, HSL and WKH participated in writing and revising the final manuscript. All authors read and approved the final manuscript.

## Additional data files

The following additional data are available with the online version of this paper. Additional data file 1 is a diagram describing the filtering steps for obtaining reliable candidate regulators. Additional data file 2 contains the matrices representing overall information about EPMs of the nitrogen depletion and the cell cycle conditions. Additional data file 3 contains detailed descriptions of condition-specific features of EPMs with their average gene expression profiles. Additional data file 4 contains the case studies that describe the RMs and their regulators in several cell cycle EPMs, heat shock EPM 0 and nitrogen depletion EPM 2. Additional data file 5 contains the matrices describing nitrogen depletion EPM 0, which includes various functional gene groups that formed several EPMs in other conditions. Additional data file 6 is a Venn diagram showing the numbers of overlapped regulators among three conditions. Additional data file 7 is a table listing enriched functional categories for the target genes of all confirmed transcription factors. Additional data file 8 includes bar graphs representing the relative nucleosome occupancy levels on the promoters of condition-specific target genes of Uga3 and Yap1. Additional data file 9 is a diagram showing the regulatory networks among transcription factors under the heat shock and the nitrogen depletion conditions. Additional data file 10 is a table listing enriched functional categories for the targets of FFL groups. Additional data file 11 contains the matrices describing all EPMs and RMs, including lists of synergistic pairs of regulators. Additional data file 12 is a table listing possible regulatory relationships among transcription factors in the same EPM. Additional data file 13 contains detailed descriptions of RMs in nitrogen depletion EPM 12 and a comparison with the modules determined using other algorithms. Additional data file 14 contains detailed descriptions of the criteria for choosing appropriate cut-off values.

## Supplementary Material

Additional data file 1 Filtering steps for obtaining reliable candidate regulators. Click here for file

Additional data file 2 Matrices representing overall information about EPMs of the nitrogen depletion and the cell cycle conditions. Click here for file

Additional data file 3 Detailed descriptions of condition-specific features of EPMs with their average gene expression profiles Click here for file

Additional data file 4 Case studies that describe the RMs and their regulators in several cell cycle EPMs, heat shock EPM 0 and nitrogen depletion EPM 2. Click here for file

Additional data file 5 Matrices describing nitrogen depletion EPM 0, which includes various functional gene groups that formed several EPMs in other conditions. Click here for file

Additional data file 6 Numbers of overlapped regulators among three conditions. Click here for file

Additional data file 7 Enriched functional categories for the target genes of all confirmed transcription factors. Click here for file

Additional data file 8 Relative nucleosome occupancy levels on the promoters of condition-specific target genes of Uga3 and Yap1 Click here for file

Additional data file 9 Regulatory networks among transcription factors under the heat shock and the nitrogen depletion conditions. Click here for file

Additional data file 10 Enriched functional categories for the targets of FFL groups. Click here for file

Additional data file 11 Matrices describing all EPMs and RMs, including lists of synergistic pairs of regulators. Click here for file

Additional data file 12 Possible regulatory relationships among transcription factors in the same EPM. Click here for file

Additional data file 13 Detailed descriptions of RMs in nitrogen depletion EPM 12 and a comparison with the modules determined using other algorithms. Click here for file

Additional data file 14 Criteria for choosing appropriate cut-off values. Click here for file
