# Supplementary material for: High-resolution analysis of condition-specific regulatory modules in Saccharomyces cerevisiae
Source: Genome Biol. 2008 Jan 3;9(1):R2. doi: 10.1186/gb-2008-9-1-r2 (PMC2395236; doi:10.1186/gb-2008-9-1-r2)
Supplement: Additional data file 1 — Filtering steps for obtaining reliable candidate regulators. [file gb-2008-9-1-r2-S1.pdf]

(a)

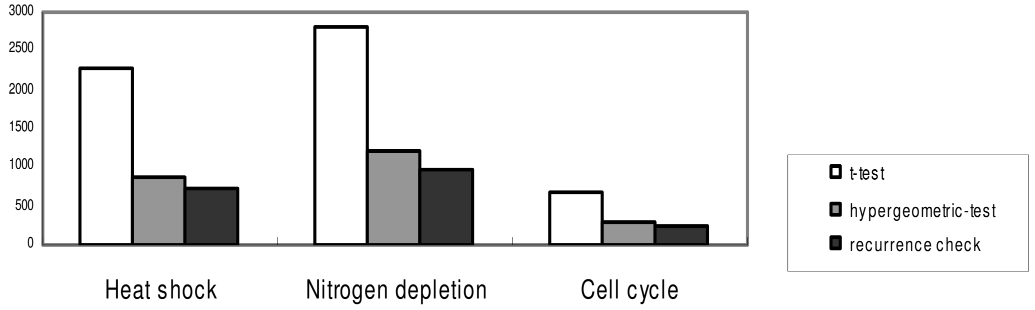

(b)

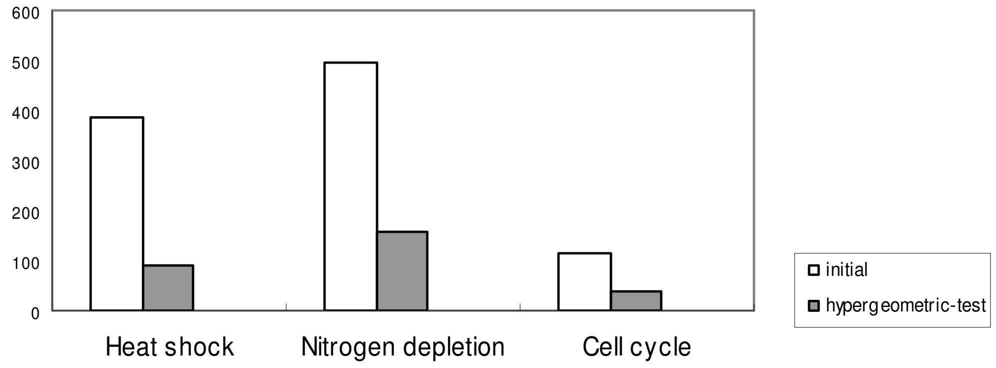

Additional data 1. Filtering candidate regulators

Bars shown in (a) and (b) represent numbers of motif evidence and ChIP-chip evidence, respectively, which survived from association test with expression patterns and recurrence test under each condition.
