# Supplementary figures and images for: High-resolution analysis of condition-specific regulatory modules in Saccharomyces cerevisiae
Source: Genome Biol. 2008 Jan 3;9(1):R2. doi: 10.1186/gb-2008-9-1-r2 (PMC2395236; doi:10.1186/gb-2008-9-1-r2)

Additional data 2.

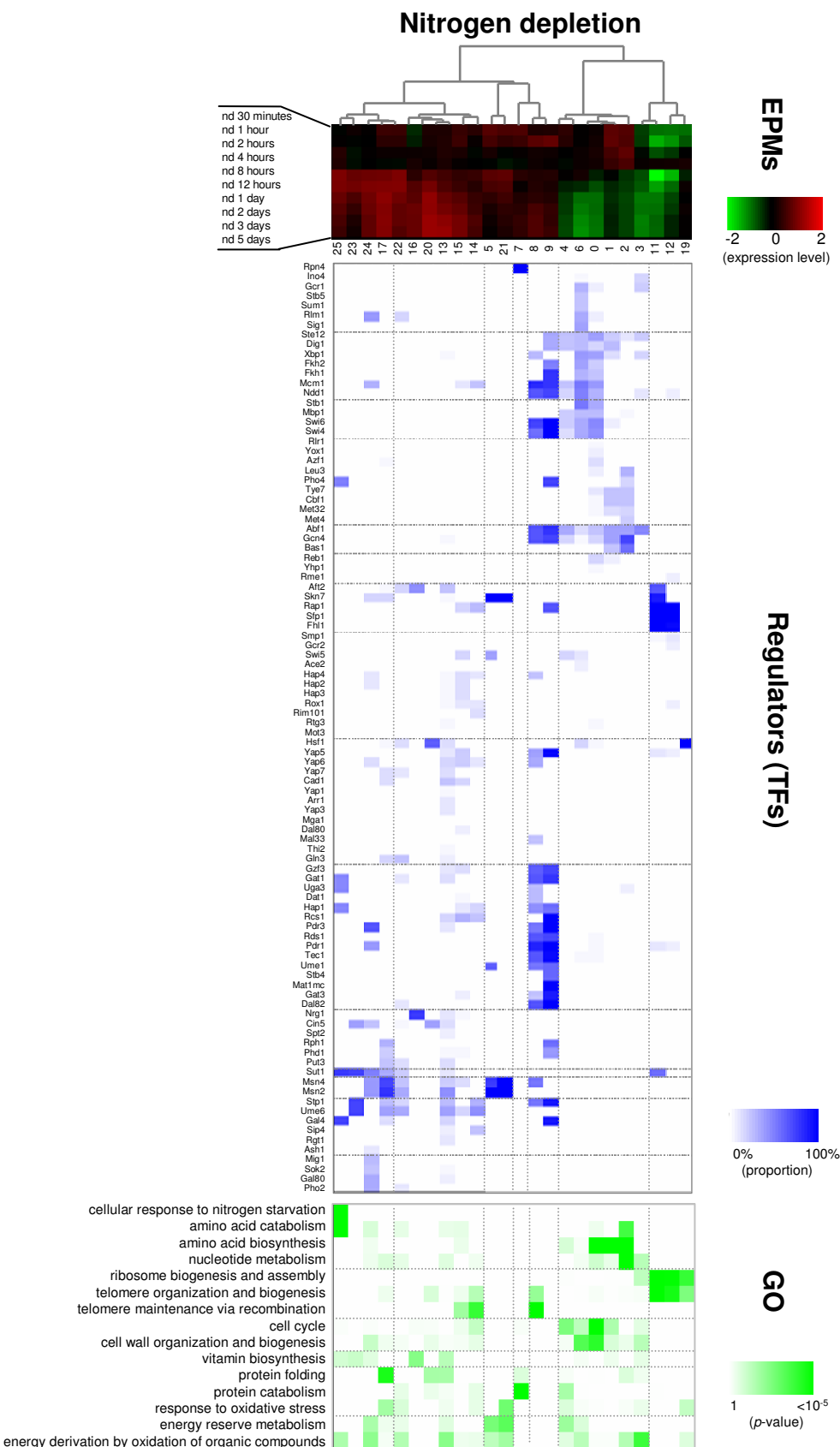

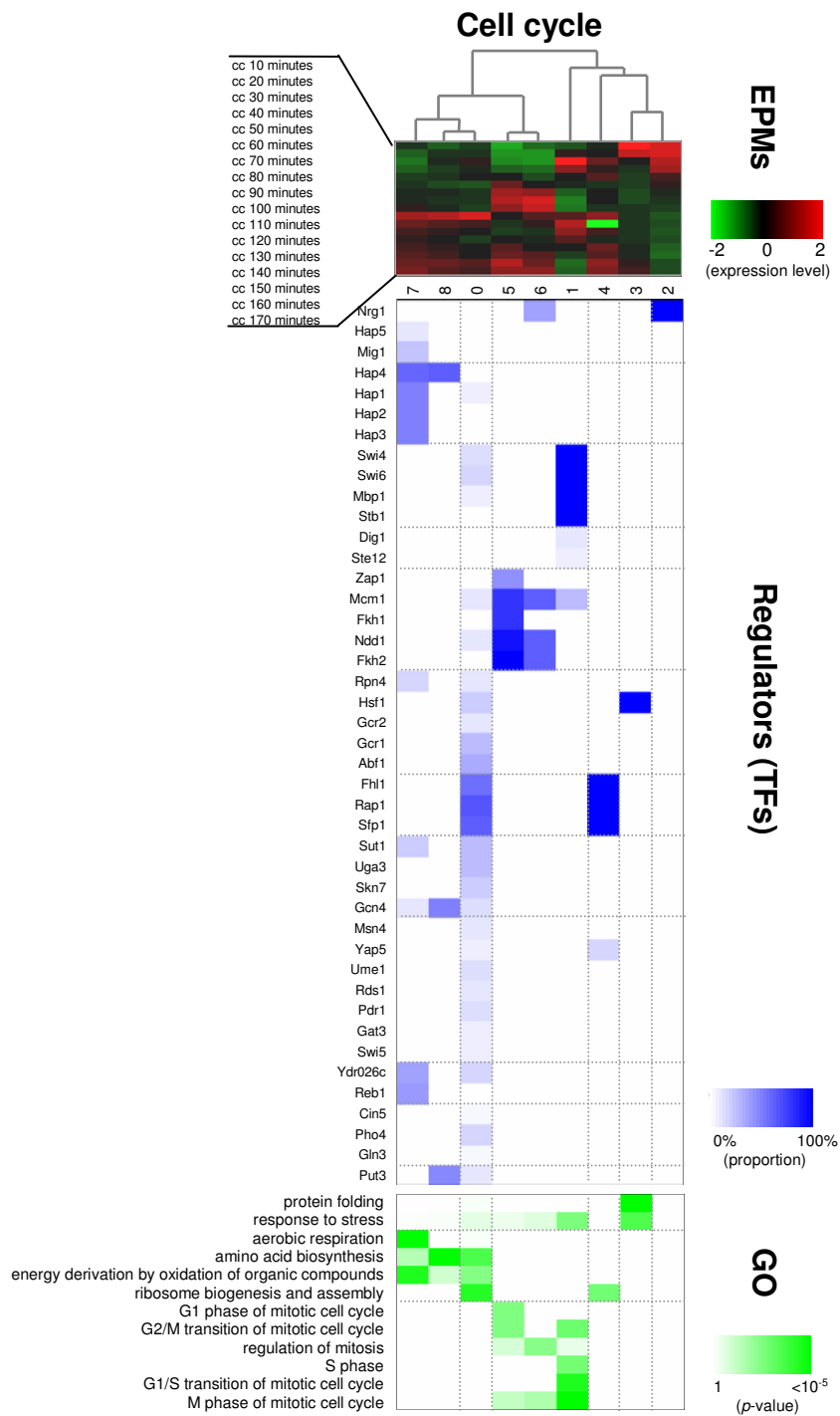

Supplement: Additional data file 2 — Matrices representing overall information about EPMs of the nitrogen depletion and the cell cycle conditions. [file gb-2008-9-1-r2-S2.pdf]

## Additional data 9. Regulatory networks among TFs

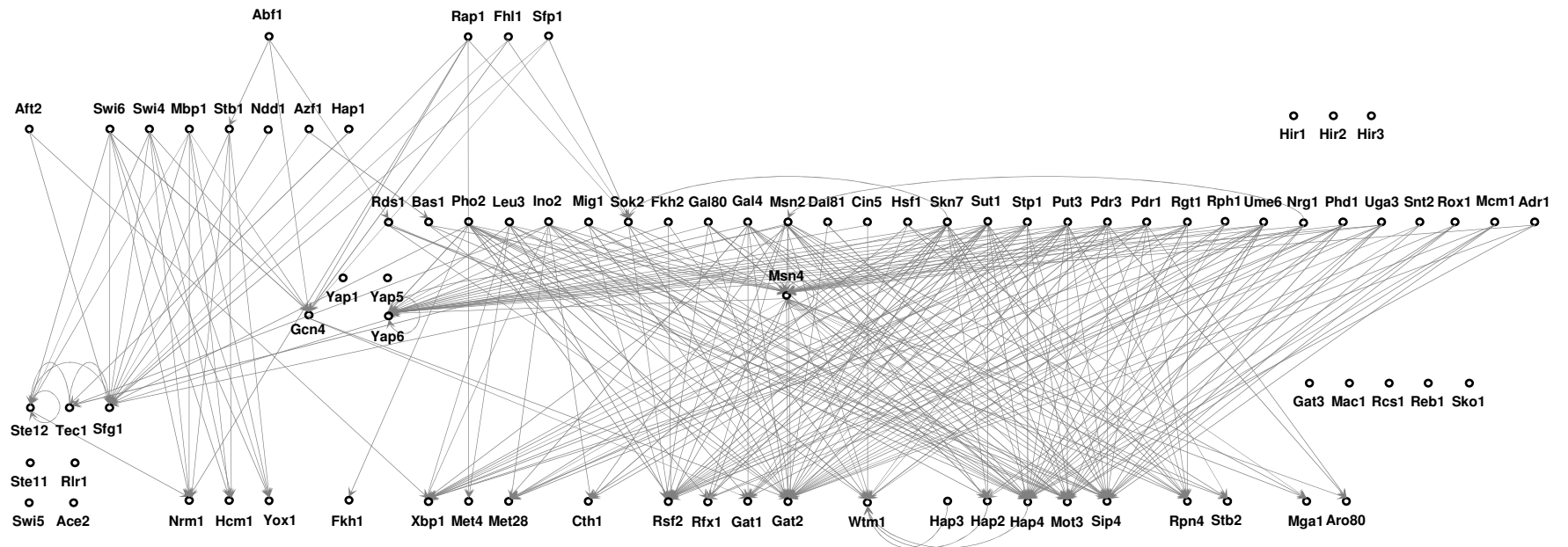

< Heat shock >

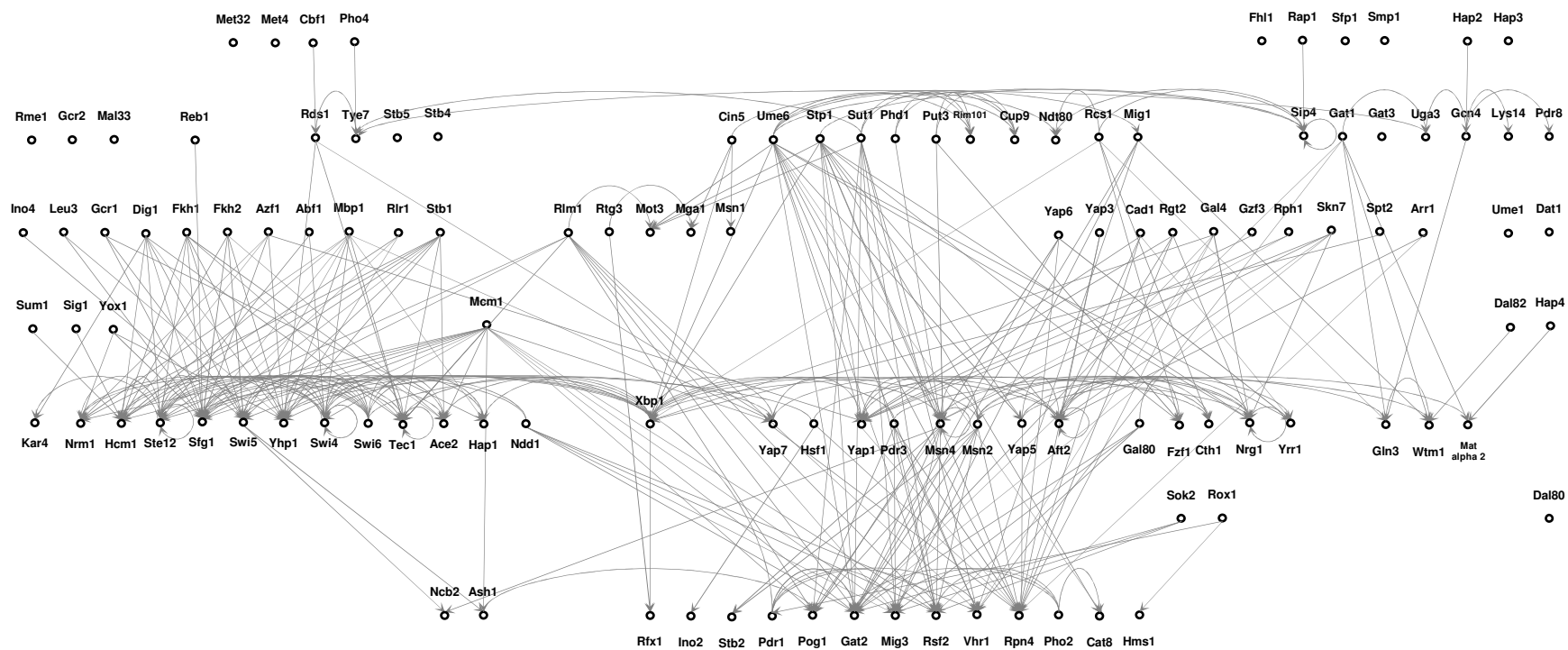

< Nitrogen Depletion >

Supplement: Additional data file 9 — Regulatory networks among transcription factors under the heat shock and the nitrogen depletion conditions. [file gb-2008-9-1-r2-S9.pdf]
