# Supplementary material for: High-resolution analysis of condition-specific regulatory modules in Saccharomyces cerevisiae
Source: Genome Biol. 2008 Jan 3;9(1):R2. doi: 10.1186/gb-2008-9-1-r2 (PMC2395236; doi:10.1186/gb-2008-9-1-r2)
Supplement: Additional data file 3 — Detailed descriptions of condition-specific features of EPMs with their average gene expression profiles [file gb-2008-9-1-r2-S3.pdf]

Additional data 3. Condition Specific Expression Profiles

1. Heat shock

A

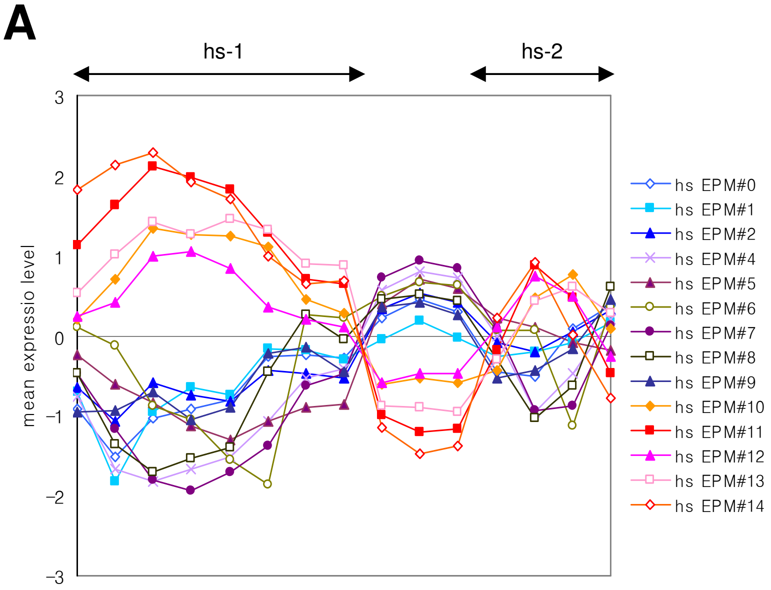

B hs-1

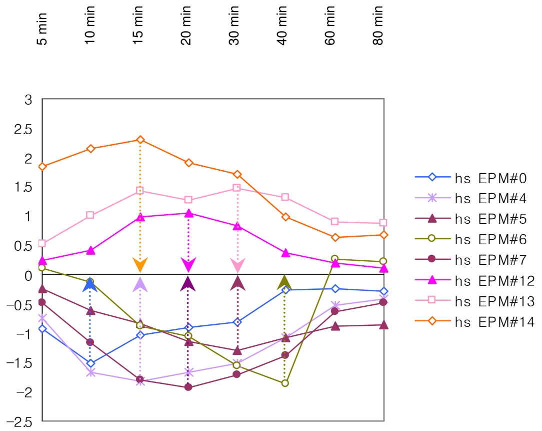

C hs-2

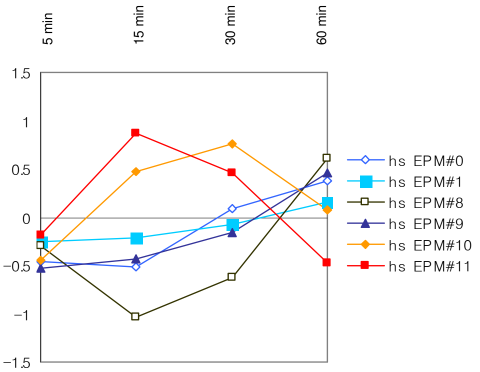

A.

Gasch et al. [14] conducted heat shock experiments in two different ways, referred to as hs-1 and hs-2. For the hs-1 experiments, YPD heated to 49℃ was added until the cell culture reached 37℃. For the hs-2, cultured cells were resuspended in an equal volume of media that had been preheated to 37℃. (As the experimental methods were slightly different, the ways of fluctuating expression profiles of genes displayed somewhat different features.)

B.

In hs-1, the peak points of down-regulated EPMs were different as their functionalities. It seems that the lowest peak point implies the starting point of adaptation, by raising their expression levels as time goes by. EPM#0, consisting of mating, cell wall, and cell cycle related genes, peaked at 10min. And three ribosome biogenesis EPMs (EPM#4, #5, #7) exhibited distinct peak points, which implies the differences of regulatory mechanisms exist among them. EPM#4, #7, and #5 reached at their lowest peak points at 15min, 20min, and 30min, respectively. Besides, EPM#6, containing histone genes, peaked at 40min and displayed abrupt increase.

In up-regulated EPMs, EPM#10, #11 and #14 (stress response EPMs) reached at their highest expression levels at 15min, while EPM#12, containing fermentation genes, peaked at 20min. EPM#13 whose member genes are involved in amino acid metabolism peaked at 30min.

C.

In hs-2, further discriminative features are observed which could not be observed in hs-1. Repressed EPMs of hs-1 exhibited slight increase after the adaptation to heat shock condition.

2. Nitrogen Depletion

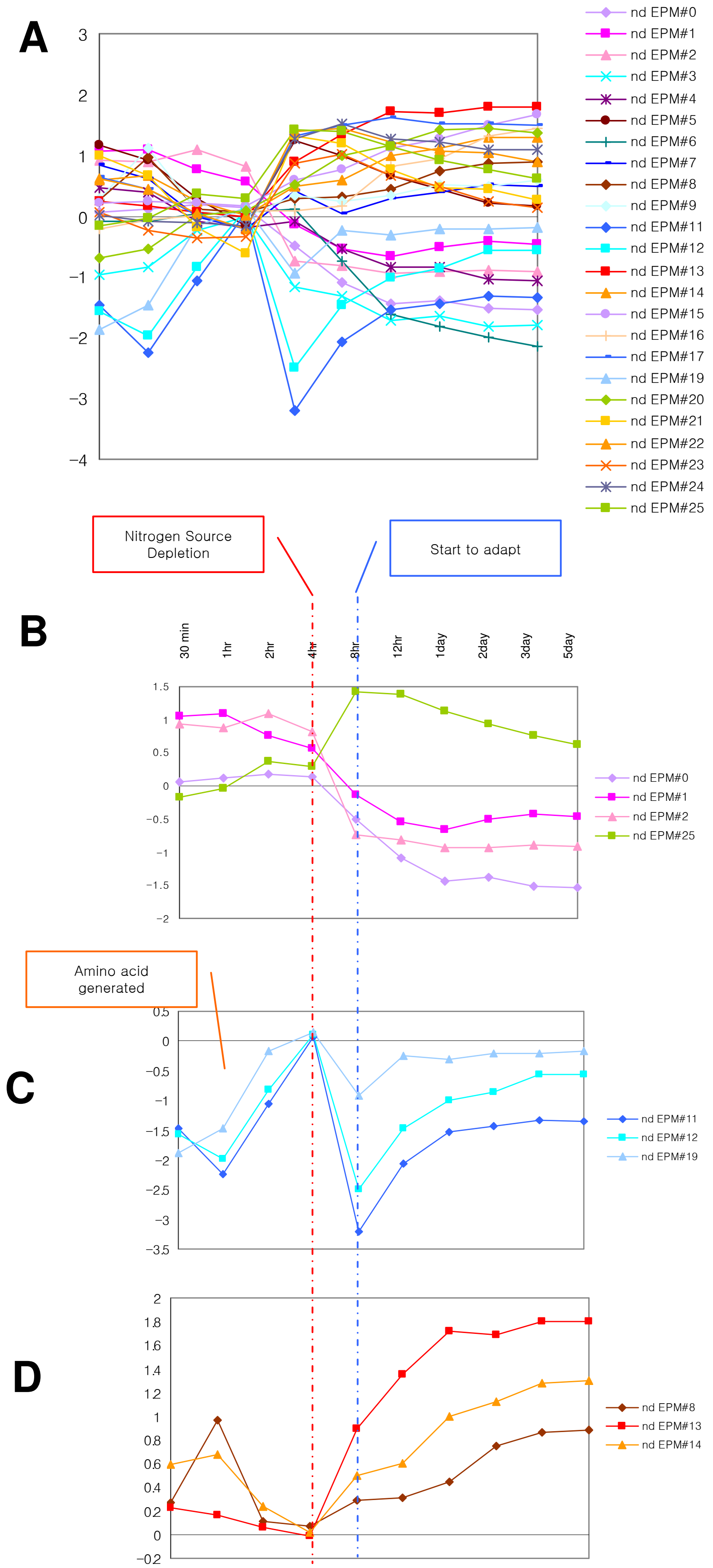

A.

For the nitrogen depletion experiments, Gasch et al. [14] used minimal media without amino acids or adenine and with limiting concetrations of ammonium sulfate. We conjectured that cells behaved as amino acid starvation response in the early time points because of the initial small amount of nitrogen source.

B.

EPM #1, #2, which contain amino acid biosynthetic genes, display induction until 4hr, and exhibit disrupt declination at 4hr. It seems that they are not able to use no more nitrogen sources at that time, because of nitrogen depletion. EPM#0 exhibits similar pattern with the two EPMs.

On the other hand, EPM#25 displayed reversed pattern. The genes in EPM#25, largely involved in response to nitrogen starvation, abruptly induced at 4hr, which implies the defense mechanism for nitrogen deprivation were turned on.

C.

Protein biosynthesis and ribosome biogenesis EPMs (EPM#11, #12, #19) seem to display the fluctuation dependent to amino acid availability. After 1hr, some amount of amino acids could be generated by amino acid biosynthetic EPMs (EPM#0,#1,#2), and the ribosome biogenesis EPMs start to be induced. And after nitrogen depletion(4hr), they abruptly repressed. The increase after 8hr seem to be caused by adaptation.

D.

EPM#8, #13, #14 includes some cell wall related genes, *FLO1. FLO10,* and *FLO9* , respectively, which are silenced at telomere in normal conditions and cause flocculation of haploid yeast cells. EPM#8 and #14 also includes several genes involved in telomere maintenance via recombination, which might contribute to the activation of the silenced *FLO* genes for enhancing the ability to penetrate into nutrient-rich media of the cells.

3. Cell cycle

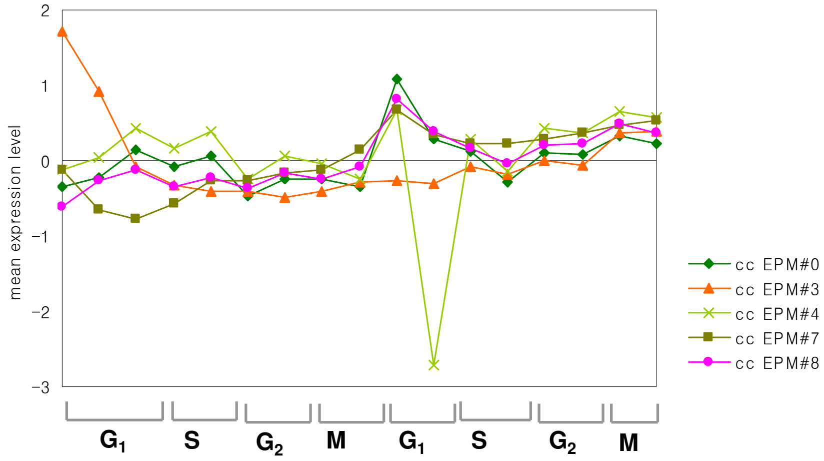

EPMs for ribosome biogenesis (EPM#0, #4), energy generation (EPM#7), and amino acid metabolism (EPM#8) peaked at G1 and G2/M transition phases, which seem to be required for growth check which determines whether further cell cycle progression would be occurred or not.

EPM#3, which includes protein folding genes, display abrupt declination at the start of experiments. It seems that the heat shock treatment for cell cycle synchronization affects the generation of EPM#3.
