# Supplementary material for: High-resolution analysis of condition-specific regulatory modules in Saccharomyces cerevisiae
Source: Genome Biol. 2008 Jan 3;9(1):R2. doi: 10.1186/gb-2008-9-1-r2 (PMC2395236; doi:10.1186/gb-2008-9-1-r2)
Supplement: Additional data file 4 — Case studies that describe the RMs and their regulators in several cell cycle EPMs, heat shock EPM 0 and nitrogen depletion EPM 2. [file gb-2008-9-1-r2-S4.pdf]

Additional data 4. Detailed case studies for condition-specific modules

1. Phase-specific cell cycle regulation: cell cycle EPM#1, EPM#5, and EPM#6

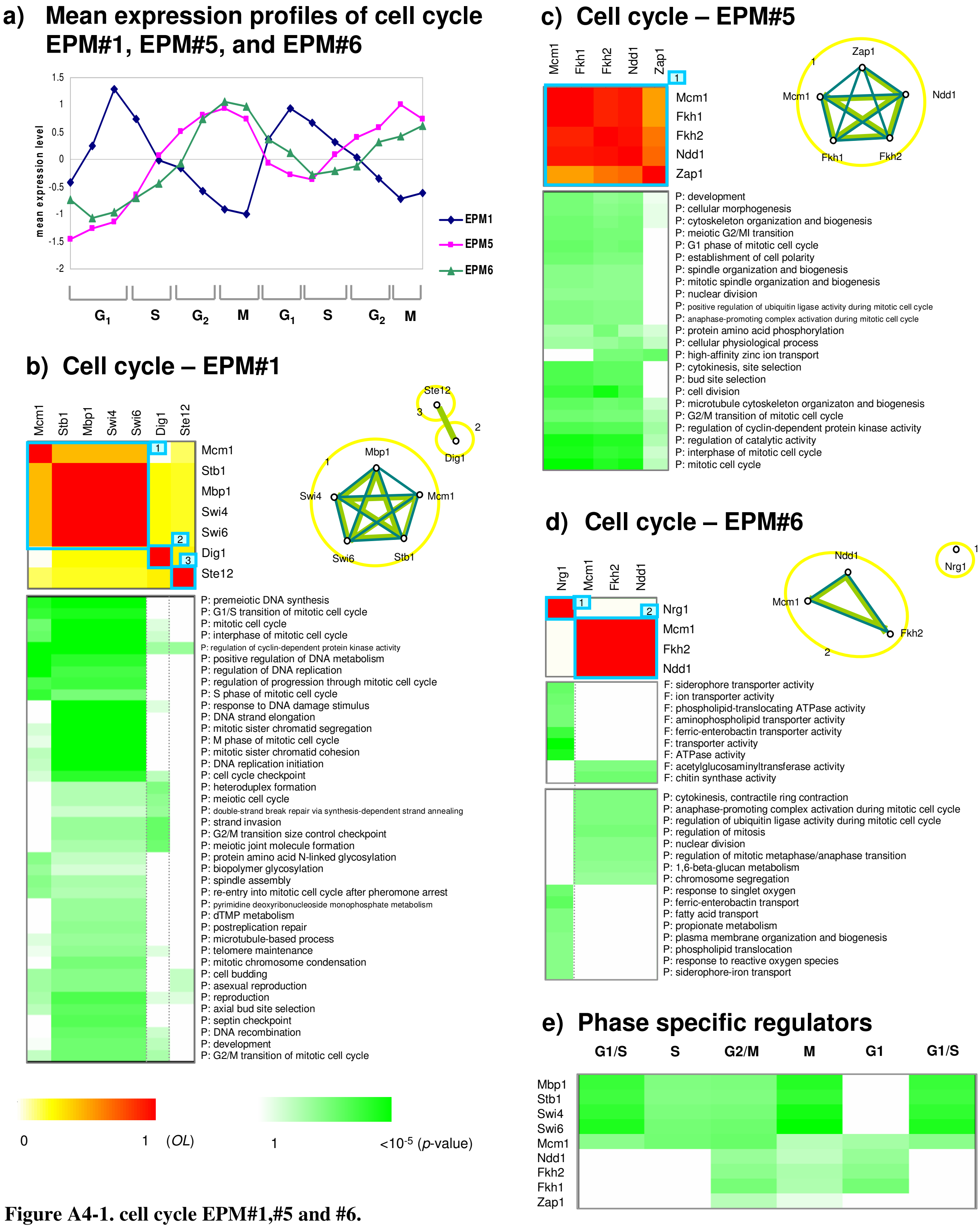

Figure A4-1. cell cycle EPM#1,#5 and #6.
a) Mean expression profiles of cell cycle EPM#1, EPM#5, and EPM#6. b) EPM#1 of cell cycle. c) EPM#5 of cell cycle. d) EPM#6 of cell cycle. e) Phase-specific enrichment of the target genes of cell cycle regulators. Only the regulators related to specific phases of cell cycle are shown.* P, F, and C are abbreviations for Biological Process, Molecular Function, and Cellular Component of the Gene Ontology categories, respectively

EPM#1, EPM#5, and EPM#6 of cell cycle condition showed detailed controlling mechanisms of mitotic cell cycle by representing phase-specific regulators and their synergistic relationships.

In EPM#1, there were three RMs whose common gene expression level peaked at the G1/S phase (Figure A4-1 a and b). All RMs include the genes annotated as 'P (Biological Process): regulation of cyclin-dependent protein kinase activity' that are required for the cell-cycle control system [52]. RM#1 was significantly enriched in the Gene Ontology categories corresponding to cell cycle phases from G1/S to mitosis. Other enriched Gene Ontology categories of the RM represented the events that occur in G1/S (corresponding to the START of cell cycle) and S phase, including 'P: re-entry into mitotic cell cycle', 'P: DNA replication initiation'. In fact, four of the five regulators in RM#1 (Mbp1, Swi4/6, and Stb1) are well-known TFs for G1/S phase in cell cycle. Many genes related to the G1/S transition are controlled by SBF (Swi4/6) and MBF (Mbp1/Swi6) [53]. Stb1, a Swi6-binding protein, is also involved in the G1/S transition [54]. Besides, MBF (Mbp1/Swi6) and Mcm1 have also been reported to participate in the S phase control. MBF stimulates expression of the genes involved in DNA synthesis and repair [55-57], and Mcm1 is reported to have a role in the efficient DNA replication initiation at the autonomously replicating sequences from which *S. cerevisiae* cells initiate DNA synthesis [58]. RM#1 was also enriched in the categories related to the G2/M transition. This result is supported by the previous reports that SBF and MBF also regulate expression of the G2/M cyclin (Clb2), which inhibits further expression of the G1/S cyclins (Cln1 and Cln2) [59] and promotes entry into mitosis [60]. Mcm1 has also been reported to participate in the G2/M-specific regulation [61]. RM#2 and RM#3, whose regulators are well-known TFs involved in mating process (Dig1 and Ste12, respectively) [62], commonly include the genes related to cell cycle along with RM#1 in our result, suggesting that their functional roles may also be involved in regulation of overall processes in cell cycle.

In the EPM#5, all the regulators shared the same target genes, generating only one RM, and the gene expression level peaked at the G2/M phase (Figure A4-1 a and c). This EPM included five regulators (Zap1, Mcm1, Fkh1/2, and Ndd1) whose predicted target genes were significantly enriched in categories that represent the G2/M transition, mitosis, and the G1 phase. Except Zap1, all regulators are known as the G2/M phase-specific regulators [63], and we also found many synergistic pairs among these regulators that are consistent with the known physical and genetic interactions: Fkh1-Fkh2, Fkh1-Ndd1, Fkh1-Mcm1, Fkh2-Mcm1, Fkh2-Ndd1, Mcm1-Ndd1 [63-68]. In addition, it has been reported that Mcm1, Fkh2, and Ndd1 regulate the genes whose expression is necessary for both entry into and exit from mitosis [69]. Moreover, Mcm1 has also been reported to participate in the M/G1-specific regulation [70,71]. Interestingly, Zap1 appeared to regulate the genes for 'P: high-affinity zinc ion transport' and 'P: G2/M transition of mitotic cell cycle' in our result. Zap1 is originally known as a zinc-responsive TF[72] which regulates the genes that control zinc homeostasis. And it has been reported that zinc deficiency disrupts cell cycle and arrests specific phases in other organisms [73,74]. By considering these, we suggest that Zap1 may affect cell cycle progression in *S. cerevisiae* by controlling zinc ion transportation.

EPM#6 consisted of two RMs, and three of its four regulators (Mcm1, Fkh2, and Ndd1) overlapped with those of EPM#5 (Figure A4-1 d). However, the mean expression pattern of EPM#6 changed more slowly than that of EPM#5 (Figure A4-1), and enriched Gene Ontology categories of EPM#6 genes were somewhat different from those of EPM#5 genes. Two RMs in EPM#6 appeared to be involved in mitosis, especially in cytokinesis. RM#1, whose regulators were Mcm1, Fkh2, and Ndd1, was enriched in 'P: regulation of mitotic metaphase/anaphase transition', 'P: cytokinesis, contractile ring contraction' and the categories related to cell wall biosynthesis including 'F (Molecular Function): chitin synthase activity' and 'P: 1,6-β-glucan metabolism', which is important for the process of cytokinesis [75]. This RM was also enriched in 'P: anaphase-promoting complex activation during mitotic cell cycle' indicating its involvement in the activation of the master regulator of mitosis and metaphase-to-anaphase transition [76]. RM#2, whose regulator was Nrg1, was enriched in 'P: phospholipid translocation', 'P: plasma membrane organization and biogenesis', and 'F: phospholipid translocating ATPase activity'. All of the enriched categories reflect the process of membrane transportation for cytokinesis. Membrane lipids supply and lipid bilayer rearrangement in an ATP-dependent manner are essential for cell division [77-79]. Through these findings, we could infer that RM#1 and RM#2 are participating in cytokinesis process in distinct ways, and Nrg1 may act as a regulator that controls the genes involved in the membrane lipid transport for cytokinesis. Notably, our results indicated that EPM#5 and EPM#6 exhibit distinct roles in mitosis although their regulators are quite similar. This example shows that the partnership of regulators can determine their regulatory roles along with the change in the expression pattern of their target genes; Mcm1, Fkh2, and Ndd1 are the common regulators in EPM#5 and EPM#6, but they are acting with Fkh1 and Zap1 only in EPM#5 by sharing target genes and displaying synergism.

To further investigate overall cell cycle phase-specific regulation, we gathered all predicted target genes of cell cycle regulators and looked into their enrichment in each mitotic cell cycle phase categories in the Gene Ontology (Figure A4-1 e). We note that the G1 and G1/S transition phase can be distinguished as the END and the START of cell cycle, respectively. From our result, we could conjecture that Mbp1, Swi4/6, and Stb1 regulate the genes related to cell cycle phase from the START (G1/S) to mitosis. Many studies suggest that these regulators control the genes related to the G1/S and S phase. However, our result suggested that these regulators take part in regulation of the genes related to not only the G1/S and S, but also the G2/M and M phase.

We also found that Mcm1, Fkh1/2, and Ndd1 regulate genes related to the G2/M, M, and G1 phase, the END point of cell cycle. All these TFs are originally well-known for the regulation of the G2/M transition phase. Additionally, our result suggested that these TFs may also take part in the mitotic exit, and several independent studies about these TFs support our result, as introduced before. Interestingly, Mcm1 was predicted to participate in the regulation throughout the cell cycle in our result. This is supported by several previous studies reporting that Mcm1 takes part in regulation of various cell cycle phase-related genes including the S [58], G2/M [61, 69], and M/G1 [69, 70]. In addition, another computational approach suggested that Mcm1 also controls the G1/S [80].

2. Mating and cell wall biogenesis: heat shock EPM#0

Heat shock EPM#0 is enriched in the 'P: sexual reproduction', 'P: conjugation with cellular fusion', and 'P: cell wall biosynthesis' (Figure A4-2). It is known that the polarized growth during mating projection involves new cell wall synthesis, a process that relies on activation of the cell integrity pathway [81]. This EPM shows a clear example of detailed delineation of functional subparts in the cell wall synthesis for mating projection formation process by several RMs.

Ste12, Tec1, Swi4, and Swi6, regulators of RM#2 and RM#5, appeared to regulate the genes in 'P: response to pheromone' and 'C (Cellular Component): mating projection tip'. As mentioned before, Ste12 is well known to regulate mating-related genes, and Tec1 is also reported to be related to the mating factor response pathway [82]**.** And it is known that SBF (Swi4 and Swi6) participates in the cell integrity pathway which maintains cell wall integrity during polarized growth [83].

Most RMs were found to include cell wall biosynthetic genes, and several combinations of the RMs appeared to assume some distinct parts of the cell wall biogenesis process, which could be divided into two major subparts corresponding to two layers of cell walls: the inner layer consisting of glucan polymer and chitin, and the outer layer containing highly glycosylated mannoproteins [84, 85]. For inner layer, RM#2 (Ste12) was enriched in the 'P: cell wall chitin biosynthesis' and RM#3 (Mbp1 and Stb1) in the 'P: glucan metabolism'. And, RM#1, RM#2, RM#3, and RM#4, whose regulators were Abf1, Ste12, Mbp1, Stb1, and Swi4/6, were including some genes for 'P: protein amino acid glycosylation', which reflects generation of glycosylated mannoprotein comprising outer layer. Among the glycoprotein synthesis part of the outer layer, RM#1 (Abf1) was enriched in the 'F: α-1,6-mannosyltransferase activity'.

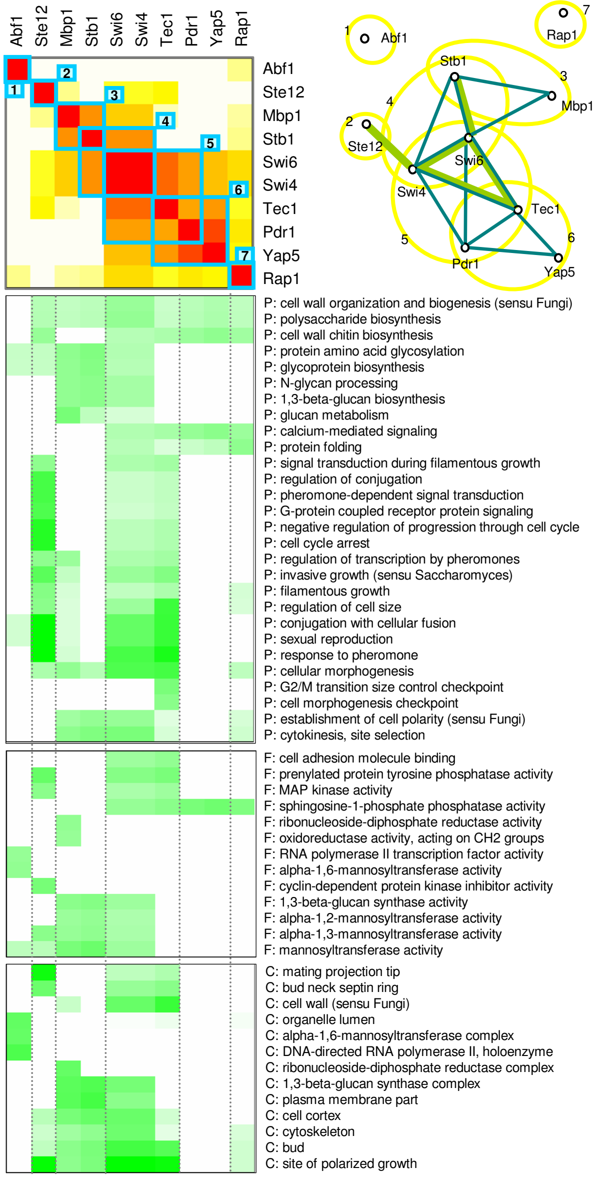

Figure A4-2. Heat shock EPM#0.

Besides, RM#2, RM#3, RM#4, and RM#5 were found to include the genes whose products are localized in the 'C: site of polarized growth', which implies their involvement in polarized growth during mating [86]. The roles of the predicted regulators were largely consistent with the previous reports that SBF (Swi4/6), Mbp1, Ste12, and Tec1 have roles in cell wall metabolism [46, 47, 87, 88]. Besides, our result suggested that Abf1, Pdr1, Yap5, and Rap1 also have a role in regulation of cell wall biosynthetic genes under heat shock condition.

3. Amino acid metabolism: nitrogen depletion EPM#2

In the EPM#2 on nitrogen depletion condition, twelve transcription factors were identified as regulators of this EPM. By calculating the overlap matrix of target genes of the regulators, we could find six RMs, each of which shared highly overlapped target genes, and seven synergistic pairs in this EPM. Figure A4-3 shows how well RMs are defined as functional subparts in the EPM#2. The enriched categories indicated that member genes of this EPM were largely involved in cell wall biosynthesis and amino acid metabolism; of twelve transcription factors found to regulate this EPM, two transcription factors were assigned to two RMs related to cell wall biosynthesis (RM#1 and #2) and all the other ten transcription factors were assigned to four RMs that were enriched with various amino acid metabolism-related genes (RM#3, #4, #5 and #6).

In *S. cerevisiae*, polarized growth is required for projection formation during mating response [89], and cell wall synthesis is involved in the polarized growth [85]. It seems that RM#1 and RM#2 reflects the cell wall biosynthetic process during mating response; they commonly included several genes annotated as 'cell wall biosynthesis'. RM#1, whose regulator was Ste12, also appeared to be enriched in 'response to pheromone' and 'cell wall chitin biosynthesis'. In agreement with our results, Ste12 has been shown to be involved in mating [90-92] and bind to the promoters of several cell wall genes [93]. The member genes of RM#2, regulated by Mbp1, were enriched in 'phospholipids transport', 'phosphatidylinositol metabolism' and 'β-glucan biosynthesis', which indicated their involvement in the process of generating cell wall. This observation is supported by the fact that glycosyl-phosphatidylinositol proteins comprise the outer cell wall layer [94] and β-glucan is one of the major components of cell wall [85], and the experimental evidence indicating the role for Mbp1 in cell wall metabolism [88].

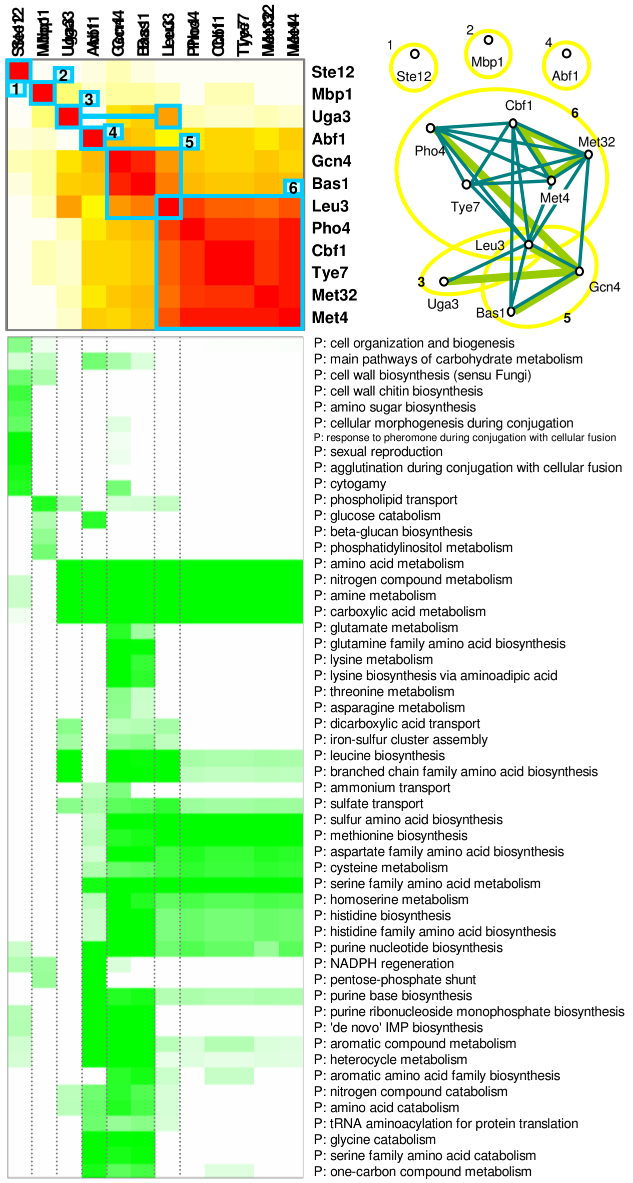

Figure A4-3. Nitrogen depletion EPM#2.

Four RMs, from RM#3 to RM#6, were commonly enriched in 'amino acid metabolism' and 'nitrogen compound metabolism'. And each or some combination of the RMs appeared to be enriched in several specified amino acid metabolism-related categories, which indicates that amino acid metabolism can be divided into several subparts. Especially, Gcn4 and Bas1 in RM#5 often acted with the regulators of other RMs to participate in the regulation of various amino acid metabolisms.

RM#5 was enriched in the Gene Ontology categories related to amino acid and purine nucleotide metabolism, and contained three regulators, Gcn4, Bas1 and Leu3. All these regulators have been reported as main transcriptional regulators of amino acid metabolism; Gcn4 is a regulator of almost all kinds of amino acid metabolism and purine nucleotide metabolism [95-98]; Bas1 is also known to control some amino acids and purine biosynthesis with Gcn4 [90-101]; Leu3 is known to activate genes involved in the metabolism of branched chain amino acids and ammonia assimilation [102, 103]. RM#3 and RM#5 were commonly enriched in 'branched chain family amino acid biosynthesis'. RM#3 had two regulators, Leu3 and Uga3. As mentioned before, Leu3 is a known regulator for branched chain amino acid metabolism. And Uga3, which is a known regulator for nitrogen utilization that activates the genes of the ɣ-aminobutyrate utilization pathway [104], was also suggested to be a regulator of branched chain amino acid metabolism in our results.

RM#4, RM#5 and RM#6 commonly included the genes in 'sulfur amino acid biosynthesis', 'methionine biosynthesis' and 'cysteine metabolism', which indicates their involvement in sulfur amino acid metabolism. They also appeared to include the genes in 'purine nucleotide biosynthesis' and 'histidine biosynthesis', which are functionally related not only to each other but also to the sulfur amino acid metabolism; purine metabolism shares pathway with histidine metabolism through a common metabolic intermediate 5´-phosphoribosyl-4-carboxamide-5-aminoimidazole [105], whose accumulation interferes with a step in methionine biosynthesis [106]. In addition, RM#4 and RM#5 were enriched in 'one-carbon compound metabolism', 'glycine catabolism' and 'serine family amino acid catabolism'. It is known that both glycine and serine are primary one-carbon sources for purine biosynthesis [107]. Most of the predicted transcription factors for the three RMs have literature evidence for their regulatory roles in the enriched categories. Abf1, a regulator of RM#4, is known to regulate the adenine biosynthetic process with Bas1 [101]. Among 6 regulators of RM#6, Cbf1, Met4 and Met32 are well known for transcriptional regulation of sulfur amino acid metabolic pathways. Their physical interactions have also been reported [108], and our analysis results for synergism included all the pairs of the three regulators. Some other regulators in RM#6 have also been reported to be related to the control of sulfur amino acid metabolism. There is a report that overexpression of Pho4 can suppress methionine auxotrophy of a *cbf1* mutant [109], which implies the involvement of Pho4 in sulfur-related metabolism in the absence of Cbf1. In addition, other relevant computational approaches for revealing the combinations of transcription factors through module analysis suggested Tye7 as a co-regulator of Cbf1 [110, 111].
