# Supplementary material for: High-resolution analysis of condition-specific regulatory modules in Saccharomyces cerevisiae
Source: Genome Biol. 2008 Jan 3;9(1):R2. doi: 10.1186/gb-2008-9-1-r2 (PMC2395236; doi:10.1186/gb-2008-9-1-r2)
Supplement: Additional data file 5 — Matrices describing nitrogen depletion EPM 0, which includes various functional gene groups that formed several EPMs in other conditions. [file gb-2008-9-1-r2-S5.pdf]

Additional data 5. Co-expression of diverse functional modules in a specific condition

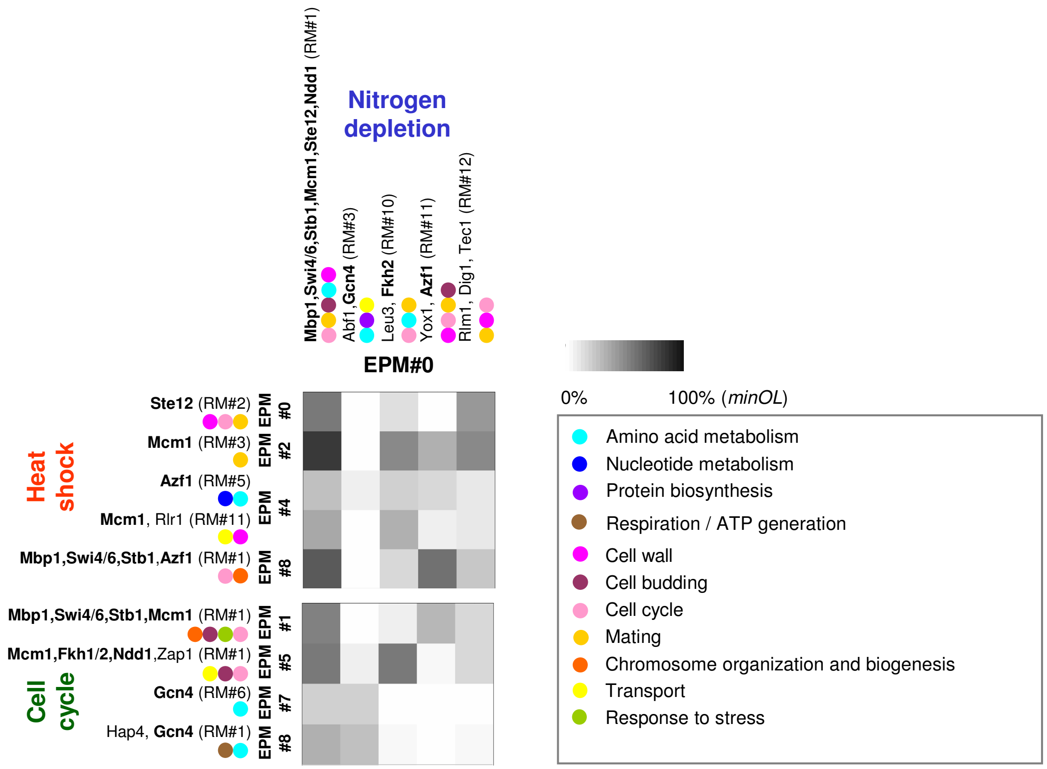

In nitrogen depletion condition, a number of genes related to various functions including amino acid and nucleotide metabolism, mating, cell wall, and cell cycle are co-expressed so as to be organized as a large EPM, nitrogen depletion EPM#0. The functional gene groups included in the EPM formed multiple EPMs in other conditions (heat shock and cell cycle condition). The *minOL* is used for the purpose of showing the degree of inclusion.
