# Supplementary material for: High-resolution analysis of condition-specific regulatory modules in Saccharomyces cerevisiae
Source: Genome Biol. 2008 Jan 3;9(1):R2. doi: 10.1186/gb-2008-9-1-r2 (PMC2395236; doi:10.1186/gb-2008-9-1-r2)
Supplement: Additional data file 6 — Numbers of overlapped regulators among three conditions. [file gb-2008-9-1-r2-S6.pdf]

Additional data 6. Overall statistics of confirmed TFs for all conditions

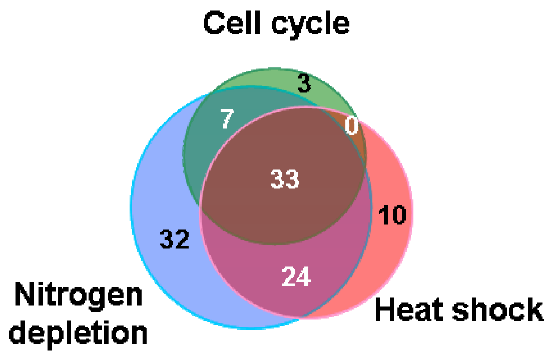

Total TF number	109
Heat shock	67
Nitrogen depletion	96
Cell cycle	43
