# Supplementary material for: High-resolution analysis of condition-specific regulatory modules in Saccharomyces cerevisiae
Source: Genome Biol. 2008 Jan 3;9(1):R2. doi: 10.1186/gb-2008-9-1-r2 (PMC2395236; doi:10.1186/gb-2008-9-1-r2)
Supplement: Additional data file 7 — Enriched functional categories for the target genes of all confirmed transcription factors. [file gb-2008-9-1-r2-S7.pdf]

Additional data 7. Enriched functional categories of all TFs

TF (overall *OL*)	cond.	GO biological process	source
confirmed in all three conditions (hs, nd, and cc)			
ABF1 (0.1483)	cc	P:telomere maintenance via recombination (7.45E-09)	
		P:pyruvate metabolism (1.28E-06)	
		P:generation of precursor metabolites and energy (0.000174481)	
		P:amino acid biosynthesis (0.002346199)	
	hs	P:ribosome biogenesis and assembly (4.85E-11)	[113]
		P:amino acid metabolism (2.16E-05)	
		P:tRNA aminoacylation (0.000568016)	
		F:DNA-directed RNA polymerase activity (3.86E-09)	
	nd	P:amino acid metabolism (4.45E-20)	
		P:purine base metabolism (2.88E-09)	
		P:tRNA aminoacylation (1.17E-05)	
		P:NADPH regeneration (0.000119622)	
CIN5 (0.0910)	cc	P:glucose catabolism to ethanol (0.00372245)	
		P:glutamine biosynthesis (0.000745379)	
	hs	P:glycogen metabolism (0.000138358)	
		F:glucosidase activity (6.80E-05)	
	nd	P:biological process unknown (4.61E-07)	
		P:response to stress (0.004125134)	[112], [114]
FHL1 (0.6270)	cc	P:protein biosynthesis (1.03E-68)	
		P:protein complex assembly (2.66E-07)	
		P:ribosome biogenesis and assembly (6.11E-07)	[112]
		P:telomere organization and biogenesis (8.80E-06)	
	hs	P:protein biosynthesis (2.51E-89)	
		P:ribosome biogenesis and assembly (3.90E-16)	
		P:protein complex assembly (8.05E-10)	
		P:telomere organization and biogenesis (2.41E-06)	
	nd	P:protein biosynthesis (3.51E-123)	
		P:ribosome biogenesis and assembly (6.13E-13)	
		P:protein complex assembly (3.38E-10)	
		P:telomere organization and biogenesis (1.50E-07)	
FKH2 (0.1134)	cc	P:cytokinesis (1.65E-07)	
		P: regulation of progression through cell cycle (0.0004058478)	[115]
		P:G2/M transition of mitotic cell cycle (0.005879056)	
	hs	P:lysosome organization and biogenesis (0.002236136)	
		P:glucose 6-phosphate metabolism (0.004467604)	
		P:glucose 1-phosphate metabolism (0.004467604)	
	nd	P:cytokinesis (5.29E-05)	
		P:spindle organization and biogenesis (0.000170514)	
		P:interphase of mitotic cell cycle (0.000174912)	
		P:cell wall organization and biogenesis (0.000448491)	
GAT3 (0.2794)	cc	P:telomere maintenance via recombination (9.89E-13)	no annotation
	hs	P:biological process unknown (0.000510403)	
	nd	P:telomere maintenance via recombination (2.36E-07)	
		P:biological process unknown (0.000199891)	
GCN4 (0.1352)	cc	P:amino acid metabolism (1.77E-11)	[112]
		P:telomere maintenance via recombination (3.46E-09)	
	hs	P:amino acid metabolism (2.50E-06)	
		P:histone dephosphorylation (0.007139643)	
		P:amino acid metabolism (2.80E-56)	

	nd	P:nucleotide biosynthesis (7.35E-08)	
		P:tricarboxylic acid cycle intermediate metabolism (0.000238118)	
		P:telomere maintenance via recombination (0.00074303)	
HAP1 (0.1242)	cc	P:ATP synthesis coupled electron transport (1.05E-16)	[112]
		P:aerobic respiration (2.28E-05)	
		P:sterol metabolism (lipid biosyn.) (3.12E-05)	
	hs	P:ATP synthesis coupled electron transport (4.51E-14)	
		P:aerobic respiration (2.76E-09)	
		P:heme metabolism (0.002314036)	
	nd	P:telomere maintenance via recombination (1.63E-08)	
		P:sterol metabolism (0.001909559)	
		P:ATP synthesis coupled electron transport (0.00780255)	
HAP2 (0.1741)	cc	P:ATP synthesis coupled electron transport (4.98E-18)	[112]
	hs	P:ATP synthesis coupled electron transport (2.67E-07)	
		P:chronological cell aging (0.000582)	
		P:aerobic respiration (0.001100007)	
		P:gluconeogenesis (0.002372348)	
	nd	P:ATP synthesis coupled electron transport (2.44E-10)	
	nd	P:telomere maintenance via recombination (0.00170189)	
HAP3 (0.1591)	cc	P:ATP synthesis coupled electron transport (4.98E-18)	[112]
	hs	P:ATP synthesis coupled electron transport (2.67E-07)	
		P:chronological cell aging (0.000582)	
		P:aerobic respiration (0.001100007)	
		P:gluconeogenesis (0.002372348)	
	nd	P:ATP synthesis coupled electron transport (9.19E-09)	
		P:telomere maintenance via recombination (0.001180736)	
		P:ATP synthesis coupled proton transport (0.001752568)	
HAP4 (0.1553)	cc	P:ATP synthesis coupled electron transport (9.80E-18)	[112]
		P:aerobic respiration (5.62E-06)	
		P:amino acid biosynthesis (3.79E-05)	
		P:ATP synthesis coupled proton transport (0.000146038)	
		P:tricarboxylic acid cycle intermediate metabolism (0.006117622)	
	hs	P:ATP synthesis coupled electron transport (2.67E-07)	
		P:chronological cell aging (0.000582)	
		P:aerobic respiration (0.001100007)	
		P:gluconeogenesis (0.002372348)	
	nd	P:ATP synthesis coupled electron transport (1.81E-09)	
		P:telomere maintenance via recombination (1.15E-06)	
		P:ATP synthesis coupled proton transport (0.000135819)	
		P:mating type switching (0.004677589)	
HSF1 (0.2021)	cc	P:telomere maintenance via recombination (3.11E-11)	
		P:protein folding (6.94E-08)	[116]
		P:glycolysis (gluconeogenesis) (2.52E-06)	
		P:energy derivation by oxidation of organic compounds (3.32E-05)	
		P:generation of precursor metabolites and energy (9.92E-05)	
	hs	P:protein folding (1.61E-21)	
		P:SRP-dependent cotranslational protein targeting to membrane, translocation (2.02E-07)	
		P:intracellular protein transport (0.001847964)	
		P:proteasome assembly (0.002353463)	
		P:protein targeting (0.003476779)	
		P:chromatin assembly or disassembly (0.006144569)	
	nd	P:protein folding (1.31E-08)	
		P:glutamine family amino acid metabolism (0.001259295)	
		P:proteasome assembly (0.003479314)	

		P:allantoin metabolism (0.005153888)	
		P:SRP-dependent cotranslational protein targeting to membrane (0.005258995)	
MBP1 (0.3676)	cc	P:DNA replication (2.68E-12)	
		P:mitotic cell cycle (6.42E-09)	[112]
		P:regulation of cyclin-dependent protein kinase activity (1.60E-07)	
		P:cell cycle checkpoint (1.00E-05)	
		P:response to stress (0.0008657)	
		P:cell budding (0.008870957)	
	hs	P:DNA replication (2.54E-07)	
		P:regulation of cyclin-dependent protein kinase activity (1.08E-05)	
		P:protein biosynthesis (0.000124828)	
		P:mitotic cell cycle (0.000346003)	
		P:sterol biosynthesis (0.005429651)	
	nd	P:mitotic cell cycle (5.25E-09)	
		P:cell wall organization and biogenesis (5.49E-09)	
		P:regulation of cyclin-dependent protein kinase activity (2.50E-07)	
		P:establishment and/or maintenance of cell polarity (5.03E-07)	
		P:cell budding (1.33E-06)	
		P:cell cycle checkpoint (0.000104041)	
		P:DNA replication (0.000700144)	
		P:sexual reproduction (0.002335527)	
		P:signal transduction during filamentous growth (0.006663571)	
MCM1 (0.1953)	cc	P:telomere maintenance via recombination (5.20E-10)	
		P:mitotic cell cycle (1.60E-06)	[112]
		P:establishment of cell polarity (0.009221478)	
	hs	P:response to pheromone during conjugation with cellular fusion (0.000289443)	
		P:cell wall organization and biogenesis (0.001227871)	
		P:iron-sulfur cluster assembly (0.004705398)	
		P:cyclin catabolism (0.006807503)	
		P:regulation of cyclin-dependent protein kinase activity (0.009258796)	
	nd	P:sexual reproduction (7.01E-10)	
		P:mitotic cell cycle (9.93E-08)	
		P:response to pheromone (1.68E-07)	
		P:cell wall organization and biogenesis (5.71E-07)	
		P:establishment of cell polarity (0.000204818)	
		P:cell budding (0.000311685)	
MIG1 (0.0350)	cc	P:ATP synthesis coupled electron transport (2.31E-08)	
		P:aerobic respiration (0.000199583)	[117]
		P:energy derivation by oxidation of organic compounds (0.002981965)	
	hs	P:glycogen metabolism (1.05E-05)	
		P:energy derivation by oxidation of organic compounds (0.000125658)	
		P:response to oxidative stress (0.003018826)	
	nd	P:regulation of glycogen catabolism (0.007138049)	
		P:fatty acid transport (0.007138049)	
		P:biotin biosynthesis (0.008915248)	
MSN4 (0.1533)	cc	P:telomere maintenance via recombination (1.18E-06)	
		P:response to oxidative stress (0.003843926)	
	hs	P:generation of precursor metabolites and energy (1.19E-14)	
		P:response to stress (3.17E-12)	[112]
		P:protein folding (1.84E-09)	
		P:glycogen metabolism (6.11E-07)	
		P:ATP synthesis coupled electron transport (4.10E-05)	
		P:SRP-dependent cotranslational protein targeting to membrane, translocation (5.66E-05)	
		P:trehalose metabolism (0.000176794)	

[illegible]		P:aerobic respiration (0.000222821)	
		P:chronological cell aging (0.000255677)	
		P:pentose-phosphate shunt, oxidative branch (0.001085942)	
	nd	P:response to stress (4.70E-05)	
		P:glycogen metabolism (0.000169711)	
		P:biological process unknown (0.000503307)	
		P:telomere maintenance via recombination (0.000595455)	
		P:protein folding (0.006561522)	
NDD1 (0.2086)	cc	P:telomere maintenance via recombination (8.82E-11)	
		P:cytokinesis (2.02E-05)	
		P:mitotic cell cycle (0.000410768)	[112]
		P:regulation of cyclin-dependent protein kinase activity (0.0030155)	
	hs	P:regulation of cyclin-dependent protein kinase activity (2.84E-05)	
		P:cell wall organization and biogenesis (0.000568016)	
	nd	P:cytokinesis (1.31E-08)	
		P:cell wall organization and biogenesis (1.70E-08)	
		P:regulation of cyclin-dependent protein kinase activity (4.39E-08)	
		P:mitotic cell cycle (9.33E-07)	
		P:establishment of cell polarity (4.51E-05)	
		P:response to pheromone during conjugation with cellular fusion (0.000182855)	
		P:telomere maintenance via recombination (0.000540669)	
		P:SRP-dependent cotranslational protein targeting to membrane, translocation (0.00246)	
NRG1 (0.1005)	cc	P:ferric iron transport (0.001490757)	
		P:fatty acid transport (0.005951036)	
		P:propionate metabolism (0.007433806)	
		P:siderophore-iron transport (0.008914585)	
	hs	P:energy derivation by oxidation of organic compounds (9.58E-08)	
		P:response to oxidative stress (0.000872563)	[118]
		P:aerobic respiration (0.002608658)	
		P:trehalose metabolism (0.003347525)	
		P:glycogen catabolism (0.003347525)	
	nd	P:hexose metabolism (6.11E-05)	
		P:biological process unknown (0.001461617)	
		P:succinate transport (0.008199165)	
PDR1 (0.1708)	cc	P:telomere maintenance via recombination (2.91E-13)	
		P:protein biosynthesis (0.000870377)	
	hs	P:carbohydrate metabolism (2.31E-05)	
		P:response to stress (8.06E-05)	
		P:amino acid catabolism (0.000271653)	
		P:glycogen metabolism (0.000322667)	
		P:arginine catabolism (0.000451339)	
		P:carboxylic acid metabolism (0.001971697)	
		P:biological process unknown (0.00345017)	
		P:hexose transport (0.009522326)	[119]
	nd	P:telomere maintenance via recombination (1.71E-05)	
		P:protein biosynthesis (0.001612308)	
		P:glutamine family amino acid biosynthesis (0.003887589)	
		P:arginine biosynthesis (0.005940799)	
	cc	P:steroid biosynthesis (0.000129082)	
		P:amino acid biosynthesis (0.000135962)	
		P:generation of precursor metabolites and energy (1.80E-10)	
		P:carbohydrate metabolism (2.68E-07)	
		P:protein refolding (3.03E-05)	
		P:response to stress (0.000123194)	

PUT3 (0.1227)	hs	P:glycogen metabolism (0.00022772)	
		P:trehalose metabolism (0.000964509)	
		P:glutamine family amino acid biosynthesis (0.001318333)	
		P:aerobic respiration (0.002061356)	
		P:alcohol biosynthesis (0.00250657)	
		P:ATP synthesis coupled electron transport (0.003674061)	
		P:proteasome assembly (0.009164113)	
		P:pentose-phosphate shunt, oxidative branch (0.009164113)	
	nd	P:transition metal ion transport (0.001701555)	
		P:response to temperature stimulus (0.002214734)	
		P:hexose metabolism (0.004303704)	
		P:siderophore transport (0.005392145)	
		P:response to stimulus (0.006540635)	
		P:carbohydrate transport (0.007472337)	
RAP1 (0.5317)	cc	P:protein biosynthesis (6.09E-66)	
		P:telomere maintenance via recombination (4.33E-08)	
		P:protein complex assembly (6.56E-07)	
		P:ribosome biogenesis and assembly (1.76E-06)	[112]
		P:glycolysis (0.000261521)	
		P:alcohol metabolism (0.004184356)	
		P:ergosterol biosynthesis (0.007400393)	
	hs	P:protein biosynthesis (1.01E-76)	
		P:ribosome biogenesis and assembly (4.69E-15)	
		P:protein complex assembly (1.11E-07)	
		P:telomere organization and biogenesis (5.75E-06)	
		P:amino acid metabolism (5.20E-05)	
	nd	P:protein biosynthesis (1.91E-97)	
		P:ribosome biogenesis and assembly (3.05E-10)	
		P:telomere organization and biogenesis (1.85E-09)	
		P:protein complex assembly (2.45E-08)	
		P:telomere maintenance via recombination (1.59E-05)	
RDS1 (0.0423)	cc	P:telomere maintenance via recombination (1.17E-08)	
		P:ergosterol biosynthesis (1.62E-05)	
		P:alcohol metabolism (0.00392493)	
	hs	P:carbohydrate metabolism (0.000273627)	
		P:glycogen metabolism (0.001817391)	
		P:regulation of gluconeogenesis (0.0037147)	
		F:UTP:glucose-1-phosphate uridylyltransferase activity (0.008497317)	
		P:coenzyme A transport (0.008497317)	
	nd	P:telomere maintenance via recombination (1.04E-06)	
		P:isoprenoid metabolism (0.000512299)	
		P:biological process unknown (0.006276664)	
		P:ergosterol biosynthesis (0.0095383)	
REB1 (0.0858)	cc	P:ubiquitin-dependent protein catabolism (3.16E-05)	
		P:proteolysis (9.34E-05)	
		P:electron transport (0.002948648)	
		P:glucan metabolism (0.005560803)	
	hs	P:ribosome biogenesis and assembly (1.64E-07)	
		P:transcription from RNA polymerase I promoter (0.001443953)	[112]
		P:tRNA aminoacylation (0.000143059)	
		P:protein biosynthesis (0.001585267)	
		P:posttranslational protein targeting to membrane (0.007755173)	
		P:ion transport (0.009431997)	
		P:cellular localization (1.95E-05)	

	nd	P:tRNA aminoacylation (0.000425856)	
		P:transport (0.000958107)	
		P:amino acid metabolism (0.001600864)	
		P:posttranslational protein targeting to membrane (0.003846905)	
		P:cell wall organization and biogenesis (0.002646024)	
		P:protein targeting (0.006972959)	
		P:establishment of cell polarity (0.008539257)	
RPN4 (0.0470)	cc	P:glycolysis (2.91E-05)	
		P:ubiquitin-dependent protein catabolism (0.000781782)	
		P:proteolysis (0.001813857)	[112]
		P:energy derivation by oxidation of organic compounds (0.00241272)	
	hs	P:protein folding (2.55E-12)	
		P:response to stress (6.31E-08)	
		P:protein catabolism (8.50E-06)	
		P:SRP-dependent cotranslational protein targeting to membrane, translocation (3.87E-05)	
		P:cell redox homeostasis (5.98E-05)	
		P:generation of precursor metabolites and energy (7.51E-05)	
		P:proteolysis (0.000318121)	[112]
		P:ubiquitin-dependent protein catabolism (0.002759552)	
		P:glycogen metabolism (0.003186606)	
		P:propionate metabolism (0.004386166)	
	nd	P:proteolysis (6.23E-15)	[112]
		P:ubiquitin-dependent protein catabolism (7.05E-11)	
		P:protein modification (1.14E-07)	
		P:cell aging (0.00708813)	
SFP1 (0.5972)	cc	P:protein biosynthesis (1.87E-66)	
		P:ribosome biogenesis and assembly (1.05E-06)	[120]
		P:telomere maintenance via recombination (1.53E-06)	
		P:pyruvate metabolism (0.000218871)	
		P:alcohol biosynthesis (0.001288423)	
	hs	P:protein biosynthesis (1.82E-80)	
		P:ribosome biogenesis and assembly (7.11E-15)	
		P:telomere organization and biogenesis (1.02E-06)	
		P:agglutination (0.006652765)	
	nd	P:protein biosynthesis (3.10E-122)	
		P:ribosome biogenesis and assembly (7.40E-13)	
		P:telomere organization and biogenesis (1.71E-07)	
		P:negative regulation of protein biosynthesis (0.005392791)	
SKN7 (0.2665)	cc	P:protein biosynthesis (1.95E-13)	
		P:telomere maintenance via recombination (7.37E-10)	
		P:ribosome biogenesis and assembly (0.001499504)	
	hs	P:protein biosynthesis (3.68E-47)	
		P:ribosomal subunit assembly (2.30E-09)	
		P:generation of precursor metabolites and energy (3.58E-09)	
		P:glycogen metabolism (6.33E-05)	
		P:response to oxidative stress (0.000118541)	[112]
		P:trehalose biosynthesis (0.00048412)	
		P:tricarboxylic acid cycle (0.000614238)	
		P:pentose-phosphate shunt (0.001558242)	
		P:cellular respiration (0.001647824)	
		P:telomere organization and biogenesis (0.003656976)	
		P:protein refolding (0.003780974)	
		P:NADPH regeneration (0.004192185)	
		P:protein biosynthesis (9.58E-48)	

	nd	P:ribosomal subunit assembly (1.47E-11)	
		P:telomere organization and biogenesis (0.000249483)	
		P:energy reserve metabolism (0.000676495)	
		P:response to oxidative stress (0.00579379)	
STB1 (0.3129)	cc	P:DNA replication (2.42E-13)	
		P:mitotic cell cycle (5.60E-10)	[112]
		P:cell cycle checkpoint (6.46E-05)	
		P:response to stress (0.002699099)	
		P:cell budding (0.005011804)	
		P:telomere organization and biogenesis (0.006902975)	
	hs	P:regulation of cyclin-dependent protein kinase activity (4.40E-06)	
		P:DNA replication (0.000297966)	
		P:mitotic cell cycle (0.001914146)	
		P:cell wall organization and biogenesis (0.003162033)	
		P:establishment of cell polarity (0.00411929)	
	nd	P:cell wall organization and biogenesis (1.51E-09)	
		P:mitotic cell cycle (3.46E-09)	
		P:regulation of cyclin-dependent protein kinase activity (1.68E-08)	
		P:cytokinesis (1.10E-07)	
		P:sexual reproduction (4.32E-07)	
		P:cell budding (0.000199807)	
		P:establishment of cell polarity (0.000289061)	
		P:sterol metabolism (0.00275283)	
STE12 (0.1991)	hs	P:sexual reproduction (8.22E-05)	[112]
		P:filamentous growth (0.002316712)	
		P:regulation of cell size (0.004901259)	
		P:cell cycle arrest (0.004952447)	
		P:vitamin B6 biosynthesis (0.009838998)	
	nd	P:sexual reproduction (9.13E-13)	
		P:mitotic cell cycle (2.34E-09)	
		P:cell wall organization and biogenesis (2.21E-05)	
		P:mitochondrion localization (8.70E-05)	
		P:cell budding (0.000193738)	
		P:DNA replication (0.000633302)	
		P:establishment of cell polarity (0.001411096)	
		P:vacuole organization and biogenesis (0.008961569)	
SUT1 (0.1403)	cc	P:protein biosynthesis (3.33E-11)	
		P:ATP synthesis coupled electron transport (0.00022481)	
		P:ribosome biogenesis and assembly (0.000449651)	
		P:generation of precursor metabolites and energy (0.001604264)	
	hs	P:generation of precursor metabolites and energy (4.63E-16)	
		P:carbohydrate metabolism (1.78E-12)	
		P:response to stress (2.77E-07)	
		P:aerobic respiration (6.35E-07)	
		P:glycogen metabolism (2.79E-06)	
		P:tricarboxylic acid cycle (4.54E-06)	
		P:propionate metabolism (0.000331445)	
		P:protein refolding (0.001104638)	
		P:alcohol biosynthesis (0.003068428)	
		P:pentose-phosphate shunt (0.004724122)	
		P:glutamate biosynthesis (0.007800988)	
		P:NADPH regeneration (0.009691356)	
		P:protein biosynthesis (5.73E-17)	
		P:ribosomal subunit assembly (5.54E-06)	

	nd	P:ergosterol biosynthesis (0.003464819)	[121]
		P:generation of precursor metabolites and energy (0.004462221)	
		P:regulation of glycolysis (0.009161704)	
SWI4 (0.2925)	cc	P:DNA replication (9.32E-12)	
		P:telomere maintenance via recombination (4.35E-11)	
		P:cell cycle (1.60E-10)	[112]
		P:DNA repair (7.67E-06)	
	hs	P:sexual reproduction (0.000807385)	
		P:cell wall organization and biogenesis (0.00130753)	
		P:cytokinesis, site selection (0.002269138)	
		P:cell budding (0.00763255)	
		P:establishment of cell polarity (0.008739271)	
		P:regulation of cyclin-dependent protein kinase activity (0.006185118)	
	nd	P:mitotic cell cycle (1.21E-08)	
		P:cell wall organization and biogenesis (8.19E-07)	
		P:amino acid metabolism (2.57E-06)	
		P:cytokinesis (3.24E-06)	
		P:cell budding (1.43E-05)	
		P:establishment of cell polarity (3.97E-05)	
SWI5 (0.0439)	cc	P:telomere maintenance via recombination (4.97E-15)	
	hs	P:cell wall organization and biogenesis (0.000634125)	
		P:pentose-phosphate shunt, oxidative branch (0.005950593)	
	nd	P:cytokinesis, completion of separation (1.08E-05)	[112]
		P:telomere maintenance via recombination (0.00584125)	
SWI6 (0.3019)	cc	P:DNA replication (2.41E-11)	
		P:telomere maintenance via recombination (7.03E-11)	
		P:cell cycle (9.98E-11)	[112]
		P:cell cycle checkpoint (2.42E-05)	
		P:response to stress (0.003338005)	
	hs	P:regulation of cyclin-dependent protein kinase activity (4.66E-05)	
		P:DNA replication (0.000189903)	
		P:sexual reproduction (0.000545235)	
		P:cell wall organization and biogenesis (0.001201043)	
		P:protein biosynthesis (0.002534844)	
		P:cell budding (0.004054469)	
		P:establishment of cell polarity (0.005596326)	
		P:filamentous growth (0.009777862)	
	nd	P:regulation of cyclin-dependent protein kinase activity (7.98E-09)	
		P:cell wall organization and biogenesis (5.30E-08)	
		P:sexual reproduction (9.14E-06)	
		P:cell budding (4.31E-05)	
		P:amino acid metabolism (4.66E-05)	
		P:establishment of cell polarity (0.000123182)	
UGA3 (0.0295)	cc	P:protein biosynthesis (5.93E-17)	
		P:ribosome biogenesis and assembly (6.23E-05)	
	hs	P:generation of precursor metabolites and energy (6.13E-15)	
		P:carbohydrate metabolism (1.24E-08)	
		P:glycogen metabolism (9.35E-06)	
		P:pentose-phosphate shunt, oxidative branch (5.31E-05)	
		P:response to stress (0.000148758)	
		P:protein refolding (0.000180899)	
		P:ATP synthesis coupled electron transport (0.000446187)	
		P:NADPH regeneration (0.001717829)	
		P:branched chain family amino acid biosynthesis (1.24E-14)	

	nd	P:telomere maintenance via recombination (2.06E-05)	
		P:regulation of glycolysis (0.000231732)	
		P:sterol biosynthesis (0.000242527)	
		P:alcohol metabolism (0.000371794)	
		P:generation of precursor metabolites and energy (0.00168821)	
YAP5 (0.2047)	cc	P:telomere maintenance via recombination (1.17E-08)	no annotation
		P:protein biosynthesis (0.000254815)	
	hs	P:biological process unknown (0.000915919)	
	nd	P:telomere maintenance via recombination (2.74E-05)	
		P:aldehyde metabolism (0.000124576)	
		P:protein biosynthesis (0.004963387)	
		P:biological process unknown (0.006310324)	
confirmed in cc and nd			
DIG1 (0.0354)	cc	P:cell size control checkpoint (0.000894454)	
		P:cell cycle (0.003945446)	
		P:meiotic cell cycle (0.006054798)	
	nd	P:sexual reproduction (1.81E-13)	[112]
		P:cell wall organization and biogenesis (1.13E-05)	
		P:mitotic cell cycle (1.43E-05)	
		P:budding cell bud growth (0.00037212)	
		P:inositol lipid-mediated signaling (0.002911757)	
		P:establishment and/or maintenance of cell polarity (sensu Fungi) (0.005447692)	
		P:response to abiotic stimulus (0.005541891)	
		P:SRP-dependent cotranslational protein targeting to membrane (0.005852328)	
FKH1 (0.0409)	cc	P:mitotic cell cycle (2.26E-05)	[112]
		P:cytokinesis (0.00010763)	
		P:establishment of cell polarity (0.002563068)	
	nd	P:mitotic cell cycle (1.88E-08)	
		P:cytokinesis (2.29E-06)	
		P:sexual reproduction (4.09E-05)	
		P:cell wall organization and biogenesis (4.43E-05)	
GCR1 (0.0409)	cc	P:glycolysis (2.22E-08)	[112]
		P:alcohol metabolism (4.15E-07)	
		P:energy derivation by oxidation of organic compounds (1.43E-06)	
		P:rRNA modification (5.82E-05)	
		P:regulation of cell redox homeostasis (0.001107773)	
		P:fermentation (0.002692313)	
		P:35S primary transcript processing (0.004641205)	
		P:ergosterol biosynthesis (0.005803953)	
	nd	P:transposition, RNA-mediated (5.53E-10)	
		P:glycolysis (1.37E-09)	
		P:alcohol catabolism (2.28E-07)	
		P:energy derivation by oxidation of organic compounds (1.17E-06)	
		P:glutamine family amino acid metabolism (0.001410872)	
		P:cell cycle (0.002262634)	
GCR2 (0.0821)	cc	P:glycolysis (1.87E-08)	[112]
		P:alcohol metabolism (6.76E-08)	
		P:fermentation (0.00032808)	
	nd	P:ribosomal small subunit export from nucleus (0.004465608)	
		P:ribosomal small subunit biogenesis (0.007136987)	
GLN3 (0)	cc	P:glutamine biosynthesis (0.00104353)	[122]
		P:amino acid biosynthesis (0.004663081)	
	nd	P:thiamin biosynthesis (0.000687268)	
		P:cell redox homeostasis (0.004521275)	

		P:biological process unknown (0.00999131)	
PHO4 (0)	cc	P:alcohol metabolism (0.000892152)	
		P:ergosterol biosynthesis (0.002193558)	
		P:amino acid biosynthesis (0.002849144)	
		P:pyruvate metabolism (0.004521797)	
		P:ribosomal large subunit assembly and maintenance (0.005560803)	
	nd	P:amino acid metabolism (4.06E-15)	
		P:cellular response to nitrogen starvation (1.25E-08)	
		P:nucleotide biosynthesis (0.006996571)	
		P:glycolysis (0.00913288)	
UME1 (0.0629)	cc	P:telomere maintenance via recombination (4.36E-13)	
		P:ergosterol biosynthesis (3.60E-05)	
		P:alcohol metabolism (0.008208477)	
	nd	P:telomere maintenance via recombination (1.47E-07)	
confirmed in hs and nd			
ACE2 (0.0231)	hs	P:generation of precursor metabolites and energy (2.32E-05)	
		P:trehalose metabolism (0.000158289)	
		P:response to stress (0.005467194)	
		P:response to methylglyoxal (0.008474531)	
	nd	P:cytokinesis, completion of separation (6.55E-08)	[89]
		P:cell wall organization and biogenesis (0.001774695)	
AFT2 (0.0662)	hs	P:iron ion homeostasis (1.75E-05)	[112]
		P:siderophore transport (0.004220706)	
		P:iron-sulfur cluster assembly (0.005237811)	
		P:regulation of gluconeogenesis (0.00635561)	
	nd	P:protein biosynthesis (4.75E-25)	
		P:ribosomal subunit assembly (1.54E-07)	
		P:pyridoxine metabolism (0.000554223)	
		P:telomere organization and biogenesis (0.002255812)	
		P:iron ion homeostasis (0.00408746)	
		P:thiamin metabolism (0.009279171)	
AZF1 (0.1234)	hs	P:cytokinesis, site selection (7.30E-05)	
		P:amino acid metabolism (0.000614107)	
		P:establishment of cell polarity (0.001289685)	
		P:cell budding (0.001789324)	
		P:pyrimidine salvage (0.002398001)	
		P:cell cycle checkpoint (0.00832863)	
	nd	P:cell wall organization and biogenesis (1.12E-05)	[123]
		P:cell cycle (0.000305513)	
		P:reproduction (0.000697229)	
		P:axial bud site selection (0.001021724)	
		P:tRNA aminoacylation (0.002555682)	
		P:cell budding (0.006759657)	
BAS1 (0)	hs	P:energy derivation by oxidation of organic compounds (1.12E-05)	
		P:response to stress (2.45E-05)	
		P:pentose-phosphate shunt, oxidative branch (0.000342826)	
		P:response to reactive oxygen species (0.000714512)	
		P:alcohol metabolism (0.002364807)	
		P:NADPH regeneration (0.0030155)	
		P:ubiquitin-dependent protein catabolism (0.008830063)	
	nd	P:amino acid metabolism (1.87E-53)	
		P:purine base metabolism (1.47E-09)	[112]
		P:tricarboxylic acid cycle intermediate metabolism (0.002194587)	
		P:iron-sulfur cluster assembly (0.008126373)	

GAL4 (0.0681)	hs	P:generation of precursor metabolites and energy (6.80E-09)	
		P:carbohydrate metabolism (1.90E-06)	[112], [124]
		P:ATP synthesis coupled electron transport (0.000418275)	
		P:glycogen metabolism (0.00149061)	
		P:aerobic respiration (0.001746665)	
	nd	P:cellular response to nitrogen starvation (2.21E-07)	
		P:asparagine catabolism (2.21E-07)	
		P:glutamine family amino acid catabolism (2.88E-05)	
		P:biological process unknown (0.00049482)	
		P:alcohol metabolism (0.000526586)	
		P:cellular carbohydrate metabolism (0.00161056)	
GAL80 (0.0605)	hs	P:generation of precursor metabolites and energy (1.02E-10)	
		P:carbohydrate metabolism (8.15E-07)	[112], [124]
		P:energy reserve metabolism (6.76E-05)	
		P:ATP synthesis coupled electron transport (0.000105976)	
		P:pentose-phosphate shunt (0.000270708)	
		P:NADPH regeneration (0.000581574)	
		P:response to stress (0.00173538)	
		P:response to reactive oxygen species (0.002979127)	
		P:amino acid catabolism (0.004627473)	
	nd	P:proline catabolism (0.000176404)	
		P:glycogen catabolism (0.000610996)	
		P:glutamine family amino acid catabolism (0.00222256)	
		P:carbohydrate catabolism (0.00299385)	
		P:chaperone cofactor-dependent protein folding (0.005515802)	
		P:generation of precursor metabolites and energy (0.006971599)	
LEU3 (0.0307)	hs	P:generation of precursor metabolites and energy (7.55E-14)	
		P:branched chain family amino acid biosynthesis (7.59E-09)	[112]
		P:carbohydrate metabolism (9.76E-08)	
		P:energy reserve metabolism (1.49E-07)	
		P:ATP synthesis coupled electron transport (8.37E-06)	
		P:aerobic respiration (1.20E-05)	
		P:iron-sulfur cluster assembly (0.001571388)	
		P:response to stress (0.00384227)	
		P:propionate metabolism (0.005795568)	
	nd	P:amino acid metabolism (2.54E-23)	
		P:purine nucleotide biosynthesis (0.00790666)	
		P:regulation of cyclin-dependent protein kinase activity (0.009258796)	
MAT1mc (0.2310)	hs	P:biological process unknown (8.97E-05)	
	nd	P:biological process unknown (5.95E-06)	
MSN2 (0.1380)	hs	P:generation of precursor metabolites and energy (1.85E-15)	
		P:carbohydrate metabolism (4.78E-12)	
		P:response to stress (1.60E-10)	[112]
		P:protein folding (4.99E-10)	
		P:glycogen metabolism (8.74E-07)	
		P:response to oxidative stress (4.90E-06)	
		P:ATP synthesis coupled electron transport (5.26E-05)	
		P:SRP-dependent cotranslational protein targeting to membrane, translocation (6.82E-05)	
		P:chronological cell aging (0.000306581)	
		P:NADPH regeneration (0.000457109)	
		P:pentose-phosphate shunt, oxidative branch (0.001216579)	
		P:biological process unknown (0.002143235)	
		P:SRP-dependent cotranslational protein targeting to membrane (0.0021496)	
		P:trehalose metabolism (0.003943189)	

		P:tricarboxylic acid cycle (0.005789132)	
	nd	P:response to stress (1.19E-06)	
		P:carbohydrate metabolism (6.72E-06)	
		P:biological process unknown (0.000195196)	
		P:protein folding (0.00047966)	
		P:glycogen metabolism (0.000633063)	
		P:generation of precursor metabolites and energy (0.001797435)	
		P:response to oxidative stress (0.005932035)	
		P:carbohydrate transport (0.00651661)	
		P:alcohol metabolism (0.007075006)	
PDR3 (0.0424)	hs	P:generation of precursor metabolites and energy (8.12E-09)	
		P:carbohydrate metabolism (3.61E-08)	
		P:response to stress (8.87E-07)	[125]
		P:energy reserve metabolism (2.60E-06)	
		P:amino acid catabolism (6.14E-05)	
		P:response to oxidative stress (0.000101947)	
		P:aerobic respiration (0.000565841)	
		P:tricarboxylic acid cycle (0.006006125)	
		P:propionate metabolism (0.006212145)	
		P:amine transport (0.006715592)	
	nd	P:telomere maintenance via recombination (1.80E-05)	
		P:biological process unknown (2.59E-05)	
		P:glycogen catabolism (0.002907916)	
PHD1 (0.0433)	hs	P:generation of precursor metabolites and energy (2.09E-06)	
		P:protein refolding (8.96E-05)	
		P:cellular carbohydrate metabolism (0.000252824)	
		P:cofactor metabolism (0.004556098)	
		P:siderophore transport (0.006560661)	
		P:amino acid catabolism (0.006774133)	
		P:glycogen metabolism (0.007479989)	
		P:regulation of gluconeogenesis (0.009842158)	
	nd	P:hexose transport (0.000500041)	
		P:flocculation via cell wall protein-carbohydrate interaction (0.001971962)	[112]
		P:glycerophosphodiester transport (0.007602862)	
		P:coenzyme A transport (0.007602862)	
PHO2 (0.0236)	hs	P:protein biosynthesis (4.74E-55)	
		P:ribosomal subunit assembly (3.88E-11)	
		P:generation of precursor metabolites and energy (5.42E-06)	
		P:ATP synthesis coupled electron transport (2.26E-05)	
		P:telomere organization and biogenesis (0.000760425)	
		P:energy reserve metabolism (0.000908864)	
		P:glycogen metabolism (0.008484564)	
	nd	P:iron ion homeostasis (0.00015337)	
		P:glycogen catabolism (0.00025485)	
		P:siderophore transport (0.000434978)	
		P:generation of precursor metabolites and energy (0.000963692)	
		P:gluconeogenesis (0.003783755)	
		P:glycogen metabolism (0.004695964)	
RCS1 (0.0287)	hs	P:ribosome biogenesis and assembly (2.80E-05)	
		P:peptidyl-amino acid modification (0.008522178)	
	nd	P:protein polyubiquitination (0.000252408)	
		P:biological process unknown (0.005561098)	
		P:N-terminal protein amino acid modification (0.008588199)	
		P:generation of precursor metabolites and energy (0.000101233)	

RGT1 (0.0130)	hs	P:ATP synthesis coupled electron transport (0.001318218)	
		P:cellular carbohydrate metabolism (0.003346991)	
		P:glycogen metabolism (0.003408846)	
		P:response to stress (0.004612087)	
		P:chronological cell aging (0.007990463)	
	nd	P:response to arsenic (0.000437948)	
		P:glucose metabolism (0.005107811)	
		P:fumarate transport (0.005515802)	[112]
		P:succinate transport (0.005515802)	[112]
		P:gluconeogenesis (0.008852659)	
RLM1 (0.0420)	hs	P:response to stress (1.27E-09)	
		P:protein folding (1.03E-07)	
		P:SRP-dependent cotranslational protein targeting to membrane, translocation (3.62E-06)	
		P:cotranslational protein targeting to membrane (4.53E-05)	
		P:chaperone cofactor-dependent protein folding (0.003279666)	
		P:mitochondrial genome maintenance (0.004507712)	
		P:protein targeting (0.006432784)	
		P:protein transport (0.009169133)	
	nd	P:sexual reproduction (4.64E-12)	
		P:cell wall organization and biogenesis (6.20E-07)	[126]
		P:transposition, RNA-mediated (1.31E-05)	
		P:agglutination (1.39E-05)	
		P:cell projection biogenesis (0.006155377)	
		P:cellular carbohydrate metabolism (0.007570576)	
RLR1 (0.0317)	hs	P:fatty acid desaturation (0.00313059)	
		P:iron ion homeostasis (0.003851818)	
		P:protein amino acid N-linked glycosylation (0.008522178)	
	nd	P:cell wall organization and biogenesis (9.60E-05)	
		P:sexual reproduction (0.000307954)	
		P:mitotic cell cycle (0.000649398)	
		P:M phase of mitotic cell cycle (0.006286435)	
		P:G1/S transition of mitotic cell cycle (0.008892814)	
ROX1 (0.0325)	hs	P:carbohydrate metabolism (0.001107774)	
		P:lysosome organization and biogenesis (0.004025045)	
		P:gluconeogenesis (0.004777738)	
		P:glycogen metabolism (0.005924332)	
		P:energy derivation by oxidation of organic compounds (0.006210891)	
		P:energy reserve metabolism (0.009035148)	
	nd	P:regulation of carbohydrate biosynthesis (0.003424547)	
		P:arsenite transport (0.005217651)	
		P:transcription from RNA polymerase I promoter (0.00983042)	
RPH1 (0.0570)	hs	P:carbohydrate metabolism (2.78E-07)	
		P:energy derivation by oxidation of organic compounds (7.89E-06)	
		P:energy reserve metabolism (1.87E-05)	
		P:generation of precursor metabolites and energy (3.53E-05)	
		P:glycogen metabolism (0.000140722)	
		P:age-dependent response to oxidative stress during chronological cell aging (0.001768639)	
		P:pentose-phosphate shunt (0.004521275)	
		P:NADPH regeneration (0.007346268)	
		P:coenzyme A transport (0.009391771)	
	nd	P:response to stress (0.005636976)	
		P:chaperone cofactor-dependent protein folding (0.006559332)	
		P:coenzyme A transport (0.006559332)	
		P:carbohydrate metabolism (0.00040844)	

SOK2 (0.0930)	hs	P:generation of precursor metabolites and energy (0.000630206)	
		P:glycogen metabolism (0.003951403)	
		P:energy reserve metabolism (0.006047012)	
	nd	P:hexose transport (0.001891316)	
		P:energy derivation by oxidation of organic compounds (0.00241272)	
		P:carbohydrate metabolism (0.003193597)	
		P:glycogen metabolism (0.00360181)	
STP1 (0.0952)	hs	P:generation of precursor metabolites and energy (1.25E-11)	
		P:carbohydrate metabolism (9.66E-09)	
		P:energy reserve metabolism (3.21E-05)	
		P:cofactor catabolism (7.42E-05)	
		P:regulation of gluconeogenesis (0.000127985)	
		P:response to stress (0.0002433)	
		P:tricarboxylic acid cycle (0.000487749)	
		P:protein refolding (0.000565496)	
		P:response to oxidative stress (0.00071388)	
		P:glycogen metabolism (0.000815018)	
		P:tricarboxylic acid cycle intermediate metabolism (0.001276382)	
		P:vacuolar protein catabolism (0.001829724)	
		P:sporulation (0.002347208)	
		P:propionate metabolism (0.006425556)	
		P:iron ion homeostasis (0.00721943)	
		P:glutamate metabolism (0.007609596)	
		P:alcohol biosynthesis (0.008123352)	
	nd	P:biological process unknown (5.89E-05)	
		P:telomere maintenance via recombination (0.000365075)	
		P:regulation of gluconeogenesis (0.002467876)	
		P:alcohol metabolism (0.002834335)	
		P:generation of precursor metabolites and energy (0.005162845)	
		P:response to stress (0.006955772)	
		P:iron ion homeostasis (0.00721943)	
		P:response to arsenic (0.009473606)	
		P:microautophagy (0.009473606)	
TEC1 (0.1893)	hs	P:response to pheromone (5.82E-07)	
		P:regulation of cell size (9.54E-05)	
		P:filamentous growth (0.003607995)	
	nd	P:response to pheromone (1.28E-07)	
		P:telomere maintenance via recombination (3.54E-06)	
		P:filamentous growth (0.006952671)	[112]
		P:cell wall organization and biogenesis (0.008061579)	
		P:establishment of cell polarity (0.00984166)	
UME6 (0.0982)	hs	P:response to stress (1.83E-11)	
		P:protein folding (5.14E-09)	
		P:generation of precursor metabolites and energy (1.19E-08)	
		P:carbohydrate metabolism (2.98E-06)	
		P:response to oxidative stress (8.27E-06)	
		P:energy reserve metabolism (9.61E-06)	
		P:SRP-dependent cotranslational protein targeting to membrane, translocation (0.000153939)	
		P:glycogen metabolism (0.000199672)	
		P:regulation of gluconeogenesis (0.000236134)	
		P:protein catabolism (0.000406214)	
		P:alcohol metabolism (0.003323295)	
		P:chronological cell aging (0.006412976)	
		P:proteasome assembly (0.008745136)	

		P:amino acid catabolism (0.009754466)	
	nd	P:response to stress (0.000200674)	
		P:biological process unknown (0.000635801)	
		P:positive regulation of gluconeogenesis (0.001032217)	
		P:gluconeogenesis (0.0012503)	
		P:multidrug transport (0.001635714)	
		P:alcohol metabolism (0.001766734)	
		P:generation of precursor metabolites and energy (0.002047936)	
		P:oxygen and reactive oxygen species metabolism (0.002948857)	
		P:hexose transport (0.004065415)	
		P:fatty acid transport (0.00593292)	
YAP1 (0)	hs	P:response to oxidative stress (7.05E-05)	[112]
	nd	P:response to inorganic substance (1.44E-05)	
YAP6 (0.0527)	hs	P:ribosome biogenesis and assembly (0.003612095)	[127]
		P:low affinity iron ion transport (0.008199165)	
	nd	P:aldehyde metabolism (0.000114126)	
		P:flocculation via cell wall protein-carbohydrate interaction (0.000173115)	
		P:response to inorganic substance (0.002445818)	[128]
confirmed only in nd			
ARR1	nd	P:response to inorganic substance (0.00014869)	
		P:arsenite transport (0.005068575)	[112]
		P:response to chemical stimulus (0.006578537)	
ASH1	nd	P:re-entry into mitotic cell cycle (0.002234803)	
		P:cellular carbohydrate metabolism (0.007237093)	
CAD1	nd	P:aldehyde metabolism (0.000187543)	
		P:response to inorganic substance (0.000229033)	
		P:biological process unknown (0.000615773)	
		P:response to drug (0.002326584)	[112]
		P:mitochondrial protein processing (0.003174142)	
		P:glutathione metabolism (0.005810755)	
CBF1	nd	P:amino acid metabolism (1.22E-19)	[112]
		P:regulation of mRNA stability (0.001806087)	
		P:nucleotide biosynthesis (0.002202035)	
DAL80	nd	P:base-excision repair, AP site formation (0.005803557)	
		P:1,6-beta-glucan metabolism (0.009655305)	
DAL82	nd	P:telomere maintenance via recombination (7.54E-07)	
		P:biological process unknown (1.46E-05)	
DAT1	nd	P:telomere maintenance via recombination (8.13E-12)	no annotation
GAT1	nd	P:aspartate family amino acid catabolism (7.83E-08)	
		P:cellular response to nitrogen starvation (1.31E-07)	[112]
		P:glutamine family amino acid catabolism (3.88E-07)	
		P:telomere maintenance via recombination (2.39E-05)	
		P:biological process unknown (5.79E-05)	
		P:thiamin biosynthesis (0.001757254)	
GZF3	nd	P:telomere maintenance via recombination (1.99E-07)	
		P:biological process unknown (0.000865976)	
		P:thiamin biosynthesis (0.000935569)	
INO4	nd	P:membrane lipid biosynthesis (1.58E-06)	[112]
		P:phosphatidylethanolamine biosynthesis (8.40E-05)	
		P:glycerophospholipid biosynthesis (0.000127891)	
		P:alcohol metabolism (0.001094776)	
		P:amino acid derivative biosynthesis (0.001714422)	
		P:sulfur amino acid metabolism (2.22E-15)	[112]

MET32	nd	P:serine family amino acid metabolism (1.42E-05)	
		P:histidine metabolism (0.003478561)	
		P:nucleotide biosynthesis (0.004848226)	
MET4	nd	P:sulfur amino acid metabolism (4.17E-13)	[112]
		P:serine family amino acid metabolism (2.82E-06)	
		P:purine nucleotide biosynthesis (0.00059808)	
		P:histidine metabolism (0.001590326)	
MOT3	nd	P:amine transport (0.000986794)	
		P:nicotinamide riboside metabolism (0.00104353)	
		P:urea transport (0.002086127)	
		P:mannose metabolism (0.00312779)	
		P:fructose metabolism (0.006247192)	
RIM101	nd	P:hexose transport (0.000830468)	
RME1	nd	P:protein biosynthesis (1.10E-05)	
RTG3	nd	P:cotranslational protein targeting to membrane (0.004798705)	
		P:glutamine family amino acid biosynthesis (0.007184084)	[129]
SIG1	nd	P:transposition, RNA-mediated (3.48E-08)	
		P:pheromone-dependent signal transduction during conjugation with cellular fusion (0.000697515)	[130]
		P:posttranslational protein targeting to membrane (0.001601925)	
SIP4	nd	P:protein polyubiquitination (0.004474847)	
		P:cell wall organization and biogenesis (0.007583263)	
SMP1	nd	P:protein biosynthesis (2.82E-08)	
		P:ribosome biogenesis and assembly (6.31E-05)	
SPT2	nd	P:biological process unknown (0.007099743)	no annotation
STB4	nd	P:biological process unknown (0.000404341)	unknown
STB5	nd	P:transposition, RNA-mediated (9.56E-09)	
		P:pheromone-dependent signal transduction during conjugation with cellular fusion (0.005240133)	
SUM1	nd	P:cell wall organization and biogenesis (0.000894317)	
		P:transposition, RNA-mediated (0.001127791)	
THI2	nd	P:thiamin biosynthesis (4.52E-08)	[112]
		P:pyridoxine metabolism (0.00011246)	
TYE7	nd	P:sulfur amino acid metabolism (2.19E-15)	
		P:serine family amino acid metabolism (4.78E-07)	
		P:regulation of mRNA stability (0.003617958)	
XBP1	nd	P:reproduction (2.39E-07)	
		P:establishment of cell polarity (8.59E-07)	
		P:cell budding (1.41E-06)	
		P:ergosterol biosynthesis (8.44E-06)	
		P:response to pheromone during conjugation with cellular fusion (0.000335338)	
		P:cell wall organization and biogenesis (0.000388349)	
		P:vacuole inheritance (0.001006102)	
		P:cell cycle (0.002389602)	
		P:secretory pathway (0.002803363)	
		P:G1/S-specific transcription in mitotic cell cycle (0.00554338)	[131]
		P:mitochondrion inheritance (0.00728001)	
		P:telomere maintenance via recombination (0.008753987)	
YAP3	nd	P:response to inorganic substance (0.00014869)	no annotation
YAP7	nd	P:response to inorganic substance (6.02E-05)	
		P:pentose-phosphate shunt, oxidative branch (0.001082116)	
		P:mitochondrial protein processing (0.001612065)	
		P:glutathione metabolism (0.002968186)	
		P:oxygen and reactive oxygen species metabolism (0.003595947)	
		P:response to stress (0.004612087)	[112]

		P:coenzyme metabolism (0.007998573)	
		P:NADPH regeneration (0.009258796)	
YHP1	nd	P:glutamate biosynthesis (0.00064596)	
		P:tricarboxylic acid cycle intermediate metabolism (0.001401029)	
YOX1	nd	P:regulation of cell cycle (0.000405848)	[112]
		P:cell wall organization and biogenesis (0.000568016)	
		P:regulation of nucleobase, nucleoside, nucleotide and nucleic acid metabolism (0.005835575)	
MGA1	nd	none	
MAL33	nd	none	
confirmed only in hs			
ADR1	hs	P:generation of precursor metabolites and energy (3.21E-08)	
		P:ATP synthesis coupled electron transport (9.16E-05)	
		P:aerobic respiration (0.000215868)	
		P:carbohydrate metabolism (0.000564975)	
		P:protein refolding (0.002767974)	
		P:amino acid catabolism (0.004166724)	
		P:glycogen metabolism (0.004608455)	
		P:iron-sulfur cluster assembly (0.005796893)	
		P:glucose metabolism (0.006632696)	[132]
DAL81	hs	P:protein folding (1.66E-15)	
		P:response to stress (2.09E-08)	
		P:SRP-dependent cotranslational protein targeting to membrane, translocation (2.88E-06)	
		P:protein targeting (0.001106108)	
		P:proteasome assembly (0.001206951)	
		P:protein transport (0.002238281)	
HIR1	hs	P:chromatin assembly or disassembly (8.97E-10)	[112]
		P:DNA repair (0.005392718)	
HIR2	hs	P:chromatin assembly or disassembly (8.97E-10)	[112]
		P:DNA repair (0.005392718)	
HIR3	hs	P:chromatin assembly or disassembly (8.97E-10)	[112]
		P:DNA repair (0.005392718)	
INO2	hs	P:generation of precursor metabolites and energy (9.20E-07)	
		P:carbohydrate metabolism (2.58E-06)	
		P:iron ion homeostasis (0.00279855)	
		P:glucose metabolism (0.003119564)	
		P:vacuolar protein catabolism (0.003838672)	
MAC1	hs	P:copper ion import (0.00372245)	[112]
SKO1	hs	P:protein amino acid myristoylation (0.004465608)	
		P:cell wall organization and biogenesis (0.007580525)	[133]
SNT2	hs	P:ATP synthesis coupled electron transport (2.53E-08)	unknown
		P:energy reserve metabolism (2.21E-05)	
		P:aerobic respiration (0.000191786)	
		P:amino acid catabolism (0.001034743)	
		P:pentose-phosphate shunt, oxidative branch (0.002580187)	
		P:response to stress (0.003672458)	
		P:myo-inositol metabolism (0.00530317)	
STE11	hs	P:tRNA aminoacylation (0.001958268)	
		P:G-protein signaling, adenylate cyclase activity (0.004467604)	
confirmed only in cc			
HAP5	cc	P:ATP synthesis coupled electron transport (5.26E-07)	[112]
		P:generation of precursor metabolites and energy (0.000664279)	[112]
		P:iron-sulfur cluster assembly (0.008914585)	
YDR026c	cc	P:telomere maintenance via recombination (7.45E-09)	unknown
		P:generation of precursor metabolites and energy (0.006971599)	

		P:nitrogen compound metabolism (0.009615493)	
ZAP1	cc	P:zinc ion transport (0.008914585)	[112]

Additional data file 7. Enriched functional categories of all TFs

Functional enrichment analysis was performed for all the target genes of confirmed TFs in three conditions (heat shock; hs, cell cycle; cc, nitrogen depletion; nd). Yellow mark indicates that the TF is already annotated as the enriched functional cateogory in the Saccharomyces Genome Database [112]. Green mark represents that the TF has some additional evidence related to the enriched category, which can be found in papers or other web sites. Overall OLs are calculated by summing the OLs between the targets of each TF in different conditions.
