# Supplementary material for: High-resolution analysis of condition-specific regulatory modules in Saccharomyces cerevisiae
Source: Genome Biol. 2008 Jan 3;9(1):R2. doi: 10.1186/gb-2008-9-1-r2 (PMC2395236; doi:10.1186/gb-2008-9-1-r2)
Supplement: Additional data file 8 — Relative nucleosome occupancy levels on the promoters of condition-specific target genes of Uga3 and Yap1 [file gb-2008-9-1-r2-S8.pdf]

Additional data 8. Condition-specific nucleosome occupancy in the promoters of ‘condition-altered’ TF target genes

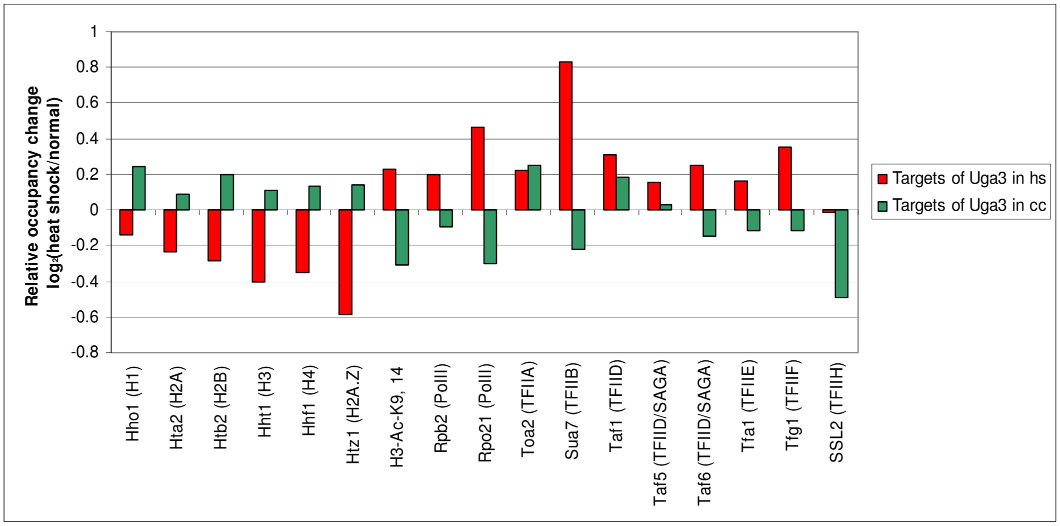

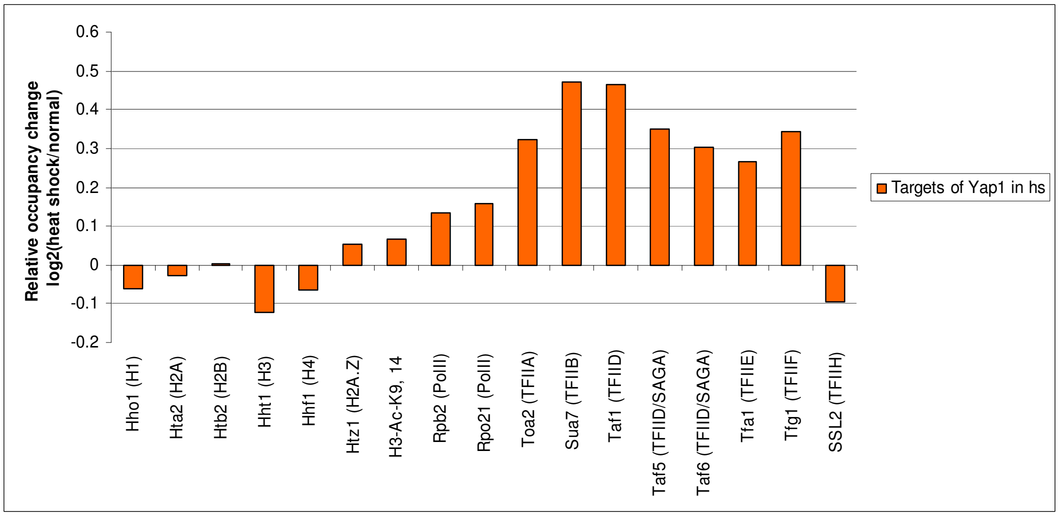

The graphs show the averaged log ratio of binding signal in the 37˚C sample over the signal in the 25˚C sample. Promoters of Uga3 in heat shock condition exhibited lower occupancy of all kinds of nucleosomes except acetylated H3, which is known to have a positive correlation with transcriptional activation. The higher occupancy of transcriptional machinery components implies that the promoters are more accessible to the transcriptional machinery in heat shock condition. In contrast, promoters of Uga3 in cell cycle condition showed largely opposite pattern. Though some transcriptional machinery components display higher occupancy in heat shock condition than normal condition, lower occupancy of RNA polymerase II indicated that less transcription might be occurred in heat shock condition.

Promoters of Yap1 target genes in heat shock condition also displayed nucleosome occupancy pattern similar to the pattern of Uga3 targets in heat shock condition. (Yap1 had no predicted target genes in cell cycle condition)
