# Supplementary material for: High-resolution analysis of condition-specific regulatory modules in Saccharomyces cerevisiae
Source: Genome Biol. 2008 Jan 3;9(1):R2. doi: 10.1186/gb-2008-9-1-r2 (PMC2395236; doi:10.1186/gb-2008-9-1-r2)
Supplement: Additional data file 11 — Matrices describing all EPMs and RMs, including lists of synergistic pairs of regulators. [file gb-2008-9-1-r2-S11.zip › htmls/upper_4.htm]

RMs (Regulator Set Modules)


**RMs (Regulator Set Modules)**

**--- condition ----
Heat shock
Nitrogen Depletion
Cell Cycle

--- EPMs ---**
