# Supplementary material for: High-resolution analysis of condition-specific regulatory modules in Saccharomyces cerevisiae
Source: Genome Biol. 2008 Jan 3;9(1):R2. doi: 10.1186/gb-2008-9-1-r2 (PMC2395236; doi:10.1186/gb-2008-9-1-r2)
Supplement: Additional data file 11 — Matrices describing all EPMs and RMs, including lists of synergistic pairs of regulators. [file gb-2008-9-1-r2-S11.zip › htmls/C0_EPMs_matrix/EPM_0.GO_enrichment.matrix.html]

|  |  |  |  |  |  |  |  |  |  |  |
| --- | --- | --- | --- | --- | --- | --- | --- | --- | --- | --- |
| Abf1 | Ste12 | Mbp1 | Stb1 | Swi6 | Swi4 | Tec1 | Pdr1 | Yap5 | Rap1 | Biological Process |
|  |  |  |  |  |  |  |  |  |  | P:protein folding |
|  |  |  |  |  |  |  |  |  |  | P:calcium-mediated signaling |
|  |  |  |  |  |  |  |  |  |  | P:chitin metabolism |
|  |  |  |  |  |  |  |  |  |  | P:cell wall chitin metabolism |
|  |  |  |  |  |  |  |  |  |  | P:chitin biosynthesis |
|  |  |  |  |  |  |  |  |  |  | P:chromosome localization |
|  |  |  |  |  |  |  |  |  |  | P:cellular polysaccharide metabolism |
|  |  |  |  |  |  |  |  |  |  | P:glucan metabolism |
|  |  |  |  |  |  |  |  |  |  | P:polysaccharide metabolism |
|  |  |  |  |  |  |  |  |  |  | P:1,3-beta-glucan metabolism |
|  |  |  |  |  |  |  |  |  |  | P:n-glycan processing |
|  |  |  |  |  |  |  |  |  |  | P:1,3-beta-glucan biosynthesis |
|  |  |  |  |  |  |  |  |  |  | P:glycoprotein metabolism |
|  |  |  |  |  |  |  |  |  |  | P:glycoprotein biosynthesis |
|  |  |  |  |  |  |  |  |  |  | P:protein amino acid glycosylation |
|  |  |  |  |  |  |  |  |  |  | P:biopolymer glycosylation |
|  |  |  |  |  |  |  |  |  |  | P:cell wall chitin biosynthesis |
|  |  |  |  |  |  |  |  |  |  | P:positive regulation of transcription by pheromones |
|  |  |  |  |  |  |  |  |  |  | P:positive regulation of transcription from RNA polymerase II promoter by pheromones |
|  |  |  |  |  |  |  |  |  |  | P:regulation of cell cycle |
|  |  |  |  |  |  |  |  |  |  | P:regulation of progression through cell cycle |
|  |  |  |  |  |  |  |  |  |  | P:growth |
|  |  |  |  |  |  |  |  |  |  | P:regulation of transcription by pheromones |
|  |  |  |  |  |  |  |  |  |  | P:signal transduction during filamentous growth |
|  |  |  |  |  |  |  |  |  |  | P:regulation of transcription from RNA polymerase II promoter by pheromones |
|  |  |  |  |  |  |  |  |  |  | P:filamentous growth |
|  |  |  |  |  |  |  |  |  |  | P:cell growth |
|  |  |  |  |  |  |  |  |  |  | P:cell surface receptor linked signal transduction |
|  |  |  |  |  |  |  |  |  |  | P:g-protein coupled receptor protein signaling pathway |
|  |  |  |  |  |  |  |  |  |  | P:pheromone-dependent signal transduction during conjugation with cellular fusion |
|  |  |  |  |  |  |  |  |  |  | P:regulation of conjugation |
|  |  |  |  |  |  |  |  |  |  | P:signal transduction during conjugation with cellular fusion |
|  |  |  |  |  |  |  |  |  |  | P:regulation of conjugation with cellular fusion |
|  |  |  |  |  |  |  |  |  |  | P:response to stimulus |
|  |  |  |  |  |  |  |  |  |  | P:cell cycle arrest |
|  |  |  |  |  |  |  |  |  |  | P:negative regulation of progression through cell cycle |
|  |  |  |  |  |  |  |  |  |  | P:response to pheromone during conjugation with cellular fusion |
|  |  |  |  |  |  |  |  |  |  | P:regulation of cell size |
|  |  |  |  |  |  |  |  |  |  | P:invasive growth (sensu Saccharomyces) |
|  |  |  |  |  |  |  |  |  |  | P:cell morphogenesis checkpoint |
|  |  |  |  |  |  |  |  |  |  | P:g2/M transition checkpoint |
|  |  |  |  |  |  |  |  |  |  | P:g2/M transition size control checkpoint |
|  |  |  |  |  |  |  |  |  |  | P:cell size control checkpoint |
|  |  |  |  |  |  |  |  |  |  | P:cell wall organization and biogenesis (sensu Fungi) |
|  |  |  |  |  |  |  |  |  |  | P:polysaccharide biosynthesis |
|  |  |  |  |  |  |  |  |  |  | P:biopolymer biosynthesis |
|  |  |  |  |  |  |  |  |  |  | P:cell wall biosynthesis |
|  |  |  |  |  |  |  |  |  |  | P:cell wall polysaccharide biosynthesis (sensu Fungi) |
|  |  |  |  |  |  |  |  |  |  | P:cell wall biosynthesis (sensu Fungi) |
|  |  |  |  |  |  |  |  |  |  | P:cytokinesis, site selection |
|  |  |  |  |  |  |  |  |  |  | P:bud site selection |
|  |  |  |  |  |  |  |  |  |  | P:establishment and/or maintenance of cell polarity |
|  |  |  |  |  |  |  |  |  |  | P:establishment and/or maintenance of cell polarity (sensu Fungi) |
|  |  |  |  |  |  |  |  |  |  | P:establishment of cell polarity |
|  |  |  |  |  |  |  |  |  |  | P:cytokinesis |
|  |  |  |  |  |  |  |  |  |  | P:establishment of cell polarity (sensu Fungi) |
|  |  |  |  |  |  |  |  |  |  | P:morphogenesis |
|  |  |  |  |  |  |  |  |  |  | P:cellular morphogenesis |
|  |  |  |  |  |  |  |  |  |  | P:development |
|  |  |  |  |  |  |  |  |  |  | P:reproduction |
|  |  |  |  |  |  |  |  |  |  | P:response to chemical stimulus |
|  |  |  |  |  |  |  |  |  |  | P:reproductive cellular physiological process |
|  |  |  |  |  |  |  |  |  |  | P:reproductive physiological process |
|  |  |  |  |  |  |  |  |  |  | P:interaction between organisms |
|  |  |  |  |  |  |  |  |  |  | P:conjugation with cellular fusion |
|  |  |  |  |  |  |  |  |  |  | P:conjugation |
|  |  |  |  |  |  |  |  |  |  | P:response to pheromone |
|  |  |  |  |  |  |  |  |  |  | P:sexual reproduction |
|
| Abf1 | Ste12 | Mbp1 | Stb1 | Swi6 | Swi4 | Tec1 | Pdr1 | Yap5 | Rap1 | Molecular Function |
|  |  |  |  |  |  |  |  |  |  | F:alpha-1,6-mannosyltransferase activity |
|  |  |  |  |  |  |  |  |  |  | F:rNA polymerase II transcription factor activity |
|  |  |  |  |  |  |  |  |  |  | F:alpha-1,2-mannosyltransferase activity |
|  |  |  |  |  |  |  |  |  |  | F:oxidoreductase activity, acting on CH2 groups |
|  |  |  |  |  |  |  |  |  |  | F:ribonucleoside-diphosphate reductase activity |
|  |  |  |  |  |  |  |  |  |  | F:oxidoreductase activity, acting on CH2 groups, disulfide as acceptor |
|  |  |  |  |  |  |  |  |  |  | F:delta24(24-1) sterol reductase activity |
|  |  |  |  |  |  |  |  |  |  | F:transferase activity, transferring hexosyl groups |
|  |  |  |  |  |  |  |  |  |  | F:transferase activity, transferring glycosyl groups |
|  |  |  |  |  |  |  |  |  |  | F:alpha-1,3-mannosyltransferase activity |
|  |  |  |  |  |  |  |  |  |  | F:mannosyltransferase activity |
|  |  |  |  |  |  |  |  |  |  | F:1,3-beta-glucan synthase activity |
|  |  |  |  |  |  |  |  |  |  | F:kinase inhibitor activity |
|  |  |  |  |  |  |  |  |  |  | F:protein kinase inhibitor activity |
|  |  |  |  |  |  |  |  |  |  | F:cyclin-dependent protein kinase inhibitor activity |
|  |  |  |  |  |  |  |  |  |  | F:mAP kinase activity |
|  |  |  |  |  |  |  |  |  |  | F:phosphotransferase activity, alcohol group as acceptor |
|  |  |  |  |  |  |  |  |  |  | F:protein kinase activity |
|  |  |  |  |  |  |  |  |  |  | F:cell adhesion molecule binding |
|  |  |  |  |  |  |  |  |  |  | F:prenylated protein tyrosine phosphatase activity |
|  |  |  |  |  |  |  |  |  |  | F:sphingosine-1-phosphate phosphatase activity |
|
| Abf1 | Ste12 | Mbp1 | Stb1 | Swi6 | Swi4 | Tec1 | Pdr1 | Yap5 | Rap1 | Cellular Component |
|  |  |  |  |  |  |  |  |  |  | C:transcription factor complex |
|  |  |  |  |  |  |  |  |  |  | C:mannosyltransferase complex |
|  |  |  |  |  |  |  |  |  |  | C:alpha-1,6-mannosyltransferase complex |
|  |  |  |  |  |  |  |  |  |  | C:organelle lumen |
|  |  |  |  |  |  |  |  |  |  | C:membrane-enclosed lumen |
|  |  |  |  |  |  |  |  |  |  | C:transcription factor TFIIE complex |
|  |  |  |  |  |  |  |  |  |  | C:dNA-directed RNA polymerase II, holoenzyme |
|  |  |  |  |  |  |  |  |  |  | C:bud neck septin structure |
|  |  |  |  |  |  |  |  |  |  | C:bud neck septin ring |
|  |  |  |  |  |  |  |  |  |  | C:cleavage apparatus septin structure |
|  |  |  |  |  |  |  |  |  |  | C:cell projection |
|  |  |  |  |  |  |  |  |  |  | C:mating projection |
|  |  |  |  |  |  |  |  |  |  | C:mating projection tip |
|  |  |  |  |  |  |  |  |  |  | C:cell projection part |
|  |  |  |  |  |  |  |  |  |  | C:ribonucleoside-diphosphate reductase complex |
|  |  |  |  |  |  |  |  |  |  | C:plasma membrane part |
|  |  |  |  |  |  |  |  |  |  | C:1,3-beta-glucan synthase complex |
|  |  |  |  |  |  |  |  |  |  | C:external encapsulating structure part |
|  |  |  |  |  |  |  |  |  |  | C:cell wall part |
|  |  |  |  |  |  |  |  |  |  | C:bud neck |
|  |  |  |  |  |  |  |  |  |  | C:cell cortex part |
|  |  |  |  |  |  |  |  |  |  | C:cell cortex |
|  |  |  |  |  |  |  |  |  |  | C:cytoskeleton |
|  |  |  |  |  |  |  |  |  |  | C:septin ring |
|  |  |  |  |  |  |  |  |  |  | C:septin cytoskeleton |
|  |  |  |  |  |  |  |  |  |  | C:site of polarized growth |
|  |  |  |  |  |  |  |  |  |  | C:bud scar |
|  |  |  |  |  |  |  |  |  |  | C:bud |
|  |  |  |  |  |  |  |  |  |  | C:external encapsulating structure |
|  |  |  |  |  |  |  |  |  |  | C:cell wall (sensu Fungi) |
|  |  |  |  |  |  |  |  |  |  | C:cell wall |
|
