# Supplementary material for: High-resolution analysis of condition-specific regulatory modules in Saccharomyces cerevisiae
Source: Genome Biol. 2008 Jan 3;9(1):R2. doi: 10.1186/gb-2008-9-1-r2 (PMC2395236; doi:10.1186/gb-2008-9-1-r2)
Supplement: Additional data file 11 — Matrices describing all EPMs and RMs, including lists of synergistic pairs of regulators. [file gb-2008-9-1-r2-S11.zip › htmls/C0_EPMs_matrix/EPM_1.GO_enrichment.matrix.html]

|  |  |  |  |  |  |  |  |  |
| --- | --- | --- | --- | --- | --- | --- | --- | --- |
| Gat3 | Abf1 | Yap5 | Swi4 | Swi6 | Pdr1 | Tec1 | Mat1mc | Biological Process |
|  |  |  |  |  |  |  |  | P:biological process unknown |
|  |  |  |  |  |  |  |  | P:positive regulation of protein biosynthesis |
|  |  |  |  |  |  |  |  | P:positive regulation of biosynthesis |
|  |  |  |  |  |  |  |  | P:positive regulation of translation |
|  |  |  |  |  |  |  |  | P:positive regulation of cellular biosynthesis |
|  |  |  |  |  |  |  |  | P:peptidyl-methionine modification |
|  |  |  |  |  |  |  |  | P:positive regulation of protein metabolism |
|  |  |  |  |  |  |  |  | P:interspecies interaction between organisms |
|  |  |  |  |  |  |  |  | P:n-terminal peptidyl-methionine acetylation |
|
| Gat3 | Abf1 | Yap5 | Swi4 | Swi6 | Pdr1 | Tec1 | Mat1mc | Molecular Function |
|  |  |  |  |  |  |  |  | F:molecular function unknown |
|
| Gat3 | Abf1 | Yap5 | Swi4 | Swi6 | Pdr1 | Tec1 | Mat1mc | Cellular Component |
|  |  |  |  |  |  |  |  | C:cellular component unknown |
|  |  |  |  |  |  |  |  | C:natB complex |
|
