# Supplementary material for: High-resolution analysis of condition-specific regulatory modules in Saccharomyces cerevisiae
Source: Genome Biol. 2008 Jan 3;9(1):R2. doi: 10.1186/gb-2008-9-1-r2 (PMC2395236; doi:10.1186/gb-2008-9-1-r2)
Supplement: Additional data file 11 — Matrices describing all EPMs and RMs, including lists of synergistic pairs of regulators. [file gb-2008-9-1-r2-S11.zip › htmls/C0_EPMs_matrix/EPM_10.GO_enrichment.matrix.html]

|  |  |  |  |  |  |  |  |  |  |  |  |  |  |  |  |  |
| --- | --- | --- | --- | --- | --- | --- | --- | --- | --- | --- | --- | --- | --- | --- | --- | --- |
| Yap1 | Swi6 | Sut1 | Leu3 | Put3 | Rpn4 | Ume6 | Stp1 | Skn7 | Gal4 | Pho2 | Swi5 | Ace2 | Snt2 | Msn2 | Msn4 | Biological Process |
|  |  |  |  |  |  |  |  |  |  |  |  |  |  |  |  | P:glutathione metabolism |
|  |  |  |  |  |  |  |  |  |  |  |  |  |  |  |  | P:signal transduction during filamentous growth |
|  |  |  |  |  |  |  |  |  |  |  |  |  |  |  |  | P:response to toxin |
|  |  |  |  |  |  |  |  |  |  |  |  |  |  |  |  | P:aldehyde catabolism |
|  |  |  |  |  |  |  |  |  |  |  |  |  |  |  |  | P:lactate metabolism |
|  |  |  |  |  |  |  |  |  |  |  |  |  |  |  |  | P:methylglyoxal metabolism |
|  |  |  |  |  |  |  |  |  |  |  |  |  |  |  |  | P:methylglyoxal catabolism to D-lactate |
|  |  |  |  |  |  |  |  |  |  |  |  |  |  |  |  | P:response to methylglyoxal |
|  |  |  |  |  |  |  |  |  |  |  |  |  |  |  |  | P:methylglyoxal catabolism |
|  |  |  |  |  |  |  |  |  |  |  |  |  |  |  |  | P:phosphorylated carbohydrate dephosphorylation |
|  |  |  |  |  |  |  |  |  |  |  |  |  |  |  |  | P:inositol phosphate dephosphorylation |
|  |  |  |  |  |  |  |  |  |  |  |  |  |  |  |  | P:late endosome to vacuole transport |
|  |  |  |  |  |  |  |  |  |  |  |  |  |  |  |  | P:cTP biosynthesis |
|  |  |  |  |  |  |  |  |  |  |  |  |  |  |  |  | P:pyrimidine ribonucleotide biosynthesis |
|  |  |  |  |  |  |  |  |  |  |  |  |  |  |  |  | P:pyrimidine ribonucleotide metabolism |
|  |  |  |  |  |  |  |  |  |  |  |  |  |  |  |  | P:pyrimidine ribonucleoside triphosphate biosynthesis |
|  |  |  |  |  |  |  |  |  |  |  |  |  |  |  |  | P:pyrimidine ribonucleoside triphosphate metabolism |
|  |  |  |  |  |  |  |  |  |  |  |  |  |  |  |  | P:cTP metabolism |
|  |  |  |  |  |  |  |  |  |  |  |  |  |  |  |  | P:anaerobic respiration |
|  |  |  |  |  |  |  |  |  |  |  |  |  |  |  |  | P:aerobic respiration |
|  |  |  |  |  |  |  |  |  |  |  |  |  |  |  |  | P:cellular respiration |
|  |  |  |  |  |  |  |  |  |  |  |  |  |  |  |  | P:citrate metabolism |
|  |  |  |  |  |  |  |  |  |  |  |  |  |  |  |  | P:acetyl-CoA metabolism |
|  |  |  |  |  |  |  |  |  |  |  |  |  |  |  |  | P:tricarboxylic acid cycle intermediate metabolism |
|  |  |  |  |  |  |  |  |  |  |  |  |  |  |  |  | P:cofactor catabolism |
|  |  |  |  |  |  |  |  |  |  |  |  |  |  |  |  | P:coenzyme catabolism |
|  |  |  |  |  |  |  |  |  |  |  |  |  |  |  |  | P:glutamate metabolism |
|  |  |  |  |  |  |  |  |  |  |  |  |  |  |  |  | P:urea cycle intermediate metabolism |
|  |  |  |  |  |  |  |  |  |  |  |  |  |  |  |  | P:tricarboxylic acid cycle |
|  |  |  |  |  |  |  |  |  |  |  |  |  |  |  |  | P:arginine metabolism |
|  |  |  |  |  |  |  |  |  |  |  |  |  |  |  |  | P:acetyl-CoA catabolism |
|  |  |  |  |  |  |  |  |  |  |  |  |  |  |  |  | P:glutamate biosynthesis |
|  |  |  |  |  |  |  |  |  |  |  |  |  |  |  |  | P:arginine biosynthesis |
|  |  |  |  |  |  |  |  |  |  |  |  |  |  |  |  | P:amine metabolism |
|  |  |  |  |  |  |  |  |  |  |  |  |  |  |  |  | P:mitochondrial genome maintenance |
|  |  |  |  |  |  |  |  |  |  |  |  |  |  |  |  | P:glutamine family amino acid biosynthesis |
|  |  |  |  |  |  |  |  |  |  |  |  |  |  |  |  | P:amino acid biosynthesis |
|  |  |  |  |  |  |  |  |  |  |  |  |  |  |  |  | P:amino acid and derivative metabolism |
|  |  |  |  |  |  |  |  |  |  |  |  |  |  |  |  | P:amino acid metabolism |
|  |  |  |  |  |  |  |  |  |  |  |  |  |  |  |  | P:nitrogen compound metabolism |
|  |  |  |  |  |  |  |  |  |  |  |  |  |  |  |  | P:organic acid metabolism |
|  |  |  |  |  |  |  |  |  |  |  |  |  |  |  |  | P:carboxylic acid metabolism |
|  |  |  |  |  |  |  |  |  |  |  |  |  |  |  |  | P:nitrogen compound biosynthesis |
|  |  |  |  |  |  |  |  |  |  |  |  |  |  |  |  | P:glutamine family amino acid metabolism |
|  |  |  |  |  |  |  |  |  |  |  |  |  |  |  |  | P:amine biosynthesis |
|  |  |  |  |  |  |  |  |  |  |  |  |  |  |  |  | P:sodium ion homeostasis |
|  |  |  |  |  |  |  |  |  |  |  |  |  |  |  |  | P:pentose-phosphate shunt, oxidative branch |
|  |  |  |  |  |  |  |  |  |  |  |  |  |  |  |  | P:response to hydrogen peroxide |
|  |  |  |  |  |  |  |  |  |  |  |  |  |  |  |  | P:cell wall organization and biogenesis |
|  |  |  |  |  |  |  |  |  |  |  |  |  |  |  |  | P:external encapsulating structure organization and biogenesis |
|  |  |  |  |  |  |  |  |  |  |  |  |  |  |  |  | P:sulfur utilization |
|  |  |  |  |  |  |  |  |  |  |  |  |  |  |  |  | P:sulfate assimilation |
|  |  |  |  |  |  |  |  |  |  |  |  |  |  |  |  | P:copper ion homeostasis |
|  |  |  |  |  |  |  |  |  |  |  |  |  |  |  |  | P:zinc ion homeostasis |
|  |  |  |  |  |  |  |  |  |  |  |  |  |  |  |  | P:cell redox homeostasis |
|  |  |  |  |  |  |  |  |  |  |  |  |  |  |  |  | P:regulation of cell redox homeostasis |
|  |  |  |  |  |  |  |  |  |  |  |  |  |  |  |  | P:response to oxidative stress |
|  |  |  |  |  |  |  |  |  |  |  |  |  |  |  |  | P:oxygen and reactive oxygen species metabolism |
|  |  |  |  |  |  |  |  |  |  |  |  |  |  |  |  | P:organelle inheritance |
|  |  |  |  |  |  |  |  |  |  |  |  |  |  |  |  | P:cell homeostasis |
|  |  |  |  |  |  |  |  |  |  |  |  |  |  |  |  | P:homeostasis |
|  |  |  |  |  |  |  |  |  |  |  |  |  |  |  |  | P:aging |
|  |  |  |  |  |  |  |  |  |  |  |  |  |  |  |  | P:cell aging |
|  |  |  |  |  |  |  |  |  |  |  |  |  |  |  |  | P:cell ion homeostasis |
|  |  |  |  |  |  |  |  |  |  |  |  |  |  |  |  | P:ion homeostasis |
|  |  |  |  |  |  |  |  |  |  |  |  |  |  |  |  | P:response to chemical stimulus |
|  |  |  |  |  |  |  |  |  |  |  |  |  |  |  |  | P:age-dependent response to reactive oxygen species during chronological cell aging |
|  |  |  |  |  |  |  |  |  |  |  |  |  |  |  |  | P:age-dependent response to reactive oxygen species |
|  |  |  |  |  |  |  |  |  |  |  |  |  |  |  |  | P:response to stimulus |
|  |  |  |  |  |  |  |  |  |  |  |  |  |  |  |  | P:response to stress |
|  |  |  |  |  |  |  |  |  |  |  |  |  |  |  |  | P:response to reactive oxygen species |
|  |  |  |  |  |  |  |  |  |  |  |  |  |  |  |  | P:superoxide metabolism |
|  |  |  |  |  |  |  |  |  |  |  |  |  |  |  |  | P:chronological cell aging |
|  |  |  |  |  |  |  |  |  |  |  |  |  |  |  |  | P:age-dependent general metabolic decline |
|  |  |  |  |  |  |  |  |  |  |  |  |  |  |  |  | P:age-dependent response to oxidative stress |
|  |  |  |  |  |  |  |  |  |  |  |  |  |  |  |  | P:age-dependent response to oxidative stress during chronological cell aging |
|  |  |  |  |  |  |  |  |  |  |  |  |  |  |  |  | P:age-dependent general metabolic decline during chronological cell aging |
|  |  |  |  |  |  |  |  |  |  |  |  |  |  |  |  | P:energy derivation by oxidation of organic compounds |
|  |  |  |  |  |  |  |  |  |  |  |  |  |  |  |  | P:cellular metabolism |
|  |  |  |  |  |  |  |  |  |  |  |  |  |  |  |  | P:cellular process |
|  |  |  |  |  |  |  |  |  |  |  |  |  |  |  |  | P:transition metal ion transport |
|  |  |  |  |  |  |  |  |  |  |  |  |  |  |  |  | P:cellular physiological process |
|  |  |  |  |  |  |  |  |  |  |  |  |  |  |  |  | P:pyruvate metabolism |
|  |  |  |  |  |  |  |  |  |  |  |  |  |  |  |  | P:catabolism |
|  |  |  |  |  |  |  |  |  |  |  |  |  |  |  |  | P:generation of precursor metabolites and energy |
|  |  |  |  |  |  |  |  |  |  |  |  |  |  |  |  | P:metabolism |
|  |  |  |  |  |  |  |  |  |  |  |  |  |  |  |  | P:copper ion transport |
|  |  |  |  |  |  |  |  |  |  |  |  |  |  |  |  | P:physiological process |
|  |  |  |  |  |  |  |  |  |  |  |  |  |  |  |  | P:double-strand break repair via single-strand annealing |
|  |  |  |  |  |  |  |  |  |  |  |  |  |  |  |  | P:double-strand break repair via synthesis-dependent strand annealing |
|  |  |  |  |  |  |  |  |  |  |  |  |  |  |  |  | P:branched chain family amino acid catabolism |
|  |  |  |  |  |  |  |  |  |  |  |  |  |  |  |  | P:mitochondrion organization and biogenesis |
|  |  |  |  |  |  |  |  |  |  |  |  |  |  |  |  | P:heteroduplex formation |
|  |  |  |  |  |  |  |  |  |  |  |  |  |  |  |  | P:strand invasion |
|  |  |  |  |  |  |  |  |  |  |  |  |  |  |  |  | P:meiotic joint molecule formation |
|
| Yap1 | Swi6 | Sut1 | Leu3 | Put3 | Rpn4 | Ume6 | Stp1 | Skn7 | Gal4 | Pho2 | Swi5 | Ace2 | Snt2 | Msn2 | Msn4 | Molecular Function |
|  |  |  |  |  |  |  |  |  |  |  |  |  |  |  |  | F:lactate dehydrogenase activity |
|  |  |  |  |  |  |  |  |  |  |  |  |  |  |  |  | F:oxidoreductase activity, acting on CH-OH group of donors |
|  |  |  |  |  |  |  |  |  |  |  |  |  |  |  |  | F:oxidoreductase activity, acting on the CH-OH group of donors, cytochrome as acceptor |
|  |  |  |  |  |  |  |  |  |  |  |  |  |  |  |  | F:structural molecule activity |
|  |  |  |  |  |  |  |  |  |  |  |  |  |  |  |  | F:glucose-6-phosphate 1-dehydrogenase activity |
|  |  |  |  |  |  |  |  |  |  |  |  |  |  |  |  | F:d-lactate dehydrogenase (cytochrome) activity |
|  |  |  |  |  |  |  |  |  |  |  |  |  |  |  |  | F:lactoylglutathione lyase activity |
|  |  |  |  |  |  |  |  |  |  |  |  |  |  |  |  | F:carbon-sulfur lyase activity |
|  |  |  |  |  |  |  |  |  |  |  |  |  |  |  |  | F:exopeptidase activity |
|  |  |  |  |  |  |  |  |  |  |  |  |  |  |  |  | F:dipeptidyl-peptidase and tripeptidyl-peptidase activity |
|  |  |  |  |  |  |  |  |  |  |  |  |  |  |  |  | F:recombinase activity |
|  |  |  |  |  |  |  |  |  |  |  |  |  |  |  |  | F:g-protein coupled receptor activity |
|  |  |  |  |  |  |  |  |  |  |  |  |  |  |  |  | F:mating-type factor pheromone receptor activity |
|  |  |  |  |  |  |  |  |  |  |  |  |  |  |  |  | F:inositol-1(or 4)-monophosphatase activity |
|  |  |  |  |  |  |  |  |  |  |  |  |  |  |  |  | F:mating-type a-factor pheromone receptor activity |
|  |  |  |  |  |  |  |  |  |  |  |  |  |  |  |  | F:pheromone receptor activity |
|  |  |  |  |  |  |  |  |  |  |  |  |  |  |  |  | F:cTP synthase activity |
|  |  |  |  |  |  |  |  |  |  |  |  |  |  |  |  | F:isocitrate dehydrogenase (NAD+) activity |
|  |  |  |  |  |  |  |  |  |  |  |  |  |  |  |  | F:argininosuccinate lyase activity |
|  |  |  |  |  |  |  |  |  |  |  |  |  |  |  |  | F:amidine-lyase activity |
|  |  |  |  |  |  |  |  |  |  |  |  |  |  |  |  | F:branched-chain-amino-acid transaminase activity |
|  |  |  |  |  |  |  |  |  |  |  |  |  |  |  |  | F:aconitate hydratase activity |
|  |  |  |  |  |  |  |  |  |  |  |  |  |  |  |  | F:s-acetyltransferase activity |
|  |  |  |  |  |  |  |  |  |  |  |  |  |  |  |  | F:dihydrolipoamide S-acyltransferase activity |
|  |  |  |  |  |  |  |  |  |  |  |  |  |  |  |  | F:dihydrolipoyllysine-residue acetyltransferase activity |
|  |  |  |  |  |  |  |  |  |  |  |  |  |  |  |  | F:structural constituent of cell wall |
|  |  |  |  |  |  |  |  |  |  |  |  |  |  |  |  | F:carboxyl- and carbamoyltransferase activity |
|  |  |  |  |  |  |  |  |  |  |  |  |  |  |  |  | F:inositol-3-phosphate synthase activity |
|  |  |  |  |  |  |  |  |  |  |  |  |  |  |  |  | F:intramolecular lyase activity |
|  |  |  |  |  |  |  |  |  |  |  |  |  |  |  |  | F:ornithine carbamoyltransferase activity |
|  |  |  |  |  |  |  |  |  |  |  |  |  |  |  |  | F:oxidoreductase activity, acting on the CH-CH group of donors, quinone or related compound as acceptor |
|  |  |  |  |  |  |  |  |  |  |  |  |  |  |  |  | F:carboxypeptidase activity |
|  |  |  |  |  |  |  |  |  |  |  |  |  |  |  |  | F:succinate dehydrogenase (ubiquinone) activity |
|  |  |  |  |  |  |  |  |  |  |  |  |  |  |  |  | F:serine carboxypeptidase activity |
|  |  |  |  |  |  |  |  |  |  |  |  |  |  |  |  | F:saccharolysin activity |
|  |  |  |  |  |  |  |  |  |  |  |  |  |  |  |  | F:carboxypeptidase C activity |
|  |  |  |  |  |  |  |  |  |  |  |  |  |  |  |  | F:copper, zinc superoxide dismutase activity |
|  |  |  |  |  |  |  |  |  |  |  |  |  |  |  |  | F:alcohol dehydrogenase (NADP+) activity |
|  |  |  |  |  |  |  |  |  |  |  |  |  |  |  |  | F:thiol-disulfide exchange intermediate activity |
|  |  |  |  |  |  |  |  |  |  |  |  |  |  |  |  | F:aldo-keto reductase activity |
|  |  |  |  |  |  |  |  |  |  |  |  |  |  |  |  | F:oxidoreductase activity |
|  |  |  |  |  |  |  |  |  |  |  |  |  |  |  |  | F:oxidoreductase activity, acting on superoxide radicals as acceptor |
|  |  |  |  |  |  |  |  |  |  |  |  |  |  |  |  | F:superoxide dismutase activity |
|  |  |  |  |  |  |  |  |  |  |  |  |  |  |  |  | F:manganese superoxide dismutase activity |
|  |  |  |  |  |  |  |  |  |  |  |  |  |  |  |  | F:copper uptake transporter activity |
|  |  |  |  |  |  |  |  |  |  |  |  |  |  |  |  | F:thioredoxin peroxidase activity |
|  |  |  |  |  |  |  |  |  |  |  |  |  |  |  |  | F:phosphoglycerate kinase activity |
|  |  |  |  |  |  |  |  |  |  |  |  |  |  |  |  | F:antioxidant activity |
|  |  |  |  |  |  |  |  |  |  |  |  |  |  |  |  | F:catalytic activity |
|  |  |  |  |  |  |  |  |  |  |  |  |  |  |  |  | F:peroxidase activity |
|  |  |  |  |  |  |  |  |  |  |  |  |  |  |  |  | F:oxidoreductase activity, acting on peroxide as acceptor |
|
| Yap1 | Swi6 | Sut1 | Leu3 | Put3 | Rpn4 | Ume6 | Stp1 | Skn7 | Gal4 | Pho2 | Swi5 | Ace2 | Snt2 | Msn2 | Msn4 | Cellular Component |
|  |  |  |  |  |  |  |  |  |  |  |  |  |  |  |  | C:vacuolar lumen (sensu Fungi) |
|  |  |  |  |  |  |  |  |  |  |  |  |  |  |  |  | C:cell part |
|  |  |  |  |  |  |  |  |  |  |  |  |  |  |  |  | C:cell |
|  |  |  |  |  |  |  |  |  |  |  |  |  |  |  |  | C:vacuole |
|  |  |  |  |  |  |  |  |  |  |  |  |  |  |  |  | C:vacuole (sensu Fungi) |
|  |  |  |  |  |  |  |  |  |  |  |  |  |  |  |  | C:storage vacuole |
|  |  |  |  |  |  |  |  |  |  |  |  |  |  |  |  | C:lytic vacuole |
|  |  |  |  |  |  |  |  |  |  |  |  |  |  |  |  | C:cytoplasmic part |
|  |  |  |  |  |  |  |  |  |  |  |  |  |  |  |  | C:cytoplasm |
|  |  |  |  |  |  |  |  |  |  |  |  |  |  |  |  | C:membrane-bound organelle |
|  |  |  |  |  |  |  |  |  |  |  |  |  |  |  |  | C:intracellular membrane-bound organelle |
|  |  |  |  |  |  |  |  |  |  |  |  |  |  |  |  | C:anchored to membrane |
|  |  |  |  |  |  |  |  |  |  |  |  |  |  |  |  | C:anchored to plasma membrane |
|  |  |  |  |  |  |  |  |  |  |  |  |  |  |  |  | C:external encapsulating structure |
|  |  |  |  |  |  |  |  |  |  |  |  |  |  |  |  | C:cell wall (sensu Fungi) |
|  |  |  |  |  |  |  |  |  |  |  |  |  |  |  |  | C:cell wall |
|  |  |  |  |  |  |  |  |  |  |  |  |  |  |  |  | C:cytosol |
|  |  |  |  |  |  |  |  |  |  |  |  |  |  |  |  | C:mitochondrial matrix |
|  |  |  |  |  |  |  |  |  |  |  |  |  |  |  |  | C:mitochondrial lumen |
|
