# Supplementary material for: High-resolution analysis of condition-specific regulatory modules in Saccharomyces cerevisiae
Source: Genome Biol. 2008 Jan 3;9(1):R2. doi: 10.1186/gb-2008-9-1-r2 (PMC2395236; doi:10.1186/gb-2008-9-1-r2)
Supplement: Additional data file 11 — Matrices describing all EPMs and RMs, including lists of synergistic pairs of regulators. [file gb-2008-9-1-r2-S11.zip › htmls/C0_EPMs_matrix/EPM_10.Overlap.matrix.html]

|  |  |  |  |  |  |  |  |  |  |  |  |  |  |  |  |
| --- | --- | --- | --- | --- | --- | --- | --- | --- | --- | --- | --- | --- | --- | --- | --- |
| Yap1 | Swi6 | Sut1 | Leu3 | Put3 | Rpn4 | Ume6 | Stp1 | Skn7 | Gal4 | Pho2 | Swi5 | Ace2 | Snt2 | Msn2 | Msn4 |
|  |  |  |  |  |  |  |  |  |  |  |  |  |  |  |  | Yap1 |
|  |  |  |  |  |  |  |  |  |  |  |  |  |  |  |  | Swi6 |
|  |  |  |  |  |  |  |  |  |  |  |  |  |  |  |  | Sut1 |
|  |  |  |  |  |  |  |  |  |  |  |  |  |  |  |  | Leu3 |
|  |  |  |  |  |  |  |  |  |  |  |  |  |  |  |  | Put3 |
|  |  |  |  |  |  |  |  |  |  |  |  |  |  |  |  | Rpn4 |
|  |  |  |  |  |  |  |  |  |  |  |  |  |  |  |  | Ume6 |
|  |  |  |  |  |  |  |  |  |  |  |  |  |  |  |  | Stp1 |
|  |  |  |  |  |  |  |  |  |  |  |  |  |  |  |  | Skn7 |
|  |  |  |  |  |  |  |  |  |  |  |  |  |  |  |  | Gal4 |
|  |  |  |  |  |  |  |  |  |  |  |  |  |  |  |  | Pho2 |
|  |  |  |  |  |  |  |  |  |  |  |  |  |  |  |  | Swi5 |
|  |  |  |  |  |  |  |  |  |  |  |  |  |  |  |  | Ace2 |
|  |  |  |  |  |  |  |  |  |  |  |  |  |  |  |  | Snt2 |
|  |  |  |  |  |  |  |  |  |  |  |  |  |  |  |  | Msn2 |
|  |  |  |  |  |  |  |  |  |  |  |  |  |  |  |  | Msn4 |
 Yap1 | Swi6 | Sut1 | Leu3 | Put3 | Rpn4 | Ume6 | Stp1 | Skn7 | Gal4 | Pho2 | Swi5 | Ace2 | Snt2 | Msn2 | Msn4 |
