# Supplementary material for: High-resolution analysis of condition-specific regulatory modules in Saccharomyces cerevisiae
Source: Genome Biol. 2008 Jan 3;9(1):R2. doi: 10.1186/gb-2008-9-1-r2 (PMC2395236; doi:10.1186/gb-2008-9-1-r2)
Supplement: Additional data file 11 — Matrices describing all EPMs and RMs, including lists of synergistic pairs of regulators. [file gb-2008-9-1-r2-S11.zip › htmls/C0_EPMs_matrix/EPM_11.RM.matrix.html]

Regulators vs. RM target gene list

|  |  |  |  |  |  |  |  |  |  |  |  |  |  |  |  |  |  |  |  |  |  |  |  |  |  |  |  |  |  |  |  |  |  |  |  |  |  |  |  |  |  |  |
| --- | --- | --- | --- | --- | --- | --- | --- | --- | --- | --- | --- | --- | --- | --- | --- | --- | --- | --- | --- | --- | --- | --- | --- | --- | --- | --- | --- | --- | --- | --- | --- | --- | --- | --- | --- | --- | --- | --- | --- | --- | --- | --- |
|  | Hap3 | Hap2 | Hap4 | Ace2 | Mbp1 | Hsf1 | Rlm1 | Sko1 | Cin5 | Aft2 | Fkh2 | Yap6 | Rox1 | Hap1 | Bas1 | Nrg1 | Rph1 | Mcm1 | Mig1 | Rgt1 | Sok2 | Ino2 | Ume6 | Rpn4 | Dal81 | Snt2 | Gal80 | Phd1 | Put3 | Uga3 | Leu3 | Pho2 | Gal4 | Adr1 | Rds1 | Pdr3 | Pdr1 | Msn2 | Msn4 | Stp1 | Skn7 | Sut1 |
| RM\_1 |  |  |  |  |  |  |  |  |  |  |  |  |  |  |  |  |  |  |  |  |  |  |  |  |  |  |  |  |  |  |  |  |  |  |  |  |  |  |  |  |  |  |
| RM\_2 |  |  |  |  |  |  |  |  |  |  |  |  |  |  |  |  |  |  |  |  |  |  |  |  |  |  |  |  |  |  |  |  |  |  |  |  |  |  |  |  |  |  |
| RM\_3 |  |  |  |  |  |  |  |  |  |  |  |  |  |  |  |  |  |  |  |  |  |  |  |  |  |  |  |  |  |  |  |  |  |  |  |  |  |  |  |  |  |  |
| RM\_4 |  |  |  |  |  |  |  |  |  |  |  |  |  |  |  |  |  |  |  |  |  |  |  |  |  |  |  |  |  |  |  |  |  |  |  |  |  |  |  |  |  |  |
| RM\_5 |  |  |  |  |  |  |  |  |  |  |  |  |  |  |  |  |  |  |  |  |  |  |  |  |  |  |  |  |  |  |  |  |  |  |  |  |  |  |  |  |  |  |
| RM\_6 |  |  |  |  |  |  |  |  |  |  |  |  |  |  |  |  |  |  |  |  |  |  |  |  |  |  |  |  |  |  |  |  |  |  |  |  |  |  |  |  |  |  |
| RM\_7 |  |  |  |  |  |  |  |  |  |  |  |  |  |  |  |  |  |  |  |  |  |  |  |  |  |  |  |  |  |  |  |  |  |  |  |  |  |  |  |  |  |  |
| RM\_8 |  |  |  |  |  |  |  |  |  |  |  |  |  |  |  |  |  |  |  |  |  |  |  |  |  |  |  |  |  |  |  |  |  |  |  |  |  |  |  |  |  |  |
| RM\_9 |  |  |  |  |  |  |  |  |  |  |  |  |  |  |  |  |  |  |  |  |  |  |  |  |  |  |  |  |  |  |  |  |  |  |  |  |  |  |  |  |  |  |
| RM\_10 |  |  |  |  |  |  |  |  |  |  |  |  |  |  |  |  |  |  |  |  |  |  |  |  |  |  |  |  |  |  |  |  |  |  |  |  |  |  |  |  |  |  |
| RM\_11 |  |  |  |  |  |  |  |  |  |  |  |  |  |  |  |  |  |  |  |  |  |  |  |  |  |  |  |  |  |  |  |  |  |  |  |  |  |  |  |  |  |  |
| RM\_12 |  |  |  |  |  |  |  |  |  |  |  |  |  |  |  |  |  |  |  |  |  |  |  |  |  |  |  |  |  |  |  |  |  |  |  |  |  |  |  |  |  |  |
| RM\_13 |  |  |  |  |  |  |  |  |  |  |  |  |  |  |  |  |  |  |  |  |  |  |  |  |  |  |  |  |  |  |  |  |  |  |  |  |  |  |  |  |  |  |
| RM\_14 |  |  |  |  |  |  |  |  |  |  |  |  |  |  |  |  |  |  |  |  |  |  |  |  |  |  |  |  |  |  |  |  |  |  |  |  |  |  |  |  |  |  |
| RM\_15 |  |  |  |  |  |  |  |  |  |  |  |  |  |  |  |  |  |  |  |  |  |  |  |  |  |  |  |  |  |  |  |  |  |  |  |  |  |  |  |  |  |  |
| RM\_16 |  |  |  |  |  |  |  |  |  |  |  |  |  |  |  |  |  |  |  |  |  |  |  |  |  |  |  |  |  |  |  |  |  |  |  |  |  |  |  |  |  |  |
| RM\_17 |  |  |  |  |  |  |  |  |  |  |  |  |  |  |  |  |  |  |  |  |  |  |  |  |  |  |  |  |  |  |  |  |  |  |  |  |  |  |  |  |  |  |
| RM\_18 |  |  |  |  |  |  |  |  |  |  |  |  |  |  |  |  |  |  |  |  |  |  |  |  |  |  |  |  |  |  |  |  |  |  |  |  |  |  |  |  |  |  |
| RM\_19 |  |  |  |  |  |  |  |  |  |  |  |  |  |  |  |  |  |  |  |  |  |  |  |  |  |  |  |  |  |  |  |  |  |  |  |  |  |  |  |  |  |  |
| RM\_20 |  |  |  |  |  |  |  |  |  |  |  |  |  |  |  |  |  |  |  |  |  |  |  |  |  |  |  |  |  |  |  |  |  |  |  |  |  |  |  |  |  |  |
| RM\_21 |  |  |  |  |  |  |  |  |  |  |  |  |  |  |  |  |  |  |  |  |  |  |  |  |  |  |  |  |  |  |  |  |  |  |  |  |  |  |  |  |  |  |
| RM\_22 |  |  |  |  |  |  |  |  |  |  |  |  |  |  |  |  |  |  |  |  |  |  |  |  |  |  |  |  |  |  |  |  |  |  |  |  |  |  |  |  |  |  |
| RM\_23 |  |  |  |  |  |  |  |  |  |  |  |  |  |  |  |  |  |  |  |  |  |  |  |  |  |  |  |  |  |  |  |  |  |  |  |  |  |  |  |  |  |  |
| RM\_24 |  |  |  |  |  |  |  |  |  |  |  |  |  |  |  |  |  |  |  |  |  |  |  |  |  |  |  |  |  |  |  |  |  |  |  |  |  |  |  |  |  |  |
| RM\_25 |  |  |  |  |  |  |  |  |  |  |  |  |  |  |  |  |  |  |  |  |  |  |  |  |  |  |  |  |  |  |  |  |  |  |  |  |  |  |  |  |  |  |

Synergistic Pair of Regulators

1. Sok2\*Sut1

2. Cin5\*Msn4

3. Fkh2\*Yap6

4. Cin5\*Fkh2

5. Rox1\*Sut1

6. Ace2\*Mbp1

7. Gal80\*Msn4

8. Gal80\*Msn2

9. Msn4\*Uga3

10. Msn2\*Put3

11. Msn4\*Put3

12. Msn2\*Rgt1

13. Msn4\*Rgt1

14. Msn2\*Pho2

15. Msn4\*Pho2

16. Msn2\*Uga3

17. Msn2\*Msn4

18. Msn2\*Stp1

19. Msn4\*Stp1

20. Msn2\*Rph1

21. Msn4\*Rph1

22. Msn2\*Skn7

23. Msn4\*Skn7

24. Gal4\*Msn2

25. Gal4\*Msn4

26. Msn4\*Sut1

27. Adr1\*Msn2

28. Msn2\*Ume6

29. Msn4\*Ume6

30. Leu3\*Msn4

31. Msn2\*Sut1

32. Dal81\*Msn4

33. Adr1\*Msn4

34. Msn4\*Rds1

35. Msn4\*Nrg1

36. Msn2\*Phd1

37. Msn4\*Phd1

38. Dal81\*Msn2

39. Msn2\*Pdr1

40. Msn4\*Pdr1

41. Msn2\*Rds1

42. Leu3\*Msn2

43. Mig1\*Msn4

44. Msn4\*Snt2

45. Msn2\*Snt2

46. Mig1\*Msn2

47. Msn2\*Nrg1

48. Msn2\*Pdr3

49. Msn4\*Pdr3

50. Ino2\*Msn2

51. Ino2\*Msn4

52. Put3\*Sut1

53. Adr1\*Sut1

54. Gal80\*Sut1

55. Msn2\*Rpn4

56. Msn4\*Rpn4

57. Put3\*Rds1

58. Sut1\*Uga3

59. Rph1\*Sut1

60. Rgt1\*Sut1

61. Leu3\*Sut1

62. Nrg1\*Stp1

63. Rgt1\*Skn7

64. Ino2\*Ume6

65. Adr1\*Gal80

66. Rpn4\*Ume6

67. Gal80\*Skn7

68. Stp1\*Ume6

69. Pdr1\*Sut1

70. Nrg1\*Sut1

71. Mig1\*Rgt1

72. Stp1\*Sut1

73. Pho2\*Rds1

74. Leu3\*Pdr3

75. Gal4\*Sut1

76. Mig1\*Sut1

77. Gal80\*Uga3

78. Snt2\*Sut1

79. Gal80\*Pdr1

80. Skn7\*Snt2

81. Pdr3\*Uga3

82. Adr1\*Rds1

83. Phd1\*Sut1

84. Gal80\*Pdr3

85. Gal80\*Put3

86. Pdr3\*Put3

87. Pho2\*Sut1

88. Gal80\*Ume6

89. Gal80\*Pho2

90. Pdr1\*Rgt1

91. Pdr3\*Rgt1

92. Pho2\*Skn7

93. Rds1\*Sut1

94. Adr1\*Gal4

95. Pdr1\*Uga3

96. Adr1\*Pdr3

97. Rds1\*Uga3

98. Put3\*Ume6

99. Skn7\*Uga3

100. Adr1\*Put3

101. Put3\*Stp1

102. Leu3\*Put3

103. Leu3\*Pdr1

104. Sut1\*Ume6

105. Pdr3\*Sut1

106. Skn7\*Stp1

107. Pho2\*Ume6

108. Put3\*Skn7

109. Leu3\*Phd1

110. Pdr1\*Pdr3

111. Dal81\*Ume6

112. Pho2\*Rgt1

113. Gal4\*Mig1

114. Pdr1\*Put3

115. Adr1\*Uga3

116. Pdr1\*Ume6

117. Leu3\*Skn7

118. Pho2\*Put3

119. Skn7\*Sut1

120. Pdr3\*Rds1

121. Pdr3\*Ume6

122. Put3\*Uga3

123. Rgt1\*Stp1

124. Rgt1\*Ume6

125. Leu3\*Rds1

126. Pho2\*Uga3

127. Stp1\*Uga3

128. Ino2\*Pdr3

129. Mig1\*Pdr1

130. Mig1\*Pdr3

131. Adr1\*Skn7

132. Phd1\*Uga3

133. Rph1\*Skn7

134. Leu3\*Uga3

135. Put3\*Rgt1

136. Rgt1\*Uga3

137. Nrg1\*Skn7

138. Gal4\*Gal80

139. Leu3\*Stp1

140. Bas1\*Skn7

141. Pdr3\*Pho2

142. Gal80\*Snt2

143. Gal80\*Leu3

144. Phd1\*Stp1

145. Gal4\*Skn7

146. Phd1\*Put3

147. Mig1\*Uga3

148. Skn7\*Ume6

149. Mig1\*Skn7

150. Gal4\*Pdr3

151. Phd1\*Pho2

152. Mig1\*Put3

153. Gal4\*Pdr1

154. Pdr3\*Stp1

155. Gal4\*Uga3

156. Mcm1\*Pdr1

157. Hap2\*Hap4

158. Hap3\*Hap4

Matrix of enriched GO

EPM matrix
