# Supplementary material for: High-resolution analysis of condition-specific regulatory modules in Saccharomyces cerevisiae
Source: Genome Biol. 2008 Jan 3;9(1):R2. doi: 10.1186/gb-2008-9-1-r2 (PMC2395236; doi:10.1186/gb-2008-9-1-r2)
Supplement: Additional data file 11 — Matrices describing all EPMs and RMs, including lists of synergistic pairs of regulators. [file gb-2008-9-1-r2-S11.zip › htmls/C0_EPMs_matrix/EPM_12.GO_enrichment.matrix.html]

|  |  |  |  |
| --- | --- | --- | --- |
| Aft2 | Ume6 | Rpn4 | Biological Process |
|  |  |  | P:siderophore transport |
|  |  |  | P:fatty acid beta-oxidation |
|  |  |  | P:synapsis |
|  |  |  | P:acetate metabolism |
|  |  |  | P:carboxylic acid metabolism |
|  |  |  | P:organic acid metabolism |
|  |  |  | P:mAPKKK cascade during cell wall biogenesis |
|  |  |  | P:acetyl-CoA biosynthesis |
|  |  |  | P:acetate fermentation |
|  |  |  | P:carnitine metabolism |
|  |  |  | P:cell communication |
|  |  |  | P:intracellular signaling cascade |
|  |  |  | P:signal transduction |
|
| Aft2 | Ume6 | Rpn4 | Molecular Function |
|  |  |  | F:t-SNARE activity |
|  |  |  | F:clathrin binding |
|  |  |  | F:ligase activity, forming carbon-sulfur bonds |
|  |  |  | F:o-acetyltransferase activity |
|  |  |  | F:small conjugating protein binding |
|  |  |  | F:xenobiotic-transporting ATPase activity |
|  |  |  | F:xenobiotic transporter activity |
|  |  |  | F:acid-thiol ligase activity |
|  |  |  | F:coA-ligase activity |
|  |  |  | F:carnitine O-acetyltransferase activity |
|  |  |  | F:carnitine O-acyltransferase activity |
|  |  |  | F:acetate-CoA ligase activity |
|  |  |  | F:3-hydroxyacyl-CoA dehydrogenase activity |
|  |  |  | F:enoyl-CoA hydratase activity |
|  |  |  | F:sUMO binding |
|  |  |  | F:sUMO polymer binding |
|  |  |  | F:aspartyl aminopeptidase activity |
|  |  |  | F:1-phosphatidylinositol 4-kinase activity |
|
| Aft2 | Ume6 | Rpn4 | Cellular Component |
|  |  |  | C:sNARE complex |
|  |  |  | C:synaptonemal complex |
|  |  |  | C:peroxisome |
|  |  |  | C:microbody |
|  |  |  | C:transverse filament |
|  |  |  | C:peroxisomal part |
|  |  |  | C:peroxisomal matrix |
|  |  |  | C:microbody part |
|
