# Supplementary material for: High-resolution analysis of condition-specific regulatory modules in Saccharomyces cerevisiae
Source: Genome Biol. 2008 Jan 3;9(1):R2. doi: 10.1186/gb-2008-9-1-r2 (PMC2395236; doi:10.1186/gb-2008-9-1-r2)
Supplement: Additional data file 11 — Matrices describing all EPMs and RMs, including lists of synergistic pairs of regulators. [file gb-2008-9-1-r2-S11.zip › htmls/C0_EPMs_matrix/EPM_13.GO_enrichment.matrix.html]

|  |  |  |  |
| --- | --- | --- | --- |
| Gcn4 | Put3 | Hsf1 | Biological Process |
|  |  |  | P:mitochondrial genome maintenance |
|  |  |  | P:negative regulation of exit from mitosis |
|  |  |  | P:catabolism |
|  |  |  | P:proline catabolism |
|  |  |  | P:cellular catabolism |
|  |  |  | P:proline catabolism to glutamate |
|  |  |  | P:response to redox state |
|  |  |  | P:nitrogen utilization |
|  |  |  | P:redox signal response |
|  |  |  | P:ornithine metabolism |
|  |  |  | P:methionine biosynthesis |
|  |  |  | P:intracellular copper ion transport |
|  |  |  | P:histone dephosphorylation |
|  |  |  | P:cellular metabolism |
|  |  |  | P:aspartate family amino acid metabolism |
|  |  |  | P:cellular biosynthesis |
|  |  |  | P:aspartate family amino acid biosynthesis |
|  |  |  | P:metabolism |
|  |  |  | P:urea cycle intermediate metabolism |
|  |  |  | P:arginine metabolism |
|  |  |  | P:primary metabolism |
|  |  |  | P:arginine biosynthesis |
|  |  |  | P:biosynthesis |
|  |  |  | P:organic acid metabolism |
|  |  |  | P:carboxylic acid metabolism |
|  |  |  | P:amine metabolism |
|  |  |  | P:amino acid and derivative metabolism |
|  |  |  | P:amino acid metabolism |
|  |  |  | P:nitrogen compound biosynthesis |
|  |  |  | P:amino acid biosynthesis |
|  |  |  | P:amine biosynthesis |
|  |  |  | P:glutamine family amino acid metabolism |
|  |  |  | P:nitrogen compound metabolism |
|  |  |  | P:glutamine family amino acid biosynthesis |
|
| Gcn4 | Put3 | Hsf1 | Molecular Function |
|  |  |  | F:carboxypeptidase activity |
|  |  |  | F:hydrolase activity, acting on carbon-nitrogen (but not peptide) bonds, in cyclic amides |
|  |  |  | F:metallocarboxypeptidase activity |
|  |  |  | F:4-aminobutyrate transaminase activity |
|  |  |  | F:aminopeptidase I activity |
|  |  |  | F:proline dehydrogenase activity |
|  |  |  | F:gly-X carboxypeptidase activity |
|  |  |  | F:allantoinase activity |
|  |  |  | F:metallopeptidase activity |
|  |  |  | F:metalloexopeptidase activity |
|  |  |  | F:exopeptidase activity |
|  |  |  | F:ornithine carbamoyltransferase activity |
|  |  |  | F:carboxyl- and carbamoyltransferase activity |
|  |  |  | F:copper ion transporter activity |
|  |  |  | F:cDP-alcohol phosphatidyltransferase activity |
|  |  |  | F:metallochaperone activity |
|  |  |  | F:oxidoreductase activity |
|  |  |  | F:dioxygenase activity |
|  |  |  | F:oxidoreductase activity, acting on single donors with incorporation of molecular oxygen, incorporation of two atoms of oxygen |
|  |  |  | F:oxidoreductase activity, acting on single donors with incorporation of molecular oxygen |
|  |  |  | F:cystathionine beta-lyase activity |
|  |  |  | F:copper chaperone activity |
|  |  |  | F:saccharopine dehydrogenase activity |
|  |  |  | F:alkaline phosphatase activity |
|  |  |  | F:histidinol-phosphate transaminase activity |
|  |  |  | F:saccharopine dehydrogenase (NAD+, L-lysine-forming) activity |
|  |  |  | F:3-hydroxyanthranilate 3,4-dioxygenase activity |
|  |  |  | F:catalytic activity |
|  |  |  | F:diacylglycerol cholinephosphotransferase activity |
|  |  |  | F:carbamoyl-phosphate synthase (glutamine-hydrolyzing) activity |
|  |  |  | F:carbamoyl-phosphate synthase activity |
|
| Gcn4 | Put3 | Hsf1 | Cellular Component |
|  |  |  | C:carbamoyl-phosphate synthase complex |
|  |  |  | C:vacuole |
|  |  |  | C:vacuole (sensu Fungi) |
|  |  |  | C:storage vacuole |
|  |  |  | C:lytic vacuole |
|
