# Supplementary material for: High-resolution analysis of condition-specific regulatory modules in Saccharomyces cerevisiae
Source: Genome Biol. 2008 Jan 3;9(1):R2. doi: 10.1186/gb-2008-9-1-r2 (PMC2395236; doi:10.1186/gb-2008-9-1-r2)
Supplement: Additional data file 11 — Matrices describing all EPMs and RMs, including lists of synergistic pairs of regulators. [file gb-2008-9-1-r2-S11.zip › htmls/C0_EPMs_matrix/EPM_14.GO_enrichment.matrix.html]

|  |  |  |  |  |  |  |  |  |  |  |  |  |  |  |  |  |  |  |  |
| --- | --- | --- | --- | --- | --- | --- | --- | --- | --- | --- | --- | --- | --- | --- | --- | --- | --- | --- | --- |
| Aft2 | Hsf1 | Ino2 | Gcn4 | Rgt1 | Rox1 | Mig1 | Msn2 | Msn4 | Ume6 | Sut1 | Pdr3 | Pdr1 | Stp1 | Nrg1 | Pho2 | Gal4 | Put3 | Skn7 | Biological Process |
|  |  |  |  |  |  |  |  |  |  |  |  |  |  |  |  |  |  |  | P:glycogen catabolism |
|  |  |  |  |  |  |  |  |  |  |  |  |  |  |  |  |  |  |  | P:glucan catabolism |
|  |  |  |  |  |  |  |  |  |  |  |  |  |  |  |  |  |  |  | P:iron-sulfur cluster assembly |
|  |  |  |  |  |  |  |  |  |  |  |  |  |  |  |  |  |  |  | P:metallo-sulfur cluster assembly |
|  |  |  |  |  |  |  |  |  |  |  |  |  |  |  |  |  |  |  | P:ornithine metabolism |
|  |  |  |  |  |  |  |  |  |  |  |  |  |  |  |  |  |  |  | P:nonprotein amino acid metabolism |
|  |  |  |  |  |  |  |  |  |  |  |  |  |  |  |  |  |  |  | P:arginine catabolism to ornithine |
|  |  |  |  |  |  |  |  |  |  |  |  |  |  |  |  |  |  |  | P:arginine catabolism |
|  |  |  |  |  |  |  |  |  |  |  |  |  |  |  |  |  |  |  | P:multidrug transport |
|  |  |  |  |  |  |  |  |  |  |  |  |  |  |  |  |  |  |  | P:regulation of nitrogen metabolism |
|  |  |  |  |  |  |  |  |  |  |  |  |  |  |  |  |  |  |  | P:microautophagy |
|  |  |  |  |  |  |  |  |  |  |  |  |  |  |  |  |  |  |  | P:regulation of nitrogen utilization |
|  |  |  |  |  |  |  |  |  |  |  |  |  |  |  |  |  |  |  | P:response to arsenic |
|  |  |  |  |  |  |  |  |  |  |  |  |  |  |  |  |  |  |  | P:cation homeostasis |
|  |  |  |  |  |  |  |  |  |  |  |  |  |  |  |  |  |  |  | P:di-, tri-valent inorganic cation homeostasis |
|  |  |  |  |  |  |  |  |  |  |  |  |  |  |  |  |  |  |  | P:metal ion homeostasis |
|  |  |  |  |  |  |  |  |  |  |  |  |  |  |  |  |  |  |  | P:mitochondrial iron ion transport |
|  |  |  |  |  |  |  |  |  |  |  |  |  |  |  |  |  |  |  | P:iron ion homeostasis |
|  |  |  |  |  |  |  |  |  |  |  |  |  |  |  |  |  |  |  | P:transition metal ion homeostasis |
|  |  |  |  |  |  |  |  |  |  |  |  |  |  |  |  |  |  |  | P:protein metabolism |
|  |  |  |  |  |  |  |  |  |  |  |  |  |  |  |  |  |  |  | P:cellular protein metabolism |
|  |  |  |  |  |  |  |  |  |  |  |  |  |  |  |  |  |  |  | P:mitochondrial transport |
|  |  |  |  |  |  |  |  |  |  |  |  |  |  |  |  |  |  |  | P:cellular macromolecule metabolism |
|  |  |  |  |  |  |  |  |  |  |  |  |  |  |  |  |  |  |  | P:protein targeting to mitochondrion |
|  |  |  |  |  |  |  |  |  |  |  |  |  |  |  |  |  |  |  | P:c-terminal protein amino acid methylation |
|  |  |  |  |  |  |  |  |  |  |  |  |  |  |  |  |  |  |  | P:regulation of sulfur metabolism |
|  |  |  |  |  |  |  |  |  |  |  |  |  |  |  |  |  |  |  | P:cytochrome bc(1) complex assembly |
|  |  |  |  |  |  |  |  |  |  |  |  |  |  |  |  |  |  |  | P:oxidative phosphorylation |
|  |  |  |  |  |  |  |  |  |  |  |  |  |  |  |  |  |  |  | P:mitochondrial electron transport, ubiquinol to cytochrome c |
|  |  |  |  |  |  |  |  |  |  |  |  |  |  |  |  |  |  |  | P:electron transport |
|  |  |  |  |  |  |  |  |  |  |  |  |  |  |  |  |  |  |  | P:aTP synthesis coupled electron transport |
|  |  |  |  |  |  |  |  |  |  |  |  |  |  |  |  |  |  |  | P:aTP synthesis coupled electron transport (sensu Eukaryota) |
|  |  |  |  |  |  |  |  |  |  |  |  |  |  |  |  |  |  |  | P:g2/M transition of mitotic cell cycle |
|  |  |  |  |  |  |  |  |  |  |  |  |  |  |  |  |  |  |  | P:glucan biosynthesis |
|  |  |  |  |  |  |  |  |  |  |  |  |  |  |  |  |  |  |  | P:polysaccharide metabolism |
|  |  |  |  |  |  |  |  |  |  |  |  |  |  |  |  |  |  |  | P:cellular polysaccharide metabolism |
|  |  |  |  |  |  |  |  |  |  |  |  |  |  |  |  |  |  |  | P:medium-chain fatty acid metabolism |
|  |  |  |  |  |  |  |  |  |  |  |  |  |  |  |  |  |  |  | P:energy reserve metabolism |
|  |  |  |  |  |  |  |  |  |  |  |  |  |  |  |  |  |  |  | P:glucan metabolism |
|  |  |  |  |  |  |  |  |  |  |  |  |  |  |  |  |  |  |  | P:carbohydrate metabolism |
|  |  |  |  |  |  |  |  |  |  |  |  |  |  |  |  |  |  |  | P:regulation of carbohydrate metabolism |
|  |  |  |  |  |  |  |  |  |  |  |  |  |  |  |  |  |  |  | P:glycogen metabolism |
|  |  |  |  |  |  |  |  |  |  |  |  |  |  |  |  |  |  |  | P:cellular carbohydrate metabolism |
|  |  |  |  |  |  |  |  |  |  |  |  |  |  |  |  |  |  |  | P:glycogen biosynthesis |
|  |  |  |  |  |  |  |  |  |  |  |  |  |  |  |  |  |  |  | P:energy derivation by oxidation of organic compounds |
|  |  |  |  |  |  |  |  |  |  |  |  |  |  |  |  |  |  |  | P:generation of precursor metabolites and energy |
|  |  |  |  |  |  |  |  |  |  |  |  |  |  |  |  |  |  |  | P:hexose biosynthesis |
|  |  |  |  |  |  |  |  |  |  |  |  |  |  |  |  |  |  |  | P:gluconeogenesis |
|  |  |  |  |  |  |  |  |  |  |  |  |  |  |  |  |  |  |  | P:monosaccharide biosynthesis |
|  |  |  |  |  |  |  |  |  |  |  |  |  |  |  |  |  |  |  | P:guanine metabolism |
|  |  |  |  |  |  |  |  |  |  |  |  |  |  |  |  |  |  |  | P:polyamine transport |
|  |  |  |  |  |  |  |  |  |  |  |  |  |  |  |  |  |  |  | P:carbohydrate biosynthesis |
|  |  |  |  |  |  |  |  |  |  |  |  |  |  |  |  |  |  |  | P:regulation of carbohydrate biosynthesis |
|  |  |  |  |  |  |  |  |  |  |  |  |  |  |  |  |  |  |  | P:regulation of gluconeogenesis |
|  |  |  |  |  |  |  |  |  |  |  |  |  |  |  |  |  |  |  | P:negative regulation of gluconeogenesis |
|  |  |  |  |  |  |  |  |  |  |  |  |  |  |  |  |  |  |  | P:negative regulation of carbohydrate metabolism |
|  |  |  |  |  |  |  |  |  |  |  |  |  |  |  |  |  |  |  | P:hexose metabolism |
|  |  |  |  |  |  |  |  |  |  |  |  |  |  |  |  |  |  |  | P:main pathways of carbohydrate metabolism |
|  |  |  |  |  |  |  |  |  |  |  |  |  |  |  |  |  |  |  | P:monosaccharide metabolism |
|  |  |  |  |  |  |  |  |  |  |  |  |  |  |  |  |  |  |  | P:glucose metabolism |
|  |  |  |  |  |  |  |  |  |  |  |  |  |  |  |  |  |  |  | P:amine transport |
|  |  |  |  |  |  |  |  |  |  |  |  |  |  |  |  |  |  |  | P:vacuolar protein catabolism |
|  |  |  |  |  |  |  |  |  |  |  |  |  |  |  |  |  |  |  | P:rhythmic process |
|  |  |  |  |  |  |  |  |  |  |  |  |  |  |  |  |  |  |  | P:ultradian rhythm |
|  |  |  |  |  |  |  |  |  |  |  |  |  |  |  |  |  |  |  | P:posttranslational protein folding |
|  |  |  |  |  |  |  |  |  |  |  |  |  |  |  |  |  |  |  | P:chaperone cofactor-dependent protein folding |
|  |  |  |  |  |  |  |  |  |  |  |  |  |  |  |  |  |  |  | P:protein import into nucleus, translocation |
|  |  |  |  |  |  |  |  |  |  |  |  |  |  |  |  |  |  |  | P:response to stimulus |
|  |  |  |  |  |  |  |  |  |  |  |  |  |  |  |  |  |  |  | P:protein refolding |
|  |  |  |  |  |  |  |  |  |  |  |  |  |  |  |  |  |  |  | P:protein folding |
|  |  |  |  |  |  |  |  |  |  |  |  |  |  |  |  |  |  |  | P:response to stress |
|  |  |  |  |  |  |  |  |  |  |  |  |  |  |  |  |  |  |  | P:biological\_process |
|  |  |  |  |  |  |  |  |  |  |  |  |  |  |  |  |  |  |  | P:alcohol biosynthesis |
|  |  |  |  |  |  |  |  |  |  |  |  |  |  |  |  |  |  |  | P:pyruvate metabolism |
|
| Aft2 | Hsf1 | Ino2 | Gcn4 | Rgt1 | Rox1 | Mig1 | Msn2 | Msn4 | Ume6 | Sut1 | Pdr3 | Pdr1 | Stp1 | Nrg1 | Pho2 | Gal4 | Put3 | Skn7 | Molecular Function |
|  |  |  |  |  |  |  |  |  |  |  |  |  |  |  |  |  |  |  | F:iron ion transporter activity |
|  |  |  |  |  |  |  |  |  |  |  |  |  |  |  |  |  |  |  | F:arginase activity |
|  |  |  |  |  |  |  |  |  |  |  |  |  |  |  |  |  |  |  | F:manganese ion binding |
|  |  |  |  |  |  |  |  |  |  |  |  |  |  |  |  |  |  |  | F:transition metal ion binding |
|  |  |  |  |  |  |  |  |  |  |  |  |  |  |  |  |  |  |  | F:hydrolase activity, acting on carbon-nitrogen (but not peptide) bonds, in linear amidines |
|  |  |  |  |  |  |  |  |  |  |  |  |  |  |  |  |  |  |  | F:zinc ion binding |
|  |  |  |  |  |  |  |  |  |  |  |  |  |  |  |  |  |  |  | F:ion transporter activity |
|  |  |  |  |  |  |  |  |  |  |  |  |  |  |  |  |  |  |  | F:dNA binding |
|  |  |  |  |  |  |  |  |  |  |  |  |  |  |  |  |  |  |  | F:cation transporter activity |
|  |  |  |  |  |  |  |  |  |  |  |  |  |  |  |  |  |  |  | F:transcription factor activity |
|  |  |  |  |  |  |  |  |  |  |  |  |  |  |  |  |  |  |  | F:monovalent inorganic cation transporter activity |
|  |  |  |  |  |  |  |  |  |  |  |  |  |  |  |  |  |  |  | F:hydrogen ion transporter activity |
|  |  |  |  |  |  |  |  |  |  |  |  |  |  |  |  |  |  |  | F:ubiquinol-cytochrome-c reductase activity |
|  |  |  |  |  |  |  |  |  |  |  |  |  |  |  |  |  |  |  | F:oxidoreductase activity, acting on diphenols and related substances as donors |
|  |  |  |  |  |  |  |  |  |  |  |  |  |  |  |  |  |  |  | F:oxidoreductase activity, acting on diphenols and related substances as donors, cytochrome as acceptor |
|  |  |  |  |  |  |  |  |  |  |  |  |  |  |  |  |  |  |  | F:cytochrome-c peroxidase activity |
|  |  |  |  |  |  |  |  |  |  |  |  |  |  |  |  |  |  |  | F:1,4-alpha-glucan branching enzyme activity |
|  |  |  |  |  |  |  |  |  |  |  |  |  |  |  |  |  |  |  | F:binding |
|  |  |  |  |  |  |  |  |  |  |  |  |  |  |  |  |  |  |  | F:chaperone regulator activity |
|  |  |  |  |  |  |  |  |  |  |  |  |  |  |  |  |  |  |  | F:hsp70/Hsc70 protein regulator activity |
|  |  |  |  |  |  |  |  |  |  |  |  |  |  |  |  |  |  |  | F:molecular\_function |
|  |  |  |  |  |  |  |  |  |  |  |  |  |  |  |  |  |  |  | F:uDP-glycosyltransferase activity |
|  |  |  |  |  |  |  |  |  |  |  |  |  |  |  |  |  |  |  | F:glucosyltransferase activity |
|  |  |  |  |  |  |  |  |  |  |  |  |  |  |  |  |  |  |  | F:unfolded protein binding |
|  |  |  |  |  |  |  |  |  |  |  |  |  |  |  |  |  |  |  | F:uDP-glucosyltransferase activity |
|  |  |  |  |  |  |  |  |  |  |  |  |  |  |  |  |  |  |  | F:chaperone binding |
|  |  |  |  |  |  |  |  |  |  |  |  |  |  |  |  |  |  |  | F:protein binding |
|  |  |  |  |  |  |  |  |  |  |  |  |  |  |  |  |  |  |  | F:polyamine transporter activity |
|  |  |  |  |  |  |  |  |  |  |  |  |  |  |  |  |  |  |  | F:guanine deaminase activity |
|  |  |  |  |  |  |  |  |  |  |  |  |  |  |  |  |  |  |  | F:d-lactaldehyde dehydrogenase activity |
|  |  |  |  |  |  |  |  |  |  |  |  |  |  |  |  |  |  |  | F:oxidoreductase activity, acting on peroxide as acceptor |
|  |  |  |  |  |  |  |  |  |  |  |  |  |  |  |  |  |  |  | F:peroxidase activity |
|  |  |  |  |  |  |  |  |  |  |  |  |  |  |  |  |  |  |  | F:glutathione disulfide oxidoreductase activity |
|  |  |  |  |  |  |  |  |  |  |  |  |  |  |  |  |  |  |  | F:peptide disulfide oxidoreductase activity |
|  |  |  |  |  |  |  |  |  |  |  |  |  |  |  |  |  |  |  | F:glutathione-disulfide reductase activity |
|  |  |  |  |  |  |  |  |  |  |  |  |  |  |  |  |  |  |  | F:hydrolase activity |
|  |  |  |  |  |  |  |  |  |  |  |  |  |  |  |  |  |  |  | F:oxidoreductase activity |
|  |  |  |  |  |  |  |  |  |  |  |  |  |  |  |  |  |  |  | F:antioxidant activity |
|  |  |  |  |  |  |  |  |  |  |  |  |  |  |  |  |  |  |  | F:aldo-keto reductase activity |
|  |  |  |  |  |  |  |  |  |  |  |  |  |  |  |  |  |  |  | F:c-terminal protein carboxyl methyltransferase activity |
|  |  |  |  |  |  |  |  |  |  |  |  |  |  |  |  |  |  |  | F:alpha-glucosidase activity |
|  |  |  |  |  |  |  |  |  |  |  |  |  |  |  |  |  |  |  | F:transcriptional activator activity |
|  |  |  |  |  |  |  |  |  |  |  |  |  |  |  |  |  |  |  | F:transcription regulator activity |
|  |  |  |  |  |  |  |  |  |  |  |  |  |  |  |  |  |  |  | F:ceramidase activity |
|  |  |  |  |  |  |  |  |  |  |  |  |  |  |  |  |  |  |  | F:glycogen (starch) synthase activity |
|  |  |  |  |  |  |  |  |  |  |  |  |  |  |  |  |  |  |  | F:spermidine transporter activity |
|  |  |  |  |  |  |  |  |  |  |  |  |  |  |  |  |  |  |  | F:carbonate dehydratase activity |
|  |  |  |  |  |  |  |  |  |  |  |  |  |  |  |  |  |  |  | F:glucosidase activity |
|  |  |  |  |  |  |  |  |  |  |  |  |  |  |  |  |  |  |  | F:glucan 1,4-alpha-glucosidase activity |
|  |  |  |  |  |  |  |  |  |  |  |  |  |  |  |  |  |  |  | F:hydrolase activity, hydrolyzing O-glycosyl compounds |
|  |  |  |  |  |  |  |  |  |  |  |  |  |  |  |  |  |  |  | F:hydrolase activity, acting on glycosyl bonds |
|
| Aft2 | Hsf1 | Ino2 | Gcn4 | Rgt1 | Rox1 | Mig1 | Msn2 | Msn4 | Ume6 | Sut1 | Pdr3 | Pdr1 | Stp1 | Nrg1 | Pho2 | Gal4 | Put3 | Skn7 | Cellular Component |
|  |  |  |  |  |  |  |  |  |  |  |  |  |  |  |  |  |  |  | C:cyclin-dependent protein kinase holoenzyme complex |
|  |  |  |  |  |  |  |  |  |  |  |  |  |  |  |  |  |  |  | C:cCAAT-binding factor complex |
|  |  |  |  |  |  |  |  |  |  |  |  |  |  |  |  |  |  |  | C:cytosol |
|  |  |  |  |  |  |  |  |  |  |  |  |  |  |  |  |  |  |  | C:membrane-bound organelle |
|  |  |  |  |  |  |  |  |  |  |  |  |  |  |  |  |  |  |  | C:intracellular membrane-bound organelle |
|  |  |  |  |  |  |  |  |  |  |  |  |  |  |  |  |  |  |  | C:ubiquinol-cytochrome-c reductase complex |
|  |  |  |  |  |  |  |  |  |  |  |  |  |  |  |  |  |  |  | C:respiratory chain complex III |
|  |  |  |  |  |  |  |  |  |  |  |  |  |  |  |  |  |  |  | C:mitochondrial electron transport chain |
|  |  |  |  |  |  |  |  |  |  |  |  |  |  |  |  |  |  |  | C:respiratory chain complex III (sensu Eukaryota) |
|  |  |  |  |  |  |  |  |  |  |  |  |  |  |  |  |  |  |  | C:intracellular |
|  |  |  |  |  |  |  |  |  |  |  |  |  |  |  |  |  |  |  | C:cytoplasm |
|  |  |  |  |  |  |  |  |  |  |  |  |  |  |  |  |  |  |  | C:intracellular part |
|  |  |  |  |  |  |  |  |  |  |  |  |  |  |  |  |  |  |  | C:vacuole (sensu Fungi) |
|  |  |  |  |  |  |  |  |  |  |  |  |  |  |  |  |  |  |  | C:lytic vacuole |
|  |  |  |  |  |  |  |  |  |  |  |  |  |  |  |  |  |  |  | C:vacuole |
|  |  |  |  |  |  |  |  |  |  |  |  |  |  |  |  |  |  |  | C:storage vacuole |
|  |  |  |  |  |  |  |  |  |  |  |  |  |  |  |  |  |  |  | C:cell part |
|  |  |  |  |  |  |  |  |  |  |  |  |  |  |  |  |  |  |  | C:cell |
|  |  |  |  |  |  |  |  |  |  |  |  |  |  |  |  |  |  |  | C:chaperonin-containing T-complex |
|  |  |  |  |  |  |  |  |  |  |  |  |  |  |  |  |  |  |  | C:cellular\_component |
|  |  |  |  |  |  |  |  |  |  |  |  |  |  |  |  |  |  |  | C:vacuolar lumen |
|  |  |  |  |  |  |  |  |  |  |  |  |  |  |  |  |  |  |  | C:vacuolar part |
|  |  |  |  |  |  |  |  |  |  |  |  |  |  |  |  |  |  |  | C:vacuolar lumen (sensu Fungi) |
|
