# Supplementary material for: High-resolution analysis of condition-specific regulatory modules in Saccharomyces cerevisiae
Source: Genome Biol. 2008 Jan 3;9(1):R2. doi: 10.1186/gb-2008-9-1-r2 (PMC2395236; doi:10.1186/gb-2008-9-1-r2)
Supplement: Additional data file 11 — Matrices describing all EPMs and RMs, including lists of synergistic pairs of regulators. [file gb-2008-9-1-r2-S11.zip › htmls/C0_EPMs_matrix/EPM_14.Overlap.matrix.html]

|  |  |  |  |  |  |  |  |  |  |  |  |  |  |  |  |  |  |  |
| --- | --- | --- | --- | --- | --- | --- | --- | --- | --- | --- | --- | --- | --- | --- | --- | --- | --- | --- |
| Aft2 | Hsf1 | Ino2 | Gcn4 | Rgt1 | Rox1 | Mig1 | Msn2 | Msn4 | Ume6 | Sut1 | Pdr3 | Pdr1 | Stp1 | Nrg1 | Pho2 | Gal4 | Put3 | Skn7 |
|  |  |  |  |  |  |  |  |  |  |  |  |  |  |  |  |  |  |  | Aft2 |
|  |  |  |  |  |  |  |  |  |  |  |  |  |  |  |  |  |  |  | Hsf1 |
|  |  |  |  |  |  |  |  |  |  |  |  |  |  |  |  |  |  |  | Ino2 |
|  |  |  |  |  |  |  |  |  |  |  |  |  |  |  |  |  |  |  | Gcn4 |
|  |  |  |  |  |  |  |  |  |  |  |  |  |  |  |  |  |  |  | Rgt1 |
|  |  |  |  |  |  |  |  |  |  |  |  |  |  |  |  |  |  |  | Rox1 |
|  |  |  |  |  |  |  |  |  |  |  |  |  |  |  |  |  |  |  | Mig1 |
|  |  |  |  |  |  |  |  |  |  |  |  |  |  |  |  |  |  |  | Msn2 |
|  |  |  |  |  |  |  |  |  |  |  |  |  |  |  |  |  |  |  | Msn4 |
|  |  |  |  |  |  |  |  |  |  |  |  |  |  |  |  |  |  |  | Ume6 |
|  |  |  |  |  |  |  |  |  |  |  |  |  |  |  |  |  |  |  | Sut1 |
|  |  |  |  |  |  |  |  |  |  |  |  |  |  |  |  |  |  |  | Pdr3 |
|  |  |  |  |  |  |  |  |  |  |  |  |  |  |  |  |  |  |  | Pdr1 |
|  |  |  |  |  |  |  |  |  |  |  |  |  |  |  |  |  |  |  | Stp1 |
|  |  |  |  |  |  |  |  |  |  |  |  |  |  |  |  |  |  |  | Nrg1 |
|  |  |  |  |  |  |  |  |  |  |  |  |  |  |  |  |  |  |  | Pho2 |
|  |  |  |  |  |  |  |  |  |  |  |  |  |  |  |  |  |  |  | Gal4 |
|  |  |  |  |  |  |  |  |  |  |  |  |  |  |  |  |  |  |  | Put3 |
|  |  |  |  |  |  |  |  |  |  |  |  |  |  |  |  |  |  |  | Skn7 |
 Aft2 | Hsf1 | Ino2 | Gcn4 | Rgt1 | Rox1 | Mig1 | Msn2 | Msn4 | Ume6 | Sut1 | Pdr3 | Pdr1 | Stp1 | Nrg1 | Pho2 | Gal4 | Put3 | Skn7 |
