# Supplementary material for: High-resolution analysis of condition-specific regulatory modules in Saccharomyces cerevisiae
Source: Genome Biol. 2008 Jan 3;9(1):R2. doi: 10.1186/gb-2008-9-1-r2 (PMC2395236; doi:10.1186/gb-2008-9-1-r2)
Supplement: Additional data file 11 — Matrices describing all EPMs and RMs, including lists of synergistic pairs of regulators. [file gb-2008-9-1-r2-S11.zip › htmls/C0_EPMs_matrix/EPM_14.RM.matrix.html]

Regulators vs. RM target gene list

|  |  |  |  |  |  |  |  |  |  |  |  |  |  |  |  |  |  |  |  |
| --- | --- | --- | --- | --- | --- | --- | --- | --- | --- | --- | --- | --- | --- | --- | --- | --- | --- | --- | --- |
|  | Aft2 | Hsf1 | Ino2 | Gcn4 | Rgt1 | Rox1 | Mig1 | Msn2 | Msn4 | Ume6 | Sut1 | Pdr3 | Pdr1 | Stp1 | Nrg1 | Pho2 | Gal4 | Put3 | Skn7 |
| RM\_1 |  |  |  |  |  |  |  |  |  |  |  |  |  |  |  |  |  |  |  |
| RM\_2 |  |  |  |  |  |  |  |  |  |  |  |  |  |  |  |  |  |  |  |
| RM\_3 |  |  |  |  |  |  |  |  |  |  |  |  |  |  |  |  |  |  |  |
| RM\_4 |  |  |  |  |  |  |  |  |  |  |  |  |  |  |  |  |  |  |  |
| RM\_5 |  |  |  |  |  |  |  |  |  |  |  |  |  |  |  |  |  |  |  |
| RM\_6 |  |  |  |  |  |  |  |  |  |  |  |  |  |  |  |  |  |  |  |
| RM\_7 |  |  |  |  |  |  |  |  |  |  |  |  |  |  |  |  |  |  |  |
| RM\_8 |  |  |  |  |  |  |  |  |  |  |  |  |  |  |  |  |  |  |  |
| RM\_9 |  |  |  |  |  |  |  |  |  |  |  |  |  |  |  |  |  |  |  |
| RM\_10 |  |  |  |  |  |  |  |  |  |  |  |  |  |  |  |  |  |  |  |

Synergistic Pair of Regulators

1. Gcn4\*Rox1

2. Msn4\*Put3

3. Msn4\*Pho2

4. Msn2\*Put3

5. Msn4\*Sut1

6. Msn4\*Ume6

7. Msn4\*Skn7

8. Msn2\*Pho2

9. Msn2\*Msn4

10. Msn2\*Skn7

11. Msn4\*Pdr1

12. Msn4\*Pdr3

13. Msn2\*Sut1

14. Put3\*Sut1

15. Msn2\*Ume6

16. Rgt1\*Sut1

17. Msn2\*Pdr3

18. Pdr3\*Rgt1

19. Pdr3\*Put3

20. Pdr1\*Rgt1

21. Pdr1\*Put3

22. Skn7\*Sut1

23. Mig1\*Sut1

24. Mig1\*Rgt1

25. Pdr3\*Sut1

26. Hsf1\*Msn2

27. Put3\*Skn7

28. Skn7\*Stp1

29. Pdr1\*Pdr3

30. Pdr1\*Sut1

Matrix of enriched GO

EPM matrix
