# Supplementary material for: High-resolution analysis of condition-specific regulatory modules in Saccharomyces cerevisiae
Source: Genome Biol. 2008 Jan 3;9(1):R2. doi: 10.1186/gb-2008-9-1-r2 (PMC2395236; doi:10.1186/gb-2008-9-1-r2)
Supplement: Additional data file 11 — Matrices describing all EPMs and RMs, including lists of synergistic pairs of regulators. [file gb-2008-9-1-r2-S11.zip › htmls/C0_EPMs_matrix/EPM_2.GO_enrichment.matrix.html]

|  |  |  |  |  |  |
| --- | --- | --- | --- | --- | --- |
| Mcm1 | Abf1 | Tec1 | Swi4 | Swi6 | Biological Process |
|  |  |  |  |  | P:regulation of initiation of mating projection growth |
|  |  |  |  |  | P:secretory pathway |
|  |  |  |  |  | P:secretion |
|  |  |  |  |  | P:cell projection organization and biogenesis |
|  |  |  |  |  | P:cell projection biogenesis |
|  |  |  |  |  | P:agglutination during conjugation with cellular fusion |
|  |  |  |  |  | P:agglutination |
|  |  |  |  |  | P:peptide transport |
|  |  |  |  |  | P:heterotrimeric G-protein complex cycle |
|  |  |  |  |  | P:reproduction |
|  |  |  |  |  | P:peptide pheromone export |
|  |  |  |  |  | P:response to chemical stimulus |
|  |  |  |  |  | P:reproductive cellular physiological process |
|  |  |  |  |  | P:reproductive physiological process |
|  |  |  |  |  | P:interaction between organisms |
|  |  |  |  |  | P:conjugation with cellular fusion |
|  |  |  |  |  | P:conjugation |
|  |  |  |  |  | P:sexual reproduction |
|  |  |  |  |  | P:response to pheromone during conjugation with cellular fusion |
|  |  |  |  |  | P:response to pheromone |
|  |  |  |  |  | P:cell wall glycoprotein biosynthesis |
|  |  |  |  |  | P:mannoprotein biosynthesis |
|  |  |  |  |  | P:mannoprotein metabolism |
|  |  |  |  |  | P:cell wall mannoprotein biosynthesis |
|  |  |  |  |  | P:cell wall organization and biogenesis |
|  |  |  |  |  | P:external encapsulating structure organization and biogenesis |
|  |  |  |  |  | P:cell division |
|  |  |  |  |  | P:protein retention in ER |
|  |  |  |  |  | P:plasmid maintenance |
|  |  |  |  |  | P:cell budding |
|  |  |  |  |  | P:asexual reproduction |
|  |  |  |  |  | P:macroautophagy |
|  |  |  |  |  | P:autophagic vacuole fusion |
|  |  |  |  |  | P:non-developmental growth |
|  |  |  |  |  | P:budding cell bud growth |
|
| Mcm1 | Abf1 | Tec1 | Swi4 | Swi6 | Molecular Function |
|  |  |  |  |  | F:dNA-directed RNA polymerase activity |
|  |  |  |  |  | F:glycogen synthase kinase 3 activity |
|  |  |  |  |  | F:microfilament motor activity |
|  |  |  |  |  | F:alpha-1,2-mannosyltransferase activity |
|  |  |  |  |  | F:mannosyltransferase activity |
|  |  |  |  |  | F:transferase activity, transferring hexosyl groups |
|  |  |  |  |  | F:transmembrane receptor activity |
|  |  |  |  |  | F:cell adhesion molecule binding |
|  |  |  |  |  | F:g-protein coupled receptor activity |
|  |  |  |  |  | F:mating-type factor pheromone receptor activity |
|  |  |  |  |  | F:mating-type alpha-factor pheromone receptor activity |
|  |  |  |  |  | F:pheromone receptor activity |
|
| Mcm1 | Abf1 | Tec1 | Swi4 | Swi6 | Cellular Component |
|  |  |  |  |  | C:translocon complex |
|  |  |  |  |  | C:soluble fraction |
|  |  |  |  |  | C:endoplasmic reticulum part |
|  |  |  |  |  | C:nuclear envelope-endoplasmic reticulum network |
|  |  |  |  |  | C:endoplasmic reticulum membrane |
|  |  |  |  |  | C:rNA polymerase complex |
|  |  |  |  |  | C:bud neck septin structure |
|  |  |  |  |  | C:bud neck septin ring |
|  |  |  |  |  | C:cleavage apparatus septin structure |
|  |  |  |  |  | C:external encapsulating structure |
|  |  |  |  |  | C:cell wall |
|  |  |  |  |  | C:cell wall (sensu Fungi) |
|  |  |  |  |  | C:heterotrimeric G-protein complex |
|  |  |  |  |  | C:plasma membrane |
|  |  |  |  |  | C:intrinsic to plasma membrane |
|  |  |  |  |  | C:plasma membrane part |
|  |  |  |  |  | C:integral to plasma membrane |
|  |  |  |  |  | C:cyclin-dependent protein kinase holoenzyme complex |
|  |  |  |  |  | C:bud neck |
|  |  |  |  |  | C:bud |
|  |  |  |  |  | C:actin cable |
|  |  |  |  |  | C:site of polarized growth |
|  |  |  |  |  | C:cell wall part |
|  |  |  |  |  | C:bud scar |
|  |  |  |  |  | C:external encapsulating structure part |
|
