# Supplementary material for: High-resolution analysis of condition-specific regulatory modules in Saccharomyces cerevisiae
Source: Genome Biol. 2008 Jan 3;9(1):R2. doi: 10.1186/gb-2008-9-1-r2 (PMC2395236; doi:10.1186/gb-2008-9-1-r2)
Supplement: Additional data file 11 — Matrices describing all EPMs and RMs, including lists of synergistic pairs of regulators. [file gb-2008-9-1-r2-S11.zip › htmls/C0_EPMs_matrix/EPM_5.GO_enrichment.matrix.html]

|  |  |  |  |
| --- | --- | --- | --- |
| Yap1 | Fhl1 | Mcm1 | Biological Process |
|  |  |  | P:protein metabolism |
|  |  |  | P:regulation of translational fidelity |
|  |  |  | P:cellular macromolecule metabolism |
|  |  |  | P:metabolism |
|  |  |  | P:cellular protein metabolism |
|  |  |  | P:organic cation transport |
|  |  |  | P:ammonium transport |
|  |  |  | P:biosynthesis |
|  |  |  | P:cellular biosynthesis |
|  |  |  | P:ribosome biogenesis and assembly |
|  |  |  | P:cytoplasm organization and biogenesis |
|  |  |  | P:macromolecule biosynthesis |
|  |  |  | P:ribosomal large subunit assembly and maintenance |
|  |  |  | P:protein biosynthesis |
|  |  |  | P:protein complex assembly |
|  |  |  | P:translational elongation |
|  |  |  | P:translation |
|  |  |  | P:ribosomal subunit assembly |
|  |  |  | P:ribosome assembly |
|  |  |  | P:drug transport |
|  |  |  | P:intracellular copper ion transport |
|  |  |  | P:copper ion transport |
|
| Yap1 | Fhl1 | Mcm1 | Molecular Function |
|  |  |  | F:mating pheromone activity |
|  |  |  | F:receptor binding |
|  |  |  | F:pyruvate kinase activity |
|  |  |  | F:pheromone activity |
|  |  |  | F:ammonium transporter activity |
|  |  |  | F:organic cation transporter activity |
|  |  |  | F:structural constituent of ribosome |
|  |  |  | F:structural molecule activity |
|  |  |  | F:copper ion transporter activity |
|  |  |  | F:metallochaperone activity |
|  |  |  | F:copper chaperone activity |
|  |  |  | F:aromatic-amino-acid transaminase activity |
|  |  |  | F:nitric oxide reductase activity |
|  |  |  | F:superoxide dismutase copper chaperone activity |
|  |  |  | F:oxidoreductase activity, acting on other nitrogenous compounds as donors |
|
| Yap1 | Fhl1 | Mcm1 | Cellular Component |
|  |  |  | C:intracellular non-membrane-bound organelle |
|  |  |  | C:non-membrane-bound organelle |
|  |  |  | C:protein complex |
|  |  |  | C:ribonucleoprotein complex |
|  |  |  | C:large ribosomal subunit |
|  |  |  | C:cytosol |
|  |  |  | C:cytosolic large ribosomal subunit (sensu Eukaryota) |
|  |  |  | C:ribosome |
|  |  |  | C:cytosolic ribosome (sensu Eukaryota) |
|  |  |  | C:cytosolic part |
|  |  |  | C:site of polarized growth |
|  |  |  | C:bud neck |
|  |  |  | C:bud |
|
