# Supplementary material for: High-resolution analysis of condition-specific regulatory modules in Saccharomyces cerevisiae
Source: Genome Biol. 2008 Jan 3;9(1):R2. doi: 10.1186/gb-2008-9-1-r2 (PMC2395236; doi:10.1186/gb-2008-9-1-r2)
Supplement: Additional data file 11 — Matrices describing all EPMs and RMs, including lists of synergistic pairs of regulators. [file gb-2008-9-1-r2-S11.zip › htmls/C0_EPMs_matrix/EPM_6.GO_enrichment.matrix.html]

|  |  |  |  |  |  |  |  |
| --- | --- | --- | --- | --- | --- | --- | --- |
| Mbp1 | Pdr1 | Hsf1 | Sfp1 | Hir2 | Hir1 | Hir3 | Biological Process |
|  |  |  |  |  |  |  | P:dolichol-linked oligosaccharide biosynthesis |
|  |  |  |  |  |  |  | P:dNA repair |
|  |  |  |  |  |  |  | P:biopolymer metabolism |
|  |  |  |  |  |  |  | P:chromosome organization and biogenesis |
|  |  |  |  |  |  |  | P:dNA metabolism |
|  |  |  |  |  |  |  | P:chromosome organization and biogenesis (sensu Eukaryota) |
|  |  |  |  |  |  |  | P:establishment and/or maintenance of chromatin architecture |
|  |  |  |  |  |  |  | P:chromatin assembly or disassembly |
|  |  |  |  |  |  |  | P:dNA packaging |
|  |  |  |  |  |  |  | P:response to endogenous stimulus |
|  |  |  |  |  |  |  | P:response to DNA damage stimulus |
|  |  |  |  |  |  |  | P:macromolecule metabolism |
|  |  |  |  |  |  |  | P:cell organization and biogenesis |
|  |  |  |  |  |  |  | P:organelle organization and biogenesis |
|  |  |  |  |  |  |  | P:nucleobase, nucleoside, nucleotide and nucleic acid metabolism |
|
| Mbp1 | Pdr1 | Hsf1 | Sfp1 | Hir2 | Hir1 | Hir3 | Molecular Function |
|  |  |  |  |  |  |  | F:binding |
|  |  |  |  |  |  |  | F:dNA binding |
|  |  |  |  |  |  |  | F:nucleic acid binding |
|  |  |  |  |  |  |  | F:acetylglucosaminyltransferase activity |
|  |  |  |  |  |  |  | F:1,3-beta-glucanosyltransferase activity |
|  |  |  |  |  |  |  | F:glucanosyltransferase activity |
|  |  |  |  |  |  |  | F:epsilon DNA polymerase activity |
|  |  |  |  |  |  |  | F:protein anchor |
|  |  |  |  |  |  |  | F:n-acetylglucosaminyldiphosphodolichol N-acetylglucosaminyltransferase activity |
|
| Mbp1 | Pdr1 | Hsf1 | Sfp1 | Hir2 | Hir1 | Hir3 | Cellular Component |
|  |  |  |  |  |  |  | C:dNA polymerase complex |
|  |  |  |  |  |  |  | C:uDP-N-acetylglucosamine transferase complex |
|  |  |  |  |  |  |  | C:epsilon DNA polymerase complex |
|  |  |  |  |  |  |  | C:protein complex |
|  |  |  |  |  |  |  | C:nuclear part |
|  |  |  |  |  |  |  | C:intracellular non-membrane-bound organelle |
|  |  |  |  |  |  |  | C:non-membrane-bound organelle |
|  |  |  |  |  |  |  | C:chromosome |
|  |  |  |  |  |  |  | C:chromosomal part |
|  |  |  |  |  |  |  | C:nuclear chromosome |
|  |  |  |  |  |  |  | C:nuclear chromosome part |
|  |  |  |  |  |  |  | C:chromatin |
|  |  |  |  |  |  |  | C:nuclear chromatin |
|  |  |  |  |  |  |  | C:nuclear nucleosome |
|  |  |  |  |  |  |  | C:nucleosome |
|  |  |  |  |  |  |  | C:organelle part |
|  |  |  |  |  |  |  | C:nucleus |
|  |  |  |  |  |  |  | C:intracellular organelle part |
|
