# Supplementary material for: High-resolution analysis of condition-specific regulatory modules in Saccharomyces cerevisiae
Source: Genome Biol. 2008 Jan 3;9(1):R2. doi: 10.1186/gb-2008-9-1-r2 (PMC2395236; doi:10.1186/gb-2008-9-1-r2)
Supplement: Additional data file 11 — Matrices describing all EPMs and RMs, including lists of synergistic pairs of regulators. [file gb-2008-9-1-r2-S11.zip › htmls/C0_EPMs_matrix/EPM_6.RM.matrix.html]

Regulators vs. RM target gene list

|  |  |  |  |  |  |  |  |
| --- | --- | --- | --- | --- | --- | --- | --- |
|  | Mbp1 | Pdr1 | Hsf1 | Sfp1 | Hir2 | Hir1 | Hir3 |
| RM\_1 |  |  |  |  |  |  |  |
| RM\_2 |  |  |  |  |  |  |  |

Synergistic Pair of Regulators

1. Hir1\*Hir3

2. Hir1\*Hir2

3. Hir2\*Hir3

4. Hsf1\*Pdr1

Matrix of enriched GO

EPM matrix
