# Supplementary material for: High-resolution analysis of condition-specific regulatory modules in Saccharomyces cerevisiae
Source: Genome Biol. 2008 Jan 3;9(1):R2. doi: 10.1186/gb-2008-9-1-r2 (PMC2395236; doi:10.1186/gb-2008-9-1-r2)
Supplement: Additional data file 11 — Matrices describing all EPMs and RMs, including lists of synergistic pairs of regulators. [file gb-2008-9-1-r2-S11.zip › htmls/C0_EPMs_matrix/EPM_7.GO_enrichment.matrix.html]

|  |  |  |  |  |  |  |  |  |  |  |  |  |  |
| --- | --- | --- | --- | --- | --- | --- | --- | --- | --- | --- | --- | --- | --- |
| Pho2 | Skn7 | Sfp1 | Fhl1 | Rap1 | Reb1 | Azf1 | Tec1 | Mbp1 | Swi4 | Swi6 | Ste12 | Stb1 | Biological Process |
|  |  |  |  |  |  |  |  |  |  |  |  |  | P:cell organization and biogenesis |
|  |  |  |  |  |  |  |  |  |  |  |  |  | P:ribosomal small subunit biogenesis |
|  |  |  |  |  |  |  |  |  |  |  |  |  | P:translational elongation |
|  |  |  |  |  |  |  |  |  |  |  |  |  | P:ribosome export from nucleus |
|  |  |  |  |  |  |  |  |  |  |  |  |  | P:macromolecule metabolism |
|  |  |  |  |  |  |  |  |  |  |  |  |  | P:protein biosynthesis |
|  |  |  |  |  |  |  |  |  |  |  |  |  | P:translation |
|  |  |  |  |  |  |  |  |  |  |  |  |  | P:biosynthesis |
|  |  |  |  |  |  |  |  |  |  |  |  |  | P:negative regulation of protein biosynthesis |
|  |  |  |  |  |  |  |  |  |  |  |  |  | P:negative regulation of biosynthesis |
|  |  |  |  |  |  |  |  |  |  |  |  |  | P:negative regulation of translation |
|  |  |  |  |  |  |  |  |  |  |  |  |  | P:negative regulation of cellular biosynthesis |
|  |  |  |  |  |  |  |  |  |  |  |  |  | P:chromosome organization and biogenesis |
|  |  |  |  |  |  |  |  |  |  |  |  |  | P:chromosome organization and biogenesis (sensu Eukaryota) |
|  |  |  |  |  |  |  |  |  |  |  |  |  | P:ribosomal small subunit export from nucleus |
|  |  |  |  |  |  |  |  |  |  |  |  |  | P:organelle organization and biogenesis |
|  |  |  |  |  |  |  |  |  |  |  |  |  | P:regulation of biosynthesis |
|  |  |  |  |  |  |  |  |  |  |  |  |  | P:regulation of cellular biosynthesis |
|  |  |  |  |  |  |  |  |  |  |  |  |  | P:regulation of protein metabolism |
|  |  |  |  |  |  |  |  |  |  |  |  |  | P:biological\_process |
|  |  |  |  |  |  |  |  |  |  |  |  |  | P:ribosomal large subunit assembly and maintenance |
|  |  |  |  |  |  |  |  |  |  |  |  |  | P:regulation of protein biosynthesis |
|  |  |  |  |  |  |  |  |  |  |  |  |  | P:regulation of translation |
|  |  |  |  |  |  |  |  |  |  |  |  |  | P:protein complex assembly |
|  |  |  |  |  |  |  |  |  |  |  |  |  | P:regulation of translational fidelity |
|  |  |  |  |  |  |  |  |  |  |  |  |  | P:telomere organization and biogenesis |
|  |  |  |  |  |  |  |  |  |  |  |  |  | P:telomere maintenance |
|  |  |  |  |  |  |  |  |  |  |  |  |  | P:ribosomal small subunit assembly and maintenance |
|  |  |  |  |  |  |  |  |  |  |  |  |  | P:ribosome biogenesis and assembly |
|  |  |  |  |  |  |  |  |  |  |  |  |  | P:cytoplasm organization and biogenesis |
|  |  |  |  |  |  |  |  |  |  |  |  |  | P:ribosome assembly |
|  |  |  |  |  |  |  |  |  |  |  |  |  | P:ribosomal subunit assembly |
|  |  |  |  |  |  |  |  |  |  |  |  |  | P:cellular protein metabolism |
|  |  |  |  |  |  |  |  |  |  |  |  |  | P:cellular biosynthesis |
|  |  |  |  |  |  |  |  |  |  |  |  |  | P:cellular physiological process |
|  |  |  |  |  |  |  |  |  |  |  |  |  | P:cellular macromolecule metabolism |
|  |  |  |  |  |  |  |  |  |  |  |  |  | P:cellular process |
|  |  |  |  |  |  |  |  |  |  |  |  |  | P:cellular metabolism |
|  |  |  |  |  |  |  |  |  |  |  |  |  | P:physiological process |
|  |  |  |  |  |  |  |  |  |  |  |  |  | P:metabolism |
|  |  |  |  |  |  |  |  |  |  |  |  |  | P:primary metabolism |
|  |  |  |  |  |  |  |  |  |  |  |  |  | P:macromolecule biosynthesis |
|  |  |  |  |  |  |  |  |  |  |  |  |  | P:protein metabolism |
|  |  |  |  |  |  |  |  |  |  |  |  |  | P:cell surface receptor linked signal transduction |
|  |  |  |  |  |  |  |  |  |  |  |  |  | P:signal transduction |
|  |  |  |  |  |  |  |  |  |  |  |  |  | P:cell communication |
|  |  |  |  |  |  |  |  |  |  |  |  |  | P:growth |
|  |  |  |  |  |  |  |  |  |  |  |  |  | P:vitamin biosynthesis |
|  |  |  |  |  |  |  |  |  |  |  |  |  | P:water-soluble vitamin biosynthesis |
|  |  |  |  |  |  |  |  |  |  |  |  |  | P:charged-tRNA modification |
|  |  |  |  |  |  |  |  |  |  |  |  |  | P:nucleobase catabolism |
|  |  |  |  |  |  |  |  |  |  |  |  |  | P:purine base catabolism |
|  |  |  |  |  |  |  |  |  |  |  |  |  | P:sphingolipid metabolism |
|  |  |  |  |  |  |  |  |  |  |  |  |  | P:nucleobase metabolism |
|  |  |  |  |  |  |  |  |  |  |  |  |  | P:nucleoside monophosphate metabolism |
|  |  |  |  |  |  |  |  |  |  |  |  |  | P:pyrimidine nucleoside monophosphate biosynthesis |
|  |  |  |  |  |  |  |  |  |  |  |  |  | P:deoxyribonucleoside monophosphate metabolism |
|  |  |  |  |  |  |  |  |  |  |  |  |  | P:dTMP metabolism |
|  |  |  |  |  |  |  |  |  |  |  |  |  | P:pyrimidine deoxyribonucleoside monophosphate biosynthesis |
|  |  |  |  |  |  |  |  |  |  |  |  |  | P:pyrimidine deoxyribonucleoside monophosphate metabolism |
|  |  |  |  |  |  |  |  |  |  |  |  |  | P:deoxyribonucleoside monophosphate biosynthesis |
|  |  |  |  |  |  |  |  |  |  |  |  |  | P:dTMP biosynthesis |
|  |  |  |  |  |  |  |  |  |  |  |  |  | P:nucleoside monophosphate biosynthesis |
|  |  |  |  |  |  |  |  |  |  |  |  |  | P:pyrimidine nucleoside monophosphate metabolism |
|  |  |  |  |  |  |  |  |  |  |  |  |  | P:peptidyl-arginine methylation |
|  |  |  |  |  |  |  |  |  |  |  |  |  | P:aromatic compound metabolism |
|  |  |  |  |  |  |  |  |  |  |  |  |  | P:aromatic compound biosynthesis |
|  |  |  |  |  |  |  |  |  |  |  |  |  | P:osmosensory signaling pathway via Sho1 osmosensor |
|  |  |  |  |  |  |  |  |  |  |  |  |  | P:folic acid metabolism |
|  |  |  |  |  |  |  |  |  |  |  |  |  | P:folic acid biosynthesis |
|  |  |  |  |  |  |  |  |  |  |  |  |  | P:pteridine and derivative metabolism |
|  |  |  |  |  |  |  |  |  |  |  |  |  | P:pteridine and derivative biosynthesis |
|  |  |  |  |  |  |  |  |  |  |  |  |  | P:pyridoxine biosynthesis |
|  |  |  |  |  |  |  |  |  |  |  |  |  | P:anaerobic purine catabolism |
|  |  |  |  |  |  |  |  |  |  |  |  |  | P:nitrogenous compound catabolism |
|  |  |  |  |  |  |  |  |  |  |  |  |  | P:acetate biosynthesis from carbon monoxide |
|  |  |  |  |  |  |  |  |  |  |  |  |  | P:vitamin B6 biosynthesis |
|  |  |  |  |  |  |  |  |  |  |  |  |  | P:conversion of met-tRNAf to fmet-tRNA |
|  |  |  |  |  |  |  |  |  |  |  |  |  | P:purine base metabolism |
|  |  |  |  |  |  |  |  |  |  |  |  |  | P:sphingolipid biosynthesis |
|  |  |  |  |  |  |  |  |  |  |  |  |  | P:methionyl-tRNA aminoacylation |
|  |  |  |  |  |  |  |  |  |  |  |  |  | P:axial bud site selection |
|  |  |  |  |  |  |  |  |  |  |  |  |  | P:cytokinesis |
|  |  |  |  |  |  |  |  |  |  |  |  |  | P:budding cell bud growth |
|  |  |  |  |  |  |  |  |  |  |  |  |  | P:non-developmental growth |
|  |  |  |  |  |  |  |  |  |  |  |  |  | P:bud site selection |
|  |  |  |  |  |  |  |  |  |  |  |  |  | P:cytokinesis, site selection |
|  |  |  |  |  |  |  |  |  |  |  |  |  | P:cell division |
|  |  |  |  |  |  |  |  |  |  |  |  |  | P:asexual reproduction |
|  |  |  |  |  |  |  |  |  |  |  |  |  | P:cell budding |
|  |  |  |  |  |  |  |  |  |  |  |  |  | P:rNA metabolism |
|  |  |  |  |  |  |  |  |  |  |  |  |  | P:rNA 3'-end processing |
|
| Pho2 | Skn7 | Sfp1 | Fhl1 | Rap1 | Reb1 | Azf1 | Tec1 | Mbp1 | Swi4 | Swi6 | Ste12 | Stb1 | Molecular Function |
|  |  |  |  |  |  |  |  |  |  |  |  |  | F:adenine phosphoribosyltransferase activity |
|  |  |  |  |  |  |  |  |  |  |  |  |  | F:acetolactate synthase activity |
|  |  |  |  |  |  |  |  |  |  |  |  |  | F:coenzyme binding |
|  |  |  |  |  |  |  |  |  |  |  |  |  | F:fAD binding |
|  |  |  |  |  |  |  |  |  |  |  |  |  | F:methionine-tRNA ligase activity |
|  |  |  |  |  |  |  |  |  |  |  |  |  | F:rNA helicase activity |
|  |  |  |  |  |  |  |  |  |  |  |  |  | F:tRNA (guanine-N2-)-methyltransferase activity |
|  |  |  |  |  |  |  |  |  |  |  |  |  | F:ligase activity, forming aminoacyl-tRNA and related compounds |
|  |  |  |  |  |  |  |  |  |  |  |  |  | F:ligase activity, forming carbon-oxygen bonds |
|  |  |  |  |  |  |  |  |  |  |  |  |  | F:acid phosphatase activity |
|  |  |  |  |  |  |  |  |  |  |  |  |  | F:aminoacyl-tRNA ligase activity |
|  |  |  |  |  |  |  |  |  |  |  |  |  | F:protein tag |
|  |  |  |  |  |  |  |  |  |  |  |  |  | F:rNA binding |
|  |  |  |  |  |  |  |  |  |  |  |  |  | F:molecular\_function |
|  |  |  |  |  |  |  |  |  |  |  |  |  | F:structural constituent of ribosome |
|  |  |  |  |  |  |  |  |  |  |  |  |  | F:structural molecule activity |
|  |  |  |  |  |  |  |  |  |  |  |  |  | F:translation elongation factor activity |
|  |  |  |  |  |  |  |  |  |  |  |  |  | F:nucleic acid binding |
|  |  |  |  |  |  |  |  |  |  |  |  |  | F:transmembrane receptor activity |
|  |  |  |  |  |  |  |  |  |  |  |  |  | F:mating pheromone activity |
|  |  |  |  |  |  |  |  |  |  |  |  |  | F:receptor binding |
|  |  |  |  |  |  |  |  |  |  |  |  |  | F:osmosensor activity |
|  |  |  |  |  |  |  |  |  |  |  |  |  | F:pheromone activity |
|  |  |  |  |  |  |  |  |  |  |  |  |  | F:ligase activity |
|  |  |  |  |  |  |  |  |  |  |  |  |  | F:sphingosine hydroxylase activity |
|  |  |  |  |  |  |  |  |  |  |  |  |  | F:methenyltetrahydrofolate cyclohydrolase activity |
|  |  |  |  |  |  |  |  |  |  |  |  |  | F:methylenetetrahydrofolate dehydrogenase (NADP+) activity |
|  |  |  |  |  |  |  |  |  |  |  |  |  | F:formate-tetrahydrofolate ligase activity |
|  |  |  |  |  |  |  |  |  |  |  |  |  | F:thymidylate synthase activity |
|  |  |  |  |  |  |  |  |  |  |  |  |  | F:5,10-methylenetetrahydrofolate-dependent methyltransferase activity |
|  |  |  |  |  |  |  |  |  |  |  |  |  | F:chorismate mutase activity |
|  |  |  |  |  |  |  |  |  |  |  |  |  | F:phosphoribosylaminoimidazolesuccinocarboxamide synthase activity |
|  |  |  |  |  |  |  |  |  |  |  |  |  | F:ribose-5-phosphate isomerase activity |
|
| Pho2 | Skn7 | Sfp1 | Fhl1 | Rap1 | Reb1 | Azf1 | Tec1 | Mbp1 | Swi4 | Swi6 | Ste12 | Stb1 | Cellular Component |
|  |  |  |  |  |  |  |  |  |  |  |  |  | C:eukaryotic translation elongation factor 1 complex |
|  |  |  |  |  |  |  |  |  |  |  |  |  | C:nucleolar part |
|  |  |  |  |  |  |  |  |  |  |  |  |  | C:small nucleolar ribonucleoprotein complex |
|  |  |  |  |  |  |  |  |  |  |  |  |  | C:cellular\_component |
|  |  |  |  |  |  |  |  |  |  |  |  |  | C:cell part |
|  |  |  |  |  |  |  |  |  |  |  |  |  | C:intracellular organelle |
|  |  |  |  |  |  |  |  |  |  |  |  |  | C:organelle |
|  |  |  |  |  |  |  |  |  |  |  |  |  | C:cytoplasm |
|  |  |  |  |  |  |  |  |  |  |  |  |  | C:small ribosomal subunit |
|  |  |  |  |  |  |  |  |  |  |  |  |  | C:intracellular part |
|  |  |  |  |  |  |  |  |  |  |  |  |  | C:eukaryotic 48S initiation complex |
|  |  |  |  |  |  |  |  |  |  |  |  |  | C:cytosolic small ribosomal subunit (sensu Eukaryota) |
|  |  |  |  |  |  |  |  |  |  |  |  |  | C:protein complex |
|  |  |  |  |  |  |  |  |  |  |  |  |  | C:eukaryotic 43S preinitiation complex |
|  |  |  |  |  |  |  |  |  |  |  |  |  | C:intracellular |
|  |  |  |  |  |  |  |  |  |  |  |  |  | C:cell |
|  |  |  |  |  |  |  |  |  |  |  |  |  | C:cytosol |
|  |  |  |  |  |  |  |  |  |  |  |  |  | C:cytosolic large ribosomal subunit (sensu Eukaryota) |
|  |  |  |  |  |  |  |  |  |  |  |  |  | C:organelle part |
|  |  |  |  |  |  |  |  |  |  |  |  |  | C:intracellular organelle part |
|  |  |  |  |  |  |  |  |  |  |  |  |  | C:large ribosomal subunit |
|  |  |  |  |  |  |  |  |  |  |  |  |  | C:cytoplasmic part |
|  |  |  |  |  |  |  |  |  |  |  |  |  | C:ribonucleoprotein complex |
|  |  |  |  |  |  |  |  |  |  |  |  |  | C:intracellular non-membrane-bound organelle |
|  |  |  |  |  |  |  |  |  |  |  |  |  | C:non-membrane-bound organelle |
|  |  |  |  |  |  |  |  |  |  |  |  |  | C:ribosome |
|  |  |  |  |  |  |  |  |  |  |  |  |  | C:cytosolic part |
|  |  |  |  |  |  |  |  |  |  |  |  |  | C:cytosolic ribosome (sensu Eukaryota) |
|  |  |  |  |  |  |  |  |  |  |  |  |  | C:bud |
|  |  |  |  |  |  |  |  |  |  |  |  |  | C:nucleolus |
|  |  |  |  |  |  |  |  |  |  |  |  |  | C:u4/U6 x U5 tri-snRNP complex |
|  |  |  |  |  |  |  |  |  |  |  |  |  | C:acetolactate synthase complex |
|  |  |  |  |  |  |  |  |  |  |  |  |  | C:methionyl glutamyl tRNA synthetase complex |
|  |  |  |  |  |  |  |  |  |  |  |  |  | C:lipid particle |
|  |  |  |  |  |  |  |  |  |  |  |  |  | C:cell projection |
|  |  |  |  |  |  |  |  |  |  |  |  |  | C:mating projection |
|  |  |  |  |  |  |  |  |  |  |  |  |  | C:mating projection tip |
|  |  |  |  |  |  |  |  |  |  |  |  |  | C:cell projection part |
|  |  |  |  |  |  |  |  |  |  |  |  |  | C:external encapsulating structure |
|  |  |  |  |  |  |  |  |  |  |  |  |  | C:cell wall (sensu Fungi) |
|  |  |  |  |  |  |  |  |  |  |  |  |  | C:cell wall |
|  |  |  |  |  |  |  |  |  |  |  |  |  | C:vacuole |
|  |  |  |  |  |  |  |  |  |  |  |  |  | C:extracellular region |
|  |  |  |  |  |  |  |  |  |  |  |  |  | C:vacuole (sensu Fungi) |
|  |  |  |  |  |  |  |  |  |  |  |  |  | C:storage vacuole |
|  |  |  |  |  |  |  |  |  |  |  |  |  | C:lytic vacuole |
|  |  |  |  |  |  |  |  |  |  |  |  |  | C:site of polarized growth |
|  |  |  |  |  |  |  |  |  |  |  |  |  | C:snRNP U6 |
|  |  |  |  |  |  |  |  |  |  |  |  |  | C:bud tip |
|
