# Supplementary material for: High-resolution analysis of condition-specific regulatory modules in Saccharomyces cerevisiae
Source: Genome Biol. 2008 Jan 3;9(1):R2. doi: 10.1186/gb-2008-9-1-r2 (PMC2395236; doi:10.1186/gb-2008-9-1-r2)
Supplement: Additional data file 11 — Matrices describing all EPMs and RMs, including lists of synergistic pairs of regulators. [file gb-2008-9-1-r2-S11.zip › htmls/C0_EPMs_matrix/EPM_7.RM.matrix.html]

Regulators vs. RM target gene list

|  |  |  |  |  |  |  |  |  |  |  |  |  |  |
| --- | --- | --- | --- | --- | --- | --- | --- | --- | --- | --- | --- | --- | --- |
|  | Pho2 | Skn7 | Sfp1 | Fhl1 | Rap1 | Reb1 | Azf1 | Tec1 | Mbp1 | Swi4 | Swi6 | Ste12 | Stb1 |
| RM\_1 |  |  |  |  |  |  |  |  |  |  |  |  |  |
| RM\_2 |  |  |  |  |  |  |  |  |  |  |  |  |  |
| RM\_3 |  |  |  |  |  |  |  |  |  |  |  |  |  |
| RM\_4 |  |  |  |  |  |  |  |  |  |  |  |  |  |

Synergistic Pair of Regulators

1. Swi4\*Swi6

2. Swi6\*Tec1

3. Ste12\*Swi4

4. Pho2\*Rap1

5. Pho2\*Sfp1

6. Rap1\*Sfp1

7. Fhl1\*Pho2

8. Fhl1\*Rap1

9. Rap1\*Skn7

10. Mbp1\*Swi4

11. Mbp1\*Swi6

12. Fhl1\*Sfp1

13. Stb1\*Swi6

14. Stb1\*Swi4

Matrix of enriched GO

EPM matrix
