# Supplementary material for: High-resolution analysis of condition-specific regulatory modules in Saccharomyces cerevisiae
Source: Genome Biol. 2008 Jan 3;9(1):R2. doi: 10.1186/gb-2008-9-1-r2 (PMC2395236; doi:10.1186/gb-2008-9-1-r2)
Supplement: Additional data file 11 — Matrices describing all EPMs and RMs, including lists of synergistic pairs of regulators. [file gb-2008-9-1-r2-S11.zip › htmls/C0_EPMs_matrix/EPM_8.GO_enrichment.matrix.html]

|  |  |  |  |  |  |
| --- | --- | --- | --- | --- | --- |
| Swi4 | Mbp1 | Swi6 | Azf1 | Stb1 | Biological Process |
|  |  |  |  |  | P:cell cycle checkpoint |
|  |  |  |  |  | P:cell wall organization and biogenesis |
|  |  |  |  |  | P:external encapsulating structure organization and biogenesis |
|  |  |  |  |  | P:positive regulation of apoptosis |
|  |  |  |  |  | P:positive regulation of programmed cell death |
|  |  |  |  |  | P:cell cycle |
|  |  |  |  |  | P:m phase |
|  |  |  |  |  | P:m phase of mitotic cell cycle |
|  |  |  |  |  | P:mitosis |
|  |  |  |  |  | P:spore germination |
|  |  |  |  |  | P:mitotic cell cycle |
|  |  |  |  |  | P:organelle organization and biogenesis |
|  |  |  |  |  | P:dNA replication |
|  |  |  |  |  | P:chromosome segregation |
|  |  |  |  |  | P:induction of programmed cell death |
|  |  |  |  |  | P:induction of apoptosis |
|  |  |  |  |  | P:induction of apoptosis by intracellular signals |
|  |  |  |  |  | P:dNA damage response, signal transduction resulting in induction of apoptosis |
|  |  |  |  |  | P:chromosome organization and biogenesis |
|  |  |  |  |  | P:sister chromatid segregation |
|  |  |  |  |  | P:mitotic sister chromatid segregation |
|  |  |  |  |  | P:chromosome condensation |
|  |  |  |  |  | P:mitotic chromosome condensation |
|  |  |  |  |  | P:sister chromatid cohesion |
|  |  |  |  |  | P:mitotic sister chromatid cohesion |
|  |  |  |  |  | P:dNA synthesis during DNA repair |
|  |  |  |  |  | P:mannose metabolism |
|  |  |  |  |  | P:gDP-mannose metabolism |
|  |  |  |  |  | P:cell morphogenesis checkpoint |
|  |  |  |  |  | P:gDP-mannose biosynthesis |
|  |  |  |  |  | P:nucleotide-sugar biosynthesis |
|  |  |  |  |  | P:mannose biosynthesis |
|  |  |  |  |  | P:g2/M transition checkpoint |
|  |  |  |  |  | P:cell size control checkpoint |
|  |  |  |  |  | P:cell organization and biogenesis |
|  |  |  |  |  | P:g2/M transition size control checkpoint |
|
| Swi4 | Mbp1 | Swi6 | Azf1 | Stb1 | Molecular Function |
|  |  |  |  |  | F:alpha DNA polymerase activity |
|  |  |  |  |  | F:mannose-1-phosphate guanylyltransferase activity |
|  |  |  |  |  | F:c-5 sterol desaturase activity |
|  |  |  |  |  | F:mannose-phosphate guanylyltransferase activity |
|
| Swi4 | Mbp1 | Swi6 | Azf1 | Stb1 | Cellular Component |
|  |  |  |  |  | C:vacuole (sensu Fungi) |
|  |  |  |  |  | C:lytic vacuole |
|  |  |  |  |  | C:site of polarized growth |
|  |  |  |  |  | C:storage vacuole |
|  |  |  |  |  | C:bud |
|  |  |  |  |  | C:extracellular region |
|  |  |  |  |  | C:external encapsulating structure |
|  |  |  |  |  | C:cell wall (sensu Fungi) |
|  |  |  |  |  | C:cell wall |
|  |  |  |  |  | C:chromosome |
|  |  |  |  |  | C:condensed chromosome |
|  |  |  |  |  | C:nuclear chromosome |
|  |  |  |  |  | C:condensed nuclear chromosome |
|  |  |  |  |  | C:cohesin complex |
|  |  |  |  |  | C:nuclear cohesin complex |
|
