# Supplementary material for: High-resolution analysis of condition-specific regulatory modules in Saccharomyces cerevisiae
Source: Genome Biol. 2008 Jan 3;9(1):R2. doi: 10.1186/gb-2008-9-1-r2 (PMC2395236; doi:10.1186/gb-2008-9-1-r2)
Supplement: Additional data file 11 — Matrices describing all EPMs and RMs, including lists of synergistic pairs of regulators. [file gb-2008-9-1-r2-S11.zip › htmls/C0_EPMs_matrix/EPM_9.GO_enrichment.matrix.html]

|  |  |  |  |  |
| --- | --- | --- | --- | --- |
| Swi4 | Swi6 | Mbp1 | Stb1 | Biological Process |
|  |  |  |  | P:development |
|  |  |  |  | P:positive regulation of protein kinase activity |
|  |  |  |  | P:positive regulation of transferase activity |
|  |  |  |  | P:small GTPase mediated signal transduction |
|  |  |  |  | P:regulation of mitosis |
|  |  |  |  | P:activation of MAPKK activity |
|  |  |  |  | P:activation of MAPKK activity during osmolarity sensing |
|  |  |  |  | P:activation of protein kinase activity |
|  |  |  |  | P:g1/S transition of mitotic cell cycle |
|  |  |  |  | P:intracellular signaling cascade |
|  |  |  |  | P:regulation of catalytic activity |
|  |  |  |  | P:mitotic cell cycle |
|  |  |  |  | P:regulation of biological process |
|  |  |  |  | P:axial bud site selection |
|  |  |  |  | P:regulation of exit from mitosis |
|  |  |  |  | P:regulation of protein kinase activity |
|  |  |  |  | P:regulation of kinase activity |
|  |  |  |  | P:regulation of transferase activity |
|  |  |  |  | P:cell budding |
|  |  |  |  | P:asexual reproduction |
|  |  |  |  | P:rho protein signal transduction |
|  |  |  |  | P:cellular morphogenesis |
|  |  |  |  | P:morphogenesis |
|  |  |  |  | P:g1/S-specific transcription in mitotic cell cycle |
|  |  |  |  | P:regulation of cell cycle |
|  |  |  |  | P:regulation of progression through cell cycle |
|  |  |  |  | P:cell cycle |
|  |  |  |  | P:establishment and/or maintenance of cell polarity |
|  |  |  |  | P:establishment and/or maintenance of cell polarity (sensu Fungi) |
|  |  |  |  | P:establishment of cell polarity |
|  |  |  |  | P:establishment of cell polarity (sensu Fungi) |
|  |  |  |  | P:bud site selection |
|  |  |  |  | P:cytokinesis, site selection |
|  |  |  |  | P:cell division |
|  |  |  |  | P:cytokinesis |
|  |  |  |  | P:regulation of physiological process |
|  |  |  |  | P:regulation of cellular physiological process |
|  |  |  |  | P:regulation of cellular process |
|  |  |  |  | P:signal transduction |
|  |  |  |  | P:cell communication |
|
| Swi4 | Swi6 | Mbp1 | Stb1 | Molecular Function |
|  |  |  |  | F:mAP kinase kinase kinase activity |
|  |  |  |  | F:gTPase regulator activity |
|  |  |  |  | F:c-3 sterol dehydrogenase (C-4 sterol decarboxylase) activity |
|  |  |  |  | F:adenine phosphoribosyltransferase activity |
|  |  |  |  | F:small GTPase regulator activity |
|  |  |  |  | F:kinase regulator activity |
|  |  |  |  | F:protein kinase regulator activity |
|  |  |  |  | F:hydrolase activity, acting on glycosyl bonds |
|  |  |  |  | F:hydrolase activity, hydrolyzing O-glycosyl compounds |
|  |  |  |  | F:glucosidase activity |
|  |  |  |  | F:cyclin-dependent protein kinase regulator activity |
|  |  |  |  | F:enzyme regulator activity |
|  |  |  |  | F:glucan 1,3-beta-glucosidase activity |
|  |  |  |  | F:beta-glucosidase activity |
|
| Swi4 | Swi6 | Mbp1 | Stb1 | Cellular Component |
|  |  |  |  | C:dNA replication factor A complex |
|  |  |  |  | C:cell wall part |
|  |  |  |  | C:external encapsulating structure part |
|  |  |  |  | C:cortical cytoskeleton |
|  |  |  |  | C:cortical actin cytoskeleton |
|  |  |  |  | C:cell projection |
|  |  |  |  | C:mating projection |
|  |  |  |  | C:cell projection part |
|  |  |  |  | C:incipient bud site |
|  |  |  |  | C:bud scar |
|  |  |  |  | C:mating projection tip |
|  |  |  |  | C:external encapsulating structure |
|  |  |  |  | C:cell wall |
|  |  |  |  | C:cell wall (sensu Fungi) |
|  |  |  |  | C:actin cap |
|  |  |  |  | C:bud tip |
|  |  |  |  | C:bud neck |
|  |  |  |  | C:bud |
|  |  |  |  | C:site of polarized growth |
|
