# Supplementary material for: High-resolution analysis of condition-specific regulatory modules in Saccharomyces cerevisiae
Source: Genome Biol. 2008 Jan 3;9(1):R2. doi: 10.1186/gb-2008-9-1-r2 (PMC2395236; doi:10.1186/gb-2008-9-1-r2)
Supplement: Additional data file 11 — Matrices describing all EPMs and RMs, including lists of synergistic pairs of regulators. [file gb-2008-9-1-r2-S11.zip › htmls/C13_EPMs_matrix/EPM_1.GO_enrichment.matrix.html]

|  |  |  |  |  |  |  |  |  |  |  |  |
| --- | --- | --- | --- | --- | --- | --- | --- | --- | --- | --- | --- |
| Xbp1 | Gcn4 | Bas1 | Met32 | Cbf1 | Tye7 | Swi6 | Reb1 | Abf1 | Dig1 | Ste12 | Biological Process |
|  |  |  |  |  |  |  |  |  |  |  | P:posttranslational protein targeting to membrane, translocation |
|  |  |  |  |  |  |  |  |  |  |  | P:cellular biosynthesis |
|  |  |  |  |  |  |  |  |  |  |  | P:biosynthesis |
|  |  |  |  |  |  |  |  |  |  |  | P:glutamine family amino acid metabolism |
|  |  |  |  |  |  |  |  |  |  |  | P:urea cycle intermediate metabolism |
|  |  |  |  |  |  |  |  |  |  |  | P:arginine metabolism |
|  |  |  |  |  |  |  |  |  |  |  | P:arginine biosynthesis |
|  |  |  |  |  |  |  |  |  |  |  | P:glutamine family amino acid biosynthesis |
|  |  |  |  |  |  |  |  |  |  |  | P:guanine nucleotide transport |
|  |  |  |  |  |  |  |  |  |  |  | P:argininosuccinate metabolism |
|  |  |  |  |  |  |  |  |  |  |  | P:citrulline metabolism |
|  |  |  |  |  |  |  |  |  |  |  | P:nonprotein amino acid biosynthesis |
|  |  |  |  |  |  |  |  |  |  |  | P:ornithine biosynthesis |
|  |  |  |  |  |  |  |  |  |  |  | P:nonprotein amino acid metabolism |
|  |  |  |  |  |  |  |  |  |  |  | P:ornithine metabolism |
|  |  |  |  |  |  |  |  |  |  |  | P:sulfate transport |
|  |  |  |  |  |  |  |  |  |  |  | P:sulfur amino acid transport |
|  |  |  |  |  |  |  |  |  |  |  | P:cysteine biosynthesis |
|  |  |  |  |  |  |  |  |  |  |  | P:tryptophanyl-tRNA aminoacylation |
|  |  |  |  |  |  |  |  |  |  |  | P:extrachromosomal circular DNA accumulation during cell aging |
|  |  |  |  |  |  |  |  |  |  |  | P:iron incorporation into metallo-sulfur cluster |
|  |  |  |  |  |  |  |  |  |  |  | P:metal incorporation into metallo-sulfur cluster |
|  |  |  |  |  |  |  |  |  |  |  | P:extrachromosomal rDNA circle accumulation during replicative cell aging |
|  |  |  |  |  |  |  |  |  |  |  | P:extrachromosomal circular DNA accumulation during replicative cell aging |
|  |  |  |  |  |  |  |  |  |  |  | P:sulfate assimilation |
|  |  |  |  |  |  |  |  |  |  |  | P:sulfur utilization |
|  |  |  |  |  |  |  |  |  |  |  | P:nitrogen compound biosynthesis |
|  |  |  |  |  |  |  |  |  |  |  | P:amino acid biosynthesis |
|  |  |  |  |  |  |  |  |  |  |  | P:amine biosynthesis |
|  |  |  |  |  |  |  |  |  |  |  | P:amine metabolism |
|  |  |  |  |  |  |  |  |  |  |  | P:nitrogen compound metabolism |
|  |  |  |  |  |  |  |  |  |  |  | P:organic acid metabolism |
|  |  |  |  |  |  |  |  |  |  |  | P:carboxylic acid metabolism |
|  |  |  |  |  |  |  |  |  |  |  | P:amino acid and derivative metabolism |
|  |  |  |  |  |  |  |  |  |  |  | P:amino acid metabolism |
|  |  |  |  |  |  |  |  |  |  |  | P:hyperosmotic salinity response |
|  |  |  |  |  |  |  |  |  |  |  | P:aspartate family amino acid biosynthesis |
|  |  |  |  |  |  |  |  |  |  |  | P:aspartate family amino acid metabolism |
|  |  |  |  |  |  |  |  |  |  |  | P:sulfur amino acid metabolism |
|  |  |  |  |  |  |  |  |  |  |  | P:methionine metabolism |
|  |  |  |  |  |  |  |  |  |  |  | P:cysteine metabolism |
|  |  |  |  |  |  |  |  |  |  |  | P:transsulfuration |
|  |  |  |  |  |  |  |  |  |  |  | P:sulfur metabolism |
|  |  |  |  |  |  |  |  |  |  |  | P:methionine biosynthesis |
|  |  |  |  |  |  |  |  |  |  |  | P:primary metabolism |
|  |  |  |  |  |  |  |  |  |  |  | P:metabolism |
|  |  |  |  |  |  |  |  |  |  |  | P:cellular metabolism |
|  |  |  |  |  |  |  |  |  |  |  | P:cellular physiological process |
|  |  |  |  |  |  |  |  |  |  |  | P:homoserine metabolism |
|  |  |  |  |  |  |  |  |  |  |  | P:physiological process |
|  |  |  |  |  |  |  |  |  |  |  | P:cellular process |
|  |  |  |  |  |  |  |  |  |  |  | P:serine family amino acid metabolism |
|  |  |  |  |  |  |  |  |  |  |  | P:sulfur amino acid biosynthesis |
|  |  |  |  |  |  |  |  |  |  |  | P:sulfur compound biosynthesis |
|  |  |  |  |  |  |  |  |  |  |  | P:vesicle-mediated transport |
|  |  |  |  |  |  |  |  |  |  |  | P:siroheme metabolism |
|  |  |  |  |  |  |  |  |  |  |  | P:siroheme biosynthesis |
|  |  |  |  |  |  |  |  |  |  |  | P:amino sugar catabolism |
|  |  |  |  |  |  |  |  |  |  |  | P:chitin catabolism |
|  |  |  |  |  |  |  |  |  |  |  | P:glucosamine catabolism |
|  |  |  |  |  |  |  |  |  |  |  | P:n-acetylglucosamine catabolism |
|  |  |  |  |  |  |  |  |  |  |  | P:cell wall catabolism |
|  |  |  |  |  |  |  |  |  |  |  | P:cell wall chitin catabolism |
|  |  |  |  |  |  |  |  |  |  |  | P:axial bud site selection |
|  |  |  |  |  |  |  |  |  |  |  | P:cytokinesis, site selection |
|  |  |  |  |  |  |  |  |  |  |  | P:bud site selection |
|  |  |  |  |  |  |  |  |  |  |  | P:m phase of mitotic cell cycle |
|  |  |  |  |  |  |  |  |  |  |  | P:mitosis |
|  |  |  |  |  |  |  |  |  |  |  | P:cell cycle |
|  |  |  |  |  |  |  |  |  |  |  | P:mitotic cell cycle |
|  |  |  |  |  |  |  |  |  |  |  | P:s phase of mitotic cell cycle |
|  |  |  |  |  |  |  |  |  |  |  | P:s phase |
|  |  |  |  |  |  |  |  |  |  |  | P:regulation of cell cycle |
|  |  |  |  |  |  |  |  |  |  |  | P:regulation of progression through cell cycle |
|  |  |  |  |  |  |  |  |  |  |  | P:cytogamy |
|  |  |  |  |  |  |  |  |  |  |  | P:premeiotic DNA synthesis |
|  |  |  |  |  |  |  |  |  |  |  | P:regulation of S phase of mitotic cell cycle |
|  |  |  |  |  |  |  |  |  |  |  | P:spindle assembly |
|  |  |  |  |  |  |  |  |  |  |  | P:positive regulation of DNA replication |
|  |  |  |  |  |  |  |  |  |  |  | P:spore germination |
|  |  |  |  |  |  |  |  |  |  |  | P:positive regulation of DNA metabolism |
|  |  |  |  |  |  |  |  |  |  |  | P:protein export from nucleus |
|  |  |  |  |  |  |  |  |  |  |  | P:meiosis |
|  |  |  |  |  |  |  |  |  |  |  | P:meiotic cell cycle |
|  |  |  |  |  |  |  |  |  |  |  | P:m phase of meiotic cell cycle |
|  |  |  |  |  |  |  |  |  |  |  | P:conjugation with cellular fusion |
|  |  |  |  |  |  |  |  |  |  |  | P:conjugation |
|  |  |  |  |  |  |  |  |  |  |  | P:plasma membrane fusion during cytogamy |
|  |  |  |  |  |  |  |  |  |  |  | P:sexual reproduction |
|  |  |  |  |  |  |  |  |  |  |  | P:establishment of chromatin silencing |
|  |  |  |  |  |  |  |  |  |  |  | P:dNA replication initiation |
|  |  |  |  |  |  |  |  |  |  |  | P:ribosome export from nucleus |
|  |  |  |  |  |  |  |  |  |  |  | P:dNA unwinding during replication |
|  |  |  |  |  |  |  |  |  |  |  | P:pre-replicative complex formation and maintenance |
|  |  |  |  |  |  |  |  |  |  |  | P:nuclear mRNA 5'-splice site recognition |
|  |  |  |  |  |  |  |  |  |  |  | P:rNA import into nucleus |
|
| Xbp1 | Gcn4 | Bas1 | Met32 | Cbf1 | Tye7 | Swi6 | Reb1 | Abf1 | Dig1 | Ste12 | Molecular Function |
|  |  |  |  |  |  |  |  |  |  |  | F:kinase regulator activity |
|  |  |  |  |  |  |  |  |  |  |  | F:cyclin-dependent protein kinase regulator activity |
|  |  |  |  |  |  |  |  |  |  |  | F:protein kinase regulator activity |
|  |  |  |  |  |  |  |  |  |  |  | F:sulfate transporter activity |
|  |  |  |  |  |  |  |  |  |  |  | F:nucleotidase activity |
|  |  |  |  |  |  |  |  |  |  |  | F:oxidoreductase activity, acting on sulfur group of donors, disulfide as acceptor |
|  |  |  |  |  |  |  |  |  |  |  | F:iron ion binding |
|  |  |  |  |  |  |  |  |  |  |  | F:ferrous iron binding |
|  |  |  |  |  |  |  |  |  |  |  | F:tryptophan-tRNA ligase activity |
|  |  |  |  |  |  |  |  |  |  |  | F:cystathionine gamma-lyase activity |
|  |  |  |  |  |  |  |  |  |  |  | F:l-methionine transporter activity |
|  |  |  |  |  |  |  |  |  |  |  | F:cysteine synthase activity |
|  |  |  |  |  |  |  |  |  |  |  | F:sulfite reductase (NADPH) activity |
|  |  |  |  |  |  |  |  |  |  |  | F:oxidoreductase activity, acting on sulfur group of donors, NAD or NADP as acceptor |
|  |  |  |  |  |  |  |  |  |  |  | F:3'(2'),5'-bisphosphate nucleotidase activity |
|  |  |  |  |  |  |  |  |  |  |  | F:catalytic activity |
|  |  |  |  |  |  |  |  |  |  |  | F:phosphoadenylyl-sulfate reductase (thioredoxin) activity |
|  |  |  |  |  |  |  |  |  |  |  | F:arylformamidase activity |
|  |  |  |  |  |  |  |  |  |  |  | F:sulfate adenylyltransferase (ATP) activity |
|  |  |  |  |  |  |  |  |  |  |  | F:homoserine O-acetyltransferase activity |
|  |  |  |  |  |  |  |  |  |  |  | F:o-acetylhomoserine aminocarboxypropyltransferase activity |
|  |  |  |  |  |  |  |  |  |  |  | F:adenylylsulfate kinase activity |
|  |  |  |  |  |  |  |  |  |  |  | F:l-methionine porter activity |
|  |  |  |  |  |  |  |  |  |  |  | F:neutral L-amino acid porter activity |
|  |  |  |  |  |  |  |  |  |  |  | F:sulfate adenylyltransferase activity |
|  |  |  |  |  |  |  |  |  |  |  | F:oxidoreductase activity, acting on sulfur group of donors |
|  |  |  |  |  |  |  |  |  |  |  | F:electron carrier activity |
|  |  |  |  |  |  |  |  |  |  |  | F:lyase activity |
|  |  |  |  |  |  |  |  |  |  |  | F:transferase activity |
|  |  |  |  |  |  |  |  |  |  |  | F:polyamine transporter activity |
|  |  |  |  |  |  |  |  |  |  |  | F:l-amino acid transporter activity |
|  |  |  |  |  |  |  |  |  |  |  | F:organic acid transporter activity |
|  |  |  |  |  |  |  |  |  |  |  | F:carboxylic acid transporter activity |
|  |  |  |  |  |  |  |  |  |  |  | F:neutral amino acid transporter activity |
|  |  |  |  |  |  |  |  |  |  |  | F:amino acid-polyamine transporter activity |
|  |  |  |  |  |  |  |  |  |  |  | F:acetylglutamate kinase activity |
|  |  |  |  |  |  |  |  |  |  |  | F:acetylornithine transaminase activity |
|  |  |  |  |  |  |  |  |  |  |  | F:n-acetyl-gamma-glutamyl-phosphate reductase activity |
|  |  |  |  |  |  |  |  |  |  |  | F:guanine nucleotide transporter activity |
|  |  |  |  |  |  |  |  |  |  |  | F:argininosuccinate synthase activity |
|  |  |  |  |  |  |  |  |  |  |  | F:argininosuccinate lyase activity |
|  |  |  |  |  |  |  |  |  |  |  | F:ligase activity |
|  |  |  |  |  |  |  |  |  |  |  | F:carbon-nitrogen ligase activity, with glutamine as amido-N-donor |
|  |  |  |  |  |  |  |  |  |  |  | F:ligase activity, forming carbon-nitrogen bonds |
|  |  |  |  |  |  |  |  |  |  |  | F:ornithine carbamoyltransferase activity |
|  |  |  |  |  |  |  |  |  |  |  | F:carbamoyl-phosphate synthase (glutamine-hydrolyzing) activity |
|  |  |  |  |  |  |  |  |  |  |  | F:carbamoyl-phosphate synthase activity |
|  |  |  |  |  |  |  |  |  |  |  | F:amino-acid N-acetyltransferase activity |
|  |  |  |  |  |  |  |  |  |  |  | F:asparagine synthase (glutamine-hydrolyzing) activity |
|  |  |  |  |  |  |  |  |  |  |  | F:carboxyl- and carbamoyltransferase activity |
|  |  |  |  |  |  |  |  |  |  |  | F:amidine-lyase activity |
|  |  |  |  |  |  |  |  |  |  |  | F:purine nucleotide transporter activity |
|  |  |  |  |  |  |  |  |  |  |  | F:c-methyltransferase activity |
|  |  |  |  |  |  |  |  |  |  |  | F:aTP-dependent helicase activity |
|  |  |  |  |  |  |  |  |  |  |  | F:dNA helicase activity |
|  |  |  |  |  |  |  |  |  |  |  | F:dNA-dependent ATPase activity |
|  |  |  |  |  |  |  |  |  |  |  | F:aTP-dependent DNA helicase activity |
|  |  |  |  |  |  |  |  |  |  |  | F:chromatin binding |
|  |  |  |  |  |  |  |  |  |  |  | F:ran GTPase activator activity |
|  |  |  |  |  |  |  |  |  |  |  | F:phosphoribosylaminoimidazolesuccinocarboxamide synthase activity |
|  |  |  |  |  |  |  |  |  |  |  | F:pre-mRNA 5'-splice site binding |
|  |  |  |  |  |  |  |  |  |  |  | F:uroporphyrin-III C-methyltransferase activity |
|  |  |  |  |  |  |  |  |  |  |  | F:lipid binding |
|  |  |  |  |  |  |  |  |  |  |  | F:beta-1,4-mannosyltransferase activity |
|  |  |  |  |  |  |  |  |  |  |  | F:motor activity |
|  |  |  |  |  |  |  |  |  |  |  | F:phospholipid binding |
|  |  |  |  |  |  |  |  |  |  |  | F:phosphoinositide binding |
|  |  |  |  |  |  |  |  |  |  |  | F:phosphatidylinositol binding |
|  |  |  |  |  |  |  |  |  |  |  | F:microtubule motor activity |
|  |  |  |  |  |  |  |  |  |  |  | F:transferase activity, transferring alkyl or aryl (other than methyl) groups |
|  |  |  |  |  |  |  |  |  |  |  | F:structural constituent of cytoskeleton |
|  |  |  |  |  |  |  |  |  |  |  | F:3-deoxy-7-phosphoheptulonate synthase activity |
|  |  |  |  |  |  |  |  |  |  |  | F:signal peptidase activity |
|  |  |  |  |  |  |  |  |  |  |  | F:aspartic-type signal peptidase activity |
|  |  |  |  |  |  |  |  |  |  |  | F:protein transporter activity |
|
| Xbp1 | Gcn4 | Bas1 | Met32 | Cbf1 | Tye7 | Swi6 | Reb1 | Abf1 | Dig1 | Ste12 | Cellular Component |
|  |  |  |  |  |  |  |  |  |  |  | C:cyclin-dependent protein kinase holoenzyme complex |
|  |  |  |  |  |  |  |  |  |  |  | C:cohesin complex |
|  |  |  |  |  |  |  |  |  |  |  | C:nuclear cohesin complex |
|  |  |  |  |  |  |  |  |  |  |  | C:replication fork |
|  |  |  |  |  |  |  |  |  |  |  | C:replication fork (sensu Eukaryota) |
|  |  |  |  |  |  |  |  |  |  |  | C:mCM complex |
|  |  |  |  |  |  |  |  |  |  |  | C:pre-replicative complex |
|  |  |  |  |  |  |  |  |  |  |  | C:cdc73/Paf1 complex |
|  |  |  |  |  |  |  |  |  |  |  | C:cell division site |
|  |  |  |  |  |  |  |  |  |  |  | C:cell division site part |
|  |  |  |  |  |  |  |  |  |  |  | C:septin cytoskeleton |
|  |  |  |  |  |  |  |  |  |  |  | C:septin ring |
|  |  |  |  |  |  |  |  |  |  |  | C:bud neck septin structure |
|  |  |  |  |  |  |  |  |  |  |  | C:bud neck septin ring |
|  |  |  |  |  |  |  |  |  |  |  | C:kinesin complex |
|  |  |  |  |  |  |  |  |  |  |  | C:cleavage apparatus septin structure |
|  |  |  |  |  |  |  |  |  |  |  | C:sulfite reductase complex (NADPH) |
|  |  |  |  |  |  |  |  |  |  |  | C:cytoplasm |
|  |  |  |  |  |  |  |  |  |  |  | C:cell part |
|  |  |  |  |  |  |  |  |  |  |  | C:cell |
|  |  |  |  |  |  |  |  |  |  |  | C:intracellular part |
|  |  |  |  |  |  |  |  |  |  |  | C:intracellular |
|  |  |  |  |  |  |  |  |  |  |  | C:sec complex-associated translocon complex |
|  |  |  |  |  |  |  |  |  |  |  | C:cytoplasmic part |
|  |  |  |  |  |  |  |  |  |  |  | C:carbamoyl-phosphate synthase complex |
|  |  |  |  |  |  |  |  |  |  |  | C:mitochondrial matrix |
|  |  |  |  |  |  |  |  |  |  |  | C:mitochondrial lumen |
|
