# Supplementary material for: High-resolution analysis of condition-specific regulatory modules in Saccharomyces cerevisiae
Source: Genome Biol. 2008 Jan 3;9(1):R2. doi: 10.1186/gb-2008-9-1-r2 (PMC2395236; doi:10.1186/gb-2008-9-1-r2)
Supplement: Additional data file 11 — Matrices describing all EPMs and RMs, including lists of synergistic pairs of regulators. [file gb-2008-9-1-r2-S11.zip › htmls/C13_EPMs_matrix/EPM_11.GO_enrichment.matrix.html]

|  |  |  |  |  |  |  |  |  |  |
| --- | --- | --- | --- | --- | --- | --- | --- | --- | --- |
| Smp1 | Skn7 | Fhl1 | Rap1 | Sfp1 | Aft2 | Sut1 | Pdr1 | Yap5 | Biological Process |
|  |  |  |  |  |  |  |  |  | P:ribosomal small subunit export from nucleus |
|  |  |  |  |  |  |  |  |  | P:biosynthesis |
|  |  |  |  |  |  |  |  |  | P:cellular macromolecule metabolism |
|  |  |  |  |  |  |  |  |  | P:macromolecule biosynthesis |
|  |  |  |  |  |  |  |  |  | P:protein biosynthesis |
|  |  |  |  |  |  |  |  |  | P:protein metabolism |
|  |  |  |  |  |  |  |  |  | P:macromolecule metabolism |
|  |  |  |  |  |  |  |  |  | P:cellular biosynthesis |
|  |  |  |  |  |  |  |  |  | P:translation |
|  |  |  |  |  |  |  |  |  | P:cellular protein metabolism |
|  |  |  |  |  |  |  |  |  | P:metabolism |
|  |  |  |  |  |  |  |  |  | P:primary metabolism |
|  |  |  |  |  |  |  |  |  | P:cellular metabolism |
|  |  |  |  |  |  |  |  |  | P:telomere maintenance |
|  |  |  |  |  |  |  |  |  | P:telomere organization and biogenesis |
|  |  |  |  |  |  |  |  |  | P:protein complex assembly |
|  |  |  |  |  |  |  |  |  | P:ribosomal large subunit assembly and maintenance |
|  |  |  |  |  |  |  |  |  | P:ribosomal subunit assembly |
|  |  |  |  |  |  |  |  |  | P:ribosome assembly |
|  |  |  |  |  |  |  |  |  | P:organelle organization and biogenesis |
|  |  |  |  |  |  |  |  |  | P:chromosome organization and biogenesis |
|  |  |  |  |  |  |  |  |  | P:chromosome organization and biogenesis (sensu Eukaryota) |
|  |  |  |  |  |  |  |  |  | P:regulation of biosynthesis |
|  |  |  |  |  |  |  |  |  | P:regulation of cellular biosynthesis |
|  |  |  |  |  |  |  |  |  | P:regulation of protein metabolism |
|  |  |  |  |  |  |  |  |  | P:ribosomal small subunit assembly and maintenance |
|  |  |  |  |  |  |  |  |  | P:regulation of protein biosynthesis |
|  |  |  |  |  |  |  |  |  | P:regulation of translation |
|  |  |  |  |  |  |  |  |  | P:ribosome biogenesis and assembly |
|  |  |  |  |  |  |  |  |  | P:cytoplasm organization and biogenesis |
|  |  |  |  |  |  |  |  |  | P:regulation of translational fidelity |
|  |  |  |  |  |  |  |  |  | P:physiological process |
|  |  |  |  |  |  |  |  |  | P:cellular physiological process |
|  |  |  |  |  |  |  |  |  | P:cellular process |
|  |  |  |  |  |  |  |  |  | P:translational elongation |
|  |  |  |  |  |  |  |  |  | P:biological\_process |
|
| Smp1 | Skn7 | Fhl1 | Rap1 | Sfp1 | Aft2 | Sut1 | Pdr1 | Yap5 | Molecular Function |
|  |  |  |  |  |  |  |  |  | F:structural constituent of ribosome |
|  |  |  |  |  |  |  |  |  | F:structural molecule activity |
|  |  |  |  |  |  |  |  |  | F:molecular\_function |
|  |  |  |  |  |  |  |  |  | F:translation elongation factor activity |
|
| Smp1 | Skn7 | Fhl1 | Rap1 | Sfp1 | Aft2 | Sut1 | Pdr1 | Yap5 | Cellular Component |
|  |  |  |  |  |  |  |  |  | C:nucleolus |
|  |  |  |  |  |  |  |  |  | C:organelle part |
|  |  |  |  |  |  |  |  |  | C:intracellular organelle part |
|  |  |  |  |  |  |  |  |  | C:ribosome |
|  |  |  |  |  |  |  |  |  | C:cytosol |
|  |  |  |  |  |  |  |  |  | C:ribonucleoprotein complex |
|  |  |  |  |  |  |  |  |  | C:intracellular non-membrane-bound organelle |
|  |  |  |  |  |  |  |  |  | C:non-membrane-bound organelle |
|  |  |  |  |  |  |  |  |  | C:cytoplasmic part |
|  |  |  |  |  |  |  |  |  | C:cytosolic ribosome (sensu Eukaryota) |
|  |  |  |  |  |  |  |  |  | C:large ribosomal subunit |
|  |  |  |  |  |  |  |  |  | C:cytosolic large ribosomal subunit (sensu Eukaryota) |
|  |  |  |  |  |  |  |  |  | C:protein complex |
|  |  |  |  |  |  |  |  |  | C:cytosolic part |
|  |  |  |  |  |  |  |  |  | C:small ribosomal subunit |
|  |  |  |  |  |  |  |  |  | C:cytoplasm |
|  |  |  |  |  |  |  |  |  | C:eukaryotic 43S preinitiation complex |
|  |  |  |  |  |  |  |  |  | C:eukaryotic 48S initiation complex |
|  |  |  |  |  |  |  |  |  | C:cytosolic small ribosomal subunit (sensu Eukaryota) |
|  |  |  |  |  |  |  |  |  | C:organelle |
|  |  |  |  |  |  |  |  |  | C:intracellular organelle |
|  |  |  |  |  |  |  |  |  | C:eukaryotic translation elongation factor 1 complex |
|  |  |  |  |  |  |  |  |  | C:cellular\_component |
|  |  |  |  |  |  |  |  |  | C:nucleolar part |
|  |  |  |  |  |  |  |  |  | C:small nucleolar ribonucleoprotein complex |
|  |  |  |  |  |  |  |  |  | C:cell |
|  |  |  |  |  |  |  |  |  | C:cell part |
|  |  |  |  |  |  |  |  |  | C:intracellular part |
|  |  |  |  |  |  |  |  |  | C:intracellular |
|
