# Supplementary material for: High-resolution analysis of condition-specific regulatory modules in Saccharomyces cerevisiae
Source: Genome Biol. 2008 Jan 3;9(1):R2. doi: 10.1186/gb-2008-9-1-r2 (PMC2395236; doi:10.1186/gb-2008-9-1-r2)
Supplement: Additional data file 11 — Matrices describing all EPMs and RMs, including lists of synergistic pairs of regulators. [file gb-2008-9-1-r2-S11.zip › htmls/C13_EPMs_matrix/EPM_11.RM.matrix.html]

Regulators vs. RM target gene list

|  |  |  |  |  |  |  |  |  |  |
| --- | --- | --- | --- | --- | --- | --- | --- | --- | --- |
|  | Smp1 | Skn7 | Fhl1 | Rap1 | Sfp1 | Aft2 | Sut1 | Pdr1 | Yap5 |
| RM\_1 |  |  |  |  |  |  |  |  |  |
| RM\_2 |  |  |  |  |  |  |  |  |  |
| RM\_3 |  |  |  |  |  |  |  |  |  |
| RM\_4 |  |  |  |  |  |  |  |  |  |

Synergistic Pair of Regulators

1. Fhl1\*Rap1

2. Rap1\*Sfp1

3. Fhl1\*Sfp1

4. Aft2\*Rap1

5. Aft2\*Sfp1

6. Rap1\*Skn7

7. Aft2\*Fhl1

8. Rap1\*Sut1

Matrix of enriched GO

EPM matrix
