# Supplementary material for: High-resolution analysis of condition-specific regulatory modules in Saccharomyces cerevisiae
Source: Genome Biol. 2008 Jan 3;9(1):R2. doi: 10.1186/gb-2008-9-1-r2 (PMC2395236; doi:10.1186/gb-2008-9-1-r2)
Supplement: Additional data file 11 — Matrices describing all EPMs and RMs, including lists of synergistic pairs of regulators. [file gb-2008-9-1-r2-S11.zip › htmls/C13_EPMs_matrix/EPM_14.GO_enrichment.matrix.html]

|  |  |  |  |  |  |  |  |  |  |  |  |
| --- | --- | --- | --- | --- | --- | --- | --- | --- | --- | --- | --- |
| Rox1 | Hap4 | Sip4 | Rcs1 | Ume6 | Stp1 | Rap1 | Hap1 | Mcm1 | Yap6 | Rim101 | Biological Process |
|  |  |  |  |  |  |  |  |  |  |  | P:arginyl-tRNA aminoacylation |
|  |  |  |  |  |  |  |  |  |  |  | P:development |
|  |  |  |  |  |  |  |  |  |  |  | P:mating type determination |
|  |  |  |  |  |  |  |  |  |  |  | P:sex determination |
|  |  |  |  |  |  |  |  |  |  |  | P:mating type switching |
|  |  |  |  |  |  |  |  |  |  |  | P:donor selection |
|  |  |  |  |  |  |  |  |  |  |  | P:regulation of transcription, mating-type specific |
|  |  |  |  |  |  |  |  |  |  |  | P:meiotic joint molecule formation |
|  |  |  |  |  |  |  |  |  |  |  | P:meiosis |
|  |  |  |  |  |  |  |  |  |  |  | P:meiotic cell cycle |
|  |  |  |  |  |  |  |  |  |  |  | P:m phase of meiotic cell cycle |
|  |  |  |  |  |  |  |  |  |  |  | P:protein ubiquitination |
|  |  |  |  |  |  |  |  |  |  |  | P:protein polyubiquitination |
|  |  |  |  |  |  |  |  |  |  |  | P:protein monoubiquitination |
|  |  |  |  |  |  |  |  |  |  |  | P:peptidyl-methionine modification |
|  |  |  |  |  |  |  |  |  |  |  | P:positive regulation of gluconeogenesis |
|  |  |  |  |  |  |  |  |  |  |  | P:n-terminal peptidyl-methionine acetylation |
|  |  |  |  |  |  |  |  |  |  |  | P:positive regulation of carbohydrate metabolism |
|  |  |  |  |  |  |  |  |  |  |  | P:mAPKKK cascade during cell wall biogenesis |
|  |  |  |  |  |  |  |  |  |  |  | P:meiosis I |
|  |  |  |  |  |  |  |  |  |  |  | P:m phase |
|  |  |  |  |  |  |  |  |  |  |  | P:chromosome organization and biogenesis (sensu Eukaryota) |
|  |  |  |  |  |  |  |  |  |  |  | P:nucleotide-excision repair, DNA damage recognition |
|  |  |  |  |  |  |  |  |  |  |  | P:telomere maintenance |
|  |  |  |  |  |  |  |  |  |  |  | P:telomere organization and biogenesis |
|  |  |  |  |  |  |  |  |  |  |  | P:dNA metabolism |
|  |  |  |  |  |  |  |  |  |  |  | P:dNA recombination |
|  |  |  |  |  |  |  |  |  |  |  | P:telomere maintenance via recombination |
|  |  |  |  |  |  |  |  |  |  |  | P:mitotic recombination |
|  |  |  |  |  |  |  |  |  |  |  | P:regulation of gluconeogenesis |
|  |  |  |  |  |  |  |  |  |  |  | P:synapsis |
|  |  |  |  |  |  |  |  |  |  |  | P:carbohydrate transport |
|  |  |  |  |  |  |  |  |  |  |  | P:hexose transport |
|  |  |  |  |  |  |  |  |  |  |  | P:monosaccharide transport |
|  |  |  |  |  |  |  |  |  |  |  | P:flocculation via cell wall protein-carbohydrate interaction |
|  |  |  |  |  |  |  |  |  |  |  | P:negative regulation of carbohydrate metabolism |
|  |  |  |  |  |  |  |  |  |  |  | P:flocculation |
|  |  |  |  |  |  |  |  |  |  |  | P:calcium-dependent cell-cell adhesion |
|  |  |  |  |  |  |  |  |  |  |  | P:traversing start control point of mitotic cell cycle |
|  |  |  |  |  |  |  |  |  |  |  | P:negative regulation of gluconeogenesis |
|
| Rox1 | Hap4 | Sip4 | Rcs1 | Ume6 | Stp1 | Rap1 | Hap1 | Mcm1 | Yap6 | Rim101 | Molecular Function |
|  |  |  |  |  |  |  |  |  |  |  | F:dNA binding |
|  |  |  |  |  |  |  |  |  |  |  | F:arginine-tRNA ligase activity |
|  |  |  |  |  |  |  |  |  |  |  | F:telomeric DNA binding |
|  |  |  |  |  |  |  |  |  |  |  | F:binding |
|  |  |  |  |  |  |  |  |  |  |  | F:protein binding |
|  |  |  |  |  |  |  |  |  |  |  | F:small protein activating enzyme activity |
|  |  |  |  |  |  |  |  |  |  |  | F:ligase activity |
|  |  |  |  |  |  |  |  |  |  |  | F:uRM1 activating enzyme activity |
|  |  |  |  |  |  |  |  |  |  |  | F:transcriptional repressor activity |
|  |  |  |  |  |  |  |  |  |  |  | F:transcription factor binding |
|  |  |  |  |  |  |  |  |  |  |  | F:transcription corepressor activity |
|  |  |  |  |  |  |  |  |  |  |  | F:transcription cofactor activity |
|  |  |  |  |  |  |  |  |  |  |  | F:aTP-dependent protein binding |
|  |  |  |  |  |  |  |  |  |  |  | F:ubiquitin conjugating enzyme activity |
|  |  |  |  |  |  |  |  |  |  |  | F:small protein conjugating enzyme activity |
|  |  |  |  |  |  |  |  |  |  |  | F:bis(5'-nucleosyl)-tetraphosphatase activity |
|  |  |  |  |  |  |  |  |  |  |  | F:phosphatidylinositol phosphate kinase activity |
|  |  |  |  |  |  |  |  |  |  |  | F:bis(5'-nucleosyl)-tetraphosphatase (asymmetrical) activity |
|  |  |  |  |  |  |  |  |  |  |  | F:1-phosphatidylinositol-3-phosphate 5-kinase activity |
|  |  |  |  |  |  |  |  |  |  |  | F:small conjugating protein binding |
|  |  |  |  |  |  |  |  |  |  |  | F:sUMO polymer binding |
|  |  |  |  |  |  |  |  |  |  |  | F:sUMO binding |
|  |  |  |  |  |  |  |  |  |  |  | F:carbohydrate transporter activity |
|  |  |  |  |  |  |  |  |  |  |  | F:sugar transporter activity |
|  |  |  |  |  |  |  |  |  |  |  | F:monosaccharide transporter activity |
|  |  |  |  |  |  |  |  |  |  |  | F:hexose transporter activity |
|  |  |  |  |  |  |  |  |  |  |  | F:mannose binding |
|  |  |  |  |  |  |  |  |  |  |  | F:carbohydrate binding |
|  |  |  |  |  |  |  |  |  |  |  | F:sugar binding |
|  |  |  |  |  |  |  |  |  |  |  | F:monosaccharide binding |
|  |  |  |  |  |  |  |  |  |  |  | F:galactose transporter activity |
|  |  |  |  |  |  |  |  |  |  |  | F:damaged DNA binding |
|  |  |  |  |  |  |  |  |  |  |  | F:dNA helicase activity |
|  |  |  |  |  |  |  |  |  |  |  | F:helicase activity |
|  |  |  |  |  |  |  |  |  |  |  | F:sterol O-acyltransferase activity |
|  |  |  |  |  |  |  |  |  |  |  | F:zinc ion binding |
|
| Rox1 | Hap4 | Sip4 | Rcs1 | Ume6 | Stp1 | Rap1 | Hap1 | Mcm1 | Yap6 | Rim101 | Cellular Component |
|  |  |  |  |  |  |  |  |  |  |  | C:nuclear telomere cap complex |
|  |  |  |  |  |  |  |  |  |  |  | C:telomere cap complex |
|  |  |  |  |  |  |  |  |  |  |  | C:sCF ubiquitin ligase complex |
|  |  |  |  |  |  |  |  |  |  |  | C:cullin-RING ubiquitin ligase complex |
|  |  |  |  |  |  |  |  |  |  |  | C:transverse filament |
|  |  |  |  |  |  |  |  |  |  |  | C:synaptonemal complex |
|  |  |  |  |  |  |  |  |  |  |  | C:nucleotide-excision repair factor 1 complex |
|  |  |  |  |  |  |  |  |  |  |  | C:natB complex |
|  |  |  |  |  |  |  |  |  |  |  | C:ubiquitin conjugating enzyme complex |
|
