# Supplementary material for: High-resolution analysis of condition-specific regulatory modules in Saccharomyces cerevisiae
Source: Genome Biol. 2008 Jan 3;9(1):R2. doi: 10.1186/gb-2008-9-1-r2 (PMC2395236; doi:10.1186/gb-2008-9-1-r2)
Supplement: Additional data file 11 — Matrices describing all EPMs and RMs, including lists of synergistic pairs of regulators. [file gb-2008-9-1-r2-S11.zip › htmls/C13_EPMs_matrix/EPM_14.Overlap.matrix.html]

|  |  |  |  |  |  |  |  |  |  |  |
| --- | --- | --- | --- | --- | --- | --- | --- | --- | --- | --- |
| Rox1 | Hap4 | Sip4 | Rcs1 | Ume6 | Stp1 | Rap1 | Hap1 | Mcm1 | Yap6 | Rim101 |
|  |  |  |  |  |  |  |  |  |  |  | Rox1 |
|  |  |  |  |  |  |  |  |  |  |  | Hap4 |
|  |  |  |  |  |  |  |  |  |  |  | Sip4 |
|  |  |  |  |  |  |  |  |  |  |  | Rcs1 |
|  |  |  |  |  |  |  |  |  |  |  | Ume6 |
|  |  |  |  |  |  |  |  |  |  |  | Stp1 |
|  |  |  |  |  |  |  |  |  |  |  | Rap1 |
|  |  |  |  |  |  |  |  |  |  |  | Hap1 |
|  |  |  |  |  |  |  |  |  |  |  | Mcm1 |
|  |  |  |  |  |  |  |  |  |  |  | Yap6 |
|  |  |  |  |  |  |  |  |  |  |  | Rim101 |
 Rox1 | Hap4 | Sip4 | Rcs1 | Ume6 | Stp1 | Rap1 | Hap1 | Mcm1 | Yap6 | Rim101 |
