# Supplementary material for: High-resolution analysis of condition-specific regulatory modules in Saccharomyces cerevisiae
Source: Genome Biol. 2008 Jan 3;9(1):R2. doi: 10.1186/gb-2008-9-1-r2 (PMC2395236; doi:10.1186/gb-2008-9-1-r2)
Supplement: Additional data file 11 — Matrices describing all EPMs and RMs, including lists of synergistic pairs of regulators. [file gb-2008-9-1-r2-S11.zip › htmls/C13_EPMs_matrix/EPM_15.GO_enrichment.matrix.html]

|  |  |  |  |  |  |  |  |  |  |  |  |  |  |  |  |  |  |  |  |  |
| --- | --- | --- | --- | --- | --- | --- | --- | --- | --- | --- | --- | --- | --- | --- | --- | --- | --- | --- | --- | --- |
| Phd1 | Ume6 | Nrg1 | Msn4 | Cad1 | Yap6 | Yap5 | Rcs1 | Dal80 | Rox1 | Gzf3 | Gat3 | Mcm1 | Dat1 | Swi5 | Hap1 | Rap1 | Hap4 | Hap2 | Hap3 | Biological Process |
|  |  |  |  |  |  |  |  |  |  |  |  |  |  |  |  |  |  |  |  | P:beta-glucan metabolism |
|  |  |  |  |  |  |  |  |  |  |  |  |  |  |  |  |  |  |  |  | P:meiotic DNA double-strand break formation |
|  |  |  |  |  |  |  |  |  |  |  |  |  |  |  |  |  |  |  |  | P:beta-glucan biosynthesis |
|  |  |  |  |  |  |  |  |  |  |  |  |  |  |  |  |  |  |  |  | P:1,6-beta-glucan biosynthesis |
|  |  |  |  |  |  |  |  |  |  |  |  |  |  |  |  |  |  |  |  | P:1,6-beta-glucan metabolism |
|  |  |  |  |  |  |  |  |  |  |  |  |  |  |  |  |  |  |  |  | P:prospore formation |
|  |  |  |  |  |  |  |  |  |  |  |  |  |  |  |  |  |  |  |  | P:high-affinity zinc ion transport |
|  |  |  |  |  |  |  |  |  |  |  |  |  |  |  |  |  |  |  |  | P:protein deneddylation |
|  |  |  |  |  |  |  |  |  |  |  |  |  |  |  |  |  |  |  |  | P:macroautophagy |
|  |  |  |  |  |  |  |  |  |  |  |  |  |  |  |  |  |  |  |  | P:vesicle docking |
|  |  |  |  |  |  |  |  |  |  |  |  |  |  |  |  |  |  |  |  | P:autophagic vacuole fusion |
|  |  |  |  |  |  |  |  |  |  |  |  |  |  |  |  |  |  |  |  | P:organic anion transport |
|  |  |  |  |  |  |  |  |  |  |  |  |  |  |  |  |  |  |  |  | P:organophosphate ester transport |
|  |  |  |  |  |  |  |  |  |  |  |  |  |  |  |  |  |  |  |  | P:glycerophosphodiester transport |
|  |  |  |  |  |  |  |  |  |  |  |  |  |  |  |  |  |  |  |  | P:traversing start control point of mitotic cell cycle |
|  |  |  |  |  |  |  |  |  |  |  |  |  |  |  |  |  |  |  |  | P:rRNA methylation |
|  |  |  |  |  |  |  |  |  |  |  |  |  |  |  |  |  |  |  |  | P:response to arsenic |
|  |  |  |  |  |  |  |  |  |  |  |  |  |  |  |  |  |  |  |  | P:dNA recombination |
|  |  |  |  |  |  |  |  |  |  |  |  |  |  |  |  |  |  |  |  | P:telomere maintenance via recombination |
|  |  |  |  |  |  |  |  |  |  |  |  |  |  |  |  |  |  |  |  | P:mitotic recombination |
|  |  |  |  |  |  |  |  |  |  |  |  |  |  |  |  |  |  |  |  | P:glutamine family amino acid metabolism |
|  |  |  |  |  |  |  |  |  |  |  |  |  |  |  |  |  |  |  |  | P:proline catabolism to glutamate |
|  |  |  |  |  |  |  |  |  |  |  |  |  |  |  |  |  |  |  |  | P:glutamine family amino acid biosynthesis |
|  |  |  |  |  |  |  |  |  |  |  |  |  |  |  |  |  |  |  |  | P:glutamate biosynthesis |
|  |  |  |  |  |  |  |  |  |  |  |  |  |  |  |  |  |  |  |  | P:glutamate metabolism |
|  |  |  |  |  |  |  |  |  |  |  |  |  |  |  |  |  |  |  |  | P:dicarboxylic acid transport |
|  |  |  |  |  |  |  |  |  |  |  |  |  |  |  |  |  |  |  |  | P:fumarate transport |
|  |  |  |  |  |  |  |  |  |  |  |  |  |  |  |  |  |  |  |  | P:succinate transport |
|  |  |  |  |  |  |  |  |  |  |  |  |  |  |  |  |  |  |  |  | P:transcription from mitochondrial promoter |
|  |  |  |  |  |  |  |  |  |  |  |  |  |  |  |  |  |  |  |  | P:transcription |
|  |  |  |  |  |  |  |  |  |  |  |  |  |  |  |  |  |  |  |  | P:organelle localization |
|  |  |  |  |  |  |  |  |  |  |  |  |  |  |  |  |  |  |  |  | P:peptidyl-methionine modification |
|  |  |  |  |  |  |  |  |  |  |  |  |  |  |  |  |  |  |  |  | P:n-terminal peptidyl-methionine acetylation |
|  |  |  |  |  |  |  |  |  |  |  |  |  |  |  |  |  |  |  |  | P:transcription, DNA-dependent |
|  |  |  |  |  |  |  |  |  |  |  |  |  |  |  |  |  |  |  |  | P:mating type determination |
|  |  |  |  |  |  |  |  |  |  |  |  |  |  |  |  |  |  |  |  | P:sex determination |
|  |  |  |  |  |  |  |  |  |  |  |  |  |  |  |  |  |  |  |  | P:aldehyde metabolism |
|  |  |  |  |  |  |  |  |  |  |  |  |  |  |  |  |  |  |  |  | P:mating type switching |
|  |  |  |  |  |  |  |  |  |  |  |  |  |  |  |  |  |  |  |  | P:donor selection |
|  |  |  |  |  |  |  |  |  |  |  |  |  |  |  |  |  |  |  |  | P:regulation of transcription, mating-type specific |
|  |  |  |  |  |  |  |  |  |  |  |  |  |  |  |  |  |  |  |  | P:transcription from RNA polymerase II promoter |
|  |  |  |  |  |  |  |  |  |  |  |  |  |  |  |  |  |  |  |  | P:drug transport |
|  |  |  |  |  |  |  |  |  |  |  |  |  |  |  |  |  |  |  |  | P:response to drug |
|
| Phd1 | Ume6 | Nrg1 | Msn4 | Cad1 | Yap6 | Yap5 | Rcs1 | Dal80 | Rox1 | Gzf3 | Gat3 | Mcm1 | Dat1 | Swi5 | Hap1 | Rap1 | Hap4 | Hap2 | Hap3 | Molecular Function |
|  |  |  |  |  |  |  |  |  |  |  |  |  |  |  |  |  |  |  |  | F:centromeric DNA binding |
|  |  |  |  |  |  |  |  |  |  |  |  |  |  |  |  |  |  |  |  | F:ribonuclease MRP activity |
|  |  |  |  |  |  |  |  |  |  |  |  |  |  |  |  |  |  |  |  | F:3',5'-cyclic-AMP phosphodiesterase activity |
|  |  |  |  |  |  |  |  |  |  |  |  |  |  |  |  |  |  |  |  | F:inositol polyphosphate multikinase activity |
|  |  |  |  |  |  |  |  |  |  |  |  |  |  |  |  |  |  |  |  | F:3',5'-cyclic-nucleotide phosphodiesterase activity |
|  |  |  |  |  |  |  |  |  |  |  |  |  |  |  |  |  |  |  |  | F:alcohol dehydrogenase (NADP+) activity |
|  |  |  |  |  |  |  |  |  |  |  |  |  |  |  |  |  |  |  |  | F:aldo-keto reductase activity |
|  |  |  |  |  |  |  |  |  |  |  |  |  |  |  |  |  |  |  |  | F:t-SNARE activity |
|  |  |  |  |  |  |  |  |  |  |  |  |  |  |  |  |  |  |  |  | F:high affinity zinc uptake transporter activity |
|  |  |  |  |  |  |  |  |  |  |  |  |  |  |  |  |  |  |  |  | F:zinc ion transporter activity |
|  |  |  |  |  |  |  |  |  |  |  |  |  |  |  |  |  |  |  |  | F:rRNA methyltransferase activity |
|  |  |  |  |  |  |  |  |  |  |  |  |  |  |  |  |  |  |  |  | F:rRNA (uridine-2'-O-)-methyltransferase activity |
|  |  |  |  |  |  |  |  |  |  |  |  |  |  |  |  |  |  |  |  | F:arsenate reductase activity |
|  |  |  |  |  |  |  |  |  |  |  |  |  |  |  |  |  |  |  |  | F:rRNA (uridine) methyltransferase activity |
|  |  |  |  |  |  |  |  |  |  |  |  |  |  |  |  |  |  |  |  | F:mAP kinase activity |
|  |  |  |  |  |  |  |  |  |  |  |  |  |  |  |  |  |  |  |  | F:dNA helicase activity |
|  |  |  |  |  |  |  |  |  |  |  |  |  |  |  |  |  |  |  |  | F:helicase activity |
|  |  |  |  |  |  |  |  |  |  |  |  |  |  |  |  |  |  |  |  | F:malate synthase activity |
|  |  |  |  |  |  |  |  |  |  |  |  |  |  |  |  |  |  |  |  | F:transcription regulator activity |
|  |  |  |  |  |  |  |  |  |  |  |  |  |  |  |  |  |  |  |  | F:transcriptional repressor activity |
|  |  |  |  |  |  |  |  |  |  |  |  |  |  |  |  |  |  |  |  | F:transcription factor binding |
|  |  |  |  |  |  |  |  |  |  |  |  |  |  |  |  |  |  |  |  | F:transcription cofactor activity |
|  |  |  |  |  |  |  |  |  |  |  |  |  |  |  |  |  |  |  |  | F:aryl-alcohol dehydrogenase activity |
|  |  |  |  |  |  |  |  |  |  |  |  |  |  |  |  |  |  |  |  | F:transcription corepressor activity |
|  |  |  |  |  |  |  |  |  |  |  |  |  |  |  |  |  |  |  |  | F:four-way junction DNA binding |
|  |  |  |  |  |  |  |  |  |  |  |  |  |  |  |  |  |  |  |  | F:dNA secondary structure binding |
|  |  |  |  |  |  |  |  |  |  |  |  |  |  |  |  |  |  |  |  | F:organic anion transporter activity |
|  |  |  |  |  |  |  |  |  |  |  |  |  |  |  |  |  |  |  |  | F:glycerophosphodiester transporter activity |
|  |  |  |  |  |  |  |  |  |  |  |  |  |  |  |  |  |  |  |  | F:organophosphate ester transporter activity |
|  |  |  |  |  |  |  |  |  |  |  |  |  |  |  |  |  |  |  |  | F:dicarboxylic acid transporter activity |
|  |  |  |  |  |  |  |  |  |  |  |  |  |  |  |  |  |  |  |  | F:succinate transporter activity |
|  |  |  |  |  |  |  |  |  |  |  |  |  |  |  |  |  |  |  |  | F:fumarate transporter activity |
|  |  |  |  |  |  |  |  |  |  |  |  |  |  |  |  |  |  |  |  | F:succinate:fumarate antiporter activity |
|  |  |  |  |  |  |  |  |  |  |  |  |  |  |  |  |  |  |  |  | F:isocitrate dehydrogenase (NADP+) activity |
|  |  |  |  |  |  |  |  |  |  |  |  |  |  |  |  |  |  |  |  | F:adenine phosphoribosyltransferase activity |
|  |  |  |  |  |  |  |  |  |  |  |  |  |  |  |  |  |  |  |  | F:phosphatidylinositol-3,4-bisphosphate binding |
|  |  |  |  |  |  |  |  |  |  |  |  |  |  |  |  |  |  |  |  | F:proline dehydrogenase activity |
|
| Phd1 | Ume6 | Nrg1 | Msn4 | Cad1 | Yap6 | Yap5 | Rcs1 | Dal80 | Rox1 | Gzf3 | Gat3 | Mcm1 | Dat1 | Swi5 | Hap1 | Rap1 | Hap4 | Hap2 | Hap3 | Cellular Component |
|  |  |  |  |  |  |  |  |  |  |  |  |  |  |  |  |  |  |  |  | C:gINS complex |
|  |  |  |  |  |  |  |  |  |  |  |  |  |  |  |  |  |  |  |  | C:nucleolar ribonuclease P complex |
|  |  |  |  |  |  |  |  |  |  |  |  |  |  |  |  |  |  |  |  | C:ribonuclease P complex |
|  |  |  |  |  |  |  |  |  |  |  |  |  |  |  |  |  |  |  |  | C:condensed nuclear chromosome |
|  |  |  |  |  |  |  |  |  |  |  |  |  |  |  |  |  |  |  |  | C:condensed chromosome |
|  |  |  |  |  |  |  |  |  |  |  |  |  |  |  |  |  |  |  |  | C:recQ helicase-Topo III complex |
|  |  |  |  |  |  |  |  |  |  |  |  |  |  |  |  |  |  |  |  | C:m-AAA complex |
|  |  |  |  |  |  |  |  |  |  |  |  |  |  |  |  |  |  |  |  | C:major (U2-dependent) spliceosome |
|  |  |  |  |  |  |  |  |  |  |  |  |  |  |  |  |  |  |  |  | C:snRNP U2 |
|  |  |  |  |  |  |  |  |  |  |  |  |  |  |  |  |  |  |  |  | C:natB complex |
|  |  |  |  |  |  |  |  |  |  |  |  |  |  |  |  |  |  |  |  | C:transcription factor TFIIA complex |
|  |  |  |  |  |  |  |  |  |  |  |  |  |  |  |  |  |  |  |  | C:external side of endosome membrane |
|  |  |  |  |  |  |  |  |  |  |  |  |  |  |  |  |  |  |  |  | C:small nuclear ribonucleoprotein complex |
|
