# Supplementary material for: High-resolution analysis of condition-specific regulatory modules in Saccharomyces cerevisiae
Source: Genome Biol. 2008 Jan 3;9(1):R2. doi: 10.1186/gb-2008-9-1-r2 (PMC2395236; doi:10.1186/gb-2008-9-1-r2)
Supplement: Additional data file 11 — Matrices describing all EPMs and RMs, including lists of synergistic pairs of regulators. [file gb-2008-9-1-r2-S11.zip › htmls/C13_EPMs_matrix/EPM_15.Overlap.matrix.html]

|  |  |  |  |  |  |  |  |  |  |  |  |  |  |  |  |  |  |  |  |
| --- | --- | --- | --- | --- | --- | --- | --- | --- | --- | --- | --- | --- | --- | --- | --- | --- | --- | --- | --- |
| Phd1 | Ume6 | Nrg1 | Msn4 | Cad1 | Yap6 | Yap5 | Rcs1 | Dal80 | Rox1 | Gzf3 | Gat3 | Mcm1 | Dat1 | Swi5 | Hap1 | Rap1 | Hap4 | Hap2 | Hap3 |
|  |  |  |  |  |  |  |  |  |  |  |  |  |  |  |  |  |  |  |  | Phd1 |
|  |  |  |  |  |  |  |  |  |  |  |  |  |  |  |  |  |  |  |  | Ume6 |
|  |  |  |  |  |  |  |  |  |  |  |  |  |  |  |  |  |  |  |  | Nrg1 |
|  |  |  |  |  |  |  |  |  |  |  |  |  |  |  |  |  |  |  |  | Msn4 |
|  |  |  |  |  |  |  |  |  |  |  |  |  |  |  |  |  |  |  |  | Cad1 |
|  |  |  |  |  |  |  |  |  |  |  |  |  |  |  |  |  |  |  |  | Yap6 |
|  |  |  |  |  |  |  |  |  |  |  |  |  |  |  |  |  |  |  |  | Yap5 |
|  |  |  |  |  |  |  |  |  |  |  |  |  |  |  |  |  |  |  |  | Rcs1 |
|  |  |  |  |  |  |  |  |  |  |  |  |  |  |  |  |  |  |  |  | Dal80 |
|  |  |  |  |  |  |  |  |  |  |  |  |  |  |  |  |  |  |  |  | Rox1 |
|  |  |  |  |  |  |  |  |  |  |  |  |  |  |  |  |  |  |  |  | Gzf3 |
|  |  |  |  |  |  |  |  |  |  |  |  |  |  |  |  |  |  |  |  | Gat3 |
|  |  |  |  |  |  |  |  |  |  |  |  |  |  |  |  |  |  |  |  | Mcm1 |
|  |  |  |  |  |  |  |  |  |  |  |  |  |  |  |  |  |  |  |  | Dat1 |
|  |  |  |  |  |  |  |  |  |  |  |  |  |  |  |  |  |  |  |  | Swi5 |
|  |  |  |  |  |  |  |  |  |  |  |  |  |  |  |  |  |  |  |  | Hap1 |
|  |  |  |  |  |  |  |  |  |  |  |  |  |  |  |  |  |  |  |  | Rap1 |
|  |  |  |  |  |  |  |  |  |  |  |  |  |  |  |  |  |  |  |  | Hap4 |
|  |  |  |  |  |  |  |  |  |  |  |  |  |  |  |  |  |  |  |  | Hap2 |
|  |  |  |  |  |  |  |  |  |  |  |  |  |  |  |  |  |  |  |  | Hap3 |
 Phd1 | Ume6 | Nrg1 | Msn4 | Cad1 | Yap6 | Yap5 | Rcs1 | Dal80 | Rox1 | Gzf3 | Gat3 | Mcm1 | Dat1 | Swi5 | Hap1 | Rap1 | Hap4 | Hap2 | Hap3 |
