# Supplementary material for: High-resolution analysis of condition-specific regulatory modules in Saccharomyces cerevisiae
Source: Genome Biol. 2008 Jan 3;9(1):R2. doi: 10.1186/gb-2008-9-1-r2 (PMC2395236; doi:10.1186/gb-2008-9-1-r2)
Supplement: Additional data file 11 — Matrices describing all EPMs and RMs, including lists of synergistic pairs of regulators. [file gb-2008-9-1-r2-S11.zip › htmls/C13_EPMs_matrix/EPM_16.GO_enrichment.matrix.html]

|  |  |  |
| --- | --- | --- |
| Aft2 | Nrg1 | Biological Process |
|  |  | P:water-soluble vitamin metabolism |
|  |  | P:vitamin metabolism |
|  |  | P:vitamin biosynthesis |
|  |  | P:water-soluble vitamin biosynthesis |
|  |  | P:thiamin and derivative metabolism |
|  |  | P:thiamin and derivative biosynthesis |
|  |  | P:thiamin metabolism |
|  |  | P:thiamin biosynthesis |
|  |  | P:vitamin B6 metabolism |
|  |  | P:pyridoxine metabolism |
|  |  | P:dicarboxylic acid transport |
|  |  | P:response to radiation |
|  |  | P:succinate transport |
|  |  | P:fumarate transport |
|  |  | P:response to UV |
|  |  | P:response to light stimulus |
|  |  | P:response to UV-B |
|  |  | P:response to UV-C |
|
| Aft2 | Nrg1 | Molecular Function |
|  |  | F:phosphate transporter activity |
|  |  | F:symporter activity |
|  |  | F:anion:cation symporter activity |
|  |  | F:sodium:inorganic phosphate symporter activity |
|  |  | F:sodium:phosphate symporter activity |
|  |  | F:carrier activity |
|  |  | F:dicarboxylic acid transporter activity |
|  |  | F:succinate transporter activity |
|  |  | F:succinate:fumarate antiporter activity |
|  |  | F:fumarate transporter activity |
|  |  | F:electrochemical potential-driven transporter activity |
|  |  | F:porter activity |
|
| Aft2 | Nrg1 | Cellular Component |
|  |  | C:cellular component unknown |
|
