# Supplementary material for: High-resolution analysis of condition-specific regulatory modules in Saccharomyces cerevisiae
Source: Genome Biol. 2008 Jan 3;9(1):R2. doi: 10.1186/gb-2008-9-1-r2 (PMC2395236; doi:10.1186/gb-2008-9-1-r2)
Supplement: Additional data file 11 — Matrices describing all EPMs and RMs, including lists of synergistic pairs of regulators. [file gb-2008-9-1-r2-S11.zip › htmls/C13_EPMs_matrix/EPM_17.GO_enrichment.matrix.html]

|  |  |  |  |  |  |  |  |  |  |  |  |  |  |  |  |  |
| --- | --- | --- | --- | --- | --- | --- | --- | --- | --- | --- | --- | --- | --- | --- | --- | --- |
| Hsf1 | Aft2 | Gln3 | Yap7 | Cad1 | Azf1 | Gal4 | Msn2 | Msn4 | Rph1 | Sut1 | Skn7 | Phd1 | Ume6 | Stp1 | Put3 | Biological Process |
|  |  |  |  |  |  |  |  |  |  |  |  |  |  |  |  | P:glucan catabolism |
|  |  |  |  |  |  |  |  |  |  |  |  |  |  |  |  | P:response to arsenic |
|  |  |  |  |  |  |  |  |  |  |  |  |  |  |  |  | P:glycogen catabolism |
|  |  |  |  |  |  |  |  |  |  |  |  |  |  |  |  | P:phosphate metabolism |
|  |  |  |  |  |  |  |  |  |  |  |  |  |  |  |  | P:phosphorus metabolism |
|  |  |  |  |  |  |  |  |  |  |  |  |  |  |  |  | P:mAPKKK cascade during cell wall biogenesis |
|  |  |  |  |  |  |  |  |  |  |  |  |  |  |  |  | P:fAD transport |
|  |  |  |  |  |  |  |  |  |  |  |  |  |  |  |  | P:mitochondrial electron transport, succinate to ubiquinone |
|  |  |  |  |  |  |  |  |  |  |  |  |  |  |  |  | P:c-terminal protein lipidation |
|  |  |  |  |  |  |  |  |  |  |  |  |  |  |  |  | P:oxidative phosphorylation |
|  |  |  |  |  |  |  |  |  |  |  |  |  |  |  |  | P:phosphorylation |
|  |  |  |  |  |  |  |  |  |  |  |  |  |  |  |  | P:response to stimulus |
|  |  |  |  |  |  |  |  |  |  |  |  |  |  |  |  | P:pyrimidine nucleoside triphosphate metabolism |
|  |  |  |  |  |  |  |  |  |  |  |  |  |  |  |  | P:nucleotide metabolism |
|  |  |  |  |  |  |  |  |  |  |  |  |  |  |  |  | P:azole transport |
|  |  |  |  |  |  |  |  |  |  |  |  |  |  |  |  | P:cTP biosynthesis |
|  |  |  |  |  |  |  |  |  |  |  |  |  |  |  |  | P:pyrimidine ribonucleotide biosynthesis |
|  |  |  |  |  |  |  |  |  |  |  |  |  |  |  |  | P:pyrimidine ribonucleotide metabolism |
|  |  |  |  |  |  |  |  |  |  |  |  |  |  |  |  | P:pyrimidine ribonucleoside triphosphate biosynthesis |
|  |  |  |  |  |  |  |  |  |  |  |  |  |  |  |  | P:pyrimidine ribonucleoside triphosphate metabolism |
|  |  |  |  |  |  |  |  |  |  |  |  |  |  |  |  | P:cTP metabolism |
|  |  |  |  |  |  |  |  |  |  |  |  |  |  |  |  | P:sulfur compound catabolism |
|  |  |  |  |  |  |  |  |  |  |  |  |  |  |  |  | P:glutathione catabolism |
|  |  |  |  |  |  |  |  |  |  |  |  |  |  |  |  | P:chaperone cofactor-dependent protein folding |
|  |  |  |  |  |  |  |  |  |  |  |  |  |  |  |  | P:posttranslational protein folding |
|  |  |  |  |  |  |  |  |  |  |  |  |  |  |  |  | P:protein complex assembly |
|  |  |  |  |  |  |  |  |  |  |  |  |  |  |  |  | P:proteasome assembly |
|  |  |  |  |  |  |  |  |  |  |  |  |  |  |  |  | P:'de novo' protein folding |
|  |  |  |  |  |  |  |  |  |  |  |  |  |  |  |  | P:siderophore transport |
|  |  |  |  |  |  |  |  |  |  |  |  |  |  |  |  | P:biological\_process |
|  |  |  |  |  |  |  |  |  |  |  |  |  |  |  |  | P:response to stress |
|  |  |  |  |  |  |  |  |  |  |  |  |  |  |  |  | P:protein refolding |
|  |  |  |  |  |  |  |  |  |  |  |  |  |  |  |  | P:protein folding |
|  |  |  |  |  |  |  |  |  |  |  |  |  |  |  |  | P:protein-heme linkage |
|  |  |  |  |  |  |  |  |  |  |  |  |  |  |  |  | P:cytochrome c-heme linkage |
|  |  |  |  |  |  |  |  |  |  |  |  |  |  |  |  | P:protein-tetrapyrrole linkage |
|  |  |  |  |  |  |  |  |  |  |  |  |  |  |  |  | P:monosaccharide transport |
|  |  |  |  |  |  |  |  |  |  |  |  |  |  |  |  | P:hexose transport |
|  |  |  |  |  |  |  |  |  |  |  |  |  |  |  |  | P:response to oxidative stress |
|  |  |  |  |  |  |  |  |  |  |  |  |  |  |  |  | P:oxygen and reactive oxygen species metabolism |
|  |  |  |  |  |  |  |  |  |  |  |  |  |  |  |  | P:coenzyme transport |
|  |  |  |  |  |  |  |  |  |  |  |  |  |  |  |  | P:coenzyme A transport |
|  |  |  |  |  |  |  |  |  |  |  |  |  |  |  |  | P:cofactor transport |
|  |  |  |  |  |  |  |  |  |  |  |  |  |  |  |  | P:vacuole fusion, non-autophagic |
|
| Hsf1 | Aft2 | Gln3 | Yap7 | Cad1 | Azf1 | Gal4 | Msn2 | Msn4 | Rph1 | Sut1 | Skn7 | Phd1 | Ume6 | Stp1 | Put3 | Molecular Function |
|  |  |  |  |  |  |  |  |  |  |  |  |  |  |  |  | F:aspartic-type endopeptidase activity |
|  |  |  |  |  |  |  |  |  |  |  |  |  |  |  |  | F:aromatic-amino-acid transaminase activity |
|  |  |  |  |  |  |  |  |  |  |  |  |  |  |  |  | F:cTP synthase activity |
|  |  |  |  |  |  |  |  |  |  |  |  |  |  |  |  | F:d-lactaldehyde dehydrogenase activity |
|  |  |  |  |  |  |  |  |  |  |  |  |  |  |  |  | F:protein-glutamine gamma-glutamyltransferase activity |
|  |  |  |  |  |  |  |  |  |  |  |  |  |  |  |  | F:azole transporter activity |
|  |  |  |  |  |  |  |  |  |  |  |  |  |  |  |  | F:nicotinate-nucleotide adenylyltransferase activity |
|  |  |  |  |  |  |  |  |  |  |  |  |  |  |  |  | F:protein binding |
|  |  |  |  |  |  |  |  |  |  |  |  |  |  |  |  | F:aTPase activity, coupled |
|  |  |  |  |  |  |  |  |  |  |  |  |  |  |  |  | F:hydrolase activity, acting on acid anhydrides, in phosphorus-containing anhydrides |
|  |  |  |  |  |  |  |  |  |  |  |  |  |  |  |  | F:hydrolase activity, acting on acid anhydrides |
|  |  |  |  |  |  |  |  |  |  |  |  |  |  |  |  | F:pyrophosphatase activity |
|  |  |  |  |  |  |  |  |  |  |  |  |  |  |  |  | F:aTPase activity |
|  |  |  |  |  |  |  |  |  |  |  |  |  |  |  |  | F:nucleoside-triphosphatase activity |
|  |  |  |  |  |  |  |  |  |  |  |  |  |  |  |  | F:unfolded protein binding |
|  |  |  |  |  |  |  |  |  |  |  |  |  |  |  |  | F:molecular\_function |
|  |  |  |  |  |  |  |  |  |  |  |  |  |  |  |  | F:phosphatase activator activity |
|  |  |  |  |  |  |  |  |  |  |  |  |  |  |  |  | F:mAP kinase phosphatase activity |
|  |  |  |  |  |  |  |  |  |  |  |  |  |  |  |  | F:fAD transporter activity |
|  |  |  |  |  |  |  |  |  |  |  |  |  |  |  |  | F:aromatic amino acid transporter activity |
|  |  |  |  |  |  |  |  |  |  |  |  |  |  |  |  | F:l-isoleucine transporter activity |
|  |  |  |  |  |  |  |  |  |  |  |  |  |  |  |  | F:l-glutamine transporter activity |
|  |  |  |  |  |  |  |  |  |  |  |  |  |  |  |  | F:protein tyrosine/serine/threonine phosphatase activity |
|  |  |  |  |  |  |  |  |  |  |  |  |  |  |  |  | F:l-tyrosine transporter activity |
|  |  |  |  |  |  |  |  |  |  |  |  |  |  |  |  | F:tetrapyrrole binding |
|  |  |  |  |  |  |  |  |  |  |  |  |  |  |  |  | F:heme binding |
|  |  |  |  |  |  |  |  |  |  |  |  |  |  |  |  | F:endopeptidase inhibitor activity |
|  |  |  |  |  |  |  |  |  |  |  |  |  |  |  |  | F:protease inhibitor activity |
|  |  |  |  |  |  |  |  |  |  |  |  |  |  |  |  | F:enzyme inhibitor activity |
|  |  |  |  |  |  |  |  |  |  |  |  |  |  |  |  | F:cAMP-dependent protein kinase inhibitor activity |
|  |  |  |  |  |  |  |  |  |  |  |  |  |  |  |  | F:antioxidant activity |
|  |  |  |  |  |  |  |  |  |  |  |  |  |  |  |  | F:oxidoreductase activity, acting on peroxide as acceptor |
|  |  |  |  |  |  |  |  |  |  |  |  |  |  |  |  | F:cofactor transporter activity |
|  |  |  |  |  |  |  |  |  |  |  |  |  |  |  |  | F:peroxidase activity |
|  |  |  |  |  |  |  |  |  |  |  |  |  |  |  |  | F:holocytochrome-c synthase activity |
|  |  |  |  |  |  |  |  |  |  |  |  |  |  |  |  | F:catalase activity |
|  |  |  |  |  |  |  |  |  |  |  |  |  |  |  |  | F:protein-tyrosine kinase activity |
|  |  |  |  |  |  |  |  |  |  |  |  |  |  |  |  | F:coenzyme transporter activity |
|  |  |  |  |  |  |  |  |  |  |  |  |  |  |  |  | F:coenzyme A transporter activity |
|  |  |  |  |  |  |  |  |  |  |  |  |  |  |  |  | F:glucan 1,4-alpha-glucosidase activity |
|  |  |  |  |  |  |  |  |  |  |  |  |  |  |  |  | F:hexose transporter activity |
|  |  |  |  |  |  |  |  |  |  |  |  |  |  |  |  | F:monosaccharide transporter activity |
|  |  |  |  |  |  |  |  |  |  |  |  |  |  |  |  | F:glucose transporter activity |
|  |  |  |  |  |  |  |  |  |  |  |  |  |  |  |  | F:fructose transporter activity |
|  |  |  |  |  |  |  |  |  |  |  |  |  |  |  |  | F:mannose transporter activity |
|
| Hsf1 | Aft2 | Gln3 | Yap7 | Cad1 | Azf1 | Gal4 | Msn2 | Msn4 | Rph1 | Sut1 | Skn7 | Phd1 | Ume6 | Stp1 | Put3 | Cellular Component |
|  |  |  |  |  |  |  |  |  |  |  |  |  |  |  |  | C:autophagic vacuole |
|  |  |  |  |  |  |  |  |  |  |  |  |  |  |  |  | C:vacuole |
|  |  |  |  |  |  |  |  |  |  |  |  |  |  |  |  | C:succinate dehydrogenase complex (ubiquinone) |
|  |  |  |  |  |  |  |  |  |  |  |  |  |  |  |  | C:fumarate reductase complex |
|  |  |  |  |  |  |  |  |  |  |  |  |  |  |  |  | C:succinate dehydrogenase complex |
|  |  |  |  |  |  |  |  |  |  |  |  |  |  |  |  | C:respiratory chain complex II (sensu Eukaryota) |
|  |  |  |  |  |  |  |  |  |  |  |  |  |  |  |  | C:respiratory chain complex II |
|  |  |  |  |  |  |  |  |  |  |  |  |  |  |  |  | C:external encapsulating structure |
|  |  |  |  |  |  |  |  |  |  |  |  |  |  |  |  | C:cell wall (sensu Fungi) |
|  |  |  |  |  |  |  |  |  |  |  |  |  |  |  |  | C:cell wall |
|  |  |  |  |  |  |  |  |  |  |  |  |  |  |  |  | C:cellular\_component |
|  |  |  |  |  |  |  |  |  |  |  |  |  |  |  |  | C:cytoplasm |
|
