# Supplementary material for: High-resolution analysis of condition-specific regulatory modules in Saccharomyces cerevisiae
Source: Genome Biol. 2008 Jan 3;9(1):R2. doi: 10.1186/gb-2008-9-1-r2 (PMC2395236; doi:10.1186/gb-2008-9-1-r2)
Supplement: Additional data file 11 — Matrices describing all EPMs and RMs, including lists of synergistic pairs of regulators. [file gb-2008-9-1-r2-S11.zip › htmls/C13_EPMs_matrix/EPM_17.Overlap.matrix.html]

|  |  |  |  |  |  |  |  |  |  |  |  |  |  |  |  |
| --- | --- | --- | --- | --- | --- | --- | --- | --- | --- | --- | --- | --- | --- | --- | --- |
| Hsf1 | Aft2 | Gln3 | Yap7 | Cad1 | Azf1 | Gal4 | Msn2 | Msn4 | Rph1 | Sut1 | Skn7 | Phd1 | Ume6 | Stp1 | Put3 |
|  |  |  |  |  |  |  |  |  |  |  |  |  |  |  |  | Hsf1 |
|  |  |  |  |  |  |  |  |  |  |  |  |  |  |  |  | Aft2 |
|  |  |  |  |  |  |  |  |  |  |  |  |  |  |  |  | Gln3 |
|  |  |  |  |  |  |  |  |  |  |  |  |  |  |  |  | Yap7 |
|  |  |  |  |  |  |  |  |  |  |  |  |  |  |  |  | Cad1 |
|  |  |  |  |  |  |  |  |  |  |  |  |  |  |  |  | Azf1 |
|  |  |  |  |  |  |  |  |  |  |  |  |  |  |  |  | Gal4 |
|  |  |  |  |  |  |  |  |  |  |  |  |  |  |  |  | Msn2 |
|  |  |  |  |  |  |  |  |  |  |  |  |  |  |  |  | Msn4 |
|  |  |  |  |  |  |  |  |  |  |  |  |  |  |  |  | Rph1 |
|  |  |  |  |  |  |  |  |  |  |  |  |  |  |  |  | Sut1 |
|  |  |  |  |  |  |  |  |  |  |  |  |  |  |  |  | Skn7 |
|  |  |  |  |  |  |  |  |  |  |  |  |  |  |  |  | Phd1 |
|  |  |  |  |  |  |  |  |  |  |  |  |  |  |  |  | Ume6 |
|  |  |  |  |  |  |  |  |  |  |  |  |  |  |  |  | Stp1 |
|  |  |  |  |  |  |  |  |  |  |  |  |  |  |  |  | Put3 |
 Hsf1 | Aft2 | Gln3 | Yap7 | Cad1 | Azf1 | Gal4 | Msn2 | Msn4 | Rph1 | Sut1 | Skn7 | Phd1 | Ume6 | Stp1 | Put3 |
