# Supplementary material for: High-resolution analysis of condition-specific regulatory modules in Saccharomyces cerevisiae
Source: Genome Biol. 2008 Jan 3;9(1):R2. doi: 10.1186/gb-2008-9-1-r2 (PMC2395236; doi:10.1186/gb-2008-9-1-r2)
Supplement: Additional data file 11 — Matrices describing all EPMs and RMs, including lists of synergistic pairs of regulators. [file gb-2008-9-1-r2-S11.zip › htmls/C13_EPMs_matrix/EPM_17.RM.matrix.html]

Regulators vs. RM target gene list

|  |  |  |  |  |  |  |  |  |  |  |  |  |  |  |  |  |
| --- | --- | --- | --- | --- | --- | --- | --- | --- | --- | --- | --- | --- | --- | --- | --- | --- |
|  | Hsf1 | Aft2 | Gln3 | Yap7 | Cad1 | Azf1 | Gal4 | Msn2 | Msn4 | Rph1 | Sut1 | Skn7 | Phd1 | Ume6 | Stp1 | Put3 |
| RM\_1 |  |  |  |  |  |  |  |  |  |  |  |  |  |  |  |  |
| RM\_2 |  |  |  |  |  |  |  |  |  |  |  |  |  |  |  |  |
| RM\_3 |  |  |  |  |  |  |  |  |  |  |  |  |  |  |  |  |
| RM\_4 |  |  |  |  |  |  |  |  |  |  |  |  |  |  |  |  |
| RM\_5 |  |  |  |  |  |  |  |  |  |  |  |  |  |  |  |  |
| RM\_6 |  |  |  |  |  |  |  |  |  |  |  |  |  |  |  |  |
| RM\_7 |  |  |  |  |  |  |  |  |  |  |  |  |  |  |  |  |

Synergistic Pair of Regulators

1. Azf1\*Phd1

2. Msn2\*Put3

3. Msn4\*Put3

4. Msn2\*Msn4

5. Msn2\*Stp1

6. Msn4\*Stp1

7. Msn2\*Ume6

8. Msn2\*Rph1

9. Msn4\*Rph1

10. Stp1\*Ume6

11. Gal4\*Sut1

Matrix of enriched GO

EPM matrix
