# Supplementary material for: High-resolution analysis of condition-specific regulatory modules in Saccharomyces cerevisiae
Source: Genome Biol. 2008 Jan 3;9(1):R2. doi: 10.1186/gb-2008-9-1-r2 (PMC2395236; doi:10.1186/gb-2008-9-1-r2)
Supplement: Additional data file 11 — Matrices describing all EPMs and RMs, including lists of synergistic pairs of regulators. [file gb-2008-9-1-r2-S11.zip › htmls/C13_EPMs_matrix/EPM_19.GO_enrichment.matrix.html]

|  |  |
| --- | --- |
| Hsf1 | Biological Process |
|  | P:ribosomal small subunit biogenesis |
|  | P:organelle organization and biogenesis |
|  | P:telomere organization and biogenesis |
|  | P:telomere maintenance |
|  | P:rNA metabolism |
|  | P:ribosome biogenesis and assembly |
|  | P:ribosome biogenesis |
|  | P:cytoplasm organization and biogenesis |
|
| Hsf1 | Molecular Function |
|  | F:tRNA (guanine) methyltransferase activity |
|  | F:glyceraldehyde-3-phosphate dehydrogenase activity |
|  | F:tRNA (guanine-N7-)-methyltransferase activity |
|  | F:glyceraldehyde-3-phosphate dehydrogenase (phosphorylating) activity |
|
| Hsf1 | Cellular Component |
|  | C:nucleus |
|  | C:organelle lumen |
|  | C:membrane-enclosed lumen |
|  | C:nucleolar part |
|  | C:nucleolus |
|  | C:nuclear lumen |
|
