# Supplementary material for: High-resolution analysis of condition-specific regulatory modules in Saccharomyces cerevisiae
Source: Genome Biol. 2008 Jan 3;9(1):R2. doi: 10.1186/gb-2008-9-1-r2 (PMC2395236; doi:10.1186/gb-2008-9-1-r2)
Supplement: Additional data file 11 — Matrices describing all EPMs and RMs, including lists of synergistic pairs of regulators. [file gb-2008-9-1-r2-S11.zip › htmls/C13_EPMs_matrix/EPM_2.GO_enrichment.matrix.html]

|  |  |  |  |  |  |  |  |  |  |  |  |  |
| --- | --- | --- | --- | --- | --- | --- | --- | --- | --- | --- | --- | --- |
| Ste12 | Mbp1 | Uga3 | Abf1 | Gcn4 | Bas1 | Leu3 | Pho4 | Cbf1 | Tye7 | Met32 | Met4 | Biological Process |
|  |  |  |  |  |  |  |  |  |  |  |  | P:cytogamy |
|  |  |  |  |  |  |  |  |  |  |  |  | P:carbohydrate metabolism |
|  |  |  |  |  |  |  |  |  |  |  |  | P:development |
|  |  |  |  |  |  |  |  |  |  |  |  | P:mRNA splice site selection |
|  |  |  |  |  |  |  |  |  |  |  |  | P:positive regulation of transcription from RNA polymerase II promoter by pheromones |
|  |  |  |  |  |  |  |  |  |  |  |  | P:positive regulation of transcription by pheromones |
|  |  |  |  |  |  |  |  |  |  |  |  | P:cellular carbohydrate metabolism |
|  |  |  |  |  |  |  |  |  |  |  |  | P:cellular polysaccharide metabolism |
|  |  |  |  |  |  |  |  |  |  |  |  | P:polysaccharide metabolism |
|  |  |  |  |  |  |  |  |  |  |  |  | P:cell organization and biogenesis |
|  |  |  |  |  |  |  |  |  |  |  |  | P:rNA import into nucleus |
|  |  |  |  |  |  |  |  |  |  |  |  | P:cell wall organization and biogenesis (sensu Fungi) |
|  |  |  |  |  |  |  |  |  |  |  |  | P:nuclear mRNA 5'-splice site recognition |
|  |  |  |  |  |  |  |  |  |  |  |  | P:polysaccharide biosynthesis |
|  |  |  |  |  |  |  |  |  |  |  |  | P:biopolymer biosynthesis |
|  |  |  |  |  |  |  |  |  |  |  |  | P:cell wall biosynthesis |
|  |  |  |  |  |  |  |  |  |  |  |  | P:cell wall biosynthesis (sensu Fungi) |
|  |  |  |  |  |  |  |  |  |  |  |  | P:response to stimulus |
|  |  |  |  |  |  |  |  |  |  |  |  | P:cellular morphogenesis during conjugation |
|  |  |  |  |  |  |  |  |  |  |  |  | P:cell adhesion |
|  |  |  |  |  |  |  |  |  |  |  |  | P:cellular morphogenesis during conjugation with cellular fusion |
|  |  |  |  |  |  |  |  |  |  |  |  | P:cell wall polysaccharide biosynthesis (sensu Fungi) |
|  |  |  |  |  |  |  |  |  |  |  |  | P:amino sugar metabolism |
|  |  |  |  |  |  |  |  |  |  |  |  | P:glucosamine metabolism |
|  |  |  |  |  |  |  |  |  |  |  |  | P:n-acetylglucosamine metabolism |
|  |  |  |  |  |  |  |  |  |  |  |  | P:amino sugar biosynthesis |
|  |  |  |  |  |  |  |  |  |  |  |  | P:glucosamine biosynthesis |
|  |  |  |  |  |  |  |  |  |  |  |  | P:n-acetylglucosamine biosynthesis |
|  |  |  |  |  |  |  |  |  |  |  |  | P:cell-cell adhesion |
|  |  |  |  |  |  |  |  |  |  |  |  | P:carbohydrate biosynthesis |
|  |  |  |  |  |  |  |  |  |  |  |  | P:chitin metabolism |
|  |  |  |  |  |  |  |  |  |  |  |  | P:heterophilic cell adhesion |
|  |  |  |  |  |  |  |  |  |  |  |  | P:chitin biosynthesis |
|  |  |  |  |  |  |  |  |  |  |  |  | P:cell wall chitin metabolism |
|  |  |  |  |  |  |  |  |  |  |  |  | P:cell wall chitin biosynthesis |
|  |  |  |  |  |  |  |  |  |  |  |  | P:response to chemical stimulus |
|  |  |  |  |  |  |  |  |  |  |  |  | P:agglutination during conjugation with cellular fusion |
|  |  |  |  |  |  |  |  |  |  |  |  | P:agglutination |
|  |  |  |  |  |  |  |  |  |  |  |  | P:reproduction |
|  |  |  |  |  |  |  |  |  |  |  |  | P:response to pheromone during conjugation with cellular fusion |
|  |  |  |  |  |  |  |  |  |  |  |  | P:interaction between organisms |
|  |  |  |  |  |  |  |  |  |  |  |  | P:reproductive cellular physiological process |
|  |  |  |  |  |  |  |  |  |  |  |  | P:reproductive physiological process |
|  |  |  |  |  |  |  |  |  |  |  |  | P:conjugation with cellular fusion |
|  |  |  |  |  |  |  |  |  |  |  |  | P:conjugation |
|  |  |  |  |  |  |  |  |  |  |  |  | P:response to pheromone |
|  |  |  |  |  |  |  |  |  |  |  |  | P:sexual reproduction |
|  |  |  |  |  |  |  |  |  |  |  |  | P:tubulin folding |
|  |  |  |  |  |  |  |  |  |  |  |  | P:beta-glucan metabolism |
|  |  |  |  |  |  |  |  |  |  |  |  | P:phosphatidylcholine metabolism |
|  |  |  |  |  |  |  |  |  |  |  |  | P:alcohol metabolism |
|  |  |  |  |  |  |  |  |  |  |  |  | P:beta-glucan biosynthesis |
|  |  |  |  |  |  |  |  |  |  |  |  | P:'de novo' protein folding |
|  |  |  |  |  |  |  |  |  |  |  |  | P:1,6-beta-glucan metabolism |
|  |  |  |  |  |  |  |  |  |  |  |  | P:cotranslational protein folding |
|  |  |  |  |  |  |  |  |  |  |  |  | P:1,6-beta-glucan biosynthesis |
|  |  |  |  |  |  |  |  |  |  |  |  | P:protein folding |
|  |  |  |  |  |  |  |  |  |  |  |  | P:phosphatidylinositol metabolism |
|  |  |  |  |  |  |  |  |  |  |  |  | P:phospholipid transport |
|  |  |  |  |  |  |  |  |  |  |  |  | P:lipid transport |
|  |  |  |  |  |  |  |  |  |  |  |  | P:asparaginyl-tRNA aminoacylation |
|  |  |  |  |  |  |  |  |  |  |  |  | P:pentose-phosphate shunt |
|  |  |  |  |  |  |  |  |  |  |  |  | P:adenine biosynthesis |
|  |  |  |  |  |  |  |  |  |  |  |  | P:catabolism |
|  |  |  |  |  |  |  |  |  |  |  |  | P:monosaccharide metabolism |
|  |  |  |  |  |  |  |  |  |  |  |  | P:cofactor metabolism |
|  |  |  |  |  |  |  |  |  |  |  |  | P:cellular catabolism |
|  |  |  |  |  |  |  |  |  |  |  |  | P:hexose metabolism |
|  |  |  |  |  |  |  |  |  |  |  |  | P:water-soluble vitamin metabolism |
|  |  |  |  |  |  |  |  |  |  |  |  | P:vitamin metabolism |
|  |  |  |  |  |  |  |  |  |  |  |  | P:main pathways of carbohydrate metabolism |
|  |  |  |  |  |  |  |  |  |  |  |  | P:coenzyme metabolism |
|  |  |  |  |  |  |  |  |  |  |  |  | P:glucose metabolism |
|  |  |  |  |  |  |  |  |  |  |  |  | P:nucleobase, nucleoside, nucleotide and nucleic acid metabolism |
|  |  |  |  |  |  |  |  |  |  |  |  | P:carbohydrate catabolism |
|  |  |  |  |  |  |  |  |  |  |  |  | P:cellular carbohydrate catabolism |
|  |  |  |  |  |  |  |  |  |  |  |  | P:pentose-phosphate shunt, oxidative branch |
|  |  |  |  |  |  |  |  |  |  |  |  | P:oxidoreduction coenzyme metabolism |
|  |  |  |  |  |  |  |  |  |  |  |  | P:alcohol catabolism |
|  |  |  |  |  |  |  |  |  |  |  |  | P:pyridine nucleotide metabolism |
|  |  |  |  |  |  |  |  |  |  |  |  | P:monosaccharide catabolism |
|  |  |  |  |  |  |  |  |  |  |  |  | P:nicotinamide metabolism |
|  |  |  |  |  |  |  |  |  |  |  |  | P:hexose catabolism |
|  |  |  |  |  |  |  |  |  |  |  |  | P:glucose catabolism |
|  |  |  |  |  |  |  |  |  |  |  |  | P:nADPH regeneration |
|  |  |  |  |  |  |  |  |  |  |  |  | P:nADP metabolism |
|  |  |  |  |  |  |  |  |  |  |  |  | P:asparagine metabolism |
|  |  |  |  |  |  |  |  |  |  |  |  | P:threonine metabolism |
|  |  |  |  |  |  |  |  |  |  |  |  | P:organic cation transport |
|  |  |  |  |  |  |  |  |  |  |  |  | P:ammonium transport |
|  |  |  |  |  |  |  |  |  |  |  |  | P:tricarboxylic acid cycle intermediate metabolism |
|  |  |  |  |  |  |  |  |  |  |  |  | P:asparagine biosynthesis |
|  |  |  |  |  |  |  |  |  |  |  |  | P:glutamate metabolism |
|  |  |  |  |  |  |  |  |  |  |  |  | P:homoserine biosynthesis |
|  |  |  |  |  |  |  |  |  |  |  |  | P:biological\_process |
|  |  |  |  |  |  |  |  |  |  |  |  | P:sulfate transport |
|  |  |  |  |  |  |  |  |  |  |  |  | P:leucine metabolism |
|  |  |  |  |  |  |  |  |  |  |  |  | P:leucine biosynthesis |
|  |  |  |  |  |  |  |  |  |  |  |  | P:branched chain family amino acid biosynthesis |
|  |  |  |  |  |  |  |  |  |  |  |  | P:branched chain family amino acid metabolism |
|  |  |  |  |  |  |  |  |  |  |  |  | P:nucleotide metabolism |
|  |  |  |  |  |  |  |  |  |  |  |  | P:amino acid activation |
|  |  |  |  |  |  |  |  |  |  |  |  | P:tRNA aminoacylation |
|  |  |  |  |  |  |  |  |  |  |  |  | P:tRNA aminoacylation for protein translation |
|  |  |  |  |  |  |  |  |  |  |  |  | P:secondary metabolism |
|  |  |  |  |  |  |  |  |  |  |  |  | P:nucleobase biosynthesis |
|  |  |  |  |  |  |  |  |  |  |  |  | P:pigment metabolism |
|  |  |  |  |  |  |  |  |  |  |  |  | P:pigment biosynthesis |
|  |  |  |  |  |  |  |  |  |  |  |  | P:amine catabolism |
|  |  |  |  |  |  |  |  |  |  |  |  | P:nitrogen compound catabolism |
|  |  |  |  |  |  |  |  |  |  |  |  | P:amino acid catabolism |
|  |  |  |  |  |  |  |  |  |  |  |  | P:one-carbon compound metabolism |
|  |  |  |  |  |  |  |  |  |  |  |  | P:purine base biosynthesis |
|  |  |  |  |  |  |  |  |  |  |  |  | P:glycine metabolism |
|  |  |  |  |  |  |  |  |  |  |  |  | P:serine family amino acid catabolism |
|  |  |  |  |  |  |  |  |  |  |  |  | P:glycine catabolism |
|  |  |  |  |  |  |  |  |  |  |  |  | P:heterocycle metabolism |
|  |  |  |  |  |  |  |  |  |  |  |  | P:nucleobase metabolism |
|  |  |  |  |  |  |  |  |  |  |  |  | P:ribonucleotide metabolism |
|  |  |  |  |  |  |  |  |  |  |  |  | P:ribonucleotide biosynthesis |
|  |  |  |  |  |  |  |  |  |  |  |  | P:purine ribonucleotide metabolism |
|  |  |  |  |  |  |  |  |  |  |  |  | P:purine ribonucleotide biosynthesis |
|  |  |  |  |  |  |  |  |  |  |  |  | P:purine ribonucleoside monophosphate metabolism |
|  |  |  |  |  |  |  |  |  |  |  |  | P:ribonucleoside monophosphate metabolism |
|  |  |  |  |  |  |  |  |  |  |  |  | P:aromatic compound metabolism |
|  |  |  |  |  |  |  |  |  |  |  |  | P:nucleoside monophosphate metabolism |
|  |  |  |  |  |  |  |  |  |  |  |  | P:purine base metabolism |
|  |  |  |  |  |  |  |  |  |  |  |  | P:purine nucleoside monophosphate metabolism |
|  |  |  |  |  |  |  |  |  |  |  |  | P:purine ribonucleoside monophosphate biosynthesis |
|  |  |  |  |  |  |  |  |  |  |  |  | P:ribonucleoside monophosphate biosynthesis |
|  |  |  |  |  |  |  |  |  |  |  |  | P:nucleoside monophosphate biosynthesis |
|  |  |  |  |  |  |  |  |  |  |  |  | P:iMP metabolism |
|  |  |  |  |  |  |  |  |  |  |  |  | P:iMP biosynthesis |
|  |  |  |  |  |  |  |  |  |  |  |  | P:'de novo' IMP biosynthesis |
|  |  |  |  |  |  |  |  |  |  |  |  | P:purine nucleoside monophosphate biosynthesis |
|  |  |  |  |  |  |  |  |  |  |  |  | P:iron-sulfur cluster assembly |
|  |  |  |  |  |  |  |  |  |  |  |  | P:metallo-sulfur cluster assembly |
|  |  |  |  |  |  |  |  |  |  |  |  | P:glutamate biosynthesis |
|  |  |  |  |  |  |  |  |  |  |  |  | P:urea cycle intermediate metabolism |
|  |  |  |  |  |  |  |  |  |  |  |  | P:arginine metabolism |
|  |  |  |  |  |  |  |  |  |  |  |  | P:arginine biosynthesis |
|  |  |  |  |  |  |  |  |  |  |  |  | P:phenylalanyl-tRNA aminoacylation |
|  |  |  |  |  |  |  |  |  |  |  |  | P:aromatic amino acid family metabolism |
|  |  |  |  |  |  |  |  |  |  |  |  | P:aromatic compound biosynthesis |
|  |  |  |  |  |  |  |  |  |  |  |  | P:aromatic amino acid family biosynthesis |
|  |  |  |  |  |  |  |  |  |  |  |  | P:lysine biosynthesis |
|  |  |  |  |  |  |  |  |  |  |  |  | P:lysine metabolism |
|  |  |  |  |  |  |  |  |  |  |  |  | P:lysine biosynthesis via aminoadipic acid |
|  |  |  |  |  |  |  |  |  |  |  |  | P:glutamine family amino acid biosynthesis |
|  |  |  |  |  |  |  |  |  |  |  |  | P:glutamine family amino acid metabolism |
|  |  |  |  |  |  |  |  |  |  |  |  | P:cysteine biosynthesis |
|  |  |  |  |  |  |  |  |  |  |  |  | P:nucleoside diphosphate biosynthesis |
|  |  |  |  |  |  |  |  |  |  |  |  | P:purine nucleotide metabolism |
|  |  |  |  |  |  |  |  |  |  |  |  | P:nucleotide biosynthesis |
|  |  |  |  |  |  |  |  |  |  |  |  | P:amino acid biosynthesis |
|  |  |  |  |  |  |  |  |  |  |  |  | P:amine biosynthesis |
|  |  |  |  |  |  |  |  |  |  |  |  | P:nitrogen compound biosynthesis |
|  |  |  |  |  |  |  |  |  |  |  |  | P:cysteine metabolism |
|  |  |  |  |  |  |  |  |  |  |  |  | P:serine family amino acid biosynthesis |
|  |  |  |  |  |  |  |  |  |  |  |  | P:homoserine metabolism |
|  |  |  |  |  |  |  |  |  |  |  |  | P:methionine biosynthesis |
|  |  |  |  |  |  |  |  |  |  |  |  | P:histidine biosynthesis |
|  |  |  |  |  |  |  |  |  |  |  |  | P:histidine family amino acid metabolism |
|  |  |  |  |  |  |  |  |  |  |  |  | P:histidine family amino acid biosynthesis |
|  |  |  |  |  |  |  |  |  |  |  |  | P:histidine metabolism |
|  |  |  |  |  |  |  |  |  |  |  |  | P:sulfur utilization |
|  |  |  |  |  |  |  |  |  |  |  |  | P:sulfate assimilation |
|  |  |  |  |  |  |  |  |  |  |  |  | P:sulfur compound biosynthesis |
|  |  |  |  |  |  |  |  |  |  |  |  | P:sulfur amino acid biosynthesis |
|  |  |  |  |  |  |  |  |  |  |  |  | P:aspartate family amino acid biosynthesis |
|  |  |  |  |  |  |  |  |  |  |  |  | P:aspartate family amino acid metabolism |
|  |  |  |  |  |  |  |  |  |  |  |  | P:methionine metabolism |
|  |  |  |  |  |  |  |  |  |  |  |  | P:physiological process |
|  |  |  |  |  |  |  |  |  |  |  |  | P:cellular process |
|  |  |  |  |  |  |  |  |  |  |  |  | P:cellular physiological process |
|  |  |  |  |  |  |  |  |  |  |  |  | P:amino acid metabolism |
|  |  |  |  |  |  |  |  |  |  |  |  | P:amine metabolism |
|  |  |  |  |  |  |  |  |  |  |  |  | P:organic acid metabolism |
|  |  |  |  |  |  |  |  |  |  |  |  | P:carboxylic acid metabolism |
|  |  |  |  |  |  |  |  |  |  |  |  | P:cellular biosynthesis |
|  |  |  |  |  |  |  |  |  |  |  |  | P:amino acid and derivative metabolism |
|  |  |  |  |  |  |  |  |  |  |  |  | P:nitrogen compound metabolism |
|  |  |  |  |  |  |  |  |  |  |  |  | P:biosynthesis |
|  |  |  |  |  |  |  |  |  |  |  |  | P:cellular metabolism |
|  |  |  |  |  |  |  |  |  |  |  |  | P:metabolism |
|  |  |  |  |  |  |  |  |  |  |  |  | P:primary metabolism |
|  |  |  |  |  |  |  |  |  |  |  |  | P:serine family amino acid metabolism |
|  |  |  |  |  |  |  |  |  |  |  |  | P:purine nucleotide biosynthesis |
|  |  |  |  |  |  |  |  |  |  |  |  | P:sulfur metabolism |
|  |  |  |  |  |  |  |  |  |  |  |  | P:sulfur amino acid metabolism |
|  |  |  |  |  |  |  |  |  |  |  |  | P:hyperosmotic salinity response |
|  |  |  |  |  |  |  |  |  |  |  |  | P:adenosine metabolism |
|  |  |  |  |  |  |  |  |  |  |  |  | P:adenosine biosynthesis |
|  |  |  |  |  |  |  |  |  |  |  |  | P:transsulfuration |
|  |  |  |  |  |  |  |  |  |  |  |  | P:nucleoside biosynthesis |
|  |  |  |  |  |  |  |  |  |  |  |  | P:purine ribonucleoside biosynthesis |
|  |  |  |  |  |  |  |  |  |  |  |  | P:ribonucleoside biosynthesis |
|  |  |  |  |  |  |  |  |  |  |  |  | P:purine nucleoside biosynthesis |
|  |  |  |  |  |  |  |  |  |  |  |  | P:purine nucleoside diphosphate metabolism |
|  |  |  |  |  |  |  |  |  |  |  |  | P:purine nucleoside diphosphate biosynthesis |
|  |  |  |  |  |  |  |  |  |  |  |  | P:purine ribonucleoside diphosphate metabolism |
|  |  |  |  |  |  |  |  |  |  |  |  | P:aDP metabolism |
|  |  |  |  |  |  |  |  |  |  |  |  | P:ribonucleoside diphosphate metabolism |
|  |  |  |  |  |  |  |  |  |  |  |  | P:purine ribonucleoside diphosphate biosynthesis |
|  |  |  |  |  |  |  |  |  |  |  |  | P:aDP biosynthesis |
|  |  |  |  |  |  |  |  |  |  |  |  | P:ribonucleoside diphosphate biosynthesis |
|  |  |  |  |  |  |  |  |  |  |  |  | P:acetate biosynthesis |
|  |  |  |  |  |  |  |  |  |  |  |  | P:dicarboxylic acid transport |
|  |  |  |  |  |  |  |  |  |  |  |  | P:branched chain family amino acid catabolism |
|  |  |  |  |  |  |  |  |  |  |  |  | P:oxaloacetate transport |
|  |  |  |  |  |  |  |  |  |  |  |  | P:inorganic anion transport |
|  |  |  |  |  |  |  |  |  |  |  |  | P:anion transport |
|
| Ste12 | Mbp1 | Uga3 | Abf1 | Gcn4 | Bas1 | Leu3 | Pho4 | Cbf1 | Tye7 | Met32 | Met4 | Molecular Function |
|  |  |  |  |  |  |  |  |  |  |  |  | F:ligase activity, forming carbon-carbon bonds |
|  |  |  |  |  |  |  |  |  |  |  |  | F:enzyme regulator activity |
|  |  |  |  |  |  |  |  |  |  |  |  | F:chitin synthase activity |
|  |  |  |  |  |  |  |  |  |  |  |  | F:pyruvate carboxylase activity |
|  |  |  |  |  |  |  |  |  |  |  |  | F:ran GTPase activator activity |
|  |  |  |  |  |  |  |  |  |  |  |  | F:pre-mRNA 5'-splice site binding |
|  |  |  |  |  |  |  |  |  |  |  |  | F:phosphoribosylaminoimidazolesuccinocarboxamide synthase activity |
|  |  |  |  |  |  |  |  |  |  |  |  | F:cell adhesion molecule binding |
|  |  |  |  |  |  |  |  |  |  |  |  | F:enzyme activator activity |
|  |  |  |  |  |  |  |  |  |  |  |  | F:transaldolase activity |
|  |  |  |  |  |  |  |  |  |  |  |  | F:phospholipid transporter activity |
|  |  |  |  |  |  |  |  |  |  |  |  | F:lipid transporter activity |
|  |  |  |  |  |  |  |  |  |  |  |  | F:phosphatidylinositol transporter activity |
|  |  |  |  |  |  |  |  |  |  |  |  | F:transferase activity, transferring aldehyde or ketonic groups |
|  |  |  |  |  |  |  |  |  |  |  |  | F:anion transporter activity |
|  |  |  |  |  |  |  |  |  |  |  |  | F:adenyl nucleotide binding |
|  |  |  |  |  |  |  |  |  |  |  |  | F:ketol-acid reductoisomerase activity |
|  |  |  |  |  |  |  |  |  |  |  |  | F:3-isopropylmalate dehydratase activity |
|  |  |  |  |  |  |  |  |  |  |  |  | F:fAD binding |
|  |  |  |  |  |  |  |  |  |  |  |  | F:oxaloacetate carrier activity |
|  |  |  |  |  |  |  |  |  |  |  |  | F:oxaloacetate transporter activity |
|  |  |  |  |  |  |  |  |  |  |  |  | F:sulfate porter activity |
|  |  |  |  |  |  |  |  |  |  |  |  | F:dihydroxy-acid dehydratase activity |
|  |  |  |  |  |  |  |  |  |  |  |  | F:3-chloroallyl aldehyde dehydrogenase activity |
|  |  |  |  |  |  |  |  |  |  |  |  | F:cofactor binding |
|  |  |  |  |  |  |  |  |  |  |  |  | F:dicarboxylic acid transporter activity |
|  |  |  |  |  |  |  |  |  |  |  |  | F:acetolactate synthase activity |
|  |  |  |  |  |  |  |  |  |  |  |  | F:coenzyme binding |
|  |  |  |  |  |  |  |  |  |  |  |  | F:branched-chain-amino-acid transaminase activity |
|  |  |  |  |  |  |  |  |  |  |  |  | F:adenylate kinase activity |
|  |  |  |  |  |  |  |  |  |  |  |  | F:methionine adenosyltransferase activity |
|  |  |  |  |  |  |  |  |  |  |  |  | F:methenyltetrahydrofolate cyclohydrolase activity |
|  |  |  |  |  |  |  |  |  |  |  |  | F:cystathionine gamma-lyase activity |
|  |  |  |  |  |  |  |  |  |  |  |  | F:cysteine synthase activity |
|  |  |  |  |  |  |  |  |  |  |  |  | F:formate-tetrahydrofolate ligase activity |
|  |  |  |  |  |  |  |  |  |  |  |  | F:methylenetetrahydrofolate dehydrogenase (NADP+) activity |
|  |  |  |  |  |  |  |  |  |  |  |  | F:phosphoglycerate dehydrogenase activity |
|  |  |  |  |  |  |  |  |  |  |  |  | F:methylenetetrahydrofolate reductase (NADPH) activity |
|  |  |  |  |  |  |  |  |  |  |  |  | F:2-isopropylmalate synthase activity |
|  |  |  |  |  |  |  |  |  |  |  |  | F:transferase activity, transferring pentosyl groups |
|  |  |  |  |  |  |  |  |  |  |  |  | F:transferase activity, transferring alkyl or aryl (other than methyl) groups |
|  |  |  |  |  |  |  |  |  |  |  |  | F:oxidoreductase activity, acting on sulfur group of donors |
|  |  |  |  |  |  |  |  |  |  |  |  | F:oxidoreductase activity, acting on sulfur group of donors, NAD or NADP as acceptor |
|  |  |  |  |  |  |  |  |  |  |  |  | F:transferase activity |
|  |  |  |  |  |  |  |  |  |  |  |  | F:sulfite reductase (NADPH) activity |
|  |  |  |  |  |  |  |  |  |  |  |  | F:catalytic activity |
|  |  |  |  |  |  |  |  |  |  |  |  | F:oxidoreductase activity, acting on the CH-NH group of donors |
|  |  |  |  |  |  |  |  |  |  |  |  | F:oxidoreductase activity |
|  |  |  |  |  |  |  |  |  |  |  |  | F:oxidoreductase activity, acting on the CH-NH group of donors, NAD or NADP as acceptor |
|  |  |  |  |  |  |  |  |  |  |  |  | F:aTP phosphoribosyltransferase activity |
|  |  |  |  |  |  |  |  |  |  |  |  | F:sulfate adenylyltransferase (ATP) activity |
|  |  |  |  |  |  |  |  |  |  |  |  | F:homoserine O-acetyltransferase activity |
|  |  |  |  |  |  |  |  |  |  |  |  | F:o-acetylhomoserine aminocarboxypropyltransferase activity |
|  |  |  |  |  |  |  |  |  |  |  |  | F:3'(2'),5'-bisphosphate nucleotidase activity |
|  |  |  |  |  |  |  |  |  |  |  |  | F:imidazoleglycerol-phosphate synthase activity |
|  |  |  |  |  |  |  |  |  |  |  |  | F:electron carrier activity |
|  |  |  |  |  |  |  |  |  |  |  |  | F:sulfate adenylyltransferase activity |
|  |  |  |  |  |  |  |  |  |  |  |  | F:5-methyltetrahydropteroyltri-L-glutamate-dependent methyltransferase activity |
|  |  |  |  |  |  |  |  |  |  |  |  | F:adenylosuccinate synthase activity |
|  |  |  |  |  |  |  |  |  |  |  |  | F:5-methyltetrahydropteroyltriglutamate-homocysteine S-methyltransferase activity |
|  |  |  |  |  |  |  |  |  |  |  |  | F:ligase activity, forming aminoacyl-tRNA and related compounds |
|  |  |  |  |  |  |  |  |  |  |  |  | F:aminoacyl-tRNA ligase activity |
|  |  |  |  |  |  |  |  |  |  |  |  | F:ligase activity, forming carbon-oxygen bonds |
|  |  |  |  |  |  |  |  |  |  |  |  | F:phosphoribosylformylglycinamidine cyclo-ligase activity |
|  |  |  |  |  |  |  |  |  |  |  |  | F:phosphoribosylformylglycinamidine synthase activity |
|  |  |  |  |  |  |  |  |  |  |  |  | F:phosphoribosylglycinamide formyltransferase activity |
|  |  |  |  |  |  |  |  |  |  |  |  | F:dolichol kinase activity |
|  |  |  |  |  |  |  |  |  |  |  |  | F:adenylosuccinate lyase activity |
|  |  |  |  |  |  |  |  |  |  |  |  | F:methylenetetrahydrofolate dehydrogenase (NAD+) activity |
|  |  |  |  |  |  |  |  |  |  |  |  | F:histidinol dehydrogenase activity |
|  |  |  |  |  |  |  |  |  |  |  |  | F:aspartic-type signal peptidase activity |
|  |  |  |  |  |  |  |  |  |  |  |  | F:phosphoribosylaminoimidazole carboxylase activity |
|  |  |  |  |  |  |  |  |  |  |  |  | F:phosphoribosyl-AMP cyclohydrolase activity |
|  |  |  |  |  |  |  |  |  |  |  |  | F:phosphoribosylamine-glycine ligase activity |
|  |  |  |  |  |  |  |  |  |  |  |  | F:methyltransferase activity |
|  |  |  |  |  |  |  |  |  |  |  |  | F:amidophosphoribosyltransferase activity |
|  |  |  |  |  |  |  |  |  |  |  |  | F:oxidoreductase activity, acting on the aldehyde or oxo group of donors |
|  |  |  |  |  |  |  |  |  |  |  |  | F:ammonium transporter activity |
|  |  |  |  |  |  |  |  |  |  |  |  | F:organic cation transporter activity |
|  |  |  |  |  |  |  |  |  |  |  |  | F:oxidoreductase activity, acting on the aldehyde or oxo group of donors, NAD or NADP as acceptor |
|  |  |  |  |  |  |  |  |  |  |  |  | F:transaminase activity |
|  |  |  |  |  |  |  |  |  |  |  |  | F:transferase activity, transferring nitrogenous groups |
|  |  |  |  |  |  |  |  |  |  |  |  | F:transferase activity, transferring acyl groups |
|  |  |  |  |  |  |  |  |  |  |  |  | F:ligase activity, forming carbon-nitrogen bonds |
|  |  |  |  |  |  |  |  |  |  |  |  | F:transferase activity, transferring one-carbon groups |
|  |  |  |  |  |  |  |  |  |  |  |  | F:hydroxymethyl-, formyl- and related transferase activity |
|  |  |  |  |  |  |  |  |  |  |  |  | F:hydrolase activity, acting on carbon-nitrogen (but not peptide) bonds, in cyclic amidines |
|  |  |  |  |  |  |  |  |  |  |  |  | F:methylenetetrahydrofolate dehydrogenase activity |
|  |  |  |  |  |  |  |  |  |  |  |  | F:cyclohydrolase activity |
|  |  |  |  |  |  |  |  |  |  |  |  | F:glycine hydroxymethyltransferase activity |
|  |  |  |  |  |  |  |  |  |  |  |  | F:oxidoreductase activity, acting on the CH-NH2 group of donors |
|  |  |  |  |  |  |  |  |  |  |  |  | F:glycine dehydrogenase (decarboxylating) activity |
|  |  |  |  |  |  |  |  |  |  |  |  | F:ligase activity |
|  |  |  |  |  |  |  |  |  |  |  |  | F:oxidoreductase activity, acting on the CH-NH2 group of donors, disulfide as acceptor |
|  |  |  |  |  |  |  |  |  |  |  |  | F:carbon-nitrogen lyase activity |
|  |  |  |  |  |  |  |  |  |  |  |  | F:carbamoyl-phosphate synthase activity |
|  |  |  |  |  |  |  |  |  |  |  |  | F:carbamoyl-phosphate synthase (glutamine-hydrolyzing) activity |
|  |  |  |  |  |  |  |  |  |  |  |  | F:phenylalanine-tRNA ligase activity |
|  |  |  |  |  |  |  |  |  |  |  |  | F:3-deoxy-7-phosphoheptulonate synthase activity |
|  |  |  |  |  |  |  |  |  |  |  |  | F:carbon-nitrogen ligase activity, with glutamine as amido-N-donor |
|  |  |  |  |  |  |  |  |  |  |  |  | F:aconitate hydratase activity |
|  |  |  |  |  |  |  |  |  |  |  |  | F:inorganic anion transporter activity |
|  |  |  |  |  |  |  |  |  |  |  |  | F:oxidoreductase activity, acting on the CH-OH group of donors, NAD or NADP as acceptor |
|  |  |  |  |  |  |  |  |  |  |  |  | F:oxidoreductase activity, acting on CH-OH group of donors |
|  |  |  |  |  |  |  |  |  |  |  |  | F:molecular\_function |
|  |  |  |  |  |  |  |  |  |  |  |  | F:hydro-lyase activity |
|  |  |  |  |  |  |  |  |  |  |  |  | F:transferase activity, transferring acyl groups, acyl groups converted into alkyl on transfer |
|  |  |  |  |  |  |  |  |  |  |  |  | F:sulfate transporter activity |
|  |  |  |  |  |  |  |  |  |  |  |  | F:lyase activity |
|  |  |  |  |  |  |  |  |  |  |  |  | F:carbon-oxygen lyase activity |
|
| Ste12 | Mbp1 | Uga3 | Abf1 | Gcn4 | Bas1 | Leu3 | Pho4 | Cbf1 | Tye7 | Met32 | Met4 | Cellular Component |
|  |  |  |  |  |  |  |  |  |  |  |  | C:golgi cis cisterna |
|  |  |  |  |  |  |  |  |  |  |  |  | C:nascent polypeptide-associated complex |
|  |  |  |  |  |  |  |  |  |  |  |  | C:prefoldin complex |
|  |  |  |  |  |  |  |  |  |  |  |  | C:chitosome |
|  |  |  |  |  |  |  |  |  |  |  |  | C:site of polarized growth |
|  |  |  |  |  |  |  |  |  |  |  |  | C:incipient bud site |
|  |  |  |  |  |  |  |  |  |  |  |  | C:external encapsulating structure |
|  |  |  |  |  |  |  |  |  |  |  |  | C:cell wall (sensu Fungi) |
|  |  |  |  |  |  |  |  |  |  |  |  | C:cell wall |
|  |  |  |  |  |  |  |  |  |  |  |  | C:mitochondrial part |
|  |  |  |  |  |  |  |  |  |  |  |  | C:acetolactate synthase complex |
|  |  |  |  |  |  |  |  |  |  |  |  | C:mitochondrial lumen |
|  |  |  |  |  |  |  |  |  |  |  |  | C:cytoplasmic part |
|  |  |  |  |  |  |  |  |  |  |  |  | C:mitochondrial matrix |
|  |  |  |  |  |  |  |  |  |  |  |  | C:heterogeneous nuclear ribonucleoprotein complex |
|  |  |  |  |  |  |  |  |  |  |  |  | C:cytoplasm |
|  |  |  |  |  |  |  |  |  |  |  |  | C:intracellular |
|  |  |  |  |  |  |  |  |  |  |  |  | C:sulfite reductase complex (NADPH) |
|  |  |  |  |  |  |  |  |  |  |  |  | C:cell part |
|  |  |  |  |  |  |  |  |  |  |  |  | C:cell |
|  |  |  |  |  |  |  |  |  |  |  |  | C:glycine cleavage complex |
|  |  |  |  |  |  |  |  |  |  |  |  | C:intracellular part |
|  |  |  |  |  |  |  |  |  |  |  |  | C:unlocalized protein complex |
|  |  |  |  |  |  |  |  |  |  |  |  | C:mitochondrial nucleoid |
|  |  |  |  |  |  |  |  |  |  |  |  | C:nucleoid |
|  |  |  |  |  |  |  |  |  |  |  |  | C:phenylalanine-tRNA ligase complex |
|  |  |  |  |  |  |  |  |  |  |  |  | C:carbamoyl-phosphate synthase complex |
|  |  |  |  |  |  |  |  |  |  |  |  | C:mitochondrion |
|  |  |  |  |  |  |  |  |  |  |  |  | C:cellular\_component |
|
