# Supplementary material for: High-resolution analysis of condition-specific regulatory modules in Saccharomyces cerevisiae
Source: Genome Biol. 2008 Jan 3;9(1):R2. doi: 10.1186/gb-2008-9-1-r2 (PMC2395236; doi:10.1186/gb-2008-9-1-r2)
Supplement: Additional data file 11 — Matrices describing all EPMs and RMs, including lists of synergistic pairs of regulators. [file gb-2008-9-1-r2-S11.zip › htmls/C13_EPMs_matrix/EPM_20.GO_enrichment.matrix.html]

|  |  |  |
| --- | --- | --- |
| Hsf1 | Cin5 | Biological Process |
|  |  | P:response to stress |
|  |  | P:protein folding |
|  |  | P:response to inorganic substance |
|  |  | P:response to copper ion |
|  |  | P:response to metal ion |
|
| Hsf1 | Cin5 | Molecular Function |
|  |  | F:enzyme activator activity |
|  |  | F:adenyl-nucleotide exchange factor activity |
|  |  | F:chaperone activator activity |
|  |  | F:hsp70/Hsc70 protein regulator activity |
|  |  | F:metal ion binding |
|  |  | F:ion binding |
|  |  | F:cation binding |
|  |  | F:transition metal ion binding |
|  |  | F:chaperone regulator activity |
|  |  | F:copper ion binding |
|  |  | F:aTPase stimulator activity |
|  |  | F:kinase activator activity |
|  |  | F:tetrahydrofolylpolyglutamate synthase activity |
|  |  | F:protein kinase activator activity |
|
| Hsf1 | Cin5 | Cellular Component |
|  |  | C:plasma membrane |
|  |  | C:cytosol |
|
