# Supplementary material for: High-resolution analysis of condition-specific regulatory modules in Saccharomyces cerevisiae
Source: Genome Biol. 2008 Jan 3;9(1):R2. doi: 10.1186/gb-2008-9-1-r2 (PMC2395236; doi:10.1186/gb-2008-9-1-r2)
Supplement: Additional data file 11 — Matrices describing all EPMs and RMs, including lists of synergistic pairs of regulators. [file gb-2008-9-1-r2-S11.zip › htmls/C13_EPMs_matrix/EPM_21.GO_enrichment.matrix.html]

|  |  |  |  |
| --- | --- | --- | --- |
| Skn7 | Msn2 | Msn4 | Biological Process |
|  |  |  | P:age-dependent general metabolic decline |
|  |  |  | P:age-dependent response to oxidative stress |
|  |  |  | P:response to reactive oxygen species |
|  |  |  | P:age-dependent general metabolic decline during chronological cell aging |
|  |  |  | P:age-dependent response to oxidative stress during chronological cell aging |
|  |  |  | P:trehalose metabolism |
|  |  |  | P:response to stress |
|  |  |  | P:trehalose biosynthesis |
|  |  |  | P:disaccharide biosynthesis |
|  |  |  | P:age-dependent response to reactive oxygen species |
|  |  |  | P:mitochondrial iron ion transport |
|  |  |  | P:age-dependent response to reactive oxygen species during chronological cell aging |
|  |  |  | P:carbohydrate biosynthesis |
|  |  |  | P:oxygen and reactive oxygen species metabolism |
|  |  |  | P:energy reserve metabolism |
|  |  |  | P:response to oxidative stress |
|
| Skn7 | Msn2 | Msn4 | Molecular Function |
|  |  |  | F:thioredoxin peroxidase activity |
|  |  |  | F:carbohydrate phosphatase activity |
|  |  |  | F:transferase activity, transferring glycosyl groups |
|  |  |  | F:alpha,alpha-trehalose-phosphate synthase (UDP-forming) activity |
|  |  |  | F:trehalose-phosphatase activity |
|  |  |  | F:transferase activity, transferring hexosyl groups |
|  |  |  | F:superoxide dismutase activity |
|  |  |  | F:oxidoreductase activity, acting on superoxide radicals as acceptor |
|  |  |  | F:manganese superoxide dismutase activity |
|  |  |  | F:1,4-alpha-glucan branching enzyme activity |
|
| Skn7 | Msn2 | Msn4 | Cellular Component |
|  |  |  | C:cytoplasm |
|  |  |  | C:intrinsic to peroxisomal membrane |
|  |  |  | C:alpha,alpha-trehalose-phosphate synthase complex (UDP-forming) |
|  |  |  | C:integral to peroxisomal membrane |
|
