# Supplementary material for: High-resolution analysis of condition-specific regulatory modules in Saccharomyces cerevisiae
Source: Genome Biol. 2008 Jan 3;9(1):R2. doi: 10.1186/gb-2008-9-1-r2 (PMC2395236; doi:10.1186/gb-2008-9-1-r2)
Supplement: Additional data file 11 — Matrices describing all EPMs and RMs, including lists of synergistic pairs of regulators. [file gb-2008-9-1-r2-S11.zip › htmls/C13_EPMs_matrix/EPM_22.GO_enrichment.matrix.html]

|  |  |  |  |  |  |  |  |  |  |  |  |  |  |  |  |
| --- | --- | --- | --- | --- | --- | --- | --- | --- | --- | --- | --- | --- | --- | --- | --- |
| Cin5 | Yap7 | Rlm1 | Msn2 | Msn4 | Ume6 | Sut1 | Stp1 | Pho2 | Aft2 | Put3 | Hsf1 | Gln3 | Gat1 | Dal82 | Biological Process |
|  |  |  |  |  |  |  |  |  |  |  |  |  |  |  | P:nAD metabolism |
|  |  |  |  |  |  |  |  |  |  |  |  |  |  |  | P:pentose-phosphate shunt, oxidative branch |
|  |  |  |  |  |  |  |  |  |  |  |  |  |  |  | P:mitochondrial fusion |
|  |  |  |  |  |  |  |  |  |  |  |  |  |  |  | P:heme a metabolism |
|  |  |  |  |  |  |  |  |  |  |  |  |  |  |  | P:heme a biosynthesis |
|  |  |  |  |  |  |  |  |  |  |  |  |  |  |  | P:ketone metabolism |
|  |  |  |  |  |  |  |  |  |  |  |  |  |  |  | P:steroid metabolism |
|  |  |  |  |  |  |  |  |  |  |  |  |  |  |  | P:2-oxoglutarate metabolism |
|  |  |  |  |  |  |  |  |  |  |  |  |  |  |  | P:mitochondrial genome maintenance |
|  |  |  |  |  |  |  |  |  |  |  |  |  |  |  | P:threonine catabolism |
|  |  |  |  |  |  |  |  |  |  |  |  |  |  |  | P:intracellular copper ion transport |
|  |  |  |  |  |  |  |  |  |  |  |  |  |  |  | P:siderophore-iron transport |
|  |  |  |  |  |  |  |  |  |  |  |  |  |  |  | P:positive regulation of biological process |
|  |  |  |  |  |  |  |  |  |  |  |  |  |  |  | P:positive regulation of carbohydrate metabolism |
|  |  |  |  |  |  |  |  |  |  |  |  |  |  |  | P:positive regulation of cellular process |
|  |  |  |  |  |  |  |  |  |  |  |  |  |  |  | P:positive regulation of cellular physiological process |
|  |  |  |  |  |  |  |  |  |  |  |  |  |  |  | P:positive regulation of physiological process |
|  |  |  |  |  |  |  |  |  |  |  |  |  |  |  | P:cell homeostasis |
|  |  |  |  |  |  |  |  |  |  |  |  |  |  |  | P:homeostasis |
|  |  |  |  |  |  |  |  |  |  |  |  |  |  |  | P:mitochondrial iron ion transport |
|  |  |  |  |  |  |  |  |  |  |  |  |  |  |  | P:metal ion transport |
|  |  |  |  |  |  |  |  |  |  |  |  |  |  |  | P:di-, tri-valent inorganic cation homeostasis |
|  |  |  |  |  |  |  |  |  |  |  |  |  |  |  | P:di-, tri-valent inorganic cation transport |
|  |  |  |  |  |  |  |  |  |  |  |  |  |  |  | P:transition metal ion homeostasis |
|  |  |  |  |  |  |  |  |  |  |  |  |  |  |  | P:transition metal ion transport |
|  |  |  |  |  |  |  |  |  |  |  |  |  |  |  | P:metal ion homeostasis |
|  |  |  |  |  |  |  |  |  |  |  |  |  |  |  | P:iron ion homeostasis |
|  |  |  |  |  |  |  |  |  |  |  |  |  |  |  | P:siderophore metabolism |
|  |  |  |  |  |  |  |  |  |  |  |  |  |  |  | P:ion transport |
|  |  |  |  |  |  |  |  |  |  |  |  |  |  |  | P:iron ion transport |
|  |  |  |  |  |  |  |  |  |  |  |  |  |  |  | P:cation transport |
|  |  |  |  |  |  |  |  |  |  |  |  |  |  |  | P:siderophore transport |
|  |  |  |  |  |  |  |  |  |  |  |  |  |  |  | P:ion homeostasis |
|  |  |  |  |  |  |  |  |  |  |  |  |  |  |  | P:cation homeostasis |
|  |  |  |  |  |  |  |  |  |  |  |  |  |  |  | P:cell ion homeostasis |
|  |  |  |  |  |  |  |  |  |  |  |  |  |  |  | P:physiological process |
|  |  |  |  |  |  |  |  |  |  |  |  |  |  |  | P:cellular physiological process |
|  |  |  |  |  |  |  |  |  |  |  |  |  |  |  | P:cellular process |
|  |  |  |  |  |  |  |  |  |  |  |  |  |  |  | P:mitochondrial electron transport, NADH to ubiquinone |
|  |  |  |  |  |  |  |  |  |  |  |  |  |  |  | P:positive regulation of gluconeogenesis |
|  |  |  |  |  |  |  |  |  |  |  |  |  |  |  | P:regulation of programmed cell death |
|  |  |  |  |  |  |  |  |  |  |  |  |  |  |  | P:regulation of apoptosis |
|  |  |  |  |  |  |  |  |  |  |  |  |  |  |  | P:sodium ion homeostasis |
|  |  |  |  |  |  |  |  |  |  |  |  |  |  |  | P:positive regulation of programmed cell death |
|  |  |  |  |  |  |  |  |  |  |  |  |  |  |  | P:positive regulation of apoptosis |
|  |  |  |  |  |  |  |  |  |  |  |  |  |  |  | P:malate metabolism |
|  |  |  |  |  |  |  |  |  |  |  |  |  |  |  | P:regulation of signal transduction |
|  |  |  |  |  |  |  |  |  |  |  |  |  |  |  | P:response to chemical stimulus |
|  |  |  |  |  |  |  |  |  |  |  |  |  |  |  | P:regulation of G-protein coupled receptor protein signaling pathway |
|  |  |  |  |  |  |  |  |  |  |  |  |  |  |  | P:carbohydrate metabolism |
|  |  |  |  |  |  |  |  |  |  |  |  |  |  |  | P:formation of catalytic spliceosome for second transesterification step |
|  |  |  |  |  |  |  |  |  |  |  |  |  |  |  | P:protein complex disassembly |
|  |  |  |  |  |  |  |  |  |  |  |  |  |  |  | P:spliceosome disassembly |
|  |  |  |  |  |  |  |  |  |  |  |  |  |  |  | P:u2-type spliceosome disassembly |
|  |  |  |  |  |  |  |  |  |  |  |  |  |  |  | P:catabolism |
|  |  |  |  |  |  |  |  |  |  |  |  |  |  |  | P:cellular carbohydrate metabolism |
|  |  |  |  |  |  |  |  |  |  |  |  |  |  |  | P:response to stimulus |
|  |  |  |  |  |  |  |  |  |  |  |  |  |  |  | P:cellular catabolism |
|  |  |  |  |  |  |  |  |  |  |  |  |  |  |  | P:cellular carbohydrate catabolism |
|  |  |  |  |  |  |  |  |  |  |  |  |  |  |  | P:carbohydrate catabolism |
|  |  |  |  |  |  |  |  |  |  |  |  |  |  |  | P:pentose catabolism |
|  |  |  |  |  |  |  |  |  |  |  |  |  |  |  | P:arabinose catabolism |
|  |  |  |  |  |  |  |  |  |  |  |  |  |  |  | P:arabinose metabolism |
|  |  |  |  |  |  |  |  |  |  |  |  |  |  |  | P:d-xylose metabolism |
|  |  |  |  |  |  |  |  |  |  |  |  |  |  |  | P:d-xylose catabolism |
|  |  |  |  |  |  |  |  |  |  |  |  |  |  |  | P:response to extracellular stimulus |
|  |  |  |  |  |  |  |  |  |  |  |  |  |  |  | P:response to nutrient levels |
|  |  |  |  |  |  |  |  |  |  |  |  |  |  |  | P:response to external stimulus |
|  |  |  |  |  |  |  |  |  |  |  |  |  |  |  | P:sterol biosynthesis |
|  |  |  |  |  |  |  |  |  |  |  |  |  |  |  | P:alcohol biosynthesis |
|  |  |  |  |  |  |  |  |  |  |  |  |  |  |  | P:hexose biosynthesis |
|  |  |  |  |  |  |  |  |  |  |  |  |  |  |  | P:monosaccharide biosynthesis |
|  |  |  |  |  |  |  |  |  |  |  |  |  |  |  | P:alcohol metabolism |
|  |  |  |  |  |  |  |  |  |  |  |  |  |  |  | P:gluconeogenesis |
|  |  |  |  |  |  |  |  |  |  |  |  |  |  |  | P:formation of catalytic U2-type spliceosome for second transesterification step |
|  |  |  |  |  |  |  |  |  |  |  |  |  |  |  | P:cell redox homeostasis |
|  |  |  |  |  |  |  |  |  |  |  |  |  |  |  | P:regulation of cell redox homeostasis |
|
| Cin5 | Yap7 | Rlm1 | Msn2 | Msn4 | Ume6 | Sut1 | Stp1 | Pho2 | Aft2 | Put3 | Hsf1 | Gln3 | Gat1 | Dal82 | Molecular Function |
|  |  |  |  |  |  |  |  |  |  |  |  |  |  |  | F:6-phosphogluconolactonase activity |
|  |  |  |  |  |  |  |  |  |  |  |  |  |  |  | F:oxidoreductase activity |
|  |  |  |  |  |  |  |  |  |  |  |  |  |  |  | F:oxidoreductase activity, acting on NADH or NADPH, heme protein as acceptor |
|  |  |  |  |  |  |  |  |  |  |  |  |  |  |  | F:catalase activity |
|  |  |  |  |  |  |  |  |  |  |  |  |  |  |  | F:aldehyde reductase activity |
|  |  |  |  |  |  |  |  |  |  |  |  |  |  |  | F:l-malate dehydrogenase activity |
|  |  |  |  |  |  |  |  |  |  |  |  |  |  |  | F:second spliceosomal transesterification activity |
|  |  |  |  |  |  |  |  |  |  |  |  |  |  |  | F:spliceosomal catalysis |
|  |  |  |  |  |  |  |  |  |  |  |  |  |  |  | F:malate dehydrogenase activity |
|  |  |  |  |  |  |  |  |  |  |  |  |  |  |  | F:oxidoreductase activity, acting on the CH-OH group of donors, NAD or NADP as acceptor |
|  |  |  |  |  |  |  |  |  |  |  |  |  |  |  | F:4-aminobutyrate transaminase activity |
|  |  |  |  |  |  |  |  |  |  |  |  |  |  |  | F:transporter activity |
|  |  |  |  |  |  |  |  |  |  |  |  |  |  |  | F:choline transporter activity |
|  |  |  |  |  |  |  |  |  |  |  |  |  |  |  | F:metal ion transporter activity |
|  |  |  |  |  |  |  |  |  |  |  |  |  |  |  | F:di-, tri-valent inorganic cation transporter activity |
|  |  |  |  |  |  |  |  |  |  |  |  |  |  |  | F:ion transporter activity |
|  |  |  |  |  |  |  |  |  |  |  |  |  |  |  | F:transition metal ion transporter activity |
|  |  |  |  |  |  |  |  |  |  |  |  |  |  |  | F:iron ion transporter activity |
|  |  |  |  |  |  |  |  |  |  |  |  |  |  |  | F:cation transporter activity |
|  |  |  |  |  |  |  |  |  |  |  |  |  |  |  | F:siderophore-iron transporter activity |
|  |  |  |  |  |  |  |  |  |  |  |  |  |  |  | F:siderophore transporter activity |
|  |  |  |  |  |  |  |  |  |  |  |  |  |  |  | F:nADH dehydrogenase activity |
|  |  |  |  |  |  |  |  |  |  |  |  |  |  |  | F:nADH dehydrogenase (quinone) activity |
|  |  |  |  |  |  |  |  |  |  |  |  |  |  |  | F:oxidoreductase activity, acting on NADH or NADPH, quinone or similar compound as acceptor |
|  |  |  |  |  |  |  |  |  |  |  |  |  |  |  | F:nADH dehydrogenase (ubiquinone) activity |
|  |  |  |  |  |  |  |  |  |  |  |  |  |  |  | F:transcription regulator activity |
|  |  |  |  |  |  |  |  |  |  |  |  |  |  |  | F:transcription factor activity |
|  |  |  |  |  |  |  |  |  |  |  |  |  |  |  | F:oxo-acid-lyase activity |
|  |  |  |  |  |  |  |  |  |  |  |  |  |  |  | F:methylisocitrate lyase activity |
|  |  |  |  |  |  |  |  |  |  |  |  |  |  |  | F:glucan 1,4-alpha-glucosidase activity |
|  |  |  |  |  |  |  |  |  |  |  |  |  |  |  | F:molecular function unknown |
|  |  |  |  |  |  |  |  |  |  |  |  |  |  |  | F:aMP-activated protein kinase activity |
|  |  |  |  |  |  |  |  |  |  |  |  |  |  |  | F:dodecenoyl-CoA delta-isomerase activity |
|  |  |  |  |  |  |  |  |  |  |  |  |  |  |  | F:nicotinate-nucleotide adenylyltransferase activity |
|  |  |  |  |  |  |  |  |  |  |  |  |  |  |  | F:serine-type endopeptidase activity |
|  |  |  |  |  |  |  |  |  |  |  |  |  |  |  | F:succinyltransferase activity |
|  |  |  |  |  |  |  |  |  |  |  |  |  |  |  | F:s-succinyltransferase activity |
|  |  |  |  |  |  |  |  |  |  |  |  |  |  |  | F:dihydrolipoyllysine-residue succinyltransferase activity |
|  |  |  |  |  |  |  |  |  |  |  |  |  |  |  | F:sterol 3-beta-glucosyltransferase activity |
|  |  |  |  |  |  |  |  |  |  |  |  |  |  |  | F:steroid binding |
|  |  |  |  |  |  |  |  |  |  |  |  |  |  |  | F:oxysterol binding |
|
| Cin5 | Yap7 | Rlm1 | Msn2 | Msn4 | Ume6 | Sut1 | Stp1 | Pho2 | Aft2 | Put3 | Hsf1 | Gln3 | Gat1 | Dal82 | Cellular Component |
|  |  |  |  |  |  |  |  |  |  |  |  |  |  |  | C:cytoplasmic membrane-bound vesicle |
|  |  |  |  |  |  |  |  |  |  |  |  |  |  |  | C:vesicle |
|  |  |  |  |  |  |  |  |  |  |  |  |  |  |  | C:membrane-bound vesicle |
|  |  |  |  |  |  |  |  |  |  |  |  |  |  |  | C:trans-Golgi network transport vesicle membrane |
|  |  |  |  |  |  |  |  |  |  |  |  |  |  |  | C:cytoplasmic vesicle |
|  |  |  |  |  |  |  |  |  |  |  |  |  |  |  | C:smc5-Smc6 complex |
|  |  |  |  |  |  |  |  |  |  |  |  |  |  |  | C:vacuolar lumen (sensu Fungi) |
|  |  |  |  |  |  |  |  |  |  |  |  |  |  |  | C:oxoglutarate dehydrogenase complex (sensu Eukaryota) |
|  |  |  |  |  |  |  |  |  |  |  |  |  |  |  | C:oxoglutarate dehydrogenase complex |
|  |  |  |  |  |  |  |  |  |  |  |  |  |  |  | C:intracellular organelle |
|  |  |  |  |  |  |  |  |  |  |  |  |  |  |  | C:organelle |
|  |  |  |  |  |  |  |  |  |  |  |  |  |  |  | C:membrane-bound organelle |
|  |  |  |  |  |  |  |  |  |  |  |  |  |  |  | C:intracellular membrane-bound organelle |
|  |  |  |  |  |  |  |  |  |  |  |  |  |  |  | C:vacuole |
|  |  |  |  |  |  |  |  |  |  |  |  |  |  |  | C:vacuole (sensu Fungi) |
|  |  |  |  |  |  |  |  |  |  |  |  |  |  |  | C:storage vacuole |
|  |  |  |  |  |  |  |  |  |  |  |  |  |  |  | C:lytic vacuole |
|
