# Supplementary material for: High-resolution analysis of condition-specific regulatory modules in Saccharomyces cerevisiae
Source: Genome Biol. 2008 Jan 3;9(1):R2. doi: 10.1186/gb-2008-9-1-r2 (PMC2395236; doi:10.1186/gb-2008-9-1-r2)
Supplement: Additional data file 11 — Matrices describing all EPMs and RMs, including lists of synergistic pairs of regulators. [file gb-2008-9-1-r2-S11.zip › htmls/C13_EPMs_matrix/EPM_22.Overlap.matrix.html]

|  |  |  |  |  |  |  |  |  |  |  |  |  |  |  |
| --- | --- | --- | --- | --- | --- | --- | --- | --- | --- | --- | --- | --- | --- | --- |
| Cin5 | Yap7 | Rlm1 | Msn2 | Msn4 | Ume6 | Sut1 | Stp1 | Pho2 | Aft2 | Put3 | Hsf1 | Gln3 | Gat1 | Dal82 |
|  |  |  |  |  |  |  |  |  |  |  |  |  |  |  | Cin5 |
|  |  |  |  |  |  |  |  |  |  |  |  |  |  |  | Yap7 |
|  |  |  |  |  |  |  |  |  |  |  |  |  |  |  | Rlm1 |
|  |  |  |  |  |  |  |  |  |  |  |  |  |  |  | Msn2 |
|  |  |  |  |  |  |  |  |  |  |  |  |  |  |  | Msn4 |
|  |  |  |  |  |  |  |  |  |  |  |  |  |  |  | Ume6 |
|  |  |  |  |  |  |  |  |  |  |  |  |  |  |  | Sut1 |
|  |  |  |  |  |  |  |  |  |  |  |  |  |  |  | Stp1 |
|  |  |  |  |  |  |  |  |  |  |  |  |  |  |  | Pho2 |
|  |  |  |  |  |  |  |  |  |  |  |  |  |  |  | Aft2 |
|  |  |  |  |  |  |  |  |  |  |  |  |  |  |  | Put3 |
|  |  |  |  |  |  |  |  |  |  |  |  |  |  |  | Hsf1 |
|  |  |  |  |  |  |  |  |  |  |  |  |  |  |  | Gln3 |
|  |  |  |  |  |  |  |  |  |  |  |  |  |  |  | Gat1 |
|  |  |  |  |  |  |  |  |  |  |  |  |  |  |  | Dal82 |
 Cin5 | Yap7 | Rlm1 | Msn2 | Msn4 | Ume6 | Sut1 | Stp1 | Pho2 | Aft2 | Put3 | Hsf1 | Gln3 | Gat1 | Dal82 |
