# Supplementary material for: High-resolution analysis of condition-specific regulatory modules in Saccharomyces cerevisiae
Source: Genome Biol. 2008 Jan 3;9(1):R2. doi: 10.1186/gb-2008-9-1-r2 (PMC2395236; doi:10.1186/gb-2008-9-1-r2)
Supplement: Additional data file 11 — Matrices describing all EPMs and RMs, including lists of synergistic pairs of regulators. [file gb-2008-9-1-r2-S11.zip › htmls/C13_EPMs_matrix/EPM_22.RM.matrix.html]

Regulators vs. RM target gene list

|  |  |  |  |  |  |  |  |  |  |  |  |  |  |  |  |
| --- | --- | --- | --- | --- | --- | --- | --- | --- | --- | --- | --- | --- | --- | --- | --- |
|  | Cin5 | Yap7 | Rlm1 | Msn2 | Msn4 | Ume6 | Sut1 | Stp1 | Pho2 | Aft2 | Put3 | Hsf1 | Gln3 | Gat1 | Dal82 |
| RM\_1 |  |  |  |  |  |  |  |  |  |  |  |  |  |  |  |
| RM\_2 |  |  |  |  |  |  |  |  |  |  |  |  |  |  |  |
| RM\_3 |  |  |  |  |  |  |  |  |  |  |  |  |  |  |  |
| RM\_4 |  |  |  |  |  |  |  |  |  |  |  |  |  |  |  |
| RM\_5 |  |  |  |  |  |  |  |  |  |  |  |  |  |  |  |
| RM\_6 |  |  |  |  |  |  |  |  |  |  |  |  |  |  |  |
| RM\_7 |  |  |  |  |  |  |  |  |  |  |  |  |  |  |  |
| RM\_8 |  |  |  |  |  |  |  |  |  |  |  |  |  |  |  |
| RM\_9 |  |  |  |  |  |  |  |  |  |  |  |  |  |  |  |
| RM\_10 |  |  |  |  |  |  |  |  |  |  |  |  |  |  |  |

Synergistic Pair of Regulators

1. Msn2\*Msn4

2. Stp1\*Ume6

Matrix of enriched GO

EPM matrix
