# Supplementary material for: High-resolution analysis of condition-specific regulatory modules in Saccharomyces cerevisiae
Source: Genome Biol. 2008 Jan 3;9(1):R2. doi: 10.1186/gb-2008-9-1-r2 (PMC2395236; doi:10.1186/gb-2008-9-1-r2)
Supplement: Additional data file 11 — Matrices describing all EPMs and RMs, including lists of synergistic pairs of regulators. [file gb-2008-9-1-r2-S11.zip › htmls/C13_EPMs_matrix/EPM_23.GO_enrichment.matrix.html]

|  |  |  |  |  |
| --- | --- | --- | --- | --- |
| Cin5 | Ume6 | Stp1 | Sut1 | Biological Process |
|  |  |  |  | P:biological process unknown |
|  |  |  |  | P:biotin biosynthesis |
|  |  |  |  | P:sodium ion homeostasis |
|  |  |  |  | P:biotin metabolism |
|
| Cin5 | Ume6 | Stp1 | Sut1 | Molecular Function |
|  |  |  |  | F:molecular function unknown |
|  |  |  |  | F:transcription factor activity |
|  |  |  |  | F:c-22 sterol desaturase activity |
|  |  |  |  | F:dNA binding |
|
| Cin5 | Ume6 | Stp1 | Sut1 | Cellular Component |
|  |  |  |  | C:vacuole |
|  |  |  |  | C:vacuole (sensu Fungi) |
|  |  |  |  | C:storage vacuole |
|  |  |  |  | C:lytic vacuole |
|
