# Supplementary material for: High-resolution analysis of condition-specific regulatory modules in Saccharomyces cerevisiae
Source: Genome Biol. 2008 Jan 3;9(1):R2. doi: 10.1186/gb-2008-9-1-r2 (PMC2395236; doi:10.1186/gb-2008-9-1-r2)
Supplement: Additional data file 11 — Matrices describing all EPMs and RMs, including lists of synergistic pairs of regulators. [file gb-2008-9-1-r2-S11.zip › htmls/C13_EPMs_matrix/EPM_24.GO_enrichment.matrix.html]

|  |  |  |  |  |  |  |  |  |  |  |  |  |  |  |  |  |  |
| --- | --- | --- | --- | --- | --- | --- | --- | --- | --- | --- | --- | --- | --- | --- | --- | --- | --- |
| Hap2 | Hap4 | Cin5 | Yap6 | Ash1 | Mig1 | Skn7 | Pdr3 | Gal80 | Pho2 | Sut1 | Msn2 | Msn4 | Sok2 | Mcm1 | Rlm1 | Pdr1 | Biological Process |
|  |  |  |  |  |  |  |  |  |  |  |  |  |  |  |  |  | P:fatty acid transport |
|  |  |  |  |  |  |  |  |  |  |  |  |  |  |  |  |  | P:biotin metabolism |
|  |  |  |  |  |  |  |  |  |  |  |  |  |  |  |  |  | P:biotin biosynthesis |
|  |  |  |  |  |  |  |  |  |  |  |  |  |  |  |  |  | P:generation of precursor metabolites and energy |
|  |  |  |  |  |  |  |  |  |  |  |  |  |  |  |  |  | P:phosphate metabolism |
|  |  |  |  |  |  |  |  |  |  |  |  |  |  |  |  |  | P:phosphorus metabolism |
|  |  |  |  |  |  |  |  |  |  |  |  |  |  |  |  |  | P:phosphorylation |
|  |  |  |  |  |  |  |  |  |  |  |  |  |  |  |  |  | P:cellular respiration |
|  |  |  |  |  |  |  |  |  |  |  |  |  |  |  |  |  | P:oxidative phosphorylation |
|  |  |  |  |  |  |  |  |  |  |  |  |  |  |  |  |  | P:aerobic respiration |
|  |  |  |  |  |  |  |  |  |  |  |  |  |  |  |  |  | P:mitochondrial electron transport, ubiquinol to cytochrome c |
|  |  |  |  |  |  |  |  |  |  |  |  |  |  |  |  |  | P:cell communication |
|  |  |  |  |  |  |  |  |  |  |  |  |  |  |  |  |  | P:signal transduction |
|  |  |  |  |  |  |  |  |  |  |  |  |  |  |  |  |  | P:unfolded protein response |
|  |  |  |  |  |  |  |  |  |  |  |  |  |  |  |  |  | P:eR-nuclear signaling pathway |
|  |  |  |  |  |  |  |  |  |  |  |  |  |  |  |  |  | P:intracellular signaling cascade |
|  |  |  |  |  |  |  |  |  |  |  |  |  |  |  |  |  | P:unfolded protein response, positive regulation of target gene transcription |
|  |  |  |  |  |  |  |  |  |  |  |  |  |  |  |  |  | P:positive regulation of gene-specific transcription |
|  |  |  |  |  |  |  |  |  |  |  |  |  |  |  |  |  | P:adaptation to pheromone during conjugation with cellular fusion |
|  |  |  |  |  |  |  |  |  |  |  |  |  |  |  |  |  | P:g1/S-specific transcription in mitotic cell cycle |
|  |  |  |  |  |  |  |  |  |  |  |  |  |  |  |  |  | P:reproductive cellular physiological process |
|  |  |  |  |  |  |  |  |  |  |  |  |  |  |  |  |  | P:myo-inositol metabolism |
|  |  |  |  |  |  |  |  |  |  |  |  |  |  |  |  |  | P:reproductive physiological process |
|  |  |  |  |  |  |  |  |  |  |  |  |  |  |  |  |  | P:proline metabolism |
|  |  |  |  |  |  |  |  |  |  |  |  |  |  |  |  |  | P:catabolism |
|  |  |  |  |  |  |  |  |  |  |  |  |  |  |  |  |  | P:dicarboxylic acid metabolism |
|  |  |  |  |  |  |  |  |  |  |  |  |  |  |  |  |  | P:cellular catabolism |
|  |  |  |  |  |  |  |  |  |  |  |  |  |  |  |  |  | P:regulation of glycogen biosynthesis |
|  |  |  |  |  |  |  |  |  |  |  |  |  |  |  |  |  | P:regulation of glycogen catabolism |
|  |  |  |  |  |  |  |  |  |  |  |  |  |  |  |  |  | P:energy derivation by oxidation of organic compounds |
|  |  |  |  |  |  |  |  |  |  |  |  |  |  |  |  |  | P:proline catabolism |
|  |  |  |  |  |  |  |  |  |  |  |  |  |  |  |  |  | P:carbohydrate biosynthesis |
|  |  |  |  |  |  |  |  |  |  |  |  |  |  |  |  |  | P:mAPKKK cascade during cell wall biogenesis |
|  |  |  |  |  |  |  |  |  |  |  |  |  |  |  |  |  | P:cellular polysaccharide metabolism |
|  |  |  |  |  |  |  |  |  |  |  |  |  |  |  |  |  | P:polysaccharide metabolism |
|  |  |  |  |  |  |  |  |  |  |  |  |  |  |  |  |  | P:cellular carbohydrate catabolism |
|  |  |  |  |  |  |  |  |  |  |  |  |  |  |  |  |  | P:carbohydrate catabolism |
|  |  |  |  |  |  |  |  |  |  |  |  |  |  |  |  |  | P:polysaccharide catabolism |
|  |  |  |  |  |  |  |  |  |  |  |  |  |  |  |  |  | P:cellular polysaccharide catabolism |
|  |  |  |  |  |  |  |  |  |  |  |  |  |  |  |  |  | P:glycogen catabolism |
|  |  |  |  |  |  |  |  |  |  |  |  |  |  |  |  |  | P:glucan catabolism |
|  |  |  |  |  |  |  |  |  |  |  |  |  |  |  |  |  | P:cellular carbohydrate metabolism |
|  |  |  |  |  |  |  |  |  |  |  |  |  |  |  |  |  | P:carbohydrate metabolism |
|  |  |  |  |  |  |  |  |  |  |  |  |  |  |  |  |  | P:neutral amino acid transport |
|  |  |  |  |  |  |  |  |  |  |  |  |  |  |  |  |  | P:glycogen metabolism |
|  |  |  |  |  |  |  |  |  |  |  |  |  |  |  |  |  | P:energy reserve metabolism |
|  |  |  |  |  |  |  |  |  |  |  |  |  |  |  |  |  | P:glucan metabolism |
|  |  |  |  |  |  |  |  |  |  |  |  |  |  |  |  |  | P:malate metabolism |
|  |  |  |  |  |  |  |  |  |  |  |  |  |  |  |  |  | P:response to carbohydrate stimulus |
|  |  |  |  |  |  |  |  |  |  |  |  |  |  |  |  |  | P:response to glucose stimulus |
|  |  |  |  |  |  |  |  |  |  |  |  |  |  |  |  |  | P:response to hexose stimulus |
|  |  |  |  |  |  |  |  |  |  |  |  |  |  |  |  |  | P:transcription from RNA polymerase II promoter |
|  |  |  |  |  |  |  |  |  |  |  |  |  |  |  |  |  | P:regulation of transcription, DNA-dependent |
|  |  |  |  |  |  |  |  |  |  |  |  |  |  |  |  |  | P:regulation of transcription from RNA polymerase II promoter |
|  |  |  |  |  |  |  |  |  |  |  |  |  |  |  |  |  | P:regulation of transcription |
|  |  |  |  |  |  |  |  |  |  |  |  |  |  |  |  |  | P:regulation of nucleobase, nucleoside, nucleotide and nucleic acid metabolism |
|  |  |  |  |  |  |  |  |  |  |  |  |  |  |  |  |  | P:transcription |
|  |  |  |  |  |  |  |  |  |  |  |  |  |  |  |  |  | P:glucose transport |
|  |  |  |  |  |  |  |  |  |  |  |  |  |  |  |  |  | P:re-entry into mitotic cell cycle |
|  |  |  |  |  |  |  |  |  |  |  |  |  |  |  |  |  | P:re-entry into mitotic cell cycle after pheromone arrest |
|  |  |  |  |  |  |  |  |  |  |  |  |  |  |  |  |  | P:regulation of cellular metabolism |
|  |  |  |  |  |  |  |  |  |  |  |  |  |  |  |  |  | P:regulation of metabolism |
|
| Hap2 | Hap4 | Cin5 | Yap6 | Ash1 | Mig1 | Skn7 | Pdr3 | Gal80 | Pho2 | Sut1 | Msn2 | Msn4 | Sok2 | Mcm1 | Rlm1 | Pdr1 | Molecular Function |
|  |  |  |  |  |  |  |  |  |  |  |  |  |  |  |  |  | F:ras guanyl-nucleotide exchange factor activity |
|  |  |  |  |  |  |  |  |  |  |  |  |  |  |  |  |  | F:hydrogen-transporting ATP synthase activity, rotational mechanism |
|  |  |  |  |  |  |  |  |  |  |  |  |  |  |  |  |  | F:cytochrome-c oxidase activity |
|  |  |  |  |  |  |  |  |  |  |  |  |  |  |  |  |  | F:heme-copper terminal oxidase activity |
|  |  |  |  |  |  |  |  |  |  |  |  |  |  |  |  |  | F:oxidoreductase activity, acting on heme group of donors, oxygen as acceptor |
|  |  |  |  |  |  |  |  |  |  |  |  |  |  |  |  |  | F:oxidoreductase activity, acting on heme group of donors |
|  |  |  |  |  |  |  |  |  |  |  |  |  |  |  |  |  | F:transporter activity |
|  |  |  |  |  |  |  |  |  |  |  |  |  |  |  |  |  | F:ion transporter activity |
|  |  |  |  |  |  |  |  |  |  |  |  |  |  |  |  |  | F:cation transporter activity |
|  |  |  |  |  |  |  |  |  |  |  |  |  |  |  |  |  | F:hydrogen ion transporter activity |
|  |  |  |  |  |  |  |  |  |  |  |  |  |  |  |  |  | F:monovalent inorganic cation transporter activity |
|  |  |  |  |  |  |  |  |  |  |  |  |  |  |  |  |  | F:ubiquinol-cytochrome-c reductase activity |
|  |  |  |  |  |  |  |  |  |  |  |  |  |  |  |  |  | F:oxidoreductase activity, acting on diphenols and related substances as donors |
|  |  |  |  |  |  |  |  |  |  |  |  |  |  |  |  |  | F:oxidoreductase activity, acting on diphenols and related substances as donors, cytochrome as acceptor |
|  |  |  |  |  |  |  |  |  |  |  |  |  |  |  |  |  | F:structural constituent of cell wall |
|  |  |  |  |  |  |  |  |  |  |  |  |  |  |  |  |  | F:transcriptional repressor activity |
|  |  |  |  |  |  |  |  |  |  |  |  |  |  |  |  |  | F:dNA binding |
|  |  |  |  |  |  |  |  |  |  |  |  |  |  |  |  |  | F:intramolecular lyase activity |
|  |  |  |  |  |  |  |  |  |  |  |  |  |  |  |  |  | F:inositol-3-phosphate synthase activity |
|  |  |  |  |  |  |  |  |  |  |  |  |  |  |  |  |  | F:glucan 1,4-alpha-glucosidase activity |
|  |  |  |  |  |  |  |  |  |  |  |  |  |  |  |  |  | F:transcription factor activity |
|  |  |  |  |  |  |  |  |  |  |  |  |  |  |  |  |  | F:transcription regulator activity |
|  |  |  |  |  |  |  |  |  |  |  |  |  |  |  |  |  | F:neutral amino acid transporter activity |
|  |  |  |  |  |  |  |  |  |  |  |  |  |  |  |  |  | F:oxidoreductase activity, acting on the CH-OH group of donors, NAD or NADP as acceptor |
|  |  |  |  |  |  |  |  |  |  |  |  |  |  |  |  |  | F:malate dehydrogenase activity |
|  |  |  |  |  |  |  |  |  |  |  |  |  |  |  |  |  | F:l-proline transporter activity |
|  |  |  |  |  |  |  |  |  |  |  |  |  |  |  |  |  | F:l-proline permease activity |
|  |  |  |  |  |  |  |  |  |  |  |  |  |  |  |  |  | F:l-malate dehydrogenase activity |
|  |  |  |  |  |  |  |  |  |  |  |  |  |  |  |  |  | F:mAP kinase phosphatase activity |
|  |  |  |  |  |  |  |  |  |  |  |  |  |  |  |  |  | F:protein tyrosine/serine/threonine phosphatase activity |
|  |  |  |  |  |  |  |  |  |  |  |  |  |  |  |  |  | F:oxidoreductase activity, acting on CH2 groups |
|  |  |  |  |  |  |  |  |  |  |  |  |  |  |  |  |  | F:ribonucleoside-diphosphate reductase activity |
|  |  |  |  |  |  |  |  |  |  |  |  |  |  |  |  |  | F:oxidoreductase activity, acting on CH2 groups, disulfide as acceptor |
|  |  |  |  |  |  |  |  |  |  |  |  |  |  |  |  |  | F:d-lactaldehyde dehydrogenase activity |
|  |  |  |  |  |  |  |  |  |  |  |  |  |  |  |  |  | F:oxidoreductase activity, acting on CH-OH group of donors |
|
| Hap2 | Hap4 | Cin5 | Yap6 | Ash1 | Mig1 | Skn7 | Pdr3 | Gal80 | Pho2 | Sut1 | Msn2 | Msn4 | Sok2 | Mcm1 | Rlm1 | Pdr1 | Cellular Component |
|  |  |  |  |  |  |  |  |  |  |  |  |  |  |  |  |  | C:cellular component unknown |
|  |  |  |  |  |  |  |  |  |  |  |  |  |  |  |  |  | C:ribonucleoside-diphosphate reductase complex |
|  |  |  |  |  |  |  |  |  |  |  |  |  |  |  |  |  | C:proton-transporting ATP synthase complex, coupling factor F(o) (sensu Eukaryota) |
|  |  |  |  |  |  |  |  |  |  |  |  |  |  |  |  |  | C:respiratory chain complex IV |
|  |  |  |  |  |  |  |  |  |  |  |  |  |  |  |  |  | C:proton-transporting ATP synthase complex, coupling factor F(o) |
|  |  |  |  |  |  |  |  |  |  |  |  |  |  |  |  |  | C:respiratory chain complex IV (sensu Eukaryota) |
|  |  |  |  |  |  |  |  |  |  |  |  |  |  |  |  |  | C:organelle membrane |
|  |  |  |  |  |  |  |  |  |  |  |  |  |  |  |  |  | C:membrane part |
|  |  |  |  |  |  |  |  |  |  |  |  |  |  |  |  |  | C:mitochondrial part |
|  |  |  |  |  |  |  |  |  |  |  |  |  |  |  |  |  | C:proton-transporting ATP synthase, stator stalk |
|  |  |  |  |  |  |  |  |  |  |  |  |  |  |  |  |  | C:proton-transporting ATP synthase, stator stalk (sensu Eukaryota) |
|  |  |  |  |  |  |  |  |  |  |  |  |  |  |  |  |  | C:envelope |
|  |  |  |  |  |  |  |  |  |  |  |  |  |  |  |  |  | C:organelle envelope |
|  |  |  |  |  |  |  |  |  |  |  |  |  |  |  |  |  | C:mitochondrial envelope |
|  |  |  |  |  |  |  |  |  |  |  |  |  |  |  |  |  | C:mitochondrial membrane |
|  |  |  |  |  |  |  |  |  |  |  |  |  |  |  |  |  | C:organelle inner membrane |
|  |  |  |  |  |  |  |  |  |  |  |  |  |  |  |  |  | C:mitochondrial inner membrane |
|  |  |  |  |  |  |  |  |  |  |  |  |  |  |  |  |  | C:mitochondrial membrane part |
|  |  |  |  |  |  |  |  |  |  |  |  |  |  |  |  |  | C:mitochondrial electron transport chain |
|  |  |  |  |  |  |  |  |  |  |  |  |  |  |  |  |  | C:ubiquinol-cytochrome-c reductase complex |
|  |  |  |  |  |  |  |  |  |  |  |  |  |  |  |  |  | C:respiratory chain complex III (sensu Eukaryota) |
|  |  |  |  |  |  |  |  |  |  |  |  |  |  |  |  |  | C:respiratory chain complex III |
|
