# Supplementary material for: High-resolution analysis of condition-specific regulatory modules in Saccharomyces cerevisiae
Source: Genome Biol. 2008 Jan 3;9(1):R2. doi: 10.1186/gb-2008-9-1-r2 (PMC2395236; doi:10.1186/gb-2008-9-1-r2)
Supplement: Additional data file 11 — Matrices describing all EPMs and RMs, including lists of synergistic pairs of regulators. [file gb-2008-9-1-r2-S11.zip › htmls/C13_EPMs_matrix/EPM_24.Overlap.matrix.html]

|  |  |  |  |  |  |  |  |  |  |  |  |  |  |  |  |  |
| --- | --- | --- | --- | --- | --- | --- | --- | --- | --- | --- | --- | --- | --- | --- | --- | --- |
| Hap2 | Hap4 | Cin5 | Yap6 | Ash1 | Mig1 | Skn7 | Pdr3 | Gal80 | Pho2 | Sut1 | Msn2 | Msn4 | Sok2 | Mcm1 | Rlm1 | Pdr1 |
|  |  |  |  |  |  |  |  |  |  |  |  |  |  |  |  |  | Hap2 |
|  |  |  |  |  |  |  |  |  |  |  |  |  |  |  |  |  | Hap4 |
|  |  |  |  |  |  |  |  |  |  |  |  |  |  |  |  |  | Cin5 |
|  |  |  |  |  |  |  |  |  |  |  |  |  |  |  |  |  | Yap6 |
|  |  |  |  |  |  |  |  |  |  |  |  |  |  |  |  |  | Ash1 |
|  |  |  |  |  |  |  |  |  |  |  |  |  |  |  |  |  | Mig1 |
|  |  |  |  |  |  |  |  |  |  |  |  |  |  |  |  |  | Skn7 |
|  |  |  |  |  |  |  |  |  |  |  |  |  |  |  |  |  | Pdr3 |
|  |  |  |  |  |  |  |  |  |  |  |  |  |  |  |  |  | Gal80 |
|  |  |  |  |  |  |  |  |  |  |  |  |  |  |  |  |  | Pho2 |
|  |  |  |  |  |  |  |  |  |  |  |  |  |  |  |  |  | Sut1 |
|  |  |  |  |  |  |  |  |  |  |  |  |  |  |  |  |  | Msn2 |
|  |  |  |  |  |  |  |  |  |  |  |  |  |  |  |  |  | Msn4 |
|  |  |  |  |  |  |  |  |  |  |  |  |  |  |  |  |  | Sok2 |
|  |  |  |  |  |  |  |  |  |  |  |  |  |  |  |  |  | Mcm1 |
|  |  |  |  |  |  |  |  |  |  |  |  |  |  |  |  |  | Rlm1 |
|  |  |  |  |  |  |  |  |  |  |  |  |  |  |  |  |  | Pdr1 |
 Hap2 | Hap4 | Cin5 | Yap6 | Ash1 | Mig1 | Skn7 | Pdr3 | Gal80 | Pho2 | Sut1 | Msn2 | Msn4 | Sok2 | Mcm1 | Rlm1 | Pdr1 |
