# Supplementary material for: High-resolution analysis of condition-specific regulatory modules in Saccharomyces cerevisiae
Source: Genome Biol. 2008 Jan 3;9(1):R2. doi: 10.1186/gb-2008-9-1-r2 (PMC2395236; doi:10.1186/gb-2008-9-1-r2)
Supplement: Additional data file 11 — Matrices describing all EPMs and RMs, including lists of synergistic pairs of regulators. [file gb-2008-9-1-r2-S11.zip › htmls/C13_EPMs_matrix/EPM_24.RM.matrix.html]

Regulators vs. RM target gene list

|  |  |  |  |  |  |  |  |  |  |  |  |  |  |  |  |  |  |
| --- | --- | --- | --- | --- | --- | --- | --- | --- | --- | --- | --- | --- | --- | --- | --- | --- | --- |
|  | Hap2 | Hap4 | Cin5 | Yap6 | Ash1 | Mig1 | Skn7 | Pdr3 | Gal80 | Pho2 | Sut1 | Msn2 | Msn4 | Sok2 | Mcm1 | Rlm1 | Pdr1 |
| RM\_1 |  |  |  |  |  |  |  |  |  |  |  |  |  |  |  |  |  |
| RM\_2 |  |  |  |  |  |  |  |  |  |  |  |  |  |  |  |  |  |
| RM\_3 |  |  |  |  |  |  |  |  |  |  |  |  |  |  |  |  |  |
| RM\_4 |  |  |  |  |  |  |  |  |  |  |  |  |  |  |  |  |  |
| RM\_5 |  |  |  |  |  |  |  |  |  |  |  |  |  |  |  |  |  |
| RM\_6 |  |  |  |  |  |  |  |  |  |  |  |  |  |  |  |  |  |
| RM\_7 |  |  |  |  |  |  |  |  |  |  |  |  |  |  |  |  |  |
| RM\_8 |  |  |  |  |  |  |  |  |  |  |  |  |  |  |  |  |  |

Synergistic Pair of Regulators

1. Hap2\*Hap4

2. Ash1\*Yap6

3. Gal80\*Msn2

4. Gal80\*Msn4

5. Msn2\*Sut1

6. Msn4\*Sut1

7. Msn2\*Msn4

8. Msn4\*Pho2

9. Msn2\*Pho2

10. Gal80\*Pho2

11. Mig1\*Pdr3

12. Msn2\*Pdr3

13. Msn4\*Pdr3

14. Rlm1\*Sut1

15. Gal80\*Pdr3

Matrix of enriched GO

EPM matrix
