# Supplementary material for: High-resolution analysis of condition-specific regulatory modules in Saccharomyces cerevisiae
Source: Genome Biol. 2008 Jan 3;9(1):R2. doi: 10.1186/gb-2008-9-1-r2 (PMC2395236; doi:10.1186/gb-2008-9-1-r2)
Supplement: Additional data file 11 — Matrices describing all EPMs and RMs, including lists of synergistic pairs of regulators. [file gb-2008-9-1-r2-S11.zip › htmls/C13_EPMs_matrix/EPM_25.GO_enrichment.matrix.html]

|  |  |  |  |  |  |  |
| --- | --- | --- | --- | --- | --- | --- |
| Uga3 | Hap1 | Sut1 | Pho4 | Gal4 | Gat1 | Biological Process |
|  |  |  |  |  |  | P:energy derivation by oxidation of organic compounds |
|  |  |  |  |  |  | P:lipid biosynthesis |
|  |  |  |  |  |  | P:fatty acid desaturation |
|  |  |  |  |  |  | P:oxidative phosphorylation |
|  |  |  |  |  |  | P:electron transport |
|  |  |  |  |  |  | P:aTP synthesis coupled electron transport (sensu Eukaryota) |
|  |  |  |  |  |  | P:aTP synthesis coupled electron transport |
|  |  |  |  |  |  | P:lipid metabolism |
|  |  |  |  |  |  | P:generation of precursor metabolites and energy |
|  |  |  |  |  |  | P:steroid metabolism |
|  |  |  |  |  |  | P:cellular lipid metabolism |
|  |  |  |  |  |  | P:sterol metabolism |
|  |  |  |  |  |  | P:steroid biosynthesis |
|  |  |  |  |  |  | P:sterol biosynthesis |
|  |  |  |  |  |  | P:mitochondrial electron transport, cytochrome c to oxygen |
|  |  |  |  |  |  | P:ergosterol biosynthesis |
|  |  |  |  |  |  | P:ergosterol metabolism |
|  |  |  |  |  |  | P:positive regulation of glycolysis |
|  |  |  |  |  |  | P:carbohydrate catabolism |
|  |  |  |  |  |  | P:cellular carbohydrate catabolism |
|  |  |  |  |  |  | P:alcohol catabolism |
|  |  |  |  |  |  | P:monosaccharide catabolism |
|  |  |  |  |  |  | P:hexose catabolism |
|  |  |  |  |  |  | P:glucose catabolism |
|  |  |  |  |  |  | P:regulation of catabolism |
|  |  |  |  |  |  | P:glycolysis |
|  |  |  |  |  |  | P:fructose 2,6-bisphosphate metabolism |
|  |  |  |  |  |  | P:regulation of glycolysis |
|  |  |  |  |  |  | P:alcohol metabolism |
|  |  |  |  |  |  | P:fructose metabolism |
|  |  |  |  |  |  | P:monosaccharide metabolism |
|  |  |  |  |  |  | P:positive regulation of carbohydrate metabolism |
|  |  |  |  |  |  | P:hexose metabolism |
|  |  |  |  |  |  | P:glucose metabolism |
|  |  |  |  |  |  | P:main pathways of carbohydrate metabolism |
|  |  |  |  |  |  | P:cellular metabolism |
|  |  |  |  |  |  | P:response to stimulus |
|  |  |  |  |  |  | P:biotin metabolism |
|  |  |  |  |  |  | P:biotin biosynthesis |
|  |  |  |  |  |  | P:response to stress |
|  |  |  |  |  |  | P:nitrogen compound metabolism |
|  |  |  |  |  |  | P:amine metabolism |
|  |  |  |  |  |  | P:amino acid and derivative metabolism |
|  |  |  |  |  |  | P:amino acid metabolism |
|  |  |  |  |  |  | P:cell communication |
|  |  |  |  |  |  | P:organic acid metabolism |
|  |  |  |  |  |  | P:carboxylic acid metabolism |
|  |  |  |  |  |  | P:catabolism |
|  |  |  |  |  |  | P:cellular catabolism |
|  |  |  |  |  |  | P:aspartate family amino acid metabolism |
|  |  |  |  |  |  | P:glutamine family amino acid metabolism |
|  |  |  |  |  |  | P:amine catabolism |
|  |  |  |  |  |  | P:nitrogen compound catabolism |
|  |  |  |  |  |  | P:amino acid catabolism |
|  |  |  |  |  |  | P:response to extracellular stimulus |
|  |  |  |  |  |  | P:response to external stimulus |
|  |  |  |  |  |  | P:response to nutrient levels |
|  |  |  |  |  |  | P:cellular response to starvation |
|  |  |  |  |  |  | P:response to starvation |
|  |  |  |  |  |  | P:cellular response to stimulus |
|  |  |  |  |  |  | P:cellular response to extracellular stimulus |
|  |  |  |  |  |  | P:cellular response to nutrient levels |
|  |  |  |  |  |  | P:glutamine family amino acid catabolism |
|  |  |  |  |  |  | P:aspartate family amino acid catabolism |
|  |  |  |  |  |  | P:asparagine metabolism |
|  |  |  |  |  |  | P:cellular response to nitrogen levels |
|  |  |  |  |  |  | P:cellular response to nitrogen starvation |
|  |  |  |  |  |  | P:asparagine catabolism |
|
| Uga3 | Hap1 | Sut1 | Pho4 | Gal4 | Gat1 | Molecular Function |
|  |  |  |  |  |  | F:transcription factor activity |
|  |  |  |  |  |  | F:catalytic activity |
|  |  |  |  |  |  | F:sphingosine hydroxylase activity |
|  |  |  |  |  |  | F:6-phosphofructo-2-kinase activity |
|  |  |  |  |  |  | F:c-22 sterol desaturase activity |
|  |  |  |  |  |  | F:phosphofructokinase activity |
|  |  |  |  |  |  | F:oxidoreductase activity, acting on paired donors, with incorporation or reduction of molecular oxygen, NAD or NADH as one donor, and incorporation of one atom of oxygen |
|  |  |  |  |  |  | F:coA desaturase activity |
|  |  |  |  |  |  | F:oxidoreductase activity |
|  |  |  |  |  |  | F:stearoyl-CoA 9-desaturase activity |
|  |  |  |  |  |  | F:monovalent inorganic cation transporter activity |
|  |  |  |  |  |  | F:hydrogen ion transporter activity |
|  |  |  |  |  |  | F:sterol 14-demethylase activity |
|  |  |  |  |  |  | F:oxidoreductase activity, acting on paired donors, with incorporation or reduction of molecular oxygen |
|  |  |  |  |  |  | F:cytochrome-c oxidase activity |
|  |  |  |  |  |  | F:heme-copper terminal oxidase activity |
|  |  |  |  |  |  | F:oxidoreductase activity, acting on heme group of donors |
|  |  |  |  |  |  | F:oxidoreductase activity, acting on heme group of donors, oxygen as acceptor |
|  |  |  |  |  |  | F:alpha-1,2-mannosyltransferase activity |
|  |  |  |  |  |  | F:c-5 sterol desaturase activity |
|  |  |  |  |  |  | F:hydrolase activity, acting on carbon-nitrogen (but not peptide) bonds |
|  |  |  |  |  |  | F:asparaginase activity |
|  |  |  |  |  |  | F:hydrolase activity, acting on carbon-nitrogen (but not peptide) bonds, in linear amides |
|
| Uga3 | Hap1 | Sut1 | Pho4 | Gal4 | Gat1 | Cellular Component |
|  |  |  |  |  |  | C:periplasmic space (sensu Fungi) |
|  |  |  |  |  |  | C:periplasmic space |
|  |  |  |  |  |  | C:endoplasmic reticulum |
|  |  |  |  |  |  | C:cytoplasm |
|  |  |  |  |  |  | C:mitochondrial membrane part |
|  |  |  |  |  |  | C:mitochondrial electron transport chain |
|  |  |  |  |  |  | C:respiratory chain complex IV (sensu Eukaryota) |
|  |  |  |  |  |  | C:respiratory chain complex IV |
|
