# Supplementary material for: High-resolution analysis of condition-specific regulatory modules in Saccharomyces cerevisiae
Source: Genome Biol. 2008 Jan 3;9(1):R2. doi: 10.1186/gb-2008-9-1-r2 (PMC2395236; doi:10.1186/gb-2008-9-1-r2)
Supplement: Additional data file 11 — Matrices describing all EPMs and RMs, including lists of synergistic pairs of regulators. [file gb-2008-9-1-r2-S11.zip › htmls/C13_EPMs_matrix/EPM_3.GO_enrichment.matrix.html]

|  |  |  |  |  |  |  |
| --- | --- | --- | --- | --- | --- | --- |
| Gcr1 | Ino4 | Abf1 | Ste12 | Ndd1 | Xbp1 | Biological Process |
|  |  |  |  |  |  | P:interphase of mitotic cell cycle |
|  |  |  |  |  |  | P:interphase |
|  |  |  |  |  |  | P:folic acid and derivative biosynthesis |
|  |  |  |  |  |  | P:endoplasmic reticulum organization and biogenesis |
|  |  |  |  |  |  | P:acetate biosynthesis |
|  |  |  |  |  |  | P:charged-tRNA modification |
|  |  |  |  |  |  | P:nucleobase catabolism |
|  |  |  |  |  |  | P:purine base catabolism |
|  |  |  |  |  |  | P:endoplasmic reticulum inheritance |
|  |  |  |  |  |  | P:folic acid metabolism |
|  |  |  |  |  |  | P:folic acid biosynthesis |
|  |  |  |  |  |  | P:pteridine and derivative metabolism |
|  |  |  |  |  |  | P:pteridine and derivative biosynthesis |
|  |  |  |  |  |  | P:anaerobic purine catabolism |
|  |  |  |  |  |  | P:nitrogenous compound catabolism |
|  |  |  |  |  |  | P:conversion of met-tRNAf to fmet-tRNA |
|  |  |  |  |  |  | P:acetate biosynthesis from carbon monoxide |
|  |  |  |  |  |  | P:secretion |
|  |  |  |  |  |  | P:protein targeting to ER |
|  |  |  |  |  |  | P:secretory pathway |
|  |  |  |  |  |  | P:physiological process |
|  |  |  |  |  |  | P:cellular process |
|  |  |  |  |  |  | P:cellular physiological process |
|  |  |  |  |  |  | P:carbohydrate biosynthesis |
|  |  |  |  |  |  | P:alcohol biosynthesis |
|  |  |  |  |  |  | P:hexose biosynthesis |
|  |  |  |  |  |  | P:monosaccharide biosynthesis |
|  |  |  |  |  |  | P:catabolism |
|  |  |  |  |  |  | P:gluconeogenesis |
|  |  |  |  |  |  | P:cellular catabolism |
|  |  |  |  |  |  | P:macromolecule catabolism |
|  |  |  |  |  |  | P:cellular macromolecule catabolism |
|  |  |  |  |  |  | P:carbohydrate metabolism |
|  |  |  |  |  |  | P:cellular carbohydrate metabolism |
|  |  |  |  |  |  | P:alcohol metabolism |
|  |  |  |  |  |  | P:generation of precursor metabolites and energy |
|  |  |  |  |  |  | P:energy derivation by oxidation of organic compounds |
|  |  |  |  |  |  | P:monosaccharide metabolism |
|  |  |  |  |  |  | P:hexose metabolism |
|  |  |  |  |  |  | P:main pathways of carbohydrate metabolism |
|  |  |  |  |  |  | P:glucose metabolism |
|  |  |  |  |  |  | P:cellular carbohydrate catabolism |
|  |  |  |  |  |  | P:carbohydrate catabolism |
|  |  |  |  |  |  | P:phosphatidylserine metabolism |
|  |  |  |  |  |  | P:polyol transport |
|  |  |  |  |  |  | P:cellular lipid metabolism |
|  |  |  |  |  |  | P:cTP biosynthesis |
|  |  |  |  |  |  | P:pyrimidine ribonucleotide biosynthesis |
|  |  |  |  |  |  | P:pyrimidine ribonucleotide metabolism |
|  |  |  |  |  |  | P:pyrimidine ribonucleoside triphosphate biosynthesis |
|  |  |  |  |  |  | P:pyrimidine ribonucleoside triphosphate metabolism |
|  |  |  |  |  |  | P:cTP metabolism |
|  |  |  |  |  |  | P:nucleobase metabolism |
|  |  |  |  |  |  | P:biosynthesis |
|  |  |  |  |  |  | P:rRNA metabolism |
|  |  |  |  |  |  | P:rRNA processing |
|  |  |  |  |  |  | P:35S primary transcript processing |
|  |  |  |  |  |  | P:ribosome biogenesis and assembly |
|  |  |  |  |  |  | P:cytoplasm organization and biogenesis |
|  |  |  |  |  |  | P:carboxylic acid biosynthesis |
|  |  |  |  |  |  | P:organic acid biosynthesis |
|  |  |  |  |  |  | P:lipid biosynthesis |
|  |  |  |  |  |  | P:fatty acid biosynthesis |
|  |  |  |  |  |  | P:ribosome biogenesis |
|  |  |  |  |  |  | P:cellular metabolism |
|  |  |  |  |  |  | P:metabolism |
|  |  |  |  |  |  | P:primary metabolism |
|  |  |  |  |  |  | P:organic acid metabolism |
|  |  |  |  |  |  | P:carboxylic acid metabolism |
|  |  |  |  |  |  | P:pyruvate metabolism |
|  |  |  |  |  |  | P:alcohol catabolism |
|  |  |  |  |  |  | P:monosaccharide catabolism |
|  |  |  |  |  |  | P:hexose catabolism |
|  |  |  |  |  |  | P:glycolysis |
|  |  |  |  |  |  | P:glucose catabolism |
|  |  |  |  |  |  | P:myo-inositol transport |
|  |  |  |  |  |  | P:phosphatidylglycerol biosynthesis |
|  |  |  |  |  |  | P:phosphatidylglycerol metabolism |
|
| Gcr1 | Ino4 | Abf1 | Ste12 | Ndd1 | Xbp1 | Molecular Function |
|  |  |  |  |  |  | F:cTP synthase activity |
|  |  |  |  |  |  | F:3-oxoacyl-[acyl-carrier protein] reductase activity |
|  |  |  |  |  |  | F:methionine-tRNA ligase activity |
|  |  |  |  |  |  | F:malic enzyme activity |
|  |  |  |  |  |  | F:3-oxoacyl-[acyl-carrier protein] synthase activity |
|  |  |  |  |  |  | F:guanylate kinase activity |
|  |  |  |  |  |  | F:fatty-acid synthase activity |
|  |  |  |  |  |  | F:nucleobase, nucleoside, nucleotide kinase activity |
|  |  |  |  |  |  | F:rRNA primary transcript binding |
|  |  |  |  |  |  | F:phosphofructokinase activity |
|  |  |  |  |  |  | F:alcohol dehydrogenase activity |
|  |  |  |  |  |  | F:phosphopyruvate hydratase activity |
|  |  |  |  |  |  | F:aldehyde-lyase activity |
|  |  |  |  |  |  | F:isomerase activity |
|  |  |  |  |  |  | F:kinase activity |
|  |  |  |  |  |  | F:6-phosphofructokinase activity |
|  |  |  |  |  |  | F:pyruvate kinase activity |
|  |  |  |  |  |  | F:transferase activity |
|  |  |  |  |  |  | F:glucose-6-phosphate isomerase activity |
|  |  |  |  |  |  | F:transferase activity, transferring phosphorus-containing groups |
|  |  |  |  |  |  | F:intramolecular oxidoreductase activity, interconverting aldoses and ketoses |
|  |  |  |  |  |  | F:intramolecular oxidoreductase activity |
|  |  |  |  |  |  | F:polyol transporter activity |
|  |  |  |  |  |  | F:nucleoside kinase activity |
|  |  |  |  |  |  | F:alcohol transporter activity |
|  |  |  |  |  |  | F:aconitate hydratase activity |
|  |  |  |  |  |  | F:catalytic activity |
|  |  |  |  |  |  | F:fructose-bisphosphate aldolase activity |
|  |  |  |  |  |  | F:enoyl-[acyl-carrier protein] reductase activity |
|  |  |  |  |  |  | F:[acyl-carrier protein] S-malonyltransferase activity |
|  |  |  |  |  |  | F:myo-inositol transporter activity |
|  |  |  |  |  |  | F:s-acetyltransferase activity |
|  |  |  |  |  |  | F:s-malonyltransferase activity |
|  |  |  |  |  |  | F:3-hydroxyacyl-[acyl-carrier protein] dehydratase activity |
|  |  |  |  |  |  | F:malonyltransferase activity |
|  |  |  |  |  |  | F:[acyl-carrier protein] S-acetyltransferase activity |
|  |  |  |  |  |  | F:enoyl-[acyl-carrier protein] reductase (NADH) activity |
|  |  |  |  |  |  | F:adenosine kinase activity |
|  |  |  |  |  |  | F:triose-phosphate isomerase activity |
|  |  |  |  |  |  | F:phosphatidate cytidylyltransferase activity |
|  |  |  |  |  |  | F:3-hydroxypalmitoyl-[acyl-carrier protein] dehydratase activity |
|  |  |  |  |  |  | F:carbon-oxygen lyase activity |
|  |  |  |  |  |  | F:lyase activity |
|  |  |  |  |  |  | F:hydro-lyase activity |
|  |  |  |  |  |  | F:phosphotransferase activity, alcohol group as acceptor |
|  |  |  |  |  |  | F:specific transcriptional repressor activity |
|  |  |  |  |  |  | F:protein-arginine N-methyltransferase activity |
|  |  |  |  |  |  | F:iMP dehydrogenase activity |
|  |  |  |  |  |  | F:arginine N-methyltransferase activity |
|  |  |  |  |  |  | F:dolichol kinase activity |
|  |  |  |  |  |  | F:microfilament motor activity |
|  |  |  |  |  |  | F:phosphatidylinositol transporter activity |
|  |  |  |  |  |  | F:signal peptidase activity |
|  |  |  |  |  |  | F:methylenetetrahydrofolate dehydrogenase activity |
|  |  |  |  |  |  | F:methenyltetrahydrofolate cyclohydrolase activity |
|  |  |  |  |  |  | F:formate-tetrahydrofolate ligase activity |
|  |  |  |  |  |  | F:aspartic-type signal peptidase activity |
|  |  |  |  |  |  | F:methylenetetrahydrofolate dehydrogenase (NADP+) activity |
|
| Gcr1 | Ino4 | Abf1 | Ste12 | Ndd1 | Xbp1 | Cellular Component |
|  |  |  |  |  |  | C:anchored to plasma membrane |
|  |  |  |  |  |  | C:signal peptidase complex |
|  |  |  |  |  |  | C:anchored to membrane |
|  |  |  |  |  |  | C:cell cortex part |
|  |  |  |  |  |  | C:external encapsulating structure |
|  |  |  |  |  |  | C:cell wall (sensu Fungi) |
|  |  |  |  |  |  | C:cell wall |
|  |  |  |  |  |  | C:ribonucleoprotein complex |
|  |  |  |  |  |  | C:box C/D snoRNP complex |
|  |  |  |  |  |  | C:fatty acid synthase complex |
|  |  |  |  |  |  | C:cytoplasm |
|  |  |  |  |  |  | C:phosphopyruvate hydratase complex |
|  |  |  |  |  |  | C:cytosol |
|  |  |  |  |  |  | C:6-phosphofructokinase complex |
|
