# Supplementary material for: High-resolution analysis of condition-specific regulatory modules in Saccharomyces cerevisiae
Source: Genome Biol. 2008 Jan 3;9(1):R2. doi: 10.1186/gb-2008-9-1-r2 (PMC2395236; doi:10.1186/gb-2008-9-1-r2)
Supplement: Additional data file 11 — Matrices describing all EPMs and RMs, including lists of synergistic pairs of regulators. [file gb-2008-9-1-r2-S11.zip › htmls/C13_EPMs_matrix/EPM_4.GO_enrichment.matrix.html]

|  |  |  |  |  |  |  |  |  |  |  |
| --- | --- | --- | --- | --- | --- | --- | --- | --- | --- | --- |
| Swi5 | Mcm1 | Ndd1 | Swi4 | Mbp1 | Swi6 | Ste12 | Dig1 | Abf1 | Gcn4 | Biological Process |
|  |  |  |  |  |  |  |  |  |  | P:cellular localization |
|  |  |  |  |  |  |  |  |  |  | P:isoleucine biosynthesis |
|  |  |  |  |  |  |  |  |  |  | P:isoleucine metabolism |
|  |  |  |  |  |  |  |  |  |  | P:cell cycle checkpoint |
|  |  |  |  |  |  |  |  |  |  | P:protein desumoylation |
|  |  |  |  |  |  |  |  |  |  | P:cytokinesis, contractile ring contraction |
|  |  |  |  |  |  |  |  |  |  | P:snRNA processing |
|  |  |  |  |  |  |  |  |  |  | P:regulation of mitosis |
|  |  |  |  |  |  |  |  |  |  | P:response to singlet oxygen |
|  |  |  |  |  |  |  |  |  |  | P:regulation of progression through cell cycle |
|  |  |  |  |  |  |  |  |  |  | P:regulation of cell cycle |
|  |  |  |  |  |  |  |  |  |  | P:cell division |
|  |  |  |  |  |  |  |  |  |  | P:cell cycle |
|  |  |  |  |  |  |  |  |  |  | P:physiological process |
|  |  |  |  |  |  |  |  |  |  | P:negative regulation of exit from mitosis |
|  |  |  |  |  |  |  |  |  |  | P:m phase of mitotic cell cycle |
|  |  |  |  |  |  |  |  |  |  | P:mitosis |
|  |  |  |  |  |  |  |  |  |  | P:cytokinesis |
|  |  |  |  |  |  |  |  |  |  | P:regulation of exit from mitosis |
|  |  |  |  |  |  |  |  |  |  | P:mitotic cell cycle |
|  |  |  |  |  |  |  |  |  |  | P:g1/S transition of mitotic cell cycle |
|  |  |  |  |  |  |  |  |  |  | P:snRNA metabolism |
|  |  |  |  |  |  |  |  |  |  | P:fatty acid elongation |
|  |  |  |  |  |  |  |  |  |  | P:trehalose biosynthesis |
|  |  |  |  |  |  |  |  |  |  | P:disaccharide biosynthesis |
|  |  |  |  |  |  |  |  |  |  | P:cellular physiological process |
|  |  |  |  |  |  |  |  |  |  | P:cellular process |
|  |  |  |  |  |  |  |  |  |  | P:organic acid transport |
|  |  |  |  |  |  |  |  |  |  | P:carboxylic acid transport |
|  |  |  |  |  |  |  |  |  |  | P:amine transport |
|  |  |  |  |  |  |  |  |  |  | P:amino acid transport |
|  |  |  |  |  |  |  |  |  |  | P:enterobactin transport |
|  |  |  |  |  |  |  |  |  |  | P:ferric-enterobactin transport |
|  |  |  |  |  |  |  |  |  |  | P:ferric iron transport |
|  |  |  |  |  |  |  |  |  |  | P:second-messenger-mediated signaling |
|  |  |  |  |  |  |  |  |  |  | P:plasma membrane fusion during cytogamy |
|  |  |  |  |  |  |  |  |  |  | P:phosphoinositide-mediated signaling |
|  |  |  |  |  |  |  |  |  |  | P:inositol lipid-mediated signaling |
|  |  |  |  |  |  |  |  |  |  | P:lipid modification |
|  |  |  |  |  |  |  |  |  |  | P:reproduction |
|  |  |  |  |  |  |  |  |  |  | P:interaction between organisms |
|  |  |  |  |  |  |  |  |  |  | P:reproductive cellular physiological process |
|  |  |  |  |  |  |  |  |  |  | P:reproductive physiological process |
|  |  |  |  |  |  |  |  |  |  | P:conjugation with cellular fusion |
|  |  |  |  |  |  |  |  |  |  | P:sexual reproduction |
|  |  |  |  |  |  |  |  |  |  | P:conjugation |
|  |  |  |  |  |  |  |  |  |  | P:cell communication |
|  |  |  |  |  |  |  |  |  |  | P:intracellular signaling cascade |
|  |  |  |  |  |  |  |  |  |  | P:signal transduction |
|  |  |  |  |  |  |  |  |  |  | P:macromolecule catabolism |
|  |  |  |  |  |  |  |  |  |  | P:protein metabolism |
|  |  |  |  |  |  |  |  |  |  | P:glutamine family amino acid biosynthesis |
|  |  |  |  |  |  |  |  |  |  | P:cellular macromolecule catabolism |
|  |  |  |  |  |  |  |  |  |  | P:primary metabolism |
|  |  |  |  |  |  |  |  |  |  | P:protein catabolism |
|  |  |  |  |  |  |  |  |  |  | P:biopolymer metabolism |
|  |  |  |  |  |  |  |  |  |  | P:catabolism |
|  |  |  |  |  |  |  |  |  |  | P:response to biotic stimulus |
|  |  |  |  |  |  |  |  |  |  | P:cellular macromolecule metabolism |
|  |  |  |  |  |  |  |  |  |  | P:biopolymer catabolism |
|  |  |  |  |  |  |  |  |  |  | P:cellular catabolism |
|  |  |  |  |  |  |  |  |  |  | P:cellular protein catabolism |
|  |  |  |  |  |  |  |  |  |  | P:proteolysis during cellular protein catabolism |
|  |  |  |  |  |  |  |  |  |  | P:modification-dependent macromolecule catabolism |
|  |  |  |  |  |  |  |  |  |  | P:ubiquitin-dependent protein catabolism |
|  |  |  |  |  |  |  |  |  |  | P:modification-dependent protein catabolism |
|  |  |  |  |  |  |  |  |  |  | P:cellular protein metabolism |
|  |  |  |  |  |  |  |  |  |  | P:dNA integrity checkpoint |
|  |  |  |  |  |  |  |  |  |  | P:protein modification |
|  |  |  |  |  |  |  |  |  |  | P:metabolism |
|  |  |  |  |  |  |  |  |  |  | P:macromolecule metabolism |
|  |  |  |  |  |  |  |  |  |  | P:cellular metabolism |
|  |  |  |  |  |  |  |  |  |  | P:biopolymer modification |
|  |  |  |  |  |  |  |  |  |  | P:ubiquitin cycle |
|  |  |  |  |  |  |  |  |  |  | P:proteolysis |
|  |  |  |  |  |  |  |  |  |  | P:extrachromosomal circular DNA accumulation during cell aging |
|  |  |  |  |  |  |  |  |  |  | P:extrachromosomal circular DNA accumulation during replicative cell aging |
|  |  |  |  |  |  |  |  |  |  | P:extrachromosomal rDNA circle accumulation during replicative cell aging |
|  |  |  |  |  |  |  |  |  |  | P:citrulline metabolism |
|  |  |  |  |  |  |  |  |  |  | P:argininosuccinate metabolism |
|  |  |  |  |  |  |  |  |  |  | P:energy reserve metabolism |
|  |  |  |  |  |  |  |  |  |  | P:interphase of mitotic cell cycle |
|  |  |  |  |  |  |  |  |  |  | P:interphase |
|
| Swi5 | Mcm1 | Ndd1 | Swi4 | Mbp1 | Swi6 | Ste12 | Dig1 | Abf1 | Gcn4 | Molecular Function |
|  |  |  |  |  |  |  |  |  |  | F:serine-type endopeptidase activity |
|  |  |  |  |  |  |  |  |  |  | F:cytoskeletal protein binding |
|  |  |  |  |  |  |  |  |  |  | F:actin lateral binding |
|  |  |  |  |  |  |  |  |  |  | F:actin binding |
|  |  |  |  |  |  |  |  |  |  | F:enzyme regulator activity |
|  |  |  |  |  |  |  |  |  |  | F:chitin synthase activity |
|  |  |  |  |  |  |  |  |  |  | F:alpha,alpha-trehalose-phosphate synthase (UDP-forming) activity |
|  |  |  |  |  |  |  |  |  |  | F:uDP-glycosyltransferase activity |
|  |  |  |  |  |  |  |  |  |  | F:trehalose-phosphatase activity |
|  |  |  |  |  |  |  |  |  |  | F:cellulase activity |
|  |  |  |  |  |  |  |  |  |  | F:ketoreductase activity |
|  |  |  |  |  |  |  |  |  |  | F:glycogen (starch) synthase activity |
|  |  |  |  |  |  |  |  |  |  | F:transferase activity, transferring glycosyl groups |
|  |  |  |  |  |  |  |  |  |  | F:transferase activity, transferring hexosyl groups |
|  |  |  |  |  |  |  |  |  |  | F:uDP-glucosyltransferase activity |
|  |  |  |  |  |  |  |  |  |  | F:glucosyltransferase activity |
|  |  |  |  |  |  |  |  |  |  | F:l-proline transporter activity |
|  |  |  |  |  |  |  |  |  |  | F:l-proline permease activity |
|  |  |  |  |  |  |  |  |  |  | F:siderophore transporter activity |
|  |  |  |  |  |  |  |  |  |  | F:siderophore-iron transporter activity |
|  |  |  |  |  |  |  |  |  |  | F:dihydrokaempferol 4-reductase activity |
|  |  |  |  |  |  |  |  |  |  | F:organic acid transporter activity |
|  |  |  |  |  |  |  |  |  |  | F:carboxylic acid transporter activity |
|  |  |  |  |  |  |  |  |  |  | F:amine transporter activity |
|  |  |  |  |  |  |  |  |  |  | F:porter activity |
|  |  |  |  |  |  |  |  |  |  | F:electrochemical potential-driven transporter activity |
|  |  |  |  |  |  |  |  |  |  | F:ferric-enterobactin transporter activity |
|  |  |  |  |  |  |  |  |  |  | F:amino acid transporter activity |
|  |  |  |  |  |  |  |  |  |  | F:polyamine transporter activity |
|  |  |  |  |  |  |  |  |  |  | F:amino acid permease activity |
|  |  |  |  |  |  |  |  |  |  | F:amino acid-polyamine transporter activity |
|  |  |  |  |  |  |  |  |  |  | F:glutathione disulfide oxidoreductase activity |
|  |  |  |  |  |  |  |  |  |  | F:peptide disulfide oxidoreductase activity |
|  |  |  |  |  |  |  |  |  |  | F:leucyl aminopeptidase activity |
|  |  |  |  |  |  |  |  |  |  | F:argininosuccinate synthase activity |
|  |  |  |  |  |  |  |  |  |  | F:glutathione-disulfide reductase activity |
|  |  |  |  |  |  |  |  |  |  | F:adenyl-nucleotide exchange factor activity |
|  |  |  |  |  |  |  |  |  |  | F:pantothenate kinase activity |
|  |  |  |  |  |  |  |  |  |  | F:gTPase activator activity |
|  |  |  |  |  |  |  |  |  |  | F:polynucleotide 5'-phosphatase activity |
|  |  |  |  |  |  |  |  |  |  | F:isocitrate dehydrogenase (NAD+) activity |
|  |  |  |  |  |  |  |  |  |  | F:5'-3' exoribonuclease activity |
|  |  |  |  |  |  |  |  |  |  | F:cysteine-type peptidase activity |
|  |  |  |  |  |  |  |  |  |  | F:ubiquitin activating enzyme activity |
|  |  |  |  |  |  |  |  |  |  | F:catalytic activity |
|  |  |  |  |  |  |  |  |  |  | F:small conjugating protein-specific protease activity |
|  |  |  |  |  |  |  |  |  |  | F:peptidase activity |
|  |  |  |  |  |  |  |  |  |  | F:metallopeptidase activity |
|  |  |  |  |  |  |  |  |  |  | F:saccharolysin activity |
|  |  |  |  |  |  |  |  |  |  | F:sUMO-specific protease activity |
|
| Swi5 | Mcm1 | Ndd1 | Swi4 | Mbp1 | Swi6 | Ste12 | Dig1 | Abf1 | Gcn4 | Cellular Component |
|  |  |  |  |  |  |  |  |  |  | C:bud |
|  |  |  |  |  |  |  |  |  |  | C:alpha,alpha-trehalose-phosphate synthase complex (UDP-forming) |
|  |  |  |  |  |  |  |  |  |  | C:m-AAA complex |
|  |  |  |  |  |  |  |  |  |  | C:transcription factor TFIIIB complex |
|  |  |  |  |  |  |  |  |  |  | C:cell projection |
|  |  |  |  |  |  |  |  |  |  | C:mating projection |
|  |  |  |  |  |  |  |  |  |  | C:actin cortical patch |
|  |  |  |  |  |  |  |  |  |  | C:cell projection part |
|  |  |  |  |  |  |  |  |  |  | C:site of polarized growth |
|  |  |  |  |  |  |  |  |  |  | C:mating projection tip |
|  |  |  |  |  |  |  |  |  |  | C:cell part |
|  |  |  |  |  |  |  |  |  |  | C:cell |
|  |  |  |  |  |  |  |  |  |  | C:eR-Golgi intermediate compartment |
|  |  |  |  |  |  |  |  |  |  | C:chitosome |
|  |  |  |  |  |  |  |  |  |  | C:golgi trans face |
|  |  |  |  |  |  |  |  |  |  | C:cytoplasmic part |
|  |  |  |  |  |  |  |  |  |  | C:proteasome regulatory particle (sensu Eukaryota) |
|  |  |  |  |  |  |  |  |  |  | C:proteasome regulatory particle, lid subcomplex (sensu Eukaryota) |
|  |  |  |  |  |  |  |  |  |  | C:proteasome complex (sensu Eukaryota) |
|
