# Supplementary material for: High-resolution analysis of condition-specific regulatory modules in Saccharomyces cerevisiae
Source: Genome Biol. 2008 Jan 3;9(1):R2. doi: 10.1186/gb-2008-9-1-r2 (PMC2395236; doi:10.1186/gb-2008-9-1-r2)
Supplement: Additional data file 11 — Matrices describing all EPMs and RMs, including lists of synergistic pairs of regulators. [file gb-2008-9-1-r2-S11.zip › htmls/C13_EPMs_matrix/EPM_4.RM.matrix.html]

Regulators vs. RM target gene list

|  |  |  |  |  |  |  |  |  |  |  |
| --- | --- | --- | --- | --- | --- | --- | --- | --- | --- | --- |
|  | Swi5 | Mcm1 | Ndd1 | Swi4 | Mbp1 | Swi6 | Ste12 | Dig1 | Abf1 | Gcn4 |
| RM\_1 |  |  |  |  |  |  |  |  |  |  |
| RM\_2 |  |  |  |  |  |  |  |  |  |  |
| RM\_3 |  |  |  |  |  |  |  |  |  |  |
| RM\_4 |  |  |  |  |  |  |  |  |  |  |
| RM\_5 |  |  |  |  |  |  |  |  |  |  |
| RM\_6 |  |  |  |  |  |  |  |  |  |  |

Synergistic Pair of Regulators

1. Mcm1\*Swi4

2. Ste12\*Swi5

3. Mcm1\*Ndd1

4. Mbp1\*Mcm1

5. Mcm1\*Swi6

Matrix of enriched GO

EPM matrix
