# Supplementary material for: High-resolution analysis of condition-specific regulatory modules in Saccharomyces cerevisiae
Source: Genome Biol. 2008 Jan 3;9(1):R2. doi: 10.1186/gb-2008-9-1-r2 (PMC2395236; doi:10.1186/gb-2008-9-1-r2)
Supplement: Additional data file 11 — Matrices describing all EPMs and RMs, including lists of synergistic pairs of regulators. [file gb-2008-9-1-r2-S11.zip › htmls/C13_EPMs_matrix/EPM_5.GO_enrichment.matrix.html]

|  |  |  |  |  |  |
| --- | --- | --- | --- | --- | --- |
| Msn4 | Msn2 | Skn7 | Swi5 | Ume1 | Biological Process |
|  |  |  |  |  | P:protein myristoylation |
|  |  |  |  |  | P:n-terminal protein myristoylation |
|  |  |  |  |  | P:protein amino acid myristoylation |
|  |  |  |  |  | P:cellular polysaccharide metabolism |
|  |  |  |  |  | P:polysaccharide metabolism |
|  |  |  |  |  | P:carbohydrate metabolism |
|  |  |  |  |  | P:cellular carbohydrate metabolism |
|  |  |  |  |  | P:glucan metabolism |
|  |  |  |  |  | P:glycogen metabolism |
|  |  |  |  |  | P:energy reserve metabolism |
|
| Msn4 | Msn2 | Skn7 | Swi5 | Ume1 | Molecular Function |
|  |  |  |  |  | F:protein kinase inhibitor activity |
|  |  |  |  |  | F:kinase inhibitor activity |
|  |  |  |  |  | F:transferase activity, transferring glycosyl groups |
|  |  |  |  |  | F:long-chain-fatty-acid-CoA ligase activity |
|  |  |  |  |  | F:fatty-acid ligase activity |
|  |  |  |  |  | F:transferase activity, transferring hexosyl groups |
|  |  |  |  |  | F:glycerol-3-phosphate O-acyltransferase activity |
|  |  |  |  |  | F:1,4-alpha-glucan branching enzyme activity |
|  |  |  |  |  | F:glycogen debranching enzyme activity |
|  |  |  |  |  | F:amylo-alpha-1,6-glucosidase activity |
|  |  |  |  |  | F:4-alpha-glucanotransferase activity |
|
| Msn4 | Msn2 | Skn7 | Swi5 | Ume1 | Cellular Component |
|  |  |  |  |  | C:eisosome |
|  |  |  |  |  | C:cytoplasm |
|  |  |  |  |  | C:outer membrane |
|  |  |  |  |  | C:organelle outer membrane |
|  |  |  |  |  | C:mitochondrial outer membrane |
|
