# Supplementary material for: High-resolution analysis of condition-specific regulatory modules in Saccharomyces cerevisiae
Source: Genome Biol. 2008 Jan 3;9(1):R2. doi: 10.1186/gb-2008-9-1-r2 (PMC2395236; doi:10.1186/gb-2008-9-1-r2)
Supplement: Additional data file 11 — Matrices describing all EPMs and RMs, including lists of synergistic pairs of regulators. [file gb-2008-9-1-r2-S11.zip › htmls/C13_EPMs_matrix/EPM_6.RM.matrix.html]

Regulators vs. RM target gene list

|  |  |  |  |  |  |  |  |  |  |  |  |  |  |  |  |  |  |  |  |  |  |  |  |
| --- | --- | --- | --- | --- | --- | --- | --- | --- | --- | --- | --- | --- | --- | --- | --- | --- | --- | --- | --- | --- | --- | --- | --- |
|  | Ino4 | Gcn4 | Gcr1 | Stb5 | Abf1 | Hsf1 | Tec1 | Stb1 | Mcm1 | Ndd1 | Ste12 | Dig1 | Rlm1 | Sig1 | Sum1 | Xbp1 | Mbp1 | Swi4 | Swi6 | Swi5 | Ace2 | Fkh2 | Fkh1 |
| RM\_1 |  |  |  |  |  |  |  |  |  |  |  |  |  |  |  |  |  |  |  |  |  |  |  |
| RM\_2 |  |  |  |  |  |  |  |  |  |  |  |  |  |  |  |  |  |  |  |  |  |  |  |
| RM\_3 |  |  |  |  |  |  |  |  |  |  |  |  |  |  |  |  |  |  |  |  |  |  |  |
| RM\_4 |  |  |  |  |  |  |  |  |  |  |  |  |  |  |  |  |  |  |  |  |  |  |  |
| RM\_5 |  |  |  |  |  |  |  |  |  |  |  |  |  |  |  |  |  |  |  |  |  |  |  |
| RM\_6 |  |  |  |  |  |  |  |  |  |  |  |  |  |  |  |  |  |  |  |  |  |  |  |
| RM\_7 |  |  |  |  |  |  |  |  |  |  |  |  |  |  |  |  |  |  |  |  |  |  |  |
| RM\_8 |  |  |  |  |  |  |  |  |  |  |  |  |  |  |  |  |  |  |  |  |  |  |  |
| RM\_9 |  |  |  |  |  |  |  |  |  |  |  |  |  |  |  |  |  |  |  |  |  |  |  |
| RM\_10 |  |  |  |  |  |  |  |  |  |  |  |  |  |  |  |  |  |  |  |  |  |  |  |
| RM\_11 |  |  |  |  |  |  |  |  |  |  |  |  |  |  |  |  |  |  |  |  |  |  |  |
| RM\_12 |  |  |  |  |  |  |  |  |  |  |  |  |  |  |  |  |  |  |  |  |  |  |  |
| RM\_13 |  |  |  |  |  |  |  |  |  |  |  |  |  |  |  |  |  |  |  |  |  |  |  |

Synergistic Pair of Regulators

1. Mcm1\*Ndd1

2. Fkh2\*Mcm1

3. Mcm1\*Ste12

4. Fkh2\*Ndd1

5. Rlm1\*Swi6

6. Ste12\*Swi4

7. Fkh1\*Ndd1

8. Ste12\*Swi5

9. Dig1\*Swi4

10. Ndd1\*Ste12

11. Rlm1\*Swi4

12. Dig1\*Swi5

13. Gcn4\*Sum1

14. Stb1\*Swi4

15. Stb1\*Swi6

16. Gcr1\*Stb5

17. Ndd1\*Swi4

18. Mbp1\*Swi4

19. Mbp1\*Swi6

20. Swi4\*Swi6

21. Dig1\*Mcm1

22. Mcm1\*Swi6

23. Hsf1\*Stb5

24. Fkh1\*Fkh2

25. Mcm1\*Sig1

26. Mcm1\*Swi4

27. Gcr1\*Hsf1

28. Mbp1\*Ndd1

29. Ndd1\*Swi6

30. Mbp1\*Stb1

31. Mbp1\*Xbp1

32. Dig1\*Ndd1

33. Swi6\*Xbp1

34. Swi4\*Xbp1

35. Ndd1\*Stb1

36. Gcr1\*Ste12

37. Mcm1\*Stb1

Matrix of enriched GO

EPM matrix
