# Supplementary material for: High-resolution analysis of condition-specific regulatory modules in Saccharomyces cerevisiae
Source: Genome Biol. 2008 Jan 3;9(1):R2. doi: 10.1186/gb-2008-9-1-r2 (PMC2395236; doi:10.1186/gb-2008-9-1-r2)
Supplement: Additional data file 11 — Matrices describing all EPMs and RMs, including lists of synergistic pairs of regulators. [file gb-2008-9-1-r2-S11.zip › htmls/C13_EPMs_matrix/EPM_7.GO_enrichment.matrix.html]

|  |  |
| --- | --- |
| Rpn4 | Biological Process |
|  | P:regulation of actin filament length |
|  | P:regulation of actin polymerization and/or depolymerization |
|  | P:aging |
|  | P:cell aging |
|  | P:barbed-end actin filament capping |
|  | P:actin filament capping |
|  | P:negative regulation of proteolysis |
|  | P:regulation of actin filament depolymerization |
|  | P:negative regulation of actin filament depolymerization |
|  | P:metabolism |
|  | P:cellular metabolism |
|  | P:primary metabolism |
|  | P:biopolymer metabolism |
|  | P:negative regulation of protein metabolism |
|  | P:macromolecule metabolism |
|  | P:catabolism |
|  | P:biopolymer modification |
|  | P:cellular catabolism |
|  | P:protein modification |
|  | P:macromolecule catabolism |
|  | P:cellular macromolecule catabolism |
|  | P:protein metabolism |
|  | P:biopolymer catabolism |
|  | P:cellular macromolecule metabolism |
|  | P:cellular protein metabolism |
|  | P:ubiquitin cycle |
|  | P:protein catabolism |
|  | P:cellular protein catabolism |
|  | P:proteolysis during cellular protein catabolism |
|  | P:modification-dependent macromolecule catabolism |
|  | P:ubiquitin-dependent protein catabolism |
|  | P:proteolysis |
|  | P:modification-dependent protein catabolism |
|
| Rpn4 | Molecular Function |
|  | F:rab GTPase binding |
|  | F:cytoskeletal protein binding |
|  | F:mitochondrial inner membrane peptidase activity |
|  | F:x-Pro aminopeptidase activity |
|  | F:n-acetylglucosaminyldiphosphodolichol N-acetylglucosaminyltransferase activity |
|  | F:catalytic activity |
|  | F:actin binding |
|  | F:actin filament binding |
|  | F:hydrolase activity |
|  | F:endopeptidase activity |
|  | F:peptidase activity |
|
| Rpn4 | Cellular Component |
|  | C:mitochondrial inner membrane peptidase complex |
|  | C:actin cortical patch |
|  | C:extrinsic to endoplasmic reticulum membrane |
|  | C:f-actin capping protein complex |
|  | C:uDP-N-acetylglucosamine transferase complex |
|  | C:cell cortex |
|  | C:cell cortex part |
|  | C:actin cytoskeleton |
|  | C:protein complex |
|  | C:proteasome regulatory particle, lid subcomplex (sensu Eukaryota) |
|  | C:proteasome regulatory particle (sensu Eukaryota) |
|  | C:proteasome core complex, beta-subunit complex (sensu Eukaryota) |
|  | C:proteasome complex (sensu Eukaryota) |
|  | C:proteasome core complex (sensu Eukaryota) |
|
