# Supplementary material for: High-resolution analysis of condition-specific regulatory modules in Saccharomyces cerevisiae
Source: Genome Biol. 2008 Jan 3;9(1):R2. doi: 10.1186/gb-2008-9-1-r2 (PMC2395236; doi:10.1186/gb-2008-9-1-r2)
Supplement: Additional data file 11 — Matrices describing all EPMs and RMs, including lists of synergistic pairs of regulators. [file gb-2008-9-1-r2-S11.zip › htmls/C13_EPMs_matrix/EPM_8.GO_enrichment.matrix.html]

|  |  |  |  |  |  |  |  |  |  |  |  |  |  |  |  |  |  |  |  |  |  |  |  |  |  |
| --- | --- | --- | --- | --- | --- | --- | --- | --- | --- | --- | --- | --- | --- | --- | --- | --- | --- | --- | --- | --- | --- | --- | --- | --- | --- |
| Mal33 | Yap6 | Hap1 | Hap4 | Xbp1 | Dat1 | Uga3 | Rds1 | Swi6 | Gzf3 | Tec1 | Abf1 | Dal82 | Gcn4 | Gat1 | Swi4 | Pdr3 | Stp1 | Msn4 | Ume1 | Ndd1 | Pdr1 | Mcm1 | Yap5 | Gat3 | Biological Process |
|  |  |  |  |  |  |  |  |  |  |  |  |  |  |  |  |  |  |  |  |  |  |  |  |  | P:siderophore-iron transport |
|  |  |  |  |  |  |  |  |  |  |  |  |  |  |  |  |  |  |  |  |  |  |  |  |  | P:flocculation via cell wall protein-carbohydrate interaction |
|  |  |  |  |  |  |  |  |  |  |  |  |  |  |  |  |  |  |  |  |  |  |  |  |  | P:siderophore transport |
|  |  |  |  |  |  |  |  |  |  |  |  |  |  |  |  |  |  |  |  |  |  |  |  |  | P:calcium-dependent cell-cell adhesion |
|  |  |  |  |  |  |  |  |  |  |  |  |  |  |  |  |  |  |  |  |  |  |  |  |  | P:flocculation |
|  |  |  |  |  |  |  |  |  |  |  |  |  |  |  |  |  |  |  |  |  |  |  |  |  | P:biological process unknown |
|  |  |  |  |  |  |  |  |  |  |  |  |  |  |  |  |  |  |  |  |  |  |  |  |  | P:dNA recombination |
|  |  |  |  |  |  |  |  |  |  |  |  |  |  |  |  |  |  |  |  |  |  |  |  |  | P:telomere maintenance via recombination |
|  |  |  |  |  |  |  |  |  |  |  |  |  |  |  |  |  |  |  |  |  |  |  |  |  | P:mitotic recombination |
|  |  |  |  |  |  |  |  |  |  |  |  |  |  |  |  |  |  |  |  |  |  |  |  |  | P:telomere maintenance |
|  |  |  |  |  |  |  |  |  |  |  |  |  |  |  |  |  |  |  |  |  |  |  |  |  | P:telomere organization and biogenesis |
|  |  |  |  |  |  |  |  |  |  |  |  |  |  |  |  |  |  |  |  |  |  |  |  |  | P:chromosome organization and biogenesis |
|  |  |  |  |  |  |  |  |  |  |  |  |  |  |  |  |  |  |  |  |  |  |  |  |  | P:chromosome organization and biogenesis (sensu Eukaryota) |
|  |  |  |  |  |  |  |  |  |  |  |  |  |  |  |  |  |  |  |  |  |  |  |  |  | P:dNA metabolism |
|
| Mal33 | Yap6 | Hap1 | Hap4 | Xbp1 | Dat1 | Uga3 | Rds1 | Swi6 | Gzf3 | Tec1 | Abf1 | Dal82 | Gcn4 | Gat1 | Swi4 | Pdr3 | Stp1 | Msn4 | Ume1 | Ndd1 | Pdr1 | Mcm1 | Yap5 | Gat3 | Molecular Function |
|  |  |  |  |  |  |  |  |  |  |  |  |  |  |  |  |  |  |  |  |  |  |  |  |  | F:carbohydrate binding |
|  |  |  |  |  |  |  |  |  |  |  |  |  |  |  |  |  |  |  |  |  |  |  |  |  | F:mannose binding |
|  |  |  |  |  |  |  |  |  |  |  |  |  |  |  |  |  |  |  |  |  |  |  |  |  | F:ferric-chelate reductase activity |
|  |  |  |  |  |  |  |  |  |  |  |  |  |  |  |  |  |  |  |  |  |  |  |  |  | F:sugar binding |
|  |  |  |  |  |  |  |  |  |  |  |  |  |  |  |  |  |  |  |  |  |  |  |  |  | F:monosaccharide binding |
|  |  |  |  |  |  |  |  |  |  |  |  |  |  |  |  |  |  |  |  |  |  |  |  |  | F:oxidoreductase activity, oxidizing metal ions, NAD or NADP as acceptor |
|  |  |  |  |  |  |  |  |  |  |  |  |  |  |  |  |  |  |  |  |  |  |  |  |  | F:catalytic activity |
|  |  |  |  |  |  |  |  |  |  |  |  |  |  |  |  |  |  |  |  |  |  |  |  |  | F:helicase activity |
|  |  |  |  |  |  |  |  |  |  |  |  |  |  |  |  |  |  |  |  |  |  |  |  |  | F:dNA helicase activity |
|
| Mal33 | Yap6 | Hap1 | Hap4 | Xbp1 | Dat1 | Uga3 | Rds1 | Swi6 | Gzf3 | Tec1 | Abf1 | Dal82 | Gcn4 | Gat1 | Swi4 | Pdr3 | Stp1 | Msn4 | Ume1 | Ndd1 | Pdr1 | Mcm1 | Yap5 | Gat3 | Cellular Component |
|  |  |  |  |  |  |  |  |  |  |  |  |  |  |  |  |  |  |  |  |  |  |  |  |  | C:cellular component unknown |
|
