# Supplementary material for: High-resolution analysis of condition-specific regulatory modules in Saccharomyces cerevisiae
Source: Genome Biol. 2008 Jan 3;9(1):R2. doi: 10.1186/gb-2008-9-1-r2 (PMC2395236; doi:10.1186/gb-2008-9-1-r2)
Supplement: Additional data file 11 — Matrices describing all EPMs and RMs, including lists of synergistic pairs of regulators. [file gb-2008-9-1-r2-S11.zip › htmls/C13_EPMs_matrix/EPM_8.RM.matrix.html]

Regulators vs. RM target gene list

|  |  |  |  |  |  |  |  |  |  |  |  |  |  |  |  |  |  |  |  |  |  |  |  |  |  |
| --- | --- | --- | --- | --- | --- | --- | --- | --- | --- | --- | --- | --- | --- | --- | --- | --- | --- | --- | --- | --- | --- | --- | --- | --- | --- |
|  | Mal33 | Yap6 | Hap1 | Hap4 | Xbp1 | Dat1 | Uga3 | Rds1 | Swi6 | Gzf3 | Tec1 | Abf1 | Dal82 | Gcn4 | Gat1 | Swi4 | Pdr3 | Stp1 | Msn4 | Ume1 | Ndd1 | Pdr1 | Mcm1 | Yap5 | Gat3 |
| RM\_1 |  |  |  |  |  |  |  |  |  |  |  |  |  |  |  |  |  |  |  |  |  |  |  |  |  |
| RM\_2 |  |  |  |  |  |  |  |  |  |  |  |  |  |  |  |  |  |  |  |  |  |  |  |  |  |
| RM\_3 |  |  |  |  |  |  |  |  |  |  |  |  |  |  |  |  |  |  |  |  |  |  |  |  |  |
| RM\_4 |  |  |  |  |  |  |  |  |  |  |  |  |  |  |  |  |  |  |  |  |  |  |  |  |  |
| RM\_5 |  |  |  |  |  |  |  |  |  |  |  |  |  |  |  |  |  |  |  |  |  |  |  |  |  |

Synergistic Pair of Regulators

1. Msn4\*Yap5

2. Gat3\*Msn4

3. Msn4\*Pdr1

4. Dat1\*Yap5

5. Gzf3\*Uga3

6. Hap1\*Yap5

7. Pdr1\*Yap6

8. Gzf3\*Pdr1

9. Gzf3\*Hap1

10. Hap4\*Pdr1

11. Pdr1\*Uga3

12. Gzf3\*Xbp1

13. Gzf3\*Msn4

14. Uga3\*Xbp1

15. Hap1\*Uga3

16. Hap1\*Xbp1

17. Uga3\*Yap6

18. Hap4\*Xbp1

19. Hap1\*Pdr1

20. Msn4\*Xbp1

21. Hap4\*Msn4

22. Pdr1\*Xbp1

23. Msn4\*Uga3

24. Hap1\*Msn4

25. Hap1\*Yap6

26. Hap4\*Yap6

27. Msn4\*Yap6

28. Hap1\*Hap4

29. Mcm1\*Pdr1

30. Gcn4\*Mcm1

31. Abf1\*Mcm1

32. Ndd1\*Pdr1

33. Abf1\*Pdr1

34. Abf1\*Dal82

35. Gcn4\*Pdr1

36. Abf1\*Gcn4

37. Gat1\*Pdr1

38. Dal82\*Pdr1

39. Mcm1\*Ume1

40. Pdr1\*Ume1

41. Dal82\*Gcn4

42. Mcm1\*Swi6

43. Gat1\*Mcm1

44. Abf1\*Gat1

45. Dal82\*Mcm1

46. Pdr1\*Swi4

47. Gat1\*Gcn4

48. Pdr1\*Swi6

49. Dal82\*Tec1

50. Gzf3\*Mcm1

51. Gcn4\*Ume1

52. Gcn4\*Tec1

53. Gcn4\*Gzf3

54. Gat1\*Ume1

55. Mcm1\*Swi4

56. Dal82\*Swi4

57. Dal82\*Swi6

58. Gat1\*Tec1

59. Mcm1\*Stp1

60. Dal82\*Ume1

61. Gat1\*Swi6

62. Dal82\*Pdr3

63. Dal82\*Stp1

64. Gcn4\*Ndd1

65. Abf1\*Tec1

66. Pdr1\*Stp1

67. Pdr1\*Tec1

68. Gat1\*Stp1

69. Gcn4\*Swi4

70. Dal82\*Gat1

71. Gcn4\*Stp1

72. Dal82\*Ndd1

73. Gat1\*Ndd1

74. Mcm1\*Tec1

75. Mcm1\*Ndd1

76. Rds1\*Swi6

77. Gzf3\*Tec1

78. Abf1\*Ndd1

79. Abf1\*Swi6

80. Ndd1\*Swi6

81. Pdr3\*Tec1

82. Abf1\*Swi4

83. Stp1\*Tec1

84. Ndd1\*Ume1

85. Abf1\*Ume1

86. Tec1\*Ume1

Matrix of enriched GO

EPM matrix
