# Supplementary material for: High-resolution analysis of condition-specific regulatory modules in Saccharomyces cerevisiae
Source: Genome Biol. 2008 Jan 3;9(1):R2. doi: 10.1186/gb-2008-9-1-r2 (PMC2395236; doi:10.1186/gb-2008-9-1-r2)
Supplement: Additional data file 11 — Matrices describing all EPMs and RMs, including lists of synergistic pairs of regulators. [file gb-2008-9-1-r2-S11.zip › htmls/C13_EPMs_matrix/EPM_9.GO_enrichment.matrix.html]

|  |  |  |  |  |  |  |  |  |  |  |  |  |  |  |  |  |  |  |  |  |  |  |  |  |  |  |  |  |  |  |
| --- | --- | --- | --- | --- | --- | --- | --- | --- | --- | --- | --- | --- | --- | --- | --- | --- | --- | --- | --- | --- | --- | --- | --- | --- | --- | --- | --- | --- | --- | --- |
| Ste12 | Dig1 | Pho4 | Hap1 | Rph1 | Phd1 | Fkh2 | Gal4 | Gat3 | Stp1 | Yap5 | Tec1 | Swi6 | Swi4 | Mat1mc | Pdr3 | Dal82 | Pdr1 | Rcs1 | Stb4 | Ume1 | Abf1 | Gat1 | Mcm1 | Fkh1 | Ndd1 | Gcn4 | Gzf3 | Rap1 | Rds1 | Biological Process |
|  |  |  |  |  |  |  |  |  |  |  |  |  |  |  |  |  |  |  |  |  |  |  |  |  |  |  |  |  |  | P:biological process unknown |
|  |  |  |  |  |  |  |  |  |  |  |  |  |  |  |  |  |  |  |  |  |  |  |  |  |  |  |  |  |  | P:flocculation via cell wall protein-carbohydrate interaction |
|  |  |  |  |  |  |  |  |  |  |  |  |  |  |  |  |  |  |  |  |  |  |  |  |  |  |  |  |  |  | P:calcium-dependent cell-cell adhesion |
|  |  |  |  |  |  |  |  |  |  |  |  |  |  |  |  |  |  |  |  |  |  |  |  |  |  |  |  |  |  | P:flocculation |
|
| Ste12 | Dig1 | Pho4 | Hap1 | Rph1 | Phd1 | Fkh2 | Gal4 | Gat3 | Stp1 | Yap5 | Tec1 | Swi6 | Swi4 | Mat1mc | Pdr3 | Dal82 | Pdr1 | Rcs1 | Stb4 | Ume1 | Abf1 | Gat1 | Mcm1 | Fkh1 | Ndd1 | Gcn4 | Gzf3 | Rap1 | Rds1 | Molecular Function |
|  |  |  |  |  |  |  |  |  |  |  |  |  |  |  |  |  |  |  |  |  |  |  |  |  |  |  |  |  |  | F:molecular function unknown |
|  |  |  |  |  |  |  |  |  |  |  |  |  |  |  |  |  |  |  |  |  |  |  |  |  |  |  |  |  |  | F:mannose binding |
|  |  |  |  |  |  |  |  |  |  |  |  |  |  |  |  |  |  |  |  |  |  |  |  |  |  |  |  |  |  | F:carbohydrate binding |
|  |  |  |  |  |  |  |  |  |  |  |  |  |  |  |  |  |  |  |  |  |  |  |  |  |  |  |  |  |  | F:monosaccharide binding |
|  |  |  |  |  |  |  |  |  |  |  |  |  |  |  |  |  |  |  |  |  |  |  |  |  |  |  |  |  |  | F:sugar binding |
|
| Ste12 | Dig1 | Pho4 | Hap1 | Rph1 | Phd1 | Fkh2 | Gal4 | Gat3 | Stp1 | Yap5 | Tec1 | Swi6 | Swi4 | Mat1mc | Pdr3 | Dal82 | Pdr1 | Rcs1 | Stb4 | Ume1 | Abf1 | Gat1 | Mcm1 | Fkh1 | Ndd1 | Gcn4 | Gzf3 | Rap1 | Rds1 | Cellular Component |
|  |  |  |  |  |  |  |  |  |  |  |  |  |  |  |  |  |  |  |  |  |  |  |  |  |  |  |  |  |  | C:cellular component unknown |
|
