# Supplementary material for: High-resolution analysis of condition-specific regulatory modules in Saccharomyces cerevisiae
Source: Genome Biol. 2008 Jan 3;9(1):R2. doi: 10.1186/gb-2008-9-1-r2 (PMC2395236; doi:10.1186/gb-2008-9-1-r2)
Supplement: Additional data file 11 — Matrices describing all EPMs and RMs, including lists of synergistic pairs of regulators. [file gb-2008-9-1-r2-S11.zip › htmls/C13_EPMs_matrix/EPM_9.RM.matrix.html]

Regulators vs. RM target gene list

|  |  |  |  |  |  |  |  |  |  |  |  |  |  |  |  |  |  |  |  |  |  |  |  |  |  |  |  |  |  |  |
| --- | --- | --- | --- | --- | --- | --- | --- | --- | --- | --- | --- | --- | --- | --- | --- | --- | --- | --- | --- | --- | --- | --- | --- | --- | --- | --- | --- | --- | --- | --- |
|  | Ste12 | Dig1 | Pho4 | Hap1 | Rph1 | Phd1 | Fkh2 | Gal4 | Gat3 | Stp1 | Yap5 | Tec1 | Swi6 | Swi4 | Mat1mc | Pdr3 | Dal82 | Pdr1 | Rcs1 | Stb4 | Ume1 | Abf1 | Gat1 | Mcm1 | Fkh1 | Ndd1 | Gcn4 | Gzf3 | Rap1 | Rds1 |
| RM\_1 |  |  |  |  |  |  |  |  |  |  |  |  |  |  |  |  |  |  |  |  |  |  |  |  |  |  |  |  |  |  |
| RM\_2 |  |  |  |  |  |  |  |  |  |  |  |  |  |  |  |  |  |  |  |  |  |  |  |  |  |  |  |  |  |  |
| RM\_3 |  |  |  |  |  |  |  |  |  |  |  |  |  |  |  |  |  |  |  |  |  |  |  |  |  |  |  |  |  |  |
| RM\_4 |  |  |  |  |  |  |  |  |  |  |  |  |  |  |  |  |  |  |  |  |  |  |  |  |  |  |  |  |  |  |

Synergistic Pair of Regulators

1. Phd1\*Yap5

2. Phd1\*Rap1

3. Mcm1\*Pdr1

4. Pdr1\*Ume1

5. Dal82\*Fkh1

6. Mcm1\*Ume1

7. Gat1\*Pdr1

8. Dal82\*Rcs1

9. Pdr1\*Stb4

10. Dal82\*Pdr1

11. Dal82\*Tec1

12. Gat1\*Mcm1

13. Fkh1\*Gat1

14. Fkh1\*Pdr1

15. Fkh1\*Rds1

16. Gcn4\*Pdr1

17. Gcn4\*Ume1

18. Ndd1\*Pdr1

19. Gcn4\*Mcm1

20. Fkh1\*Tec1

21. Dal82\*Mcm1

22. Gat1\*Rds1

23. Abf1\*Rcs1

24. Rcs1\*Tec1

25. Abf1\*Gat1

26. Dal82\*Rds1

27. Abf1\*Mcm1

28. Fkh1\*Mcm1

29. Gzf3\*Pdr1

30. Mcm1\*Stb4

31. Gat1\*Tec1

32. Gat1\*Ume1

33. Mcm1\*Rds1

34. Fkh1\*Gcn4

35. Gcn4\*Stb4

36. Pdr1\*Rcs1

37. Mcm1\*Rap1

38. Pdr1\*Tec1

39. Gzf3\*Rcs1

40. Gzf3\*Mcm1

41. Gat1\*Rcs1

42. Dal82\*Gcn4

43. Dal82\*Stb4

44. Pdr1\*Rap1

45. Gat1\*Stb4

46. Dal82\*Ume1

47. Fkh1\*Rcs1

48. Ndd1\*Ume1

49. Fkh1\*Stb4

50. Gat1\*Gcn4

51. Gcn4\*Tec1

52. Mcm1\*Rcs1

53. Fkh1\*Gzf3

54. Dal82\*Stp1

55. Dal82\*Swi4

56. Dal82\*Swi6

57. Fkh1\*Ume1

58. Mcm1\*Tec1

59. Gzf3\*Stb4

60. Gcn4\*Ndd1

61. Gzf3\*Tec1

62. Tec1\*Ume1

63. Stb4\*Ume1

64. Dal82\*Hap1

65. Gat1\*Stp1

66. Mat1mc\*Rcs1

67. Gat1\*Ndd1

68. Mat1mc\*Pdr1

69. Mat1mc\*Pdr3

70. Fkh1\*Ndd1

71. Dal82\*Pdr3

72. Mcm1\*Swi4

73. Mcm1\*Swi6

74. Fkh1\*Swi4

75. Fkh1\*Swi6

76. Dal82\*Ndd1

77. Mat1mc\*Swi4

78. Mat1mc\*Swi6

79. Gzf3\*Ndd1

80. Dal82\*Mat1mc

81. Pdr1\*Swi4

82. Pdr1\*Swi6

83. Rcs1\*Swi4

84. Rcs1\*Swi6

85. Ndd1\*Stb4

86. Gcn4\*Rcs1

87. Gcn4\*Gzf3

88. Gzf3\*Pdr3

89. Gat1\*Swi4

90. Gat1\*Swi6

91. Hap1\*Pdr1

92. Fkh1\*Pdr3

93. Mat1mc\*Stp1

94. Gat1\*Rap1

95. Gzf3\*Ume1

96. Pdr1\*Rph1

97. Mcm1\*Stp1

98. Gzf3\*Stp1

99. Dal82\*Rph1

100. Mat1mc\*Tec1

101. Pdr1\*Stp1

102. Gzf3\*Swi4

103. Gzf3\*Swi6

104. Gcn4\*Stp1

105. Dal82\*Gat1

106. Swi4\*Ume1

107. Swi6\*Ume1

108. Hap1\*Rcs1

109. Mcm1\*Ndd1

110. Stb4\*Tec1

111. Pdr3\*Tec1

112. Rcs1\*Ste12

113. Dig1\*Rcs1

114. Rcs1\*Rph1

115. Stb4\*Swi4

116. Stb4\*Swi6

117. Pdr3\*Swi4

118. Pdr3\*Swi6

119. Mat1mc\*Pho4

120. Fkh2\*Pdr1

121. Fkh2\*Pdr3

122. Abf1\*Ndd1

123. Rap1\*Tec1

124. Ndd1\*Rap1

125. Stp1\*Tec1

126. Ndd1\*Tec1

127. Hap1\*Rph1

128. Hap1\*Tec1

129. Rph1\*Tec1

130. Rap1\*Stp1

131. Ndd1\*Stp1

132. Swi4\*Tec1

133. Swi6\*Tec1

134. Stp1\*Swi4

135. Stp1\*Swi6

136. Ndd1\*Swi4

137. Ndd1\*Swi6

138. Gal4\*Swi4

139. Gal4\*Swi6

140. Dig1\*Swi4

141. Dig1\*Swi6

142. Ste12\*Swi4

143. Ste12\*Swi6

Matrix of enriched GO

EPM matrix
