# Supplementary material for: High-resolution analysis of condition-specific regulatory modules in Saccharomyces cerevisiae
Source: Genome Biol. 2008 Jan 3;9(1):R2. doi: 10.1186/gb-2008-9-1-r2 (PMC2395236; doi:10.1186/gb-2008-9-1-r2)
Supplement: Additional data file 11 — Matrices describing all EPMs and RMs, including lists of synergistic pairs of regulators. [file gb-2008-9-1-r2-S11.zip › htmls/C4_EPMs_matrix/EPM_1.GO_enrichment.matrix.html]

|  |  |  |  |  |  |  |  |
| --- | --- | --- | --- | --- | --- | --- | --- |
| Mcm1 | Stb1 | Mbp1 | Swi4 | Swi6 | Dig1 | Ste12 | Biological Process |
|  |  |  |  |  |  |  | P:double-strand break repair via synthesis-dependent strand annealing |
|  |  |  |  |  |  |  | P:double-strand break repair via single-strand annealing |
|  |  |  |  |  |  |  | P:meiosis |
|  |  |  |  |  |  |  | P:m phase of meiotic cell cycle |
|  |  |  |  |  |  |  | P:meiotic cell cycle |
|  |  |  |  |  |  |  | P:meiotic joint molecule formation |
|  |  |  |  |  |  |  | P:heteroduplex formation |
|  |  |  |  |  |  |  | P:biopolymer glycosylation |
|  |  |  |  |  |  |  | P:protein amino acid glycosylation |
|  |  |  |  |  |  |  | P:re-entry into mitotic cell cycle after pheromone arrest |
|  |  |  |  |  |  |  | P:re-entry into mitotic cell cycle |
|  |  |  |  |  |  |  | P:protein amino acid N-linked glycosylation |
|  |  |  |  |  |  |  | P:spindle assembly |
|  |  |  |  |  |  |  | P:cell cycle |
|  |  |  |  |  |  |  | P:regulation of nucleobase, nucleoside, nucleotide and nucleic acid metabolism |
|  |  |  |  |  |  |  | P:regulation of cellular process |
|  |  |  |  |  |  |  | P:regulation of cellular physiological process |
|  |  |  |  |  |  |  | P:regulation of progression through mitotic cell cycle |
|  |  |  |  |  |  |  | P:s phase of mitotic cell cycle |
|  |  |  |  |  |  |  | P:s phase |
|  |  |  |  |  |  |  | P:g1/S transition of mitotic cell cycle |
|  |  |  |  |  |  |  | P:regulation of DNA metabolism |
|  |  |  |  |  |  |  | P:regulation of S phase of mitotic cell cycle |
|  |  |  |  |  |  |  | P:interphase |
|  |  |  |  |  |  |  | P:interphase of mitotic cell cycle |
|  |  |  |  |  |  |  | P:premeiotic DNA synthesis |
|  |  |  |  |  |  |  | P:positive regulation of DNA replication |
|  |  |  |  |  |  |  | P:positive regulation of DNA metabolism |
|  |  |  |  |  |  |  | P:regulation of DNA replication |
|  |  |  |  |  |  |  | P:regulation of catalytic activity |
|  |  |  |  |  |  |  | P:regulation of protein kinase activity |
|  |  |  |  |  |  |  | P:regulation of kinase activity |
|  |  |  |  |  |  |  | P:regulation of transferase activity |
|  |  |  |  |  |  |  | P:regulation of cell cycle |
|  |  |  |  |  |  |  | P:regulation of progression through cell cycle |
|  |  |  |  |  |  |  | P:regulation of cyclin-dependent protein kinase activity |
|  |  |  |  |  |  |  | P:mitotic cell cycle |
|  |  |  |  |  |  |  | P:dNA replication |
|  |  |  |  |  |  |  | P:dNA-dependent DNA replication |
|  |  |  |  |  |  |  | P:negative regulation of nucleobase, nucleoside, nucleotide and nucleic acid metabolism |
|  |  |  |  |  |  |  | P:negative regulation of DNA metabolism |
|  |  |  |  |  |  |  | P:pyrimidine nucleoside monophosphate biosynthesis |
|  |  |  |  |  |  |  | P:deoxyribonucleoside monophosphate metabolism |
|  |  |  |  |  |  |  | P:dTMP metabolism |
|  |  |  |  |  |  |  | P:pyrimidine deoxyribonucleoside monophosphate biosynthesis |
|  |  |  |  |  |  |  | P:pyrimidine deoxyribonucleoside monophosphate metabolism |
|  |  |  |  |  |  |  | P:deoxyribonucleoside monophosphate biosynthesis |
|  |  |  |  |  |  |  | P:dTMP biosynthesis |
|  |  |  |  |  |  |  | P:pyrimidine nucleoside monophosphate metabolism |
|  |  |  |  |  |  |  | P:microtubule-based process |
|  |  |  |  |  |  |  | P:postreplication repair |
|  |  |  |  |  |  |  | P:telomere organization and biogenesis |
|  |  |  |  |  |  |  | P:telomere maintenance |
|  |  |  |  |  |  |  | P:chromosome organization and biogenesis |
|  |  |  |  |  |  |  | P:cell budding |
|  |  |  |  |  |  |  | P:asexual reproduction |
|  |  |  |  |  |  |  | P:g1/S-specific transcription in mitotic cell cycle |
|  |  |  |  |  |  |  | P:dNA unwinding during replication |
|  |  |  |  |  |  |  | P:chromosome condensation |
|  |  |  |  |  |  |  | P:response to stimulus |
|  |  |  |  |  |  |  | P:response to stress |
|  |  |  |  |  |  |  | P:mitotic chromosome condensation |
|  |  |  |  |  |  |  | P:nucleotide-excision repair |
|  |  |  |  |  |  |  | P:development |
|  |  |  |  |  |  |  | P:g2/M transition of mitotic cell cycle |
|  |  |  |  |  |  |  | P:dNA recombination |
|  |  |  |  |  |  |  | P:axial bud site selection |
|  |  |  |  |  |  |  | P:dNA replication, synthesis of RNA primer |
|  |  |  |  |  |  |  | P:biological\_process |
|  |  |  |  |  |  |  | P:septin checkpoint |
|  |  |  |  |  |  |  | P:dNA synthesis during DNA repair |
|  |  |  |  |  |  |  | P:reproduction |
|  |  |  |  |  |  |  | P:physiological process |
|  |  |  |  |  |  |  | P:regulation of biological process |
|  |  |  |  |  |  |  | P:cellular process |
|  |  |  |  |  |  |  | P:regulation of physiological process |
|  |  |  |  |  |  |  | P:cellular physiological process |
|  |  |  |  |  |  |  | P:biopolymer metabolism |
|  |  |  |  |  |  |  | P:nucleobase, nucleoside, nucleotide and nucleic acid metabolism |
|  |  |  |  |  |  |  | P:cell cycle checkpoint |
|  |  |  |  |  |  |  | P:chromosome segregation |
|  |  |  |  |  |  |  | P:response to endogenous stimulus |
|  |  |  |  |  |  |  | P:m phase of mitotic cell cycle |
|  |  |  |  |  |  |  | P:response to DNA damage stimulus |
|  |  |  |  |  |  |  | P:mitosis |
|  |  |  |  |  |  |  | P:m phase |
|  |  |  |  |  |  |  | P:dNA replication initiation |
|  |  |  |  |  |  |  | P:double-strand break repair |
|  |  |  |  |  |  |  | P:dNA repair |
|  |  |  |  |  |  |  | P:lagging strand elongation |
|  |  |  |  |  |  |  | P:sister chromatid segregation |
|  |  |  |  |  |  |  | P:mitotic sister chromatid segregation |
|  |  |  |  |  |  |  | P:dNA strand elongation |
|  |  |  |  |  |  |  | P:dNA metabolism |
|  |  |  |  |  |  |  | P:mitotic sister chromatid cohesion |
|  |  |  |  |  |  |  | P:sister chromatid cohesion |
|  |  |  |  |  |  |  | P:strand invasion |
|  |  |  |  |  |  |  | P:g2/M transition checkpoint |
|  |  |  |  |  |  |  | P:cell size control checkpoint |
|  |  |  |  |  |  |  | P:cell morphogenesis checkpoint |
|  |  |  |  |  |  |  | P:g2/M transition size control checkpoint |
|
| Mcm1 | Stb1 | Mbp1 | Swi4 | Swi6 | Dig1 | Ste12 | Molecular Function |
|  |  |  |  |  |  |  | F:recombinase activity |
|  |  |  |  |  |  |  | F:n-acetylglucosaminyldiphosphodolichol N-acetylglucosaminyltransferase activity |
|  |  |  |  |  |  |  | F:5,10-methylenetetrahydrofolate-dependent methyltransferase activity |
|  |  |  |  |  |  |  | F:thymidylate synthase activity |
|  |  |  |  |  |  |  | F:transferase activity, transferring phosphorus-containing groups |
|  |  |  |  |  |  |  | F:double-stranded DNA binding |
|  |  |  |  |  |  |  | F:protein kinase activity |
|  |  |  |  |  |  |  | F:sequence-specific DNA binding |
|  |  |  |  |  |  |  | F:structure-specific DNA binding |
|  |  |  |  |  |  |  | F:dNA secondary structure binding |
|  |  |  |  |  |  |  | F:molecular\_function |
|  |  |  |  |  |  |  | F:dNA binding |
|  |  |  |  |  |  |  | F:alpha DNA polymerase activity |
|  |  |  |  |  |  |  | F:protein anchor |
|  |  |  |  |  |  |  | F:enzyme regulator activity |
|  |  |  |  |  |  |  | F:kinase regulator activity |
|  |  |  |  |  |  |  | F:cyclin-dependent protein kinase regulator activity |
|  |  |  |  |  |  |  | F:protein kinase regulator activity |
|
| Mcm1 | Stb1 | Mbp1 | Swi4 | Swi6 | Dig1 | Ste12 | Cellular Component |
|  |  |  |  |  |  |  | C:vacuole |
|  |  |  |  |  |  |  | C:external encapsulating structure |
|  |  |  |  |  |  |  | C:cell wall (sensu Fungi) |
|  |  |  |  |  |  |  | C:cell wall |
|  |  |  |  |  |  |  | C:uDP-N-acetylglucosamine transferase complex |
|  |  |  |  |  |  |  | C:cyclin-dependent protein kinase holoenzyme complex |
|  |  |  |  |  |  |  | C:vacuole (sensu Fungi) |
|  |  |  |  |  |  |  | C:storage vacuole |
|  |  |  |  |  |  |  | C:lytic vacuole |
|  |  |  |  |  |  |  | C:septin cytoskeleton |
|  |  |  |  |  |  |  | C:septin ring |
|  |  |  |  |  |  |  | C:intracellular membrane-bound organelle |
|  |  |  |  |  |  |  | C:membrane-bound organelle |
|  |  |  |  |  |  |  | C:nuclear part |
|  |  |  |  |  |  |  | C:intracellular organelle |
|  |  |  |  |  |  |  | C:organelle |
|  |  |  |  |  |  |  | C:chromosome, telomeric region |
|  |  |  |  |  |  |  | C:alpha DNA polymerase:primase complex |
|  |  |  |  |  |  |  | C:cellular\_component |
|  |  |  |  |  |  |  | C:cell |
|  |  |  |  |  |  |  | C:cell part |
|  |  |  |  |  |  |  | C:dNA replication factor A complex |
|  |  |  |  |  |  |  | C:nucleus |
|  |  |  |  |  |  |  | C:intracellular non-membrane-bound organelle |
|  |  |  |  |  |  |  | C:non-membrane-bound organelle |
|  |  |  |  |  |  |  | C:condensed chromosome |
|  |  |  |  |  |  |  | C:condensed nuclear chromosome |
|  |  |  |  |  |  |  | C:replication fork (sensu Eukaryota) |
|  |  |  |  |  |  |  | C:replisome |
|  |  |  |  |  |  |  | C:replisome (sensu Eukaryota) |
|  |  |  |  |  |  |  | C:nuclear cohesin complex |
|  |  |  |  |  |  |  | C:cohesin complex |
|  |  |  |  |  |  |  | C:replication fork |
|  |  |  |  |  |  |  | C:nuclear chromosome part |
|  |  |  |  |  |  |  | C:chromosomal part |
|  |  |  |  |  |  |  | C:chromosome |
|  |  |  |  |  |  |  | C:nuclear chromosome |
|  |  |  |  |  |  |  | C:site of polarized growth |
|  |  |  |  |  |  |  | C:bud neck |
|  |  |  |  |  |  |  | C:bud |
|
