# Supplementary material for: High-resolution analysis of condition-specific regulatory modules in Saccharomyces cerevisiae
Source: Genome Biol. 2008 Jan 3;9(1):R2. doi: 10.1186/gb-2008-9-1-r2 (PMC2395236; doi:10.1186/gb-2008-9-1-r2)
Supplement: Additional data file 11 — Matrices describing all EPMs and RMs, including lists of synergistic pairs of regulators. [file gb-2008-9-1-r2-S11.zip › htmls/C4_EPMs_matrix/EPM_1.Overlap.matrix.html]

|  |  |  |  |  |  |  |
| --- | --- | --- | --- | --- | --- | --- |
| Mcm1 | Stb1 | Mbp1 | Swi4 | Swi6 | Dig1 | Ste12 |
|  |  |  |  |  |  |  | Mcm1 |
|  |  |  |  |  |  |  | Stb1 |
|  |  |  |  |  |  |  | Mbp1 |
|  |  |  |  |  |  |  | Swi4 |
|  |  |  |  |  |  |  | Swi6 |
|  |  |  |  |  |  |  | Dig1 |
|  |  |  |  |  |  |  | Ste12 |
 Mcm1 | Stb1 | Mbp1 | Swi4 | Swi6 | Dig1 | Ste12 |
