# Supplementary material for: High-resolution analysis of condition-specific regulatory modules in Saccharomyces cerevisiae
Source: Genome Biol. 2008 Jan 3;9(1):R2. doi: 10.1186/gb-2008-9-1-r2 (PMC2395236; doi:10.1186/gb-2008-9-1-r2)
Supplement: Additional data file 11 — Matrices describing all EPMs and RMs, including lists of synergistic pairs of regulators. [file gb-2008-9-1-r2-S11.zip › htmls/C4_EPMs_matrix/EPM_1.RM.matrix.html]

Regulators vs. RM target gene list

|  |  |  |  |  |  |  |  |
| --- | --- | --- | --- | --- | --- | --- | --- |
|  | Mcm1 | Stb1 | Mbp1 | Swi4 | Swi6 | Dig1 | Ste12 |
| RM\_1 |  |  |  |  |  |  |  |
| RM\_2 |  |  |  |  |  |  |  |
| RM\_3 |  |  |  |  |  |  |  |

Synergistic Pair of Regulators

1. Dig1\*Ste12

2. Swi4\*Swi6

3. Mbp1\*Swi6

4. Mbp1\*Stb1

5. Mbp1\*Swi4

6. Stb1\*Swi4

7. Stb1\*Swi6

8. Mcm1\*Swi6

9. Mcm1\*Swi4

10. Mcm1\*Stb1

Matrix of enriched GO

EPM matrix
