# Supplementary material for: High-resolution analysis of condition-specific regulatory modules in Saccharomyces cerevisiae
Source: Genome Biol. 2008 Jan 3;9(1):R2. doi: 10.1186/gb-2008-9-1-r2 (PMC2395236; doi:10.1186/gb-2008-9-1-r2)
Supplement: Additional data file 11 — Matrices describing all EPMs and RMs, including lists of synergistic pairs of regulators. [file gb-2008-9-1-r2-S11.zip › htmls/C4_EPMs_matrix/EPM_2.GO_enrichment.matrix.html]

|  |  |
| --- | --- |
| Nrg1 | Biological Process |
|  | P:pyridoxine metabolism |
|  | P:prospore formation |
|  | P:vitamin B6 metabolism |
|
| Nrg1 | Molecular Function |
|  | F:galactose transporter activity |
|  | F:t-SNARE activity |
|
| Nrg1 | Cellular Component |
|
