# Supplementary material for: High-resolution analysis of condition-specific regulatory modules in Saccharomyces cerevisiae
Source: Genome Biol. 2008 Jan 3;9(1):R2. doi: 10.1186/gb-2008-9-1-r2 (PMC2395236; doi:10.1186/gb-2008-9-1-r2)
Supplement: Additional data file 11 — Matrices describing all EPMs and RMs, including lists of synergistic pairs of regulators. [file gb-2008-9-1-r2-S11.zip › htmls/C4_EPMs_matrix/EPM_3.GO_enrichment.matrix.html]

|  |  |
| --- | --- |
| Hsf1 | Biological Process |
|  | P:mitochondrial signaling pathway |
|  | P:proteasome assembly |
|  | P:copper ion import |
|  | P:protein thiol-disulfide exchange |
|  | P:posttranslational protein folding |
|  | P:chaperone cofactor-dependent protein folding |
|  | P:response to stimulus |
|  | P:protein metabolism |
|  | P:cellular macromolecule metabolism |
|  | P:cellular protein metabolism |
|  | P:response to stress |
|  | P:protein folding |
|  | P:protein refolding |
|
| Hsf1 | Molecular Function |
|  | F:aTPase stimulator activity |
|  | F:copper ion transporter activity |
|  | F:hydrolase activity, acting on acid anhydrides, in phosphorus-containing anhydrides |
|  | F:hydrolase activity, acting on acid anhydrides |
|  | F:pyrophosphatase activity |
|  | F:nucleoside-triphosphatase activity |
|  | F:copper uptake transporter activity |
|  | F:enzyme activator activity |
|  | F:chaperone activator activity |
|  | F:aTPase activity |
|  | F:binding |
|  | F:chaperone binding |
|  | F:chaperone regulator activity |
|  | F:unfolded protein binding |
|  | F:protein binding |
|
| Hsf1 | Cellular Component |
|  | C:tORC 1 complex |
|  | C:extrinsic to internal side of plasma membrane |
|  | C:internal side of plasma membrane |
|
