# Supplementary material for: High-resolution analysis of condition-specific regulatory modules in Saccharomyces cerevisiae
Source: Genome Biol. 2008 Jan 3;9(1):R2. doi: 10.1186/gb-2008-9-1-r2 (PMC2395236; doi:10.1186/gb-2008-9-1-r2)
Supplement: Additional data file 11 — Matrices describing all EPMs and RMs, including lists of synergistic pairs of regulators. [file gb-2008-9-1-r2-S11.zip › htmls/C4_EPMs_matrix/EPM_5.GO_enrichment.matrix.html]

|  |  |  |  |  |  |
| --- | --- | --- | --- | --- | --- |
| Mcm1 | Fkh1 | Fkh2 | Ndd1 | Zap1 | Biological Process |
|  |  |  |  |  | P:zinc ion transport |
|  |  |  |  |  | P:physiological process |
|  |  |  |  |  | P:cellular physiological process |
|  |  |  |  |  | P:cellular process |
|  |  |  |  |  | P:nuclear division |
|  |  |  |  |  | P:positive regulation of ubiquitin ligase activity |
|  |  |  |  |  | P:regulation of ubiquitin ligase activity |
|  |  |  |  |  | P:positive regulation of ligase activity |
|  |  |  |  |  | P:regulation of ligase activity |
|  |  |  |  |  | P:mitotic spindle organization and biogenesis |
|  |  |  |  |  | P:spindle organization and biogenesis |
|  |  |  |  |  | P:regulation of ubiquitin ligase activity during mitotic cell cycle |
|  |  |  |  |  | P:positive regulation of ubiquitin ligase activity during mitotic cell cycle |
|  |  |  |  |  | P:anaphase-promoting complex activation |
|  |  |  |  |  | P:anaphase-promoting complex activation during mitotic cell cycle |
|  |  |  |  |  | P:cellular morphogenesis |
|  |  |  |  |  | P:morphogenesis |
|  |  |  |  |  | P:development |
|  |  |  |  |  | P:establishment and/or maintenance of cell polarity |
|  |  |  |  |  | P:establishment and/or maintenance of cell polarity (sensu Fungi) |
|  |  |  |  |  | P:meiotic G2/MI transition |
|  |  |  |  |  | P:establishment of cell polarity |
|  |  |  |  |  | P:establishment of cell polarity (sensu Fungi) |
|  |  |  |  |  | P:cytoskeleton organization and biogenesis |
|  |  |  |  |  | P:g2/M transition of mitotic cell cycle |
|  |  |  |  |  | P:g1 phase of mitotic cell cycle |
|  |  |  |  |  | P:g1 phase |
|  |  |  |  |  | P:microtubule-based process |
|  |  |  |  |  | P:regulation of protein kinase activity |
|  |  |  |  |  | P:regulation of kinase activity |
|  |  |  |  |  | P:regulation of transferase activity |
|  |  |  |  |  | P:regulation of cell cycle |
|  |  |  |  |  | P:regulation of progression through cell cycle |
|  |  |  |  |  | P:microtubule cytoskeleton organization and biogenesis |
|  |  |  |  |  | P:bud site selection |
|  |  |  |  |  | P:cytokinesis, site selection |
|  |  |  |  |  | P:g1-specific transcription in mitotic cell cycle |
|  |  |  |  |  | P:regulation of cyclin-dependent protein kinase activity |
|  |  |  |  |  | P:cell cycle |
|  |  |  |  |  | P:interphase |
|  |  |  |  |  | P:interphase of mitotic cell cycle |
|  |  |  |  |  | P:cell division |
|  |  |  |  |  | P:regulation of catalytic activity |
|  |  |  |  |  | P:cytokinesis |
|  |  |  |  |  | P:mitotic cell cycle |
|  |  |  |  |  | P:regulation of biological process |
|  |  |  |  |  | P:regulation of physiological process |
|  |  |  |  |  | P:regulation of cellular physiological process |
|  |  |  |  |  | P:regulation of cellular process |
|  |  |  |  |  | P:high-affinity zinc ion transport |
|  |  |  |  |  | P:protein amino acid phosphorylation |
|
| Mcm1 | Fkh1 | Fkh2 | Ndd1 | Zap1 | Molecular Function |
|  |  |  |  |  | F:zinc ion transporter activity |
|  |  |  |  |  | F:transcriptional activator activity |
|  |  |  |  |  | F:kinase regulator activity |
|  |  |  |  |  | F:cytoskeletal protein binding |
|  |  |  |  |  | F:protein serine/threonine kinase activity |
|  |  |  |  |  | F:polo kinase kinase activity |
|  |  |  |  |  | F:protein kinase regulator activity |
|  |  |  |  |  | F:protein kinase activity |
|  |  |  |  |  | F:cyclin-dependent protein kinase regulator activity |
|  |  |  |  |  | F:phosphotransferase activity, alcohol group as acceptor |
|  |  |  |  |  | F:kinase activity |
|  |  |  |  |  | F:enzyme regulator activity |
|  |  |  |  |  | F:high affinity zinc uptake transporter activity |
|  |  |  |  |  | F:signal transducer activity |
|  |  |  |  |  | F:receptor activity |
|
| Mcm1 | Fkh1 | Fkh2 | Ndd1 | Zap1 | Cellular Component |
|  |  |  |  |  | C:cell wall part |
|  |  |  |  |  | C:external encapsulating structure part |
|  |  |  |  |  | C:bud tip |
|  |  |  |  |  | C:intracellular organelle |
|  |  |  |  |  | C:organelle |
|  |  |  |  |  | C:intracellular part |
|  |  |  |  |  | C:intracellular |
|  |  |  |  |  | C:microtubule cytoskeleton |
|  |  |  |  |  | C:spindle |
|  |  |  |  |  | C:spindle pole |
|  |  |  |  |  | C:bud |
|  |  |  |  |  | C:site of polarized growth |
|  |  |  |  |  | C:bud scar |
|  |  |  |  |  | C:cell |
|  |  |  |  |  | C:cell part |
|  |  |  |  |  | C:cell cortex part |
|  |  |  |  |  | C:cell division site |
|  |  |  |  |  | C:cell division site part |
|  |  |  |  |  | C:actin cytoskeleton |
|  |  |  |  |  | C:bud neck contractile ring |
|  |  |  |  |  | C:contractile ring |
|  |  |  |  |  | C:cell cortex |
|  |  |  |  |  | C:cytoskeleton |
|  |  |  |  |  | C:bud neck |
|  |  |  |  |  | C:cytoskeletal part |
|
