# Supplementary material for: High-resolution analysis of condition-specific regulatory modules in Saccharomyces cerevisiae
Source: Genome Biol. 2008 Jan 3;9(1):R2. doi: 10.1186/gb-2008-9-1-r2 (PMC2395236; doi:10.1186/gb-2008-9-1-r2)
Supplement: Additional data file 11 — Matrices describing all EPMs and RMs, including lists of synergistic pairs of regulators. [file gb-2008-9-1-r2-S11.zip › htmls/C4_EPMs_matrix/EPM_6.GO_enrichment.matrix.html]

|  |  |  |  |  |
| --- | --- | --- | --- | --- |
| Nrg1 | Mcm1 | Fkh2 | Ndd1 | Biological Process |
|  |  |  |  | P:phospholipid transport |
|  |  |  |  | P:siderophore transport |
|  |  |  |  | P:response to reactive oxygen species |
|  |  |  |  | P:plasma membrane organization and biogenesis |
|  |  |  |  | P:phospholipid translocation |
|  |  |  |  | P:siderophore-iron transport |
|  |  |  |  | P:propionate metabolism |
|  |  |  |  | P:fatty acid transport |
|  |  |  |  | P:response to singlet oxygen |
|  |  |  |  | P:enterobactin transport |
|  |  |  |  | P:ferric-enterobactin transport |
|  |  |  |  | P:ferric iron transport |
|  |  |  |  | P:chromosome segregation |
|  |  |  |  | P:cytokinesis |
|  |  |  |  | P:1,6-beta-glucan metabolism |
|  |  |  |  | P:regulation of mitotic metaphase/anaphase transition |
|  |  |  |  | P:1,6-beta-glucan biosynthesis |
|  |  |  |  | P:nuclear division |
|  |  |  |  | P:positive regulation of ubiquitin ligase activity |
|  |  |  |  | P:regulation of ubiquitin ligase activity |
|  |  |  |  | P:positive regulation of ligase activity |
|  |  |  |  | P:regulation of ligase activity |
|  |  |  |  | P:regulation of ubiquitin ligase activity during mitotic cell cycle |
|  |  |  |  | P:positive regulation of ubiquitin ligase activity during mitotic cell cycle |
|  |  |  |  | P:cytokinesis, contractile ring contraction |
|  |  |  |  | P:anaphase-promoting complex activation |
|  |  |  |  | P:regulation of mitosis |
|  |  |  |  | P:anaphase-promoting complex activation during mitotic cell cycle |
|
| Nrg1 | Mcm1 | Fkh2 | Ndd1 | Molecular Function |
|  |  |  |  | F:iron ion transporter activity |
|  |  |  |  | F:phospholipid-translocating ATPase activity |
|  |  |  |  | F:aminophospholipid transporter activity |
|  |  |  |  | F:ion transporter activity |
|  |  |  |  | F:cation transporter activity |
|  |  |  |  | F:siderophore transporter activity |
|  |  |  |  | F:siderophore-iron transporter activity |
|  |  |  |  | F:ferric-enterobactin transporter activity |
|  |  |  |  | F:hydrolase activity, acting on acid anhydrides, in phosphorus-containing anhydrides |
|  |  |  |  | F:hydrolase activity, acting on acid anhydrides |
|  |  |  |  | F:pyrophosphatase activity |
|  |  |  |  | F:nucleoside-triphosphatase activity |
|  |  |  |  | F:aTPase activity |
|  |  |  |  | F:transporter activity |
|  |  |  |  | F:aTPase activity, coupled |
|  |  |  |  | F:drug transporter activity |
|  |  |  |  | F:multidrug transporter activity |
|  |  |  |  | F:aTPase activity, coupled to transmembrane movement of substances |
|  |  |  |  | F:hydrolase activity, acting on acid anhydrides, catalyzing transmembrane movement of substances |
|  |  |  |  | F:aTPase activity, coupled to movement of substances |
|  |  |  |  | F:xenobiotic-transporting ATPase activity |
|  |  |  |  | F:xenobiotic transporter activity |
|  |  |  |  | F:chitin synthase activity |
|  |  |  |  | F:acetylglucosaminyltransferase activity |
|
| Nrg1 | Mcm1 | Fkh2 | Ndd1 | Cellular Component |
|  |  |  |  | C:site of polarized growth |
|  |  |  |  | C:bud neck |
|  |  |  |  | C:bud |
|  |  |  |  | C:plasma membrane |
|  |  |  |  | C:membrane |
|
