# Supplementary material for: High-resolution analysis of condition-specific regulatory modules in Saccharomyces cerevisiae
Source: Genome Biol. 2008 Jan 3;9(1):R2. doi: 10.1186/gb-2008-9-1-r2 (PMC2395236; doi:10.1186/gb-2008-9-1-r2)
Supplement: Additional data file 11 — Matrices describing all EPMs and RMs, including lists of synergistic pairs of regulators. [file gb-2008-9-1-r2-S11.zip › htmls/C4_EPMs_matrix/EPM_7.GO_enrichment.matrix.html]

|  |  |  |  |  |  |  |  |  |  |  |  |
| --- | --- | --- | --- | --- | --- | --- | --- | --- | --- | --- | --- |
| Rpn4 | Sut1 | Reb1 | Ydr026c | Gcn4 | Hap5 | Mig1 | Hap4 | Hap3 | Hap1 | Hap2 | Biological Process |
|  |  |  |  |  |  |  |  |  |  |  | P:golgi to endosome transport |
|  |  |  |  |  |  |  |  |  |  |  | P:amino acid and derivative metabolism |
|  |  |  |  |  |  |  |  |  |  |  | P:amino acid metabolism |
|  |  |  |  |  |  |  |  |  |  |  | P:regulation of nitrogen metabolism |
|  |  |  |  |  |  |  |  |  |  |  | P:regulation of nitrogen utilization |
|  |  |  |  |  |  |  |  |  |  |  | P:nitrogen compound biosynthesis |
|  |  |  |  |  |  |  |  |  |  |  | P:amine biosynthesis |
|  |  |  |  |  |  |  |  |  |  |  | P:amino acid biosynthesis |
|  |  |  |  |  |  |  |  |  |  |  | P:heterocycle metabolism |
|  |  |  |  |  |  |  |  |  |  |  | P:aromatic compound metabolism |
|  |  |  |  |  |  |  |  |  |  |  | P:nitrogen compound metabolism |
|  |  |  |  |  |  |  |  |  |  |  | P:amino acid derivative metabolism |
|  |  |  |  |  |  |  |  |  |  |  | P:biogenic amine metabolism |
|  |  |  |  |  |  |  |  |  |  |  | P:amino acid derivative biosynthesis |
|  |  |  |  |  |  |  |  |  |  |  | P:aromatic amino acid family metabolism |
|  |  |  |  |  |  |  |  |  |  |  | P:biogenic amine biosynthesis |
|  |  |  |  |  |  |  |  |  |  |  | P:aromatic compound biosynthesis |
|  |  |  |  |  |  |  |  |  |  |  | P:aromatic amino acid family biosynthesis |
|  |  |  |  |  |  |  |  |  |  |  | P:indole derivative biosynthesis |
|  |  |  |  |  |  |  |  |  |  |  | P:indole derivative metabolism |
|  |  |  |  |  |  |  |  |  |  |  | P:indole and derivative metabolism |
|  |  |  |  |  |  |  |  |  |  |  | P:tryptophan metabolism |
|  |  |  |  |  |  |  |  |  |  |  | P:indolalkylamine metabolism |
|  |  |  |  |  |  |  |  |  |  |  | P:tryptophan biosynthesis |
|  |  |  |  |  |  |  |  |  |  |  | P:indolalkylamine biosynthesis |
|  |  |  |  |  |  |  |  |  |  |  | P:aromatic amino acid family biosynthesis, anthranilate pathway |
|  |  |  |  |  |  |  |  |  |  |  | P:macromolecule metabolism |
|  |  |  |  |  |  |  |  |  |  |  | P:primary metabolism |
|  |  |  |  |  |  |  |  |  |  |  | P:catabolism |
|  |  |  |  |  |  |  |  |  |  |  | P:cellular catabolism |
|  |  |  |  |  |  |  |  |  |  |  | P:biopolymer modification |
|  |  |  |  |  |  |  |  |  |  |  | P:macromolecule catabolism |
|  |  |  |  |  |  |  |  |  |  |  | P:protein modification |
|  |  |  |  |  |  |  |  |  |  |  | P:cellular macromolecule catabolism |
|  |  |  |  |  |  |  |  |  |  |  | P:biopolymer catabolism |
|  |  |  |  |  |  |  |  |  |  |  | P:protein metabolism |
|  |  |  |  |  |  |  |  |  |  |  | P:proteolysis |
|  |  |  |  |  |  |  |  |  |  |  | P:protein catabolism |
|  |  |  |  |  |  |  |  |  |  |  | P:cellular macromolecule metabolism |
|  |  |  |  |  |  |  |  |  |  |  | P:cellular protein catabolism |
|  |  |  |  |  |  |  |  |  |  |  | P:cellular protein metabolism |
|  |  |  |  |  |  |  |  |  |  |  | P:proteolysis during cellular protein catabolism |
|  |  |  |  |  |  |  |  |  |  |  | P:modification-dependent macromolecule catabolism |
|  |  |  |  |  |  |  |  |  |  |  | P:ubiquitin-dependent protein catabolism |
|  |  |  |  |  |  |  |  |  |  |  | P:ubiquitin cycle |
|  |  |  |  |  |  |  |  |  |  |  | P:modification-dependent protein catabolism |
|  |  |  |  |  |  |  |  |  |  |  | P:glucan metabolism |
|  |  |  |  |  |  |  |  |  |  |  | P:cellular process |
|  |  |  |  |  |  |  |  |  |  |  | P:physiological process |
|  |  |  |  |  |  |  |  |  |  |  | P:cellular physiological process |
|  |  |  |  |  |  |  |  |  |  |  | P:cellular polysaccharide metabolism |
|  |  |  |  |  |  |  |  |  |  |  | P:polysaccharide metabolism |
|  |  |  |  |  |  |  |  |  |  |  | P:amine transport |
|  |  |  |  |  |  |  |  |  |  |  | P:nADH oxidation |
|  |  |  |  |  |  |  |  |  |  |  | P:c-terminal protein lipidation |
|  |  |  |  |  |  |  |  |  |  |  | P:peptide or protein carboxyl-terminal blocking |
|  |  |  |  |  |  |  |  |  |  |  | P:metallo-sulfur cluster assembly |
|  |  |  |  |  |  |  |  |  |  |  | P:iron-sulfur cluster assembly |
|  |  |  |  |  |  |  |  |  |  |  | P:cellular metabolism |
|  |  |  |  |  |  |  |  |  |  |  | P:metabolism |
|  |  |  |  |  |  |  |  |  |  |  | P:electron transport |
|  |  |  |  |  |  |  |  |  |  |  | P:generation of precursor metabolites and energy |
|  |  |  |  |  |  |  |  |  |  |  | P:mitochondrial electron transport, cytochrome c to oxygen |
|  |  |  |  |  |  |  |  |  |  |  | P:cellular respiration |
|  |  |  |  |  |  |  |  |  |  |  | P:aerobic respiration |
|  |  |  |  |  |  |  |  |  |  |  | P:mitochondrial electron transport, ubiquinol to cytochrome c |
|  |  |  |  |  |  |  |  |  |  |  | P:phosphate metabolism |
|  |  |  |  |  |  |  |  |  |  |  | P:phosphorus metabolism |
|  |  |  |  |  |  |  |  |  |  |  | P:phosphorylation |
|  |  |  |  |  |  |  |  |  |  |  | P:aTP synthesis coupled electron transport (sensu Eukaryota) |
|  |  |  |  |  |  |  |  |  |  |  | P:oxidative phosphorylation |
|  |  |  |  |  |  |  |  |  |  |  | P:aTP synthesis coupled electron transport |
|  |  |  |  |  |  |  |  |  |  |  | P:energy derivation by oxidation of organic compounds |
|  |  |  |  |  |  |  |  |  |  |  | P:purine ribonucleotide biosynthesis |
|  |  |  |  |  |  |  |  |  |  |  | P:monovalent inorganic cation transport |
|  |  |  |  |  |  |  |  |  |  |  | P:nucleotide metabolism |
|  |  |  |  |  |  |  |  |  |  |  | P:nucleoside triphosphate metabolism |
|  |  |  |  |  |  |  |  |  |  |  | P:nucleoside triphosphate biosynthesis |
|  |  |  |  |  |  |  |  |  |  |  | P:proton transport |
|  |  |  |  |  |  |  |  |  |  |  | P:hydrogen transport |
|  |  |  |  |  |  |  |  |  |  |  | P:ribonucleoside triphosphate biosynthesis |
|  |  |  |  |  |  |  |  |  |  |  | P:ribonucleoside triphosphate metabolism |
|  |  |  |  |  |  |  |  |  |  |  | P:purine nucleoside triphosphate metabolism |
|  |  |  |  |  |  |  |  |  |  |  | P:purine nucleoside triphosphate biosynthesis |
|  |  |  |  |  |  |  |  |  |  |  | P:purine ribonucleoside triphosphate biosynthesis |
|  |  |  |  |  |  |  |  |  |  |  | P:purine ribonucleoside triphosphate metabolism |
|  |  |  |  |  |  |  |  |  |  |  | P:nucleoside phosphate metabolism |
|  |  |  |  |  |  |  |  |  |  |  | P:aTP biosynthesis |
|  |  |  |  |  |  |  |  |  |  |  | P:energy coupled proton transport, down electrochemical gradient |
|  |  |  |  |  |  |  |  |  |  |  | P:aTP synthesis coupled proton transport |
|  |  |  |  |  |  |  |  |  |  |  | P:aTP metabolism |
|  |  |  |  |  |  |  |  |  |  |  | P:cofactor metabolism |
|  |  |  |  |  |  |  |  |  |  |  | P:coenzyme metabolism |
|  |  |  |  |  |  |  |  |  |  |  | P:purine nucleotide metabolism |
|  |  |  |  |  |  |  |  |  |  |  | P:group transfer coenzyme metabolism |
|  |  |  |  |  |  |  |  |  |  |  | P:ribonucleotide metabolism |
|  |  |  |  |  |  |  |  |  |  |  | P:purine nucleotide biosynthesis |
|  |  |  |  |  |  |  |  |  |  |  | P:purine ribonucleotide metabolism |
|  |  |  |  |  |  |  |  |  |  |  | P:ribonucleotide biosynthesis |
|
| Rpn4 | Sut1 | Reb1 | Ydr026c | Gcn4 | Hap5 | Mig1 | Hap4 | Hap3 | Hap1 | Hap2 | Molecular Function |
|  |  |  |  |  |  |  |  |  |  |  | F:oxo-acid-lyase activity |
|  |  |  |  |  |  |  |  |  |  |  | F:anthranilate synthase activity |
|  |  |  |  |  |  |  |  |  |  |  | F:anthranilate phosphoribosyltransferase activity |
|  |  |  |  |  |  |  |  |  |  |  | F:indole-3-glycerol-phosphate synthase activity |
|  |  |  |  |  |  |  |  |  |  |  | F:electron carrier activity |
|  |  |  |  |  |  |  |  |  |  |  | F:catalytic activity |
|  |  |  |  |  |  |  |  |  |  |  | F:electron transporter, transferring electrons within CoQH2-cytochrome c reductase complex activity |
|  |  |  |  |  |  |  |  |  |  |  | F:ubiquinol-cytochrome-c reductase activity |
|  |  |  |  |  |  |  |  |  |  |  | F:oxidoreductase activity, acting on diphenols and related substances as donors |
|  |  |  |  |  |  |  |  |  |  |  | F:oxidoreductase activity, acting on diphenols and related substances as donors, cytochrome as acceptor |
|  |  |  |  |  |  |  |  |  |  |  | F:oxidoreductase activity |
|  |  |  |  |  |  |  |  |  |  |  | F:transporter activity |
|  |  |  |  |  |  |  |  |  |  |  | F:ion transporter activity |
|  |  |  |  |  |  |  |  |  |  |  | F:cation transporter activity |
|  |  |  |  |  |  |  |  |  |  |  | F:monovalent inorganic cation transporter activity |
|  |  |  |  |  |  |  |  |  |  |  | F:hydrogen ion transporter activity |
|  |  |  |  |  |  |  |  |  |  |  | F:hydroxymethylglutaryl-CoA reductase (NADPH) activity |
|  |  |  |  |  |  |  |  |  |  |  | F:cytochrome-c oxidase activity |
|  |  |  |  |  |  |  |  |  |  |  | F:heme-copper terminal oxidase activity |
|  |  |  |  |  |  |  |  |  |  |  | F:oxidoreductase activity, acting on heme group of donors |
|  |  |  |  |  |  |  |  |  |  |  | F:oxidoreductase activity, acting on heme group of donors, oxygen as acceptor |
|  |  |  |  |  |  |  |  |  |  |  | F:phospholipid-hydroperoxide glutathione peroxidase activity |
|  |  |  |  |  |  |  |  |  |  |  | F:glutathione peroxidase activity |
|  |  |  |  |  |  |  |  |  |  |  | F:l-proline transporter activity |
|  |  |  |  |  |  |  |  |  |  |  | F:l-proline permease activity |
|  |  |  |  |  |  |  |  |  |  |  | F:endopeptidase activity |
|  |  |  |  |  |  |  |  |  |  |  | F:peptidase activity |
|  |  |  |  |  |  |  |  |  |  |  | F:uDP-glucose:glycoprotein glucosyltransferase activity |
|  |  |  |  |  |  |  |  |  |  |  | F:diacylglycerol pyrophosphate phosphatase activity |
|  |  |  |  |  |  |  |  |  |  |  | F:phosphatidate phosphatase activity |
|  |  |  |  |  |  |  |  |  |  |  | F:nADH dehydrogenase activity |
|  |  |  |  |  |  |  |  |  |  |  | F:polyamine transporter activity |
|  |  |  |  |  |  |  |  |  |  |  | F:amine transporter activity |
|  |  |  |  |  |  |  |  |  |  |  | F:spermidine transporter activity |
|  |  |  |  |  |  |  |  |  |  |  | F:aconitate hydratase activity |
|
| Rpn4 | Sut1 | Reb1 | Ydr026c | Gcn4 | Hap5 | Mig1 | Hap4 | Hap3 | Hap1 | Hap2 | Cellular Component |
|  |  |  |  |  |  |  |  |  |  |  | C:proteasome regulatory particle (sensu Eukaryota) |
|  |  |  |  |  |  |  |  |  |  |  | C:proteasome core complex (sensu Eukaryota) |
|  |  |  |  |  |  |  |  |  |  |  | C:proteasome complex (sensu Eukaryota) |
|  |  |  |  |  |  |  |  |  |  |  | C:proteasome core complex, beta-subunit complex (sensu Eukaryota) |
|  |  |  |  |  |  |  |  |  |  |  | C:organellar ribosome |
|  |  |  |  |  |  |  |  |  |  |  | C:mitochondrial ribosome |
|  |  |  |  |  |  |  |  |  |  |  | C:anthranilate synthase complex |
|  |  |  |  |  |  |  |  |  |  |  | C:autophagic vacuole |
|  |  |  |  |  |  |  |  |  |  |  | C:cell part |
|  |  |  |  |  |  |  |  |  |  |  | C:cell |
|  |  |  |  |  |  |  |  |  |  |  | C:intracellular part |
|  |  |  |  |  |  |  |  |  |  |  | C:intracellular |
|  |  |  |  |  |  |  |  |  |  |  | C:protein complex |
|  |  |  |  |  |  |  |  |  |  |  | C:mitochondrial envelope |
|  |  |  |  |  |  |  |  |  |  |  | C:mitochondrial membrane |
|  |  |  |  |  |  |  |  |  |  |  | C:mitochondrial membrane part |
|  |  |  |  |  |  |  |  |  |  |  | C:organelle inner membrane |
|  |  |  |  |  |  |  |  |  |  |  | C:mitochondrial electron transport chain |
|  |  |  |  |  |  |  |  |  |  |  | C:mitochondrial inner membrane |
|  |  |  |  |  |  |  |  |  |  |  | C:ubiquinol-cytochrome-c reductase complex |
|  |  |  |  |  |  |  |  |  |  |  | C:respiratory chain complex III (sensu Eukaryota) |
|  |  |  |  |  |  |  |  |  |  |  | C:respiratory chain complex III |
|  |  |  |  |  |  |  |  |  |  |  | C:mitochondrion |
|  |  |  |  |  |  |  |  |  |  |  | C:mitochondrial part |
|  |  |  |  |  |  |  |  |  |  |  | C:respiratory chain complex IV |
|  |  |  |  |  |  |  |  |  |  |  | C:respiratory chain complex IV (sensu Eukaryota) |
|  |  |  |  |  |  |  |  |  |  |  | C:organelle envelope |
|  |  |  |  |  |  |  |  |  |  |  | C:envelope |
|  |  |  |  |  |  |  |  |  |  |  | C:membrane-bound organelle |
|  |  |  |  |  |  |  |  |  |  |  | C:intracellular membrane-bound organelle |
|  |  |  |  |  |  |  |  |  |  |  | C:cytoplasmic part |
|  |  |  |  |  |  |  |  |  |  |  | C:cytoplasm |
|  |  |  |  |  |  |  |  |  |  |  | C:organelle |
|  |  |  |  |  |  |  |  |  |  |  | C:intracellular organelle |
|  |  |  |  |  |  |  |  |  |  |  | C:hydrogen-translocating F-type ATPase complex |
|  |  |  |  |  |  |  |  |  |  |  | C:proton-transporting ATP synthase complex |
|  |  |  |  |  |  |  |  |  |  |  | C:proton-transporting two-sector ATPase complex |
|  |  |  |  |  |  |  |  |  |  |  | C:proton-transporting ATP synthase complex (sensu Eukaryota) |
|  |  |  |  |  |  |  |  |  |  |  | C:intracellular organelle part |
|  |  |  |  |  |  |  |  |  |  |  | C:organelle part |
|  |  |  |  |  |  |  |  |  |  |  | C:organelle membrane |
|  |  |  |  |  |  |  |  |  |  |  | C:membrane part |
|  |  |  |  |  |  |  |  |  |  |  | C:membrane |
|
