# Supplementary material for: High-resolution analysis of condition-specific regulatory modules in Saccharomyces cerevisiae
Source: Genome Biol. 2008 Jan 3;9(1):R2. doi: 10.1186/gb-2008-9-1-r2 (PMC2395236; doi:10.1186/gb-2008-9-1-r2)
Supplement: Additional data file 11 — Matrices describing all EPMs and RMs, including lists of synergistic pairs of regulators. [file gb-2008-9-1-r2-S11.zip › htmls/C4_EPMs_matrix/EPM_7.Overlap.matrix.html]

|  |  |  |  |  |  |  |  |  |  |  |
| --- | --- | --- | --- | --- | --- | --- | --- | --- | --- | --- |
| Rpn4 | Sut1 | Reb1 | Ydr026c | Gcn4 | Hap5 | Mig1 | Hap4 | Hap3 | Hap1 | Hap2 |
|  |  |  |  |  |  |  |  |  |  |  | Rpn4 |
|  |  |  |  |  |  |  |  |  |  |  | Sut1 |
|  |  |  |  |  |  |  |  |  |  |  | Reb1 |
|  |  |  |  |  |  |  |  |  |  |  | Ydr026c |
|  |  |  |  |  |  |  |  |  |  |  | Gcn4 |
|  |  |  |  |  |  |  |  |  |  |  | Hap5 |
|  |  |  |  |  |  |  |  |  |  |  | Mig1 |
|  |  |  |  |  |  |  |  |  |  |  | Hap4 |
|  |  |  |  |  |  |  |  |  |  |  | Hap3 |
|  |  |  |  |  |  |  |  |  |  |  | Hap1 |
|  |  |  |  |  |  |  |  |  |  |  | Hap2 |
 Rpn4 | Sut1 | Reb1 | Ydr026c | Gcn4 | Hap5 | Mig1 | Hap4 | Hap3 | Hap1 | Hap2 |
