# Supplementary material for: High-resolution analysis of condition-specific regulatory modules in Saccharomyces cerevisiae
Source: Genome Biol. 2008 Jan 3;9(1):R2. doi: 10.1186/gb-2008-9-1-r2 (PMC2395236; doi:10.1186/gb-2008-9-1-r2)
Supplement: Additional data file 11 — Matrices describing all EPMs and RMs, including lists of synergistic pairs of regulators. [file gb-2008-9-1-r2-S11.zip › htmls/C4_EPMs_matrix/EPM_8.GO_enrichment.matrix.html]

|  |  |  |  |
| --- | --- | --- | --- |
| Put3 | Hap4 | Gcn4 | Biological Process |
|  |  |  | P:pyrimidine nucleoside metabolism |
|  |  |  | P:pyrimidine salvage |
|  |  |  | P:cytidine metabolism |
|  |  |  | P:pyrimidine ribonucleoside metabolism |
|  |  |  | P:steroid metabolism |
|  |  |  | P:steroid biosynthesis |
|  |  |  | P:cytosine metabolism |
|  |  |  | P:folic acid and derivative biosynthesis |
|  |  |  | P:isocitrate metabolism |
|  |  |  | P:leucine biosynthesis |
|  |  |  | P:cofactor biosynthesis |
|  |  |  | P:heterocycle metabolism |
|  |  |  | P:heme a metabolism |
|  |  |  | P:heme a biosynthesis |
|  |  |  | P:coenzyme biosynthesis |
|  |  |  | P:group transfer coenzyme metabolism |
|  |  |  | P:oxidative phosphorylation |
|  |  |  | P:metabolism |
|  |  |  | P:coenzyme metabolism |
|  |  |  | P:cellular metabolism |
|  |  |  | P:glutamate biosynthesis |
|  |  |  | P:glutamate metabolism |
|  |  |  | P:main pathways of carbohydrate metabolism |
|  |  |  | P:aspartate family amino acid metabolism |
|  |  |  | P:cofactor metabolism |
|  |  |  | P:generation of precursor metabolites and energy |
|  |  |  | P:cellular biosynthesis |
|  |  |  | P:aspartate family amino acid biosynthesis |
|  |  |  | P:asparagine biosynthesis |
|  |  |  | P:biosynthesis |
|  |  |  | P:nitrogen compound metabolism |
|  |  |  | P:amine metabolism |
|  |  |  | P:amino acid and derivative metabolism |
|  |  |  | P:amino acid metabolism |
|  |  |  | P:nitrogen compound biosynthesis |
|  |  |  | P:amine biosynthesis |
|  |  |  | P:amino acid biosynthesis |
|  |  |  | P:carboxylic acid metabolism |
|  |  |  | P:organic acid metabolism |
|  |  |  | P:glutamine metabolism |
|  |  |  | P:glutamine biosynthesis |
|  |  |  | P:glutamine family amino acid biosynthesis |
|  |  |  | P:glutamine family amino acid metabolism |
|
| Put3 | Hap4 | Gcn4 | Molecular Function |
|  |  |  | F:oxysterol binding |
|  |  |  | F:steroid binding |
|  |  |  | F:cytosine deaminase activity |
|  |  |  | F:acid phosphatase activity |
|  |  |  | F:asparagine synthase (glutamine-hydrolyzing) activity |
|  |  |  | F:oxidoreductase activity, acting on the CH-NH2 group of donors, NAD or NADP as acceptor |
|  |  |  | F:ammonia ligase activity |
|  |  |  | F:glutamate-ammonia ligase activity |
|  |  |  | F:glutamate dehydrogenase (NADP+) activity |
|  |  |  | F:ligase activity, forming carbon-nitrogen bonds |
|  |  |  | F:acid-ammonia (or amide) ligase activity |
|  |  |  | F:isocitrate dehydrogenase activity |
|  |  |  | F:s-methyltransferase activity |
|  |  |  | F:2-isopropylmalate synthase activity |
|  |  |  | F:oxidoreductase activity, acting on NADH or NADPH, heme protein as acceptor |
|  |  |  | F:homocysteine S-methyltransferase activity |
|  |  |  | F:monovalent inorganic cation transporter activity |
|  |  |  | F:ligase activity |
|  |  |  | F:hydrogen ion transporter activity |
|  |  |  | F:ligase activity, forming carbon-carbon bonds |
|  |  |  | F:isocitrate dehydrogenase (NADP+) activity |
|  |  |  | F:saccharopine dehydrogenase activity |
|  |  |  | F:pyruvate carboxylase activity |
|  |  |  | F:saccharopine dehydrogenase (NADP+, L-glutamate-forming) activity |
|  |  |  | F:gTP cyclohydrolase I activity |
|  |  |  | F:gTP cyclohydrolase activity |
|  |  |  | F:catalytic activity |
|  |  |  | F:oxidoreductase activity |
|
| Put3 | Hap4 | Gcn4 | Cellular Component |
|  |  |  | C:cytoplasm |
|  |  |  | C:intracellular |
|  |  |  | C:intracellular part |
|  |  |  | C:proton-transporting ATP synthase, catalytic core |
|  |  |  | C:proton-transporting ATP synthase, catalytic core (sensu Eukaryota) |
|  |  |  | C:nucleoid |
|  |  |  | C:mitochondrial nucleoid |
|
