# Supplementary material for: High-resolution analysis of condition-specific regulatory modules in Saccharomyces cerevisiae
Source: Genome Biol. 2008 Jan 3;9(1):R2. doi: 10.1186/gb-2008-9-1-r2 (PMC2395236; doi:10.1186/gb-2008-9-1-r2)
Supplement: Additional data file 12 — Possible regulatory relationships among transcription factors in the same EPM. [file gb-2008-9-1-r2-S12.pdf]

Additional Data 12. Possible regulatory relationships between regulators in the same EPM

The regulatory relationships are obtained from our prediction (L) and/or ChIP-chip data of Harbison et al.(C).

hs : heat shock, nd : nitrogen depletion, cc : cell cycle condition

condition	EPM number	transcription factor	target transcription factors
hs	EPM#0	RAP1	
hs	EPM#0	ABF1	STB1_(L)
hs	EPM#0	YAP5	
hs	EPM#0	TEC1	STE12_(L)(C),TEC1_(C)
hs	EPM#0	MBP1	STE12_(L),SWI4_(C),ABF1_(C),PDR1_(C)
hs	EPM#0	SWI6	STE12_(L),SWI4_(C),ABF1_(C)
hs	EPM#0	SWI4	STE12_(L),SWI4_(C)
hs	EPM#0	STE12	STE12_(L)(C),TEC1_(C),RAP1_(C)
hs	EPM#0	STB1	
hs	EPM#0	PDR1	
hs	EPM#1	ABF1	
hs	EPM#1	YAP5	
hs	EPM#1	GAT3	
hs	EPM#1	SWI4	SWI4_(C)
hs	EPM#1	SWI6	SWI4_(C),ABF1_(C)
hs	EPM#1	TEC1	TEC1_(C)
hs	EPM#1	MAT1mc	
hs	EPM#1	PDR1	
hs	EPM#2	ABF1	
hs	EPM#2	TEC1	TEC1_(C)
hs	EPM#2	MCM1	SWI4_(C)
hs	EPM#2	SWI4	SWI4_(C)
hs	EPM#2	SWI6	SWI4_(C),ABF1_(C)
hs	EPM#4	FHL1	
hs	EPM#4	RAP1	
hs	EPM#4	SFP1	
hs	EPM#4	LEU3	YAP6_(L)
hs	EPM#4	MBP1	STE12_(L),SWI4_(C),ABF1_(C)
hs	EPM#4	ABF1	STB1_(L),PHO2_(C),PUT3_(C),FHL1_(C)
hs	EPM#4	MCM1	SWI4_(C)
hs	EPM#4	TEC1	STE12_(L)(C),TEC1_(C)
hs	EPM#4	MAC1	
hs	EPM#4	RCS1	RCS1_(C)
hs	EPM#4	SWI4	STE12_(L),SWI4_(C),NDD1_(C)
hs	EPM#4	SWI6	STE12_(L),SWI4_(C),ABF1_(C),NDD1_(C)
hs	EPM#4	STE12	STE12_(L)(C),TEC1_(C),RAP1_(C)
hs	EPM#4	NDD1	
hs	EPM#4	REB1	
hs	EPM#4	YAP6	YAP6_(L)(C)
hs	EPM#4	PUT3	YAP6_(L),RCS1_(C)
hs	EPM#4	AFT2	RCS1_(C)
hs	EPM#4	PHO2	YAP6_(L)
hs	EPM#4	AZF1	YAP6_(C)
hs	EPM#4	STE11	
hs	EPM#4	STB1	NDD1_(C)
hs	EPM#4	RLR1	
hs	EPM#4	HSF1	REB1_(C)
hs	EPM#4	SKN7	YAP6_(L),AFT2_(C)
hs	EPM#5	FHL1	
hs	EPM#5	MCM1	
hs	EPM#5	YAP1	
hs	EPM#6	MBP1	PDR1_(C)
hs	EPM#6	HIR1	
hs	EPM#6	HIR3	
hs	EPM#6	HIR2	

hs	EPM#6	SFP1	
hs	EPM#6	HSF1	
hs	EPM#6	PDR1	
hs	EPM#7	FHL1	
hs	EPM#7	RAP1	
hs	EPM#7	SFP1	
hs	EPM#7	MBP1	STE12_(L),SWI4_(C)
hs	EPM#7	SWI4	STE12_(L),SWI4_(C)
hs	EPM#7	SWI6	STE12_(L),SWI4_(C)
hs	EPM#7	TEC1	STE12_(L)(C),TEC1_(C)
hs	EPM#7	STE12	STE12_(L)(C),TEC1_(C),RAP1_(C)
hs	EPM#7	PHO2	
hs	EPM#7	AZF1	
hs	EPM#7	REB1	
hs	EPM#7	SKN7	
hs	EPM#7	STB1	
hs	EPM#8	MBP1	SWI4_(C)
hs	EPM#8	SWI6	SWI4_(C)
hs	EPM#8	SWI4	SWI4_(C)
hs	EPM#8	AZF1	
hs	EPM#8	STB1	
hs	EPM#9	MBP1	SWI4_(C)
hs	EPM#9	SWI4	SWI4_(C)
hs	EPM#9	SWI6	SWI4_(C)
hs	EPM#9	STB1	
hs	EPM#10	UME6	MSN4_(L)
hs	EPM#10	MSN2	MSN4_(L)(C),RPN4_(L),YAP1_(C)
hs	EPM#10	SKN7	MSN4_(L)(C),RPN4_(L),YAP1_(C),MSN2_(C)
hs	EPM#10	RPN4	PUT3_(C),YAP1_(C)
hs	EPM#10	SWI5	
hs	EPM#10	SWI6	
hs	EPM#10	PHO2	MSN4_(L)
hs	EPM#10	YAP1	RPN4_(C)
hs	EPM#10	ACE2	YAP1_(C)
hs	EPM#10	MSN4	MSN4_(L),RPN4_(L)(C),YAP1_(C)
hs	EPM#10	SUT1	MSN4_(L)(C),RPN4_(L),SUT1_(C),MSN2_(C)
hs	EPM#10	STP1	MSN4_(L)
hs	EPM#10	SNT2	
hs	EPM#10	LEU3	MSN4_(L)
hs	EPM#10	PUT3	MSN4_(L),RPN4_(L)
hs	EPM#10	GAL4	MSN4_(L),RPN4_(L)
hs	EPM#11	HSF1	RPN4_(L)(C),INO2_(C)
hs	EPM#11	MSN2	MSN4_(L)(C),HAP4_(L),YAP6_(L),HAP2_(L),RPN4_(L)
hs	EPM#11	NRG1	YAP6_(L)(C),MSN4_(L),HAP4_(L)(C),NRG1_(C),PDR1_(C)
hs	EPM#11	SKN7	SOK2_(L)(C),MSN4_(L)(C),HAP4_(L)(C),HAP2_(L),YAP6_(L),<br>RPN4_(L),NRG1_(C),ROX1_(C),MSN2_(C),AFT2_(C)
hs	EPM#11	UME6	MSN4_(L),HAP4_(L),YAP6_(L)
hs	EPM#11	HAP1	HAP1_(C),MSN2_(C),ROX1_(C)
hs	EPM#11	AFT2	
hs	EPM#11	SKO1	
hs	EPM#11	PUT3	HAP4_(L),YAP6_(L),MSN4_(L),RPN4_(L)
hs	EPM#11	SOK2	MSN4_(L)(C),HAP4_(L)(C),YAP6_(L)(C)
hs	EPM#11	SUT1	MSN4_(L)(C),HAP4_(L)(C),YAP6_(L),HAP2_(L),RPN4_(L),SUT1_(C),<br>SOK2_(C),MSN2_(C)
hs	EPM#11	CIN5	YAP6_(L)(C),ROX1_(C),CIN5_(C)
hs	EPM#11	MSN4	MSN4_(L),HAP4_(L),YAP6_(L),HAP2_(L),RPN4_(L)(C),ROX1_(C)
hs	EPM#11	FKH2	YAP6_(L)(C),ACE2_(C),SUT1_(C)
hs	EPM#11	YAP6	YAP6_(L)(C),DAL81_(C),ROX1_(C),RDS1_(C),CIN5_(C)
hs	EPM#11	ROX1	YAP6_(L)(C),HAP4_(L)(C),PDR1_(C),ROX1_(C)
hs	EPM#11	ACE2	
hs	EPM#11	MBP1	PDR1_(C)
hs	EPM#11	STP1	MSN4_(L),HAP4_(L),YAP6_(L)
hs	EPM#11	INO2	MSN4_(L),HAP4_(L),YAP6_(L),RPN4_(L)

hs	EPM#11	PDR3	MSN4_(L),HAP4_(L),YAP6_(L),RPN4_(L),PDR3_(C)
hs	EPM#11	PDR1	MSN4_(L),HAP4_(L),YAP6_(L),RPN4_(L)
hs	EPM#11	RGT1	MSN4_(L),HAP4_(L),YAP6_(L),RPN4_(L)
hs	EPM#11	MIG1	MSN4_(L),HAP4_(L),YAP6_(L)
hs	EPM#11	LEU3	MSN4_(L),HAP4_(L),YAP6_(L)
hs	EPM#11	PHO2	MSN4_(L),HAP4_(L),HAP2_(L),YAP6_(L)
hs	EPM#11	PHD1	MSN4_(L)(C),HAP4_(L)(C),HAP2_(L),SOK2_(C)
hs	EPM#11	RPH1	HAP4_(L),MSN4_(L)
hs	EPM#11	RDS1	HAP4_(L),RDS1_(C)
hs	EPM#11	ADR1	HAP4_(L),PUT3_(C)
hs	EPM#11	UGA3	HAP4_(L),YAP6_(L),MSN4_(L)
hs	EPM#11	GAL80	YAP6_(L),MSN4_(L),HAP4_(L)
hs	EPM#11	SNT2	HAP2_(L)
hs	EPM#11	GAL4	YAP6_(L),MSN4_(L),HAP4_(L),RPN4_(L),GAL80_(C)
hs	EPM#11	DAL81	UGA3_(C)
hs	EPM#11	RPN4	PDR1_(C),PUT3_(C)
hs	EPM#11	BAS1	MSN4_(L)
hs	EPM#11	RLM1	
hs	EPM#11	MCM1	HAP4_(L),ACE2_(C)
hs	EPM#11	HAP2	UGA3_(C)
hs	EPM#11	HAP4	PUT3_(C)
hs	EPM#11	HAP3	UGA3_(C)
hs	EPM#12	UME6	
hs	EPM#12	AFT2	
hs	EPM#12	RPN4	
hs	EPM#13	PUT3	
hs	EPM#13	HSF1	
hs	EPM#13	GCN4	PUT3_(C)
hs	EPM#14	HSF1	INO2_(C)
hs	EPM#14	NRG1	MSN4_(L),NRG1_(C),PDR1_(C)
hs	EPM#14	MSN2	MSN4_(L)(C)
hs	EPM#14	AFT2	
hs	EPM#14	PUT3	MSN4_(L)
hs	EPM#14	GCN4	PUT3_(C)
hs	EPM#14	ROX1	PDR1_(C),ROX1_(C)
hs	EPM#14	MSN4	MSN4_(L),ROX1_(C)
hs	EPM#14	SUT1	MSN4_(L)(C),SUT1_(C),MSN2_(C)
hs	EPM#14	SKN7	MSN4_(L)(C),NRG1_(C),ROX1_(C),MSN2_(C),AFT2_(C)
hs	EPM#14	PDR3	MSN4_(L),PDR3_(C)
hs	EPM#14	PDR1	MSN4_(L)
hs	EPM#14	STP1	MSN4_(L)
hs	EPM#14	UME6	MSN4_(L)
hs	EPM#14	PHO2	MSN4_(L)
hs	EPM#14	MIG1	MSN4_(L)
hs	EPM#14	RGT1	MSN4_(L)
hs	EPM#14	INO2	MSN4_(L)
hs	EPM#14	GAL4	MSN4_(L)
cc	EPM#0	FHL1	GCN4_(L)(C)
cc	EPM#0	RAP1	GCN4_(L),MSN4_(C)
cc	EPM#0	HAP1	HAP1_(C)
cc	EPM#0	PDR1	
cc	EPM#0	YAP5	
cc	EPM#0	GAT3	
cc	EPM#0	GCR2	
cc	EPM#0	GLN3	UGA3_(C),GCN4_(C)
cc	EPM#0	SFP1	GCN4_(L)
cc	EPM#0	MSN4	RPN4_(C)
cc	EPM#0	CIN5	CIN5_(C)
cc	EPM#0	UGA3	
cc	EPM#0	SKN7	MSN4_(C)
cc	EPM#0	PUT3	
cc	EPM#0	MCM1	SWI4_(L)(C),SWI5_(L)(C)
cc	EPM#0	SWI4	SWI4_(L)(C),HAP1_(C),NDD1_(C)

cc	EPM#0	SWI6	SWI4_(L)(C),ABF1_(C),NDD1_(C)
cc	EPM#0	YDR026c	
cc	EPM#0	GCR1	
cc	EPM#0	ABF1	PUT3_(C),FHL1_(C)
cc	EPM#0	PHO4	
cc	EPM#0	GCN4	UGA3_(C),PUT3_(C),GLN3_(C)
cc	EPM#0	MBP1	SWI4_(L)(C),ABF1_(C),PDR1_(C)
cc	EPM#0	HSF1	RPN4_(C)
cc	EPM#0	UME1	
cc	EPM#0	SUT1	SUT1_(C),MSN4_(C)
cc	EPM#0	SWI5	GAT3_(C)
cc	EPM#0	NDD1	SWI5_(L)(C)
cc	EPM#0	RDS1	RDS1_(C)
cc	EPM#0	RPN4	PDR1_(C),PUT3_(C)
cc	EPM#1	MBP1	SWI4_(L)(C)
cc	EPM#1	SWI6	SWI4_(L)(C)
cc	EPM#1	SWI4	SWI4_(L)(C)
cc	EPM#1	STB1	SWI4_(L)
cc	EPM#1	DIG1	STE12_(C)
cc	EPM#1	STE12	STE12_(C),DIG1_(C)
cc	EPM#1	MCM1	SWI4_(L)(C)
cc	EPM#2	NRG1	NRG1_(C)
cc	EPM#3	HSF1	
cc	EPM#4	FHL1	
cc	EPM#4	RAP1	
cc	EPM#4	SFP1	
cc	EPM#4	YAP5	
cc	EPM#5	FKH2	FKH1_(C)
cc	EPM#5	NDD1	
cc	EPM#5	MCM1	
cc	EPM#5	FKH1	
cc	EPM#5	ZAP1	ZAP1_(C)
cc	EPM#6	FKH2	
cc	EPM#6	NDD1	
cc	EPM#6	MCM1	
cc	EPM#6	NRG1	NRG1_(C)
cc	EPM#7	HAP1	HAP1_(C)
cc	EPM#7	HAP4	
cc	EPM#7	RPN4	REB1_(C)
cc	EPM#7	HAP2	GCN4_(C)
cc	EPM#7	HAP5	
cc	EPM#7	GCN4	
cc	EPM#7	REB1	
cc	EPM#7	YDR026c	
cc	EPM#7	SUT1	SUT1_(C),HAP4_(C)
cc	EPM#7	HAP3	
cc	EPM#7	MIG1	
cc	EPM#8	HAP4	PUT3_(C)
cc	EPM#8	GCN4	PUT3_(C)
cc	EPM#8	PUT3	
nd	EPM#0	STE12	TEC1_(L)(C),YHP1_(L),STE12_(L)(C),TYE7_(C),DIG1_(C)
nd	EPM#0	MBP1	YHP1_(L)(C),TEC1_(L),SWI4_(L)(C),ABF1_(C),YOX1_(C),PDR1_(C)
nd	EPM#0	SWI6	YHP1_(L)(C),TEC1_(L),STE12_(L),SWI4_(L)(C),ABF1_(C),YOX1_(C), TYE7_(C),NDD1_(C)
nd	EPM#0	FKH2	YHP1_(L)(C),FKH1_(C)
nd	EPM#0	DIG1	TEC1_(L)(C),YHP1_(L),STE12_(L)(C),TYE7_(C)
nd	EPM#0	SWI4	YHP1_(L)(C),TEC1_(L),STE12_(L),SWI4_(L)(C),YOX1_(C),TYE7_(C), NDD1_(C)
nd	EPM#0	FKH1	YHP1_(L)(C),TEC1_(L),STE12_(L)(C)
nd	EPM#0	MCM1	YHP1_(L)(C),TEC1_(L),STE12_(L),SWI4_(L)(C)
nd	EPM#0	GCN4	RTG3_(C),LEU3_(C),STB1_(C)
nd	EPM#0	TYE7	RDS1_(L)
nd	EPM#0	TEC1	TEC1_(L)(C),STE12_(L)(C)

nd	EPM#0	NDD1	YHP1_(L)(C),TEC1_(L),SWI4_(L)
nd	EPM#0	INO4	
nd	EPM#0	RLM1	TEC1_(L),STE12_(L)
nd	EPM#0	CBF1	RDS1_(L)(C),REB1_(C),SWI4_(C),CBF1_(C),TYE7_(C)
nd	EPM#0	MET32	
nd	EPM#0	XBP1	TEC1_(L),YHP1_(L)
nd	EPM#0	STB1	YHP1_(L),TEC1_(L),STE12_(L),NDD1_(C)
nd	EPM#0	HSF1	REB1_(C),TYE7_(C)
nd	EPM#0	ABF1	STE12_(L),SWI4_(L)
nd	EPM#0	RTG3	
nd	EPM#0	YHP1	
nd	EPM#0	YOX1	YHP1_(L)
nd	EPM#0	GCR1	
nd	EPM#0	REB1	
nd	EPM#0	AZF1	YHP1_(L)
nd	EPM#0	LEU3	YHP1_(L)
nd	EPM#0	RDS1	TEC1_(L),YHP1_(L),RDS1_(C)
nd	EPM#0	RLR1	YHP1_(L)
nd	EPM#0	PDR1	YHP1_(L)
nd	EPM#1	DIG1	STE12_(L)(C),TYE7_(C)
nd	EPM#1	GCN4	
nd	EPM#1	SWI6	STE12_(L),ABF1_(C),TYE7_(C)
nd	EPM#1	MET32	
nd	EPM#1	CBF1	REB1_(C),CBF1_(C),TYE7_(C)
nd	EPM#1	XBP1	
nd	EPM#1	ABF1	STE12_(L)
nd	EPM#1	BAS1	
nd	EPM#1	TYE7	
nd	EPM#1	STE12	STE12_(L)(C),TYE7_(C),DIG1_(C)
nd	EPM#1	REB1	
nd	EPM#2	STE12	STE12_(L)(C),TYE7_(C)
nd	EPM#2	GCN4	UGA3_(L)(C),MET4_(C),LEU3_(C)
nd	EPM#2	MBP1	ABF1_(C)
nd	EPM#2	MET32	
nd	EPM#2	BAS1	
nd	EPM#2	MET4	
nd	EPM#2	CBF1	CBF1_(C),MET4_(C),TYE7_(C)
nd	EPM#2	ABF1	STE12_(L)
nd	EPM#2	LEU3	MET4_(C)
nd	EPM#2	TYE7	
nd	EPM#2	UGA3	TYE7_(L)
nd	EPM#2	PHO4	TYE7_(L)
nd	EPM#3	STE12	STE12_(L)(C)
nd	EPM#3	INO4	
nd	EPM#3	NDD1	
nd	EPM#3	XBP1	
nd	EPM#3	ABF1	STE12_(L)
nd	EPM#3	GCR1	
nd	EPM#4	STE12	SWI5_(L)(C),STE12_(L)(C),DIG1_(C)
nd	EPM#4	MCM1	SWI5_(L)(C),STE12_(L),SWI4_(L)(C)
nd	EPM#4	SWI4	SWI5_(L),STE12_(L),SWI4_(L)(C),NDD1_(C)
nd	EPM#4	SWI5	
nd	EPM#4	MBP1	SWI5_(L),SWI4_(L)(C),ABF1_(C)
nd	EPM#4	SWI6	SWI5_(L),STE12_(L),SWI4_(L)(C),ABF1_(C),NDD1_(C)
nd	EPM#4	NDD1	SWI5_(L)(C),SWI4_(L)
nd	EPM#4	DIG1	SWI5_(L),STE12_(L)(C)
nd	EPM#4	ABF1	STE12_(L),SWI4_(L)
nd	EPM#4	GCN4	
nd	EPM#5	SWI5	
nd	EPM#5	MSN4	MSN4_(L)
nd	EPM#5	MSN2	MSN4_(L)(C)
nd	EPM#5	SKN7	MSN4_(L)(C),MSN2_(C)
nd	EPM#5	UME1	

nd	EPM#6	STE12	TEC1_(L)(C),SWI5_(L)(C),STE12_(L)(C),ACE2_(L),SUM1_(C), DIG1_(C)
nd	EPM#6	SWI4	TEC1_(L),SWI5_(L),STE12_(L),ACE2_(L),SWI4_(L)(C),NDD1_(C)
nd	EPM#6	MCM1	TEC1_(L),SWI5_(L)(C),ACE2_(L)(C),STE12_(L),SWI4_(L)(C)
nd	EPM#6	SWI6	TEC1_(L),SWI5_(L),STE12_(L),ACE2_(L),SWI4_(L)(C),ABF1_(C), NDD1_(C)
nd	EPM#6	FKH2	SWI5_(L)(C),ACE2_(L)(C),FKH1_(C)
nd	EPM#6	DIG1	TEC1_(L)(C),SWI5_(L),STE12_(L)(C)
nd	EPM#6	MBP1	TEC1_(L),SWI5_(L),SWI4_(L)(C),ABF1_(C)
nd	EPM#6	FKH1	SWI5_(L),ACE2_(L)(C),TEC1_(L),STE12_(L)(C)
nd	EPM#6	INO4	SWI5_(L)(C)
nd	EPM#6	NDD1	SWI5_(L)(C),ACE2_(L)(C),TEC1_(L),SWI4_(L)
nd	EPM#6	TEC1	TEC1_(L)(C),STE12_(L)(C)
nd	EPM#6	ACE2	
nd	EPM#6	RLM1	TEC1_(L),STE12_(L)
nd	EPM#6	SWI5	
nd	EPM#6	GCN4	STB1_(C)
nd	EPM#6	SUM1	SUM1_(C)
nd	EPM#6	XBP1	TEC1_(L),SWI5_(L),ACE2_(L)
nd	EPM#6	STB1	TEC1_(L),SWI5_(L),STE12_(L),ACE2_(L),NDD1_(C)
nd	EPM#6	GCR1	SWI5_(L)
nd	EPM#6	ABF1	STE12_(L),SWI4_(L)
nd	EPM#6	STB5	STB5_(C)
nd	EPM#6	SIG1	
nd	EPM#6	HSF1	
nd	EPM#7	RPN4	
nd	EPM#8	GAT3	
nd	EPM#8	YAP5	
nd	EPM#8	DAT1	
nd	EPM#8	GZF3	
nd	EPM#8	PDR1	
nd	EPM#8	UGA3	
nd	EPM#8	YAP6	YAP6_(C),RDS1_(C)
nd	EPM#8	XBP1	TEC1_(L),HAP1_(L)
nd	EPM#8	MSN4	XBP1_(L),MSN4_(L)
nd	EPM#8	HAP4	
nd	EPM#8	HAP1	TEC1_(C),HAP1_(C)
nd	EPM#8	MAL33	XBP1_(C)
nd	EPM#8	GCN4	UGA3_(L)(C),GAT1_(C)
nd	EPM#8	MCM1	TEC1_(L),SWI4_(L)(C)
nd	EPM#8	GAT1	UGA3_(L),GZF3_(C)
nd	EPM#8	DAL82	UGA3_(C),GAT1_(C)
nd	EPM#8	ABF1	SWI4_(L),STP1_(C)
nd	EPM#8	RDS1	TEC1_(L),RDS1_(C)
nd	EPM#8	NDD1	TEC1_(L),SWI4_(L)
nd	EPM#8	UME1	
nd	EPM#8	SWI6	TEC1_(L),SWI4_(L)(C),HAP1_(L),ABF1_(C),NDD1_(C)
nd	EPM#8	SWI4	TEC1_(L),SWI4_(L)(C),HAP1_(L)(C),NDD1_(C)
nd	EPM#8	TEC1	TEC1_(L)(C),STP1_(C)
nd	EPM#8	STP1	XBP1_(L),YAP5_(L),MSN4_(L)
nd	EPM#8	PDR3	XBP1_(L),PDR3_(C)
nd	EPM#9	GAT3	
nd	EPM#9	YAP5	
nd	EPM#9	RAP1	GAT1_(C)
nd	EPM#9	PHD1	STB4_(C),STE12_(C)
nd	EPM#9	ABF1	STE12_(L),SWI4_(L),STP1_(C)
nd	EPM#9	RDS1	TEC1_(L),RDS1_(C)
nd	EPM#9	DAL82	GAT1_(C)
nd	EPM#9	MCM1	TEC1_(L),STE12_(L),SWI4_(L)(C)
nd	EPM#9	GAT1	STB4_(C),GZF3_(C)
nd	EPM#9	FKH1	TEC1_(L),STE12_(L)(C),UME1_(C)
nd	EPM#9	RCS1	RCS1_(C),STB4_(C)
nd	EPM#9	PDR1	

nd	EPM#9	GCN4	GAT1_(C),STB4_(C)
nd	EPM#9	GZF3	
nd	EPM#9	STB4	
nd	EPM#9	PDR3	PDR3_(C)
nd	EPM#9	UME1	
nd	EPM#9	MAT1mc	
nd	EPM#9	TEC1	TEC1_(L)(C),STE12_(L)(C),STP1_(C)
nd	EPM#9	NDD1	TEC1_(L),SWI4_(L)
nd	EPM#9	STP1	YAP5_(L),STB4_(C)
nd	EPM#9	SWI4	TEC1_(L),STE12_(L),SWI4_(L)(C),HAP1_(L)(C),NDD1_(C)
nd	EPM#9	SWI6	TEC1_(L),STE12_(L),SWI4_(L)(C),HAP1_(L),ABF1_(C),NDD1_(C)
nd	EPM#9	HAP1	TEC1_(C),HAP1_(C)
nd	EPM#9	RPH1	
nd	EPM#9	STE12	TEC1_(L)(C),STE12_(L)(C),RAP1_(C),DIG1_(C)
nd	EPM#9	DIG1	TEC1_(L)(C),STE12_(L)(C),STP1_(C)
nd	EPM#9	GAL4	
nd	EPM#9	PHO4	
nd	EPM#9	FKH2	GAT1_(C),FKH1_(C)
nd	EPM#11	FHL1	
nd	EPM#11	RAP1	
nd	EPM#11	SFP1	
nd	EPM#11	PDR1	
nd	EPM#11	YAP5	AFT2_(L)
nd	EPM#11	SMP1	SMP1_(C)
nd	EPM#11	AFT2	AFT2_(L)
nd	EPM#11	SKN7	AFT2_(L)(C)
nd	EPM#11	SUT1	SUT1_(C)
nd	EPM#12	FHL1	
nd	EPM#12	RAP1	
nd	EPM#12	SFP1	
nd	EPM#12	SMP1	SMP1_(C)
nd	EPM#12	YAP5	
nd	EPM#12	RME1	
nd	EPM#12	PDR1	
nd	EPM#12	GCR2	
nd	EPM#12	ROX1	PDR1_(L)(C),ROX1_(C)
nd	EPM#12	NDD1	
nd	EPM#13	HSF1	REB1_(C),MGA1_(C)
nd	EPM#13	ROX1	YAP6_(C),HAP4_(C),ROX1_(C)
nd	EPM#13	MSN2	XBP1_(L),NRG1_(L),YAP1_(L)(C),MSN4_(L)(C),MGA1_(C)
nd	EPM#13	SUT1	RIM101_(L)(C),MSN4_(L)(C),MOT3_(L)(C),SUT1_(C),MGA1_(C), HAP4_(C),SOK2_(C),MSN2_(C)
nd	EPM#13	SOK2	YAP6_(C),MGA1_(C),MSN4_(C),HAP4_(C)
nd	EPM#13	SKN7	NRG1_(L)(C),YAP1_(L)(C),AFT2_(L)(C),MSN4_(L)(C),MGA1_(C), SOK2_(C),ROX1_(C),GAT1_(C),HAP4_(C),MSN2_(C),MOT3_(C)
nd	EPM#13	NRG1	NRG1_(L)(C),XBP1_(L),YAP6_(C),MGA1_(C),HAP4_(C),MOT3_(C), YAP7_(C)
nd	EPM#13	YAP7	AFT2_(L),YAP1_(L),YAP7_(C)
nd	EPM#13	CIN5	XBP1_(L),MGA1_(L)(C),YAP6_(C),ROX1_(C),CIN5_(C)
nd	EPM#13	THI2	
nd	EPM#13	AFT2	XBP1_(L)(C),NRG1_(L),YAP1_(L),AFT2_(L),RCS1_(C)
nd	EPM#13	PHD1	RIM101_(L),MSN4_(L)(C),HAP4_(C),SOK2_(C),MGA1_(C)
nd	EPM#13	RIM101	MGA1_(C),ROX1_(C)
nd	EPM#13	PUT3	RIM101_(L),MSN4_(L),RCS1_(C)
nd	EPM#13	GCN4	GLN3_(L)(C),PUT3_(C),RTG3_(C),GAT1_(C),RIM101_(C)
nd	EPM#13	SWI6	YAP7_(L),SOK2_(C),MOT3_(C)
nd	EPM#13	MOT3	
nd	EPM#13	MCM1	YAP7_(L)
nd	EPM#13	MGA1	
nd	EPM#13	XBP1	YAP7_(L),MGA1_(C)
nd	EPM#13	YAP6	AFT2_(L),YAP6_(C),MGA1_(C),ROX1_(C),CIN5_(C)
nd	EPM#13	DAL80	
nd	EPM#13	RCS1	XBP1_(L),SIP4_(L),GLN3_(L)(C),RCS1_(C),HAP2_(C)

nd	EPM#13	RTG3	
nd	EPM#13	STP1	XBP1_(L),NRG1_(L),YAP1_(L),AFT2_(L),SIP4_(L),YAP5_(L),MSN4_(L),
nd	EPM#13	REB1	
nd	EPM#13	MSN4	XBP1_(L),NRG1_(L),MSN4_(L),YAP1_(L)(C),ROX1_(C)
nd	EPM#13	UME6	XBP1_(L),NRG1_(L),YAP1_(L),AFT2_(L),RIM101_(L),SIP4_(L)(C),<br>YAP5_(L),MSN4_(L),MOT3_(L),GAT1_(C)
nd	EPM#13	SIP4	SIP4_(L)
nd	EPM#13	ARR1	AFT2_(L)
nd	EPM#13	YAP3	AFT2_(L)
nd	EPM#13	YAP5	AFT2_(L)
nd	EPM#13	CAD1	AFT2_(L),YAP1_(L)
nd	EPM#13	GZF3	YAP1_(L)
nd	EPM#13	GAT1	GLN3_(L),GZF3_(C)
nd	EPM#13	GLN3	MSN4_(L),GCN4_(C),GAT1_(C)
nd	EPM#13	GAL80	
nd	EPM#13	PDR3	XBP1_(L),PDR3_(C)
nd	EPM#13	SPT2	XBP1_(L),RGT1_(C)
nd	EPM#13	HAP2	GCN4_(L)(C),GAT1_(C)
nd	EPM#13	HAP3	
nd	EPM#13	HAP4	PUT3_(C)
nd	EPM#13	YAP1	YAP6_(C),ROX1_(C),AFT2_(C)
nd	EPM#13	GAL4	XBP1_(L),NRG1_(L),GAL80_(C)
nd	EPM#13	RGT1	NRG1_(L),YAP1_(L),AFT2_(L)
nd	EPM#14	ROX1	YAP6_(C),HAP4_(C),ROX1_(C)
nd	EPM#14	YAP6	YAP6_(C),ROX1_(C)
nd	EPM#14	RIM101	ROX1_(C)
nd	EPM#14	UME6	RIM101_(L),SIP4_(L)(C)
nd	EPM#14	HAP4	
nd	EPM#14	HAP1	HAP1_(C),ROX1_(C)
nd	EPM#14	RAP1	SIP4_(L)(C)
nd	EPM#14	SIP4	SIP4_(L)
nd	EPM#14	RCS1	SIP4_(L),RCS1_(C)
nd	EPM#14	STP1	SIP4_(L)
nd	EPM#14	MCM1	
nd	EPM#15	DAL80	
nd	EPM#15	GZF3	
nd	EPM#15	ROX1	YAP6_(C),HAP4_(C),ROX1_(C)
nd	EPM#15	DAT1	
nd	EPM#15	YAP6	YAP6_(C),ROX1_(C)
nd	EPM#15	YAP5	
nd	EPM#15	NRG1	NRG1_(L)(C),YAP6_(C),GAT3_(C),HAP4_(C)
nd	EPM#15	PHD1	MSN4_(L)(C),HAP4_(C)
nd	EPM#15	RCS1	RCS1_(C),HAP2_(C)
nd	EPM#15	GAT3	
nd	EPM#15	SWI5	GAT3_(C)
nd	EPM#15	MSN4	NRG1_(L),MSN4_(L),ROX1_(C)
nd	EPM#15	UME6	NRG1_(L),YAP5_(L),MSN4_(L),GAT3_(C)
nd	EPM#15	CAD1	
nd	EPM#15	MCM1	SWI5_(L)(C)
nd	EPM#15	RAP1	MSN4_(C)
nd	EPM#15	HAP2	
nd	EPM#15	HAP3	
nd	EPM#15	HAP4	
nd	EPM#15	HAP1	HAP1_(C),ROX1_(C)
nd	EPM#16	AFT2	NRG1_(L),AFT2_(L)
nd	EPM#16	NRG1	NRG1_(L)(C)
nd	EPM#17	HSF1	
nd	EPM#17	AFT2	AFT2_(L)
nd	EPM#17	MSN2	MSN4_(L)(C)
nd	EPM#17	YAP7	AFT2_(L),YAP7_(C)
nd	EPM#17	SKN7	AFT2_(L)(C),MSN4_(L)(C),MSN2_(C)
nd	EPM#17	UME6	AFT2_(L),MSN4_(L)
nd	EPM#17	AZF1	

nd	EPM#17	PHD1	MSN4_(L)(C)
nd	EPM#17	MSN4	MSN4_(L)
nd	EPM#17	RPH1	MSN4_(L)
nd	EPM#17	STP1	AFT2_(L),MSN4_(L)
nd	EPM#17	PUT3	MSN4_(L)
nd	EPM#17	GLN3	MSN4_(L)
nd	EPM#17	CAD1	AFT2_(L)
nd	EPM#17	SUT1	MSN4_(L)(C),SUT1_(C),MSN2_(C)
nd	EPM#17	GAL4	
nd	EPM#19	HSF1	
nd	EPM#20	HSF1	
nd	EPM#20	CIN5	CIN5_(C)
nd	EPM#21	MSN2	MSN4_(L)(C)
nd	EPM#21	MSN4	MSN4_(L)
nd	EPM#21	SKN7	MSN4_(L)(C),MSN2_(C)
nd	EPM#22	CIN5	CIN5_(C)
nd	EPM#22	HSF1	
nd	EPM#22	AFT2	AFT2_(L)
nd	EPM#22	MSN2	MSN4_(L)(C)
nd	EPM#22	PUT3	MSN4_(L)
nd	EPM#22	SUT1	MSN4_(L)(C),SUT1_(C),MSN2_(C)
nd	EPM#22	MSN4	MSN4_(L)
nd	EPM#22	RLM1	
nd	EPM#22	GLN3	MSN4_(L),GAT1_(C)
nd	EPM#22	UME6	AFT2_(L),MSN4_(L),GAT1_(C)
nd	EPM#22	GAT1	GLN3_(L)
nd	EPM#22	DAL82	GAT1_(C)
nd	EPM#22	STP1	AFT2_(L),MSN4_(L)
nd	EPM#22	PHO2	
nd	EPM#22	YAP7	AFT2_(L),YAP7_(C)
nd	EPM#23	CIN5	CIN5_(C)
nd	EPM#23	STP1	
nd	EPM#23	SUT1	SUT1_(C)
nd	EPM#23	UME6	
nd	EPM#24	CIN5	YAP6_(C),CIN5_(C)
nd	EPM#24	SKN7	MSN4_(L)(C),SOK2_(C),HAP4_(C),MSN2_(C)
nd	EPM#24	SOK2	YAP6_(C),MSN4_(C),HAP4_(C)
nd	EPM#24	HAP2	
nd	EPM#24	HAP4	
nd	EPM#24	ASH1	
nd	EPM#24	YAP6	YAP6_(C),CIN5_(C)
nd	EPM#24	MSN2	MSN4_(L)(C)
nd	EPM#24	MSN4	MSN4_(L)
nd	EPM#24	GAL80	
nd	EPM#24	PHO2	
nd	EPM#24	PDR3	PDR3_(C)
nd	EPM#24	SUT1	MSN4_(L)(C),SUT1_(C),HAP4_(C),SOK2_(C),MSN2_(C)
nd	EPM#24	RLM1	
nd	EPM#24	PDR1	
nd	EPM#24	MIG1	
nd	EPM#24	MCM1	ASH1_(L)
nd	EPM#25	HAP1	HAP1_(C)
nd	EPM#25	SUT1	SUT1_(C)
nd	EPM#25	GAL4	
nd	EPM#25	GAT1	UGA3_(L)
nd	EPM#25	UGA3	
nd	EPM#25	PHO4	
