# Supplementary material for: High-resolution analysis of condition-specific regulatory modules in Saccharomyces cerevisiae
Source: Genome Biol. 2008 Jan 3;9(1):R2. doi: 10.1186/gb-2008-9-1-r2 (PMC2395236; doi:10.1186/gb-2008-9-1-r2)
Supplement: Additional data file 13 — Detailed descriptions of RMs in nitrogen depletion EPM 12 and a comparison with the modules determined using other algorithms. [file gb-2008-9-1-r2-S13.pdf]

# Additional data 13. Comparing with other methods in ribosome biogenesis and assembly

## 1. Comparing Enrichment levels of ribosome biogenesis and assembly

| | | | EPM | RM | GRAM | COGRIM (B-/C+) | COGRIM (B+/C+) |
|---|---|---|---|---|---|---|---|
| ribosome biogenesis and assembly | | | 8.7 | 5.21 | 4.87 | 6.02 | - |
| | ribosomal large subunit biogenesis and assembly | | 3.64 | 3.52 | 0.58 | 2.72 | - |
| | | ribosomal large subunit assembly and maintenance | 5.78 | 4.71 | 3.5 | 2.21 | 5.05 |
| | ribosomal small subunit biogenesis and assembly | | 3.47 | 2.63 | 2.66 | 2.63 | - |
| | | ribosomal small subunit assembly and maintenance | 4.9 | 5.14 | 3.2 | 3.26 | 4.97 |
| | ribosome assembly | | 7.55 | 5.89 | 3.82 | 3.09 | 4.33 |
| | | ribosomal subunit assembly | 8.04 | 5.64 | 4.55 | 3.06 | 9.24 |
| | ribosome export from nucleus | | 4.58 | 3.04 | 3.4 | 2.26 | 2.9 |
| | | ribosome large subunit export from nucleus | - | - | 3.27 | 2.63 | 3.77 |
| | | ribosome small subunit export from nucleus | 3.14 | 2.65 | 2.17 | - | 2.82 |
| | rRNA processing | | 12.72 | 4.89 | 4.44 | 4.06 | 5.4 |
| | | processing of 20S pre-rRNA | 6.64 | 2.47 | 5.29 | 5.09 | 3.98 |
| | | 35S primary transcript processing | 4.88 | 2.18 | 3.04 | - | - |
| | | processing of 27S pre-rRNA | 3.45 | 3.11 | 3.88 | 2.95 | - |

**Table A13-1. Enrichment levels of the modules related to ribosome biogenesis and assembly**
We investigated the enrichment levels of the modules related to ribosome biogenesis and assembly. The enrichment level is calculated by negative log of the p-value. The hyphen-minus indicate that the module is not significantly enriched in the category. Our EPMs and RMs showed superior enrichment levels in the 11 of the 14 Gene Ontology categories related to the function. Some categories that have less than five genes are omitted.

# 2. Comparing with EPM#12 and GRAM

## 1) Ribosomal biogenesis; nitrogen depletion EPM#12

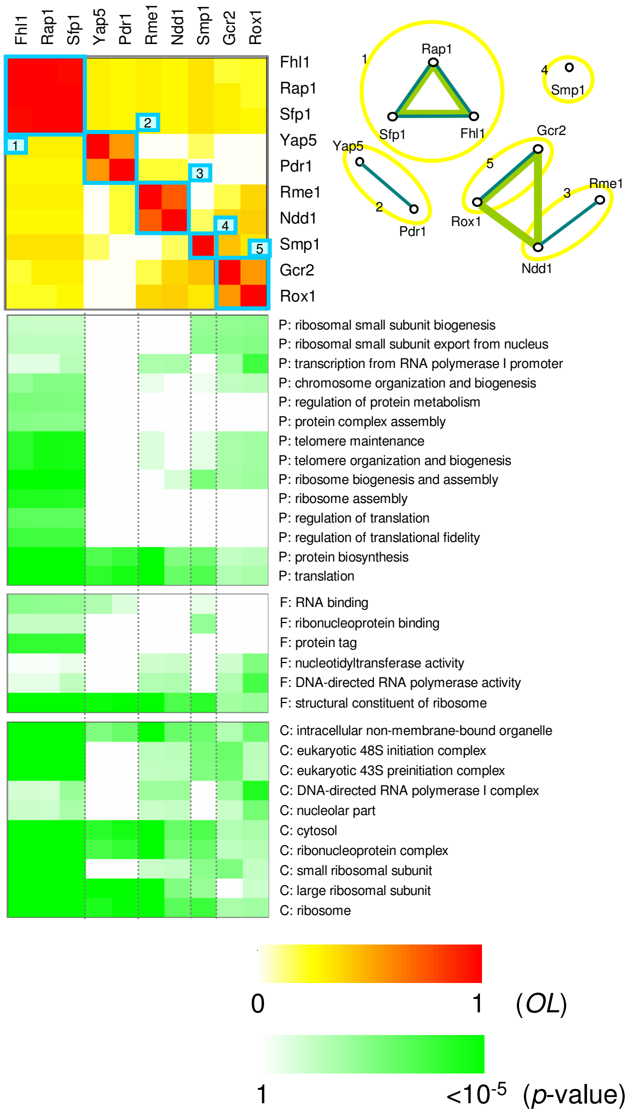


**Figure A13-1. Nitrogen depletion EPM#12 and RMs.**
* P, F, and C are abbreviations for Biological Process, Molecular Function, and Cellular Component of the Gene Ontology categories, respectively

The EPM#12 of nitrogen depletion shows another good example demonstrating the high resolution of our result. This EPM was enriched in the protein biosynthesis and each RM represents functional subpart of the process of ribosomal biogenesis and assembly for the protein biosynthesis. Ribosomes of yeast are composed of ribosomal proteins and ribosomal RNA (rRNA) species, and the process of generating the ribosomes can be divided into several subparts: rRNA synthesis, ribosomal proteins generation, and their assembly. rRNAs are transcribed by RNA polymerase I, and these rRNAs are further matured and constitute ribonucleoprotein by associating with various ribosomal proteins. Through assembly of the ribonucleoproteins with additional ribosomal proteins, ribosome biogenesis is completed. All these detailed processes for ribosome biogenesis were represented in our result. The member genes of the EPM#12 decomposed into five subparts; (1) RM#5; primary rRNA synthesis by RNA polymerase I, (2) RM#4; the conversion of a primary rRNA transcript into a mature rRNA molecule, (3) RM#1 + RM#5; ribosomal small subunit biogenesis, (4) RM#1 +RM#2 +RM#3; ribosomal large subunit biogenesis, and (5) RM#1; assembly of the ribosome subunits and auxiliary ribosomal proteins (Figure A13-1).

First, the member RM#5 were enriched in 'P: transcription from RNA polymerase I promoter' and 'C: nucleolar part', suggesting that RM#5 is related to the process of rRNA synthesis in the nucleous component [134]. Second, RM#4 was enriched in 'F: ribonucleoprotein binding', reflecting that pre-rRNAs are processed by RNA binding of ribosome biogenesis factors (ribonucleoprotein) to produce mature rRNAs [135]. Third, RM#1 and RM#5 genes were enriched in 'C: small ribosomal subunit', whereas RM#1, #2 and #3 were enriched in 'C: large ribosomal subunit'. It seems that they might reflect the process that, mature rRNAs are combined with the ribosomal proteins to form ribosomal subunit [135]. Finally, only RM#1 genes were enriched in 'P: ribosome assembly', which implies that they are involved in aggregation and assembly of generated ribosomal subunits.

The regulators of RM#1, i.e., Rap1, Fhl1, and Sfp1, which are well-known for ribosome biogenesis [120, 136, 137], acted as pivotal regulators for the whole ribosome biogenesis process in our result. And our result suggests that the regulators of RM#5 (Gcr2 and Rox1) would regulate genes involved in rRNA synthesis, although there is no clear literature evidence about that. RM#5 genes were specifically enriched in 'C: RNA polymerase I complex', 'P: transcription for RNA polymerase I promoter', and 'F: DNA-directed RNA polymerase activity' categories, which suggests that the regulators of this RM might have a role in regulation of rRNA transcription by RNA polymerase I. Besides, Yap5, Pdr1, and Smp1 have been reported as ribosome biogenesis-related regulators in other computational approaches [7,138-140].

## 2) Comparing RMs of nitrogen depletion EPM#12 with GRAM

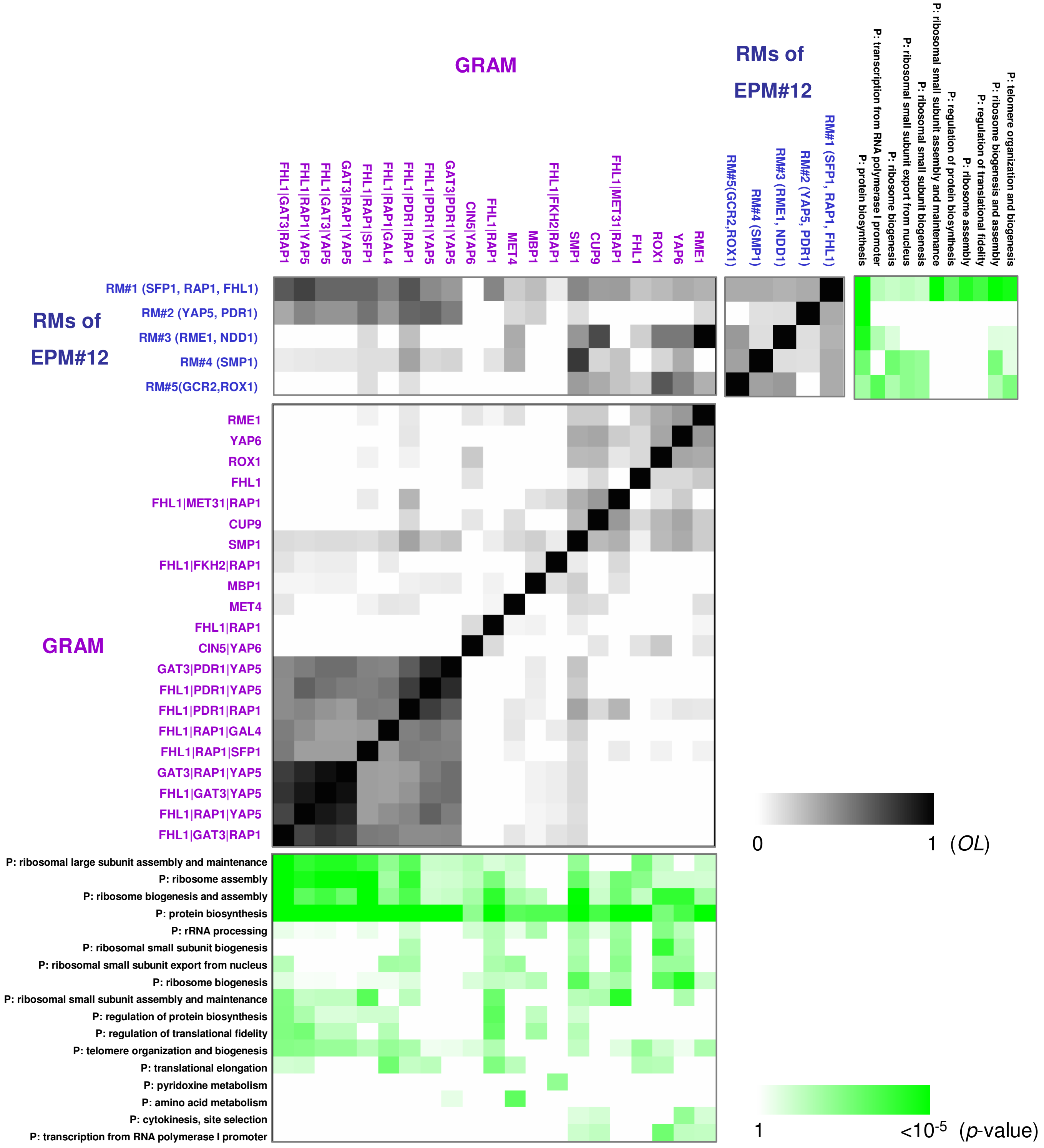


**Figure A13-2. Comparing RMs of nitrogen depletion EPM#12 with GRAM**

To compare the RMs with the modules from GRAM [5], we collected 21 ribosome biogenesis related modules from GRAM. Three overlap matrices are represented by gray. Each entry in the matrix indicates overlap level of genes between regulatory modules. And two Gene Ontology enrichment matrices are represented by green.
