# Supplementary material for: High-resolution analysis of condition-specific regulatory modules in Saccharomyces cerevisiae
Source: Genome Biol. 2008 Jan 3;9(1):R2. doi: 10.1186/gb-2008-9-1-r2 (PMC2395236; doi:10.1186/gb-2008-9-1-r2)
Supplement: Additional data file 14 — Criteria for choosing appropriate cut-off values. [file gb-2008-9-1-r2-S14.pdf]

Additional data 14 Choosing appropriate cut-off values

Cut-offs for defining regulatory modules (PCC cut-off value for defining IMC, and OL cut-off value for defining RM) were confirmed by testing several cut-off values.

1) Choosing an appropriate PCC (Pearson's correlation coefficient) cut-off value for clustering gene expression data

To identify proper PCC cut-off value, we observed the change of gene coverage and functional enrichment level of target genes of transcription factors according to the change of PCC cut-off 0.5 to 0.8, which are generally used by other methods. We calculated average Gene Ontology (GO) enrichment level (-log *p-value*) by using the highest enrichment value per transcription factor target gene set.

As shown in Figure A14-1, the numbers of distinct genes which constitute modules are sharply reduced according to increase in PCC cut-off. However, the GO enrichment level reached plateau at the PCC 0.7. So, PCC 0.7 cut-off value can be considered as an optimal criterion because it improves the functional coherence of the target genes of transcription factors with minimizing the loss of information.

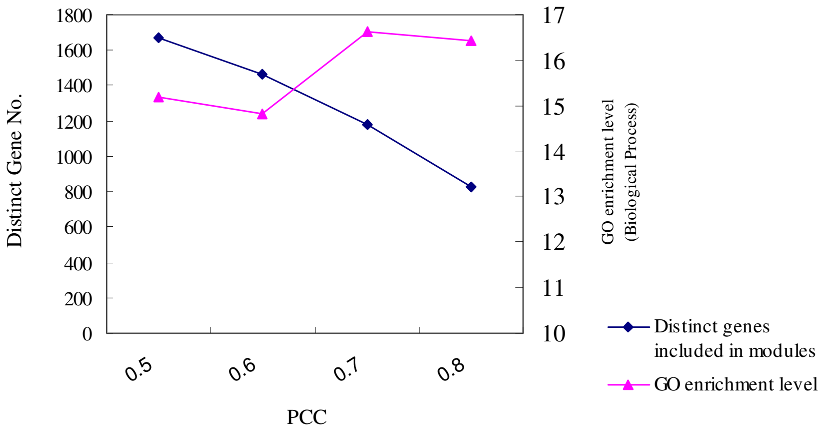

Figure A14-1. Comparing different PCC cut-off values

We also checked the overlap levels of genes between all pair of different PCC cut-off modules to identify genes are maintained according to the change of PCC cut-off value. Most of the genes consisting modules of higher PCC cut-off were retained in the modules generated by lower PCC cut off value (table A14-1). It indicates that our method generates robust results without yielding significant changes according to the change of PCC cut-off value.

	Module gene overlap (min OL)
PCC 0.5 vs PCC 0.6	0.95
PCC 0.5 vs PCC 0.7	0.96
PCC 0.6 vs PCC 0.7	0.97
PCC 0.5 vs PCC 0.8	0.98
PCC 0.6 vs PCC 0.8	0.96
PCC 0.7 vs PCC 0.8	0.98

Table A14-1. Overlap level of genes between different PCC cut-off modules

2) Choosing an appropriate OL (Overlap level)

The purpose of making RM is sub-grouping the TFs of an EPM by checking whether they have significantly overlapped target genes. If the cut-off is too large, we might obtain too many RMs that can cause unnecessary redundancy. Reversely, if the cut-off value is too small, we could get RMs which might not be separated enough for explaining detailed sub-parts of EPMs in terms of function and a number of similar RMs might be generated because of the increasing number of possible TF combinations. So, we searched for the cut-off value which makes RMs the most informative without unnecessary redundancy. To select appropriate OL cut-off value for making RM, we observed the change of the diversity of enriched Gene Ontology categories. And we also checked redundancy level of genes in each EPM by calculating average number of RMs which include a specific gene. As shown in the Figure A14-2, The number of enriched GO categories reached plateau at OL cut-off from 0.4 to 0.7, and the redundancy of RMs are commonly minimized from 0.5 to 0.8. So, the optimal cut-off values can be determined from 0.5 to 0.7. Finally, we chose OL 0.5 where the number of RMs are the smallest and also exhibit sufficient functional diversity and low redundancy

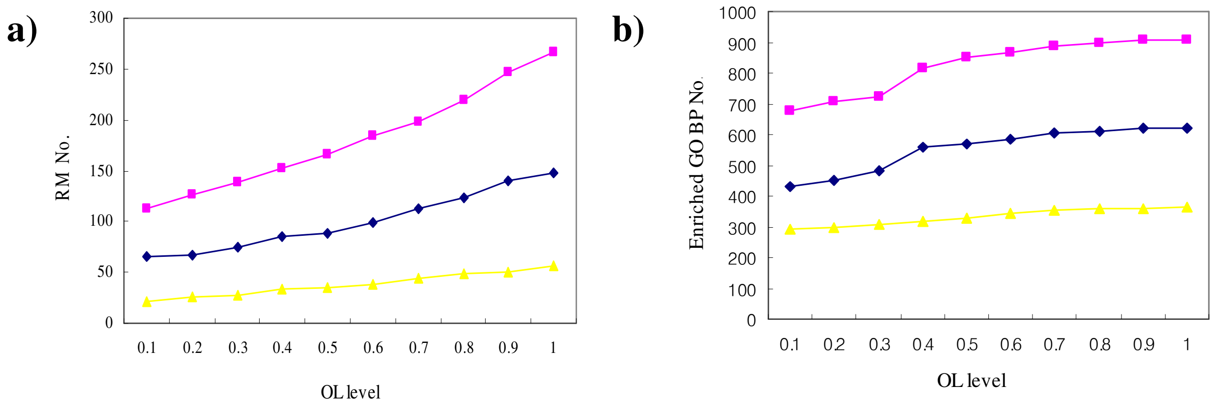

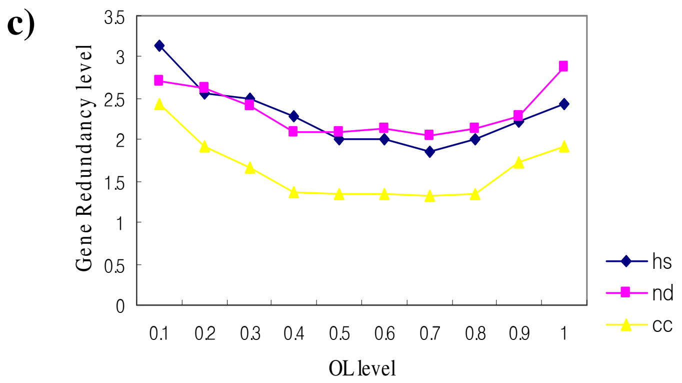

Figure A14-2. Comparison with RMs that are constructed by different OL cut-off values in a) RM number, b) enriched GO BP categories number and c) gene redundancy level. (hs; heat shock, nd; nitrogen depletion, cc; cell cycle)

3) Choosing an appropriate ChIP-chip pvalue

We selected stringent p-value (0.001) cut-off in ChIP-chip data as Harbison *et al.* [3] suggested.
